# A core outcome set for airway management research

**DOI:** 10.1111/anae.70026

**Published:** 2025-11-07

**Authors:** Jan Hansel, Alexander Fuchs, Benjamin Cornwell, Katherine Haynes, Vinay Tanna, Ahmed Mohamed, Kate Rivett, Gillian Radcliffe, Robert Greif, Tim M. Cook, Kariem El‐Boghdadly, the Airway Terminology and Outcome Measures (ATOM) Collaborators, Andreas Sotiriou, Rosanna Grimes, Vera Bohnenblust, Markus Fally, Ricarda Lippuner, Daniel Perin, David J. Brewster, Sheila N. Myatra, Ross Hofmeyr, Wenxian Li, Guillermo J. Navarro, Gerardo Cortese, Sandeep Sudan

**Affiliations:** ^1^ Division of Immunology, Immunity to Infection and Respiratory Medicine The University of Manchester Manchester UK; ^2^ Critical Care Wythenshawe Hospital, Manchester University NHS Foundation Trust Manchester UK; ^3^ Department of Anaesthesiology and Pain Medicine, Inselspital, Bern University Hospital University of Bern Bern Switzerland; ^4^ Department of Anaesthesia Royal United Hospitals Bath NHS Foundation Trust Bath UK; ^5^ Department of Anaesthesia and Perioperative Medicine Guy's and St Thomas' NHS Foundation Trust London UK; ^6^ Intensive Care Unit, Manchester Royal Infirmary Manchester University NHS Foundation Trust Manchester UK; ^7^ Difficult Airway Society London UK; ^8^ Faculty of Medicine University of Bern Bern Switzerland; ^9^ Bristol Medical School University of Bristol Bristol UK; ^10^ Centre for Human and Applied Physiological Sciences Kings College London London UK

**Keywords:** airway management, core outcome set, patient‐reported outcomes, survey

## Abstract

**Introduction:**

Airway management research has historically incorporated heterogeneous outcome selection and definitions. This impedes evidence synthesis and hinders advances in patient care. We aimed to develop a core outcome set to standardise airway management research and improve outcome reporting.

**Methods:**

We performed a systematic review of outcomes reported in airway management studies to identify candidate outcomes from a random sample of eligible studies. We used a modified Delphi survey of three key stakeholder groups: patients with experience of airway management; clinicians engaged in airway management; and others (including researchers, device manufacturers and policymakers). Outcomes were classified into five domains: procedural effectiveness; physiological parameters; adverse events; patient‐reported outcomes; and other. Outcomes were presented in random order in two sequential virtual Delphi surveys and rated by each respondent. Candidate outcome measurement instruments were identified through included studies and targeted literature searches. Subsequently, a core outcome set with suggested outcome measurement instruments was discussed and agreed.

**Results:**

The systematic review identified 4368 studies, with 68 candidate outcomes longlisted from a random sample of 100 studies. Rounds one and two Delphi surveys were completed by 453 and 155 participants, respectively, from six continents. First and second virtual panels were attended by 62 and 44 participants, respectively. The final agreed core outcome set included 11 outcomes: death; cardiac arrest; serious complications; pulmonary complications; neurological complications; airway trauma; unrecognised oesophageal intubation; hypoxaemia; first attempt success without complications; overall success without complications; and difficult airway. Proposed outcome measurement instruments were agreed for all included outcomes.

**Discussion:**

We present the first consensus‐based core outcome set for airway management research, with agreed definitions and measurement instruments. Incorporation of these outcomes into future airway management studies will harmonise evidence synthesis and improve the translatability of findings to improve clinical care.

## Plain Language Summary

Patients often have airway management for a planned general anaesthetic or as a result of a medical emergency. Procedures or devices are used to make sure that oxygen is safely delivered to the lungs at all times. Research studies of airway management look at different outcomes to see if a treatment works well and is safe. An outcome is a way of measuring how well a treatment works or how it affects a patient's health. An outcome might be something patients can feel, like pain, or something that can be seen by a healthcare professional, such as wound healing, or a test result, such as a blood pressure reading. The aim of the Airway Terminology and Outcome Measures (ATOM) project was to find the most important outcomes for airway management studies and agree how to define them. We looked at relevant studies to see what outcomes were reported in the past. We then used two surveys to ask patients with personal experience of airway management, as well as healthcare professionals and researchers, which outcomes were the most important to them. After the surveys, two online meetings with patients and airway management experts took place to agree on the final 11 outcomes. These related to patient safety and other clinical procedures and the best methods of measuring them. We hope that future studies will use these outcomes to help healthcare professionals improve patient care.

## Introduction

Airway management‐related complications contribute significantly to patient harm and litigation [1, 2]. The 7th National Audit Project (NAP7) established that airway complications account for one in eight peri‐operative cardiac arrests [3]. Litigation related to airway management makes up 9% of all claims against anaesthetists and 31% of those relating to death [2]. Complications of airway management procedures undertaken outside operating theatres are more common and associated with poorer outcomes [4]. Consequently, there is considerable interest in methods to prioritis advances in airway safety; new technology adoption; and direct new evidence generation.

Historically, studies that evaluate airway management have used heterogeneous outcomes and definitions [5, 6]. Reporting inconsistencies hamper the interpretation of literature and evidence synthesis. Previous systematic reviews have identified considerable variation in reported outcome measures, in both interventional and observational studies related to airway management, and highlighted the need for unification of terminology and outcome reporting [6, 7]. The 2024 World Health Organization Guidance for best practices for clinical trials proposes, as a key pillar of high‐quality trial design, the choice of relevant outcome measures [8]. Within this framework, it is recommended that core outcome sets should be considered for all trials [8].

A core outcome set is a consensus‐based, standardised, minimum collection of outcomes to measure and report a specific area of health [9]. This approach aims to define a set of outcomes that should be included in all relevant studies and present standardised methods to capture and describe these outcomes, known as outcome measurement instruments. The Core Outcome Measures for Perioperative and Anaesthetic Care (COMPAC) group previously developed a core outcome set for trials in peri‐operative care [10, 11]. Whereas this outcome set is relevant for peri‐operative trials in the broader sense, there is currently no agreed core outcome set for airway management research [6]. Furthermore, numerous domains of airway management, including but not limited to procedures, situations and devices, continue to lack an agreed and consistent terminology framework. With these considerations in mind, we established the airway terminology and outcome measures (ATOM) project with the aim of producing a multi‐stakeholder core outcome set for interventional and observational research related to airway management in adults, applicable to any setting where airway management takes place.

## Methods

Ethical approval was obtained prospectively. The study design followed recommended methodology by the Core Outcome Measures in Effectiveness Trials (COMET) initiative (online Supporting information Appendix S2) with the full protocol published and endorsed by major international airway management societies (online Supporting Information Appendix S3) [12].

We conducted a systematic review to identify outcomes reported in airway management studies. We updated an existing published systematic review search strategy [6] to identify interventional and observational airway studies conducted in humans from 1 January 2006 to 15 November 2023 (online Supporting Information Appendix S4). We included completed or ongoing randomised controlled trials and observational studies where the intervention of interest included any airway management technique. We excluded manikin studies; case reports; case series; conference abstracts; non‐English language publications; and studies involving children. Search results were screened independently by two reviewers at title and abstract level using Covidence screening software (Melbourne, VIC, Australia) [13]. Lack of agreement at the screening stage was discussed between the reviewers initially, and where agreement was not found, this was escalated to a third senior reviewer.

Outcome and outcome measurement instrument data were extracted into a piloted spreadsheet. Two reviewers extracted the first 10% of studies independently. For the remaining studies, one of the reviewers extracted the data, with a second reviewer verifying extractions. Due to the high number of records identified during scoping searches, we restricted outcome extraction to a subset of studies identified through random sampling, without replacement, at a ratio of 3:1 for interventional and observational study designs, respectively. A data saturation approach was adopted, where data from 100 studies were extracted initially, followed by a further 10 studies until saturation was reached. Saturation was defined as no new outcome being identified from a set of studies by two independent reviewers [14]. Sampling was followed by full‐text screening to ensure studies fulfilled eligibility criteria. Any studies excluded at this stage were replaced by a further random sample of records. The following data were extracted: study type; study population; first author; year of publication; journal of publication; interventions under investigation; each verbatim outcome reported from the study abstract, methods or results; outcome definition; whether the outcome was reported as primary or secondary, the outcome measurement instrument; and time‐points or time period at which each outcome was measured.

We followed the methodology described by Young et al. [15] to generate a long list of outcomes, mapped against a 38‐domain taxonomy proposed by the COMET initiative [16]. Exact matching items were deduplicated, with the remaining outcomes grouped for further deduplication depending on the presence of spelling, meaning and context variation by two reviewers and checked by a third senior reviewer. Outcomes were further classified into five domains relevant to airway management: procedural effectiveness; physiology; adverse events; patient‐reported outcomes; and other. The latter category consisted of outcomes that were not possible to classify into the four specified categories.

After outcome extraction, consensus was sought between a range of stakeholders using a modified Delphi process. This involved two rounds of rating outcomes followed by two consensus meetings to agree outcomes and outcome measurement instrument. We aimed to minimise attrition between rounds, in line with COMET standards [9]. Invitations for participation were sent through multiple channels, including electronic communications via international airway management societies; newsletter; e‐mail; promotion at conferences; and social media. We targeted patients with personal experience of airway management in the elective or emergency setting; clinicians engaged in airway management; and other key stakeholders, including researchers with peer‐reviewed publications related to airway management, device manufacturers, guideline developers, journal editors and others (Table 1). Patients with personal experience were recruited from clinical settings, pre‐ and postoperatively while in the hospital, and following ICU admission (online Supporting Information Appendix S5). We aimed to recruit a minimum international sample of 30 patients; 50 clinicians engaged in airway management; and 40 other key stakeholders, including researchers.

**Table 1:** Stakeholder groups.

Stakeholder group	Eligibility criteria
Patients	People who have undergone elective or emergency airway management previously or are about to undergo airway management for surgery in the elective or emergency setting.
Clinicians engaged in airway management	Anaesthetists; intensivists; emergency physicians; paramedics; operating department practitioners; anaesthetic nurses; surgeons.
Other stakeholders	Researchers, who may also be clinicians, active in the field of airway management with at least five publications in a peer‐reviewed journal; representatives of funding bodies; guideline developers; journal editors; industry representatives; inventors.

Surveys for both Delphi rounds were built and distributed using REDCap (Research Electronic Data Capture) software [17]. Participants were provided with guidance on survey completion and shown an animated video [18], co‐produced with a lay representative and translated into English, Spanish, Italian, German and French to improve engagement, followed by written electronic consent to participate. Participants were then asked to rate each outcome individually on a scale from 1 to 9, following GRADE guidance [9, 19, 20]. Scores between 1 and 3 signified outcomes of limited importance; 4–6, an important but not critical outcome; and 7–9, a critical outcome [21]. Outcomes rated critical (7–9) by ≥ 70% and of limited importance (1–3) by ≤ 15% of participants in each stakeholder group were selected for inclusion. Outcomes rated critical (7–9) by ≤ 50% of participants in each stakeholder group were not included. Both the display of outcome domains and individual outcomes within them were randomised to mitigate ‘order presentation bias’. Participants were invited to provide general comments and suggest any additional outcomes that may have been missed. Outcomes were reviewed by the steering committee between Delphi rounds, with new unique proposed outcomes included in the second round per our a priori published protocol [12].

For each outcome, detailed definitions were prepared and outcome measurement instruments proposed. Outcome measurement instruments were identified from included studies, with frequencies summarised. If no outcome measurement instruments were identified from included studies, a targeted literature search was performed for each outcome. Outcomes with similar context and meaning were grouped and harmonised, with oversight from the steering committee.

Following the survey rounds, we convened two virtual consensus panels, during which outcomes, their definitions and outcome measurement instruments were discussed, voted on and agreed. All participants who completed the second survey were invited to attend the panel. To maximise patient engagement, we extended invitations to all patients who completed the first survey. We sent a digital information pack to each prospective participant a week before the virtual panel. This contained information on outcome definitions; proposed measurement instruments; and voting results from previous rounds, along with indicative views of the steering committee. We did not collect stakeholder category information at this stage.

During the first virtual panel, all proposed outcomes not removed in the previous rounds were displayed sequentially, with time allocated for discussion, followed by real‐time voting to either ‘include’ or ‘exclude’. Due to the large number of outcomes proceeding to this stage, a threshold of ≥ 75% agreement was set for the inclusion of an outcome in the final core outcome set. The second virtual panel was dedicated to selecting outcome measurement instruments for all included outcomes. These were presented to attendees, with the voting option of ‘agree’ or ‘disagree’. Similar to the previous round, ≥ 75% was required for an instrument to receive a strong recommendation. Where 51–74% of participants voted in favour, the instrument was adopted as an interim recommendation, with further research required; where ≤ 50% of participants agreed on the instrument, no recommendation was made [9].

Descriptive statistics were used to summarise voting responses for each outcome across stakeholder groups and rounds. Differences in rating distributions were explored visually using bar plots, summary tables and hierarchical heat maps. No inferential statistical tests were performed. All analyses and visualisations were performed in R Statistical Software (v4.4.2; R Core Team 2024, Vienna, Austria).

## Results

Searches were conducted on 16 November 2023, with 72,373 new records identified before deduplication. Following deduplication, 60,023 records underwent title and abstract screening, resulting in 2863 records considered for inclusion. These records were combined with 1505 records identified in the previous systematic review to form the pool of 4368 eligible studies (online Supporting Information Figure S1).

We reached data saturation at 669 verbatim outcomes extracted from 100 studies, with no new outcomes emerging on further review of an additional 10 studies. Outcomes from procedural effectiveness (n = 305), physiology (n = 127) and adverse events (n = 122) domains were reported most frequently. Verbatim outcomes were deduplicated and amalgamated where appropriate, resulting in 68 outcomes longlisted for subsequent consensus seeking. The full list of outcomes with amalgamations and domain classifications is presented in online Supporting Information Table S1, with the list of studies contributing data in online Supporting Information Appendix S6 and longlisted outcomes in online Supporting Information Table S2.

The first Delphi survey of 68 outcomes was open between 20 November 2024 and 9 February 2025, with 453 unique respondents from a range of clinical and nonclinical backgrounds completing the survey in full (Table 2). Ten outcomes were included at this stage, with seven outcomes omitted. The remaining 51 outcomes did not achieve consensus and proceeded to the subsequent round. Seven additional outcomes were proposed by survey respondents. The second Delphi survey was open between 21 February 2025 and 1 April 2025, with 155 unique respondents completing the survey in full, rating 58 outcomes; 24 were included in this stage, with two outcomes omitted. No consensus was achieved for the remaining 32 outcomes in this round.

**Table 2:** Characteristics of Delphi survey participants.

	Clinicians*	Patients^†^	Others^‡^	All
	n = 328	n = 67	n = 58	n = 453
	(72%)	(15%)	(13%)	
Interests disclosed	11 (3%)	2 (3%)	8 (14%)	21 (5%)
Completed second round	110 (34%)	15 (22%)	30 (52%)	155 (34%)
Sex; female	119 (36%)	33 (49%)	14 (24%)	166 (37%)
Region				
Europe	138 (42%)	60 (89%)	32 (55%)	230 (51%)
Oceania	86 (26%)	1 (2%)	8 (14%)	95 (21%)
North America	50 (15%)	‐	8 (14%)	58 (13%)
Asia	31 (10%)	4 (6%)	8 (14%)	43 (9%)
South America	15 (5%)	1 (2%)	‐	16 (4%)
Africa	8 (2%)	1 (2%)	2 (3%)	11 (2%)
Experience setting				
Operating theatre	265 (81%)	62 (92%)	53 (91%)	380 (84%)
ICU	17 (5%)	4 (6%)	4 (7%)	25 (6%)
Emergency department	24 (7%)	1 (2%)	1 (2%)	26 (6%)
Prehospital	22 (7%)	‐	‐	22 (5%)
Economy^§^				
High‐income	285 (87%)	61 (91%)	48 (83%)	394 (87%)
Upper‐middle income	34 (10%)	3 (5%)	6 (10%)	43 (10%)
Lower‐middle income	8 (2%)	3 (5%)	4 (7%)	15 (3%)
Not classified	1 (< 1%)	‐	‐	1 (< 1%)

* Clinicians include physicians (n = 281), nurses (n = 17) and other healthcare personnel (n = 30) involved in airway management.

^†^ Patients include patients (n = 65) and relatives (n = 2) with experience in airway management.

^‡^ Others include researchers (n = 46), journal editors (n = 4), policymakers and regulators (n = 1) and others (n = 7) involved in airway management.

^§^ Economy of participant country according to the World Bank classification 2024.

**Figure 1:**
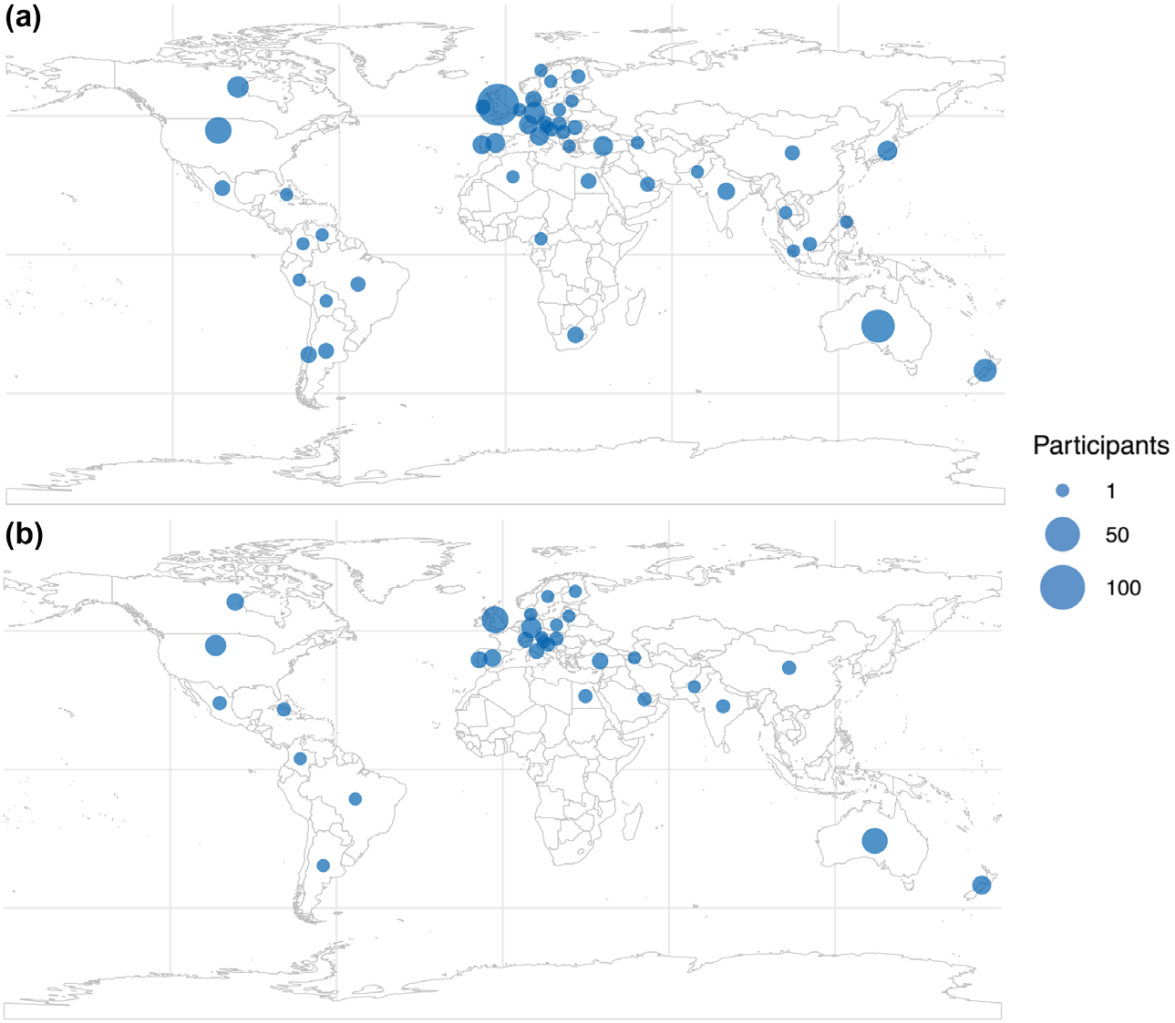
Global distribution of participants in (a) round 1 (n = 453) and (b) round 2 (n = 155) of the Delphi survey.

The global distribution of participants in both surveys is shown in Fig. 1. Detailed voting results for both surveys according to stakeholder group are reported in online Supporting Information Figures S2–S4. Following the two surveys, outcomes were reviewed and amalgamated before virtual consensus panel voting, resulting in a total of 44 outcomes for further discussions (online Supporting Information Table S3). The first virtual consensus panel meeting took place on 24 June 2025 and was attended by 62 participants. Forty‐four outcomes were assessed, with 12 included in the final core outcome set, with ≥ 75% agreement (online Supporting Information Table S4). A vote to merge ‘pulmonary aspiration’ with ‘pulmonary complications’ was taken at the second panel to align with previous definitions of pulmonary complications, yielding a total of 11 outcomes [22].

The second virtual consensus panel meeting took place on 15 July 2025 and was attended by 44 participants. Outcome measurement instruments for 11 outcomes were discussed, with additional voting on aspects of outcome definitions taking place (online Supporting Information Tables S5 and S6). Two of 11 outcome measurement instruments achieved weak consensus (cardiac arrest and pulmonary complications), with the remaining instruments supported by ≥ 75% participants. Three of 15 definition statements achieved weak consensus, with one statement relating to the scale and threshold used for difficult laryngoscopy not achieving consensus (49%, Table 3).

**Table 3:** Core outcome set for airway management research.

Outcome	Detailed definition	Outcome measurement instrument
Death	Airway‐related mortality.	Death certificate or hospital record; no single time point proposed.*
Cardiac arrest	A sudden cessation of function of the heart requiring delivery of five or more chest compressions and/or defibrillation within 24 h of airway management event.	Need for five or more chest compressions and/or defibrillation.^†^
Serious complications	An adverse event that results in death, is life‐threatening, requires hospitalisation or prolongation of existing hospitalisation, results in persistent or significant disability or incapacity or is a birth defect.	No single outcome measurement instrument proposed.*
Pulmonary complications	Composite outcome of any of the following: ‐ atelectasis; ‐ pulmonary aspiration; ‐ acute respiratory distress syndrome.	‐ Atelectasis: detected on imaging (e.g. computed tomography, chest radiograph, ultrasound). ‐ Pulmonary aspiration: clinical, radiological or bronchoscopic evidence of entry of gastric contents into trachea and/or lungs. ‐ Acute respiratory distress syndrome: diagnosed using the 2023 definition [23].^†^
Neurological complications	Composite outcome of any new brain damage or spinal cord injury.	‐ Brain damage: confirmed evidence of hypoxic‐ischaemic brain injury following procedure. ‐ Spinal cord injury: confirmed clinical diagnosis.*
Airway trauma	Injury to any area of the nasal cavity; nasopharynx; oropharynx; pharynx; glottis; subglottis; or trachea.	No single outcome measurement instrument proposed.*
Unrecognised oesophageal intubation	Oesophageal placement of a tracheal tube not identified by absence of sustained exhaled carbon dioxide or leading to a complication.	No single outcome measurement instrument proposed.*
Hypoxaemia	Defined as oxygen saturation < 90%, with severe hypoxaemia defined as oxygen saturation < 80%.	Measured with continuous pulse oximetry for at least 30 min following commencement of study intervention. Record the lowest values. Report episodes of hypoxaemia (S_p_O_2_ < 90%) and severe hypoxaemia (S_p_O_2_ < 80%).*
First attempt success without complications	Composite outcome of first attempt success (successfully established airway following first instrumentation with device and/or adjunct within 120 s confirmed with capnography) and absence of complications related to airway management.	Record whether the airway was successfully established following first instrumentation with device and/or adjunct within 120 s with capnography confirmation, in the absence of any complications related to airway management.*
Overall success without complications	Composite outcome of overall success (successfully established airway within three attempts confirmed with capnography), and absence of complications related to airway management.	Record whether the airway was successfully established within three attempts with capnography confirmation, in the absence of any complications related to airway management.*
Difficult airway	Composite outcome of any of the following: ‐ difficult facemask ventilation; ‐ difficult supraglottic airway ventilation; ‐ difficult laryngoscopy; ‐ difficult tracheal intubation; ‐ difficult front‐of‐neck airway; ‐ emergency front‐of‐neck airway.	For any participant undergoing airway management under optimised conditions (operator, position, device), record and report each of the following: ‐ difficult facemask ventilation, classified as Grade C or D (Lim classification) [24]; ‐ difficult supraglottic airway ventilation, defined as at least one failed attempt at supraglottic airway insertion; ‐ difficult laryngoscopy, with no single threshold agreed; ‐ difficult tracheal intubation, defined as at least one failed attempt at tracheal intubation; ‐ difficult front‐of‐neck airway, defined as at least one failed attempt at front‐of‐neck airway; ‐ need for emergency front‐of‐neck airway.*

* Strong recommendation.

^†^ Interim recommendation – further research required.

The final core outcome set consists of 11 outcomes from two domains (Table 3): adverse events (death; cardiac arrest; serious complications; pulmonary complications; neurological complications; airway trauma; unrecognised oesophageal intubation; hypoxaemia) and procedural effectiveness (first attempt success without complications; overall success without complications; difficult airway). Detailed background information is in online Supporting Information Table S7 with plain language explanations of outcomes in online Supporting Information Table S8.

## Discussion

We present, to our knowledge, the first international, consensus‐based core outcome set for airway management research. This comprises 11 outcomes deemed critically important by a broad range of stakeholders, including patients, clinicians and researchers, with airway management experience in various clinical and geographical settings. In addition to prioritising outcomes across procedural and adverse event domains, the ATOM collaboration has proposed standardised definitions and associated measurement instruments to enhance consistency in future airway management studies. Crucially, this outcome set was developed through a transparent, methodologically rigorous process, aligned with COMET standards and GRADE methodology, and supported by extensive patient and public involvement. The resulting framework lays the foundation for improved synthesis, comparability and translation of research findings in airway management, with the potential to influence trial design meaningfully, funding prioritisation, guideline development and, ultimately, improving patient care.

The aim of a core outcome set is to define the minimum set of outcomes that should be reported in all studies within a given field. Importantly, this is not designed to limit researchers from exploring additional relevant questions; investigators are encouraged to report supplementary outcomes that align with specific study aims, populations or interventions, provided that the core outcomes are also included [9]. A core outcome set aims to provide a consistent foundation to facilitate meta‐analysis and evidence synthesis, while allowing space for innovation and evolution of outcome frameworks. In areas such as airway management, where clinical, technical and contextual variability are substantial, this flexibility is critical. Accordingly, we emphasise that the ATOM core outcome set should be viewed as a foundational framework, not a prescriptive checklist. Ongoing refinement of both outcomes and measurement instruments will likely be required, particularly as technologies, procedures and care settings evolve. Concurrently with the dissemination of this core outcome set, the methodology of the ATOM project will be used as a framework to deliver broadly agreed airway taxonomy in future work packages.

The ATOM core outcome set prioritises outcomes that reflect the concerns and personal experiences of patients, including complications and adverse events with potentially life‐altering consequences. We endeavoured to integrate patient voices meaningfully throughout the process, with a lay member of the steering committee present at all meetings and contributing to developing the project, from conception to completion. This helped ensure that patient perspectives were not only included but shaped the framing and interpretation of candidate outcomes. In the early stages of the consensus‐seeking process, we noted a considerable discrepancy between clinician and patient views; clinicians assigned higher importance to more technical outcomes, which patients did not consider critical. Previously published observational snapshots, such as the first Sprint National Anaesthesia Project (SNAP‐1), have described patient peri‐operative journeys using a multimodal approach. Here, patient‐reported outcomes are assessed at multiple time points with routine collection of safety data, patient experience and outcomes [25]. Many of the included outcomes in the ATOM core outcome set, such as death, cardiac arrest and neurological injury, reflect known priorities for patients and their families and align with established domains of peri‐operative and critical care experience [10, 26]. Importantly, the inclusion of procedural success only when complication‐free reflects a patient‐centred framing that is more useful than technical success in isolation. This core outcome set aligns with the wider movement within anaesthesia, surgery and critical care to develop procedure‐specific outcome frameworks, such as those emerging in critical care research [26, 27].

There were several areas that failed to achieve consensus. For example, cardiac arrest was deemed an important outcome to report, but the uncertainty regarding the temporal relationship between this event and airway management was responsible for weak consensus on the definition of timing (52%). While cardiac arrest within 24 h is a timeframe in keeping with NAP7 [28], some participants felt this to be too long to associate causality with airway management. A weak consensus (71%) was achieved for the decision to amend the definition of ‘complications’ to ‘serious complications’ to align it with routine serious adverse event reporting in research. This may reflect complexities of including harm outcomes in core outcome sets described previously, as inclusion of such outcomes requires a tailored assessment [29].

While the first attempt success without complications had strong consensus as an outcome, agreement on time to perform this procedure had weak consensus in favour of 120 s rather than 90 s. Participants highlighted that 120 s was a long duration of time for an airway management procedural attempt; however, this accounts for the time taken for confirmation of successful device placement with capnography, with a proviso that this is not a target, but rather a maximum duration of time. Furthermore, whereas a shorter duration of airway management procedures may be desirable clinically, the threshold agreed in an outcome measurement instrument represents a cut‐off for the purposes of research.

An additional argument raised by panellists was the relative redundancy of a maximum time point when complications are accounted for, as both serve as safety caps. Finally, we could not achieve consensus on the instrument to define difficult laryngoscopy, be that modified Cormack and Lehane ≥ 2b or no vocal cords visible [30, 31]. This remains an area that requires further research, as the view at laryngoscopy can be reported using various tools and difficulty can be defined according to several possible thresholds, which may arguably differ depending on the tracheal intubation device. Regardless, difficult laryngoscopy should still be reported in airway management research, with investigators choosing the most appropriate scale for the context, but an agreed instrument and threshold remain to be standardised.

Incorporating this core outcome set into research practice may serve several purposes. Trialists, guideline developers and funders are encouraged to adopt the outcome set in funding applications; study protocols; and ethics submissions. The outcome set can also serve as a framework for prospective observational research, quality improvement initiatives and registry‐based studies. Implementation may be supported by the availability of standardised definitions and suggested outcome measurement instruments, which enhance reproducibility and facilitate comparison across studies. To promote uptake and adherence, we recognise the importance of a formal dissemination strategy that extends beyond publication. In line with COMET guidance, we will encourage uptake of the ATOM core outcome set into future clinical trial protocols, registry designs and funding calls by engaging early with relevant stakeholders such as research funders, ethics committees, trial networks and journal editors. Additionally, harmonised outcome reporting may inform audit standards, guideline development and communication with patients. The growing emphasis on outcome‐based quality metrics in peri‐operative and urgent care underscores the clinical value of a common, stakeholder‐informed outcome framework. This strategy is consistent with established COMET implementation approaches to encourage sustainable adoption of core outcome sets in diverse research and practice settings.

The study has limitations. First, although we employed rigorous methodology in line with COMET guidance, our systematic search was restricted to English‐language publications, potentially omitting relevant outcomes reported in studies published in other languages. Second, the outcome extraction process, while designed to achieve data saturation, was limited to a random subset of eligible studies due to the high volume of articles identified. As a result, rare or emerging outcomes may have been underrepresented. Third, although efforts were made to recruit a diverse and international panel of stakeholders, through open calls and targeted outreach, certain regions and professional groups remain underrepresented, such as those from low‐ and middle‐income countries, with most patient participants from the United Kingdom. Fourth, despite predefined eligibility criteria and structured consensus methods, the Delphi process and virtual consensus panels are inherently susceptible to response bias and group dynamics. Fifth, the panel could not agree on a consensus definition for difficult laryngoscopy; this was a potential drawback in the methodology of the proposed voting options, as no single definition reached the prespecified threshold. As such, work to define investigative thresholds for difficulty with glottic view represents a future avenue for further consensus exercises in airway management research. Sixth, we noted considerable attrition between the first and second rounds of the survey, specifically in the patient stakeholder group. We mitigated the impact of this attrition prospectively through deliberate inclusion of high participant numbers in the first round, anticipating likely temporality of patient reviews to the time of hospital admission and procedure. Finally, while outcome measurement instruments were proposed and rated by stakeholders, their validity, feasibility and measurement properties require further evaluation in prospective studies across clinical and research settings (online Supporting Information Appendix S7).

In conclusion, we present the first consensus‐based core outcome set for airway management research. Included outcomes (death; cardiac arrest; serious complications; pulmonary complications; neurological complications; airway trauma; unrecognised oesophageal intubation; hypoxaemia; first attempt success without complications; overall success without complications; and difficult airway) that are important to patients, clinicians, researchers and other key stakeholders across a range of healthcare settings and environments. The overarching aim of the ATOM core outcome set is to facilitate a research agenda that emphasises what matters to both patients and clinicians. Researchers can select outcomes for both interventional and observational research, whereas policymakers, funders and other stakeholders may use them as a common language for knowledge translation in anaesthesia and airway management. While this outcome set is not exhaustive, the long‐term objective is for standardised adoption in all future airway management research.

## Supporting information


**Plain Language Summary**.

**Appendix S1.** Steering committee, associate principal investigators and collaborators.
**Appendix S2.** COS‐STAR checklist.
**Appendix S3.** Endorsing airway management societies.
**Appendix S4.** Search strategy.
**Appendix S5.** Participant eligibility criteria.
**Appendix S6.** Studies contributing data.
**Appendix S7.** Proposed research agenda.

**Figure S1.** Modified PRISMA flow diagram.
**Figure S2.** Individual outcome voting results grouped by stakeholder group (round 1).
**Figure S3.** Individual outcome voting results grouped by stakeholder group (round 2).
**Figure S4.** Summary of votes according to stakeholder group in rounds 1 and 2.

**Table S1.** Extracted outcomes.
**Table S2.** Longlisted outcomes.
**Table S3.** Modifications to outcomes following survey rounds.
**Table S4.** Virtual consensus panel voting results for individual outcomes.
**Table S5.** Voting results on proposed modifications to included outcomes.
**Table S6.** Virtual consensus panel voting results for outcome measurement instruments.
**Table S7.** Detailed information on outcomes and outcome measurement instruments.
**Table S8.** Plain language summary of included outcomes.

## References

[1] Cook TM , Scott S , Mihai R . Litigation related to airway and respiratory complications of anaesthesia: an analysis of claims against the NHS in England 1995–2007. Anaesthesia 2010; 65: 556–563. 10.1111/j.1365-2044.2010.06331.x. 20345420

[2] Oglesby FC , Ray AG , Shurlock T , Mitra T , Cook TM . Litigation related to anaesthesia: analysis of claims against the NHS in England 2008–2018 and comparison against previous claim patterns. Anaesthesia 2022; 77: 527–537. 10.1111/anae.15685. 35247933

[3] Kane AD , Soar J , Armstrong RA , et al. Patient characteristics, anaesthetic workload and techniques in the UK: an analysis from the 7th National Audit Project (NAP) activity survey. Anaesthesia 2023; 78: 701–711. 10.1111/anae.15989. 36857758

[4] Cook TM , Woodall N , Frerk C . Major complications of airway management in the UK: results of the fourth National Audit Project of the Royal College of Anaesthetists and the Difficult Airway Society. Part 1: Anaesthesia. Br J Anaesth 2011; 106: 617–631. 10.1093/bja/aer058. 21447488

[5] Hansel J , Rogers AM , Lewis SR , Cook TM , Smith AF . Videolaryngoscopy versus direct laryngoscopy for adults undergoing tracheal intubation. Cochrane Database Syst Rev 2022; 4: CD011136. 10.1002/14651858.CD011136.pub3. 35373840 PMC8978307

[6] Ahmad I , Onwochei DN , Muldoon S , Keane O , El‐Boghdadly K . Airway management research: a systematic review. Anaesthesia 2019; 74: 225–236. 10.1111/anae.14471. 30460982

[7] Fuchs A , Koepp G , Huber M , et al. Apnoeic oxygenation during paediatric tracheal intubation: a systematic review and meta‐analysis. Br J Anaesth 2024; 132: 392–406. 10.1016/j.bja.2023.10.039. 38030551

[8] World Health Organization . Guidance for best practices for clinical trials. 2024. https://iris.who.int/items/27529498‐7904‐404f‐9a53‐e80977c953a8 (accessed 25/9/2025).

[9] Williamson PR , Altman DG , Bagley H , et al. The COMET handbook: version 1.0. Trials 2017; 18: 280. 10.1186/s13063-017-1978-4. 28681707 PMC5499094

[10] Boney O , Moonesinghe SR , Myles PS , Grocott MPW . Core outcome measures for perioperative and anaesthetic care (COMPAC): a modified Delphi process to develop a core outcome set for trials in perioperative care and anaesthesia. Br J Anaesth 2022; 128: 174–185. 10.1016/j.bja.2021.09.027. 34740438

[11] Myles PS , Grocott MP , Boney O , Moonesinghe SR . Standardizing end points in perioperative trials: towards a core and extended outcome set. Br J Anaesth 2016; 116: 586–589. 10.1093/bja/aew066. 27106961

[12] Hansel J , Fuchs A , Radcliffe G , et al. International consensus‐based core outcome set for airway management clinical trials and observational studies: the Airway Terminology and Outcome Measures (ATO) protocol. BMJ Open 2025; 15: e096886. 10.1136/bmjopen-2024-096886. PMC11969595 40180390

[13] Veritas Health Innovation . Covidence systematic review software. www.covidence.org (accessed 22/07/2025).

[14] Glaser BG , Strauss AL . The Discovery of Grounded Theory: Strategies for Qualitative Research. Piscataway, NJ: Transaction Publishers, 1999.

[15] Young AE , Brookes ST , Avery KNL , Davies A , Metcalfe C , Blazeby JM . A systematic review of core outcome set development studies demonstrates difficulties in defining unique outcomes. J Clin Epidemiol 2019; 115: 14–24. 10.1016/j.jclinepi.2019.06.016. 31276780

[16] Dodd S , Clarke M , Becker L , Mavergames C , Fish R , Williamson PR . A taxonomy has been developed for outcomes in medical research to help improve knowledge discovery. J Clin Epidemiol 2018; 96: 84–92. 10.1016/j.jclinepi.2017.12.020. 29288712 PMC5854263

[17] Harris PA , Taylor R , Thielke R , Payne J , Gonzalez N , Conde JG . Research electronic data capture (REDCap)—a metadata‐driven methodology and workflow process for providing translational research informatics support. J Biomed Inform 2009; 42: 377–381. 10.1016/j.jbi.2008.08.010. 18929686 PMC2700030

[18] Airway Terminology and Outcome Measures . Airway terminology and outcome measures video explainer. 2024. https://www.youtube.com/watch?v=APGOnpGmerU (accessed 07/08/2025).

[19] Williamson PR , Altman DG , Blazeby JM , Clarke M , Devane D , Gargon E , Tugwell P . Developing core outcome sets for clinical trials: issues to consider. Trials 2012; 13: 132. 10.1186/1745-6215-13-132. 22867278 PMC3472231

[20] De Meyer D , Kottner J , Beele H , et al. Delphi procedure in core outcome set development: rating scale and consensus criteria determined outcome selection. J Clin Epidemiol 2019; 111: 23–31. 10.1016/j.jclinepi.2019.03.011. 30922885

[21] Remus A , Smith V , Wuytack F . Methodology in core outcome set (COS) development: the impact of patient interviews and using a 5‐point versus a 9‐point delphi rating scale on core outcome selection in a cos development study. BMC Med Res Methodol 2021; 21: 10. 10.1186/s12874-020-01197-3. 33413129 PMC7791855

[22] Abbott TEF , Fowler AJ , Pelosi P , et al. A systematic review and consensus definitions for standardised end‐points in perioperative medicine: pulmonary complications. Br J Anaesth 2018; 120: 1066–1079. 10.1016/j.bja.2018.02.007. 29661384

[23] Matthay MA , Arabi Y , Arroliga AC , et al. A new global definition of acute respiratory distress syndrome. Am J Respir Crit Care Med 2024; 209: 37–47. 10.1164/rccm.202303-0558WS. 37487152 PMC10870872

[24] Lim KS , Nielsen JR . Objective description of mask ventilation. Br J Anaesth 2016; 117: 828–829. 10.1093/bja/aew368. 27956685

[25] Walker EMK , Bell M , Cook TM , Grocott MPW , Moonesinghe SR . Patient reported outcome of adult perioperative anaesthesia in the United Kingdom: a cross‐sectional observational study. Br J Anaesth 2016; 117: 758–766. 10.1093/bja/aew381. 27956674

[26] Needham DM , Sepulveda KA , Dinglas VD , Chessare CM , Friedman LA , Bingham CO III , Turnbull AE . Core outcome measures for clinical research in acute respiratory failure survivors. An international modified Delphi consensus study. Am J Respir Crit Care Med 2017; 196: 1122–1130. 10.1164/rccm.201702-0372OC. 28537429 PMC5694837

[27] Taneri PE , Kirkham JJ , Molloy EJ , et al. Protocol for the development of a core outcome set for neonatal sepsis (NESCOS). PLoS One 2023; 18: e0295325. 10.1371/journal.pone.0295325. 38051733 PMC10697588

[28] Cook TM , Oglesby F , Kane AD , Armstrong RA , Kursumovic E , Soar J . Airway and respiratory complications during anaesthesia and associated with peri‐operative cardiac arrest as reported to the 7th National Audit Project of the Royal College of Anaesthetists. Anaesthesia 2024; 79: 368–379. 10.1111/anae.16187. 38031494

[29] Tay J , Robinson C , Blazeby J , Loke Y , Lowery A , Alkhaffaf B , Kirkham JJ . Inclusion of harm outcomes in core outcome sets requires careful consideration. J Clin Epidemiol 2024; 174: 111474. 10.1016/j.jclinepi.2024.111474. 39038744

[30] Yentis SM , Lee DJH . Evaluation of an improved scoring system for the grading of direct laryngoscopy. Anaesthesia 1998; 53: 1041–1044. 10.1046/j.1365-2044.1998.00605.x. 10023271

[31] Cook TM , Nolan JP , Gabbott DA . Cricoid pressure‐‐are two hands better than one? Anaesthesia 1997; 52: 179–180. 9059107

