# Supplementary material for: A core outcome set for airway management research
Source: Anaesthesia. 2025 Nov 7;81(3):373–82. doi: 10.1111/anae.70026 (PMC12893836; doi:10.1111/anae.70026)
Supplement: Supplementary file 1 — Plain Language Summary. [file ANAE-81-373-s004.docx]

Plain Language Summary

Patients often have airway management for a general anaesthetic or as a result of a medical emergency. Procedures or devices are used to make sure that oxygen is safely delivered to the lungs at all times. Research studies of airway management look at different outcomes to see if a treatment works well and is safe. An outcome is a way of measuring how well a treatment works or how it affects a patient’s health. An outcome might be something patients can feel, like pain, or something that can be seen by a healthcare professional such as wound healing, or a test result, such as a blood pressure reading. The aim of the Airway Terminology and Outcome Measures (ATOM) project was to find the most important outcomes for airway management studies and agree how to define them. We looked at relevant studies to see what outcomes were reported in the past. We then used two surveys to ask patients with personal experience of airway management, as well as healthcare professionals, and researchers, which outcomes were the most important to them. After the surveys, two online meetings with patients and airway management experts took place to agree on the final 11 outcomes. These related to patient safety and other clinical procedures, and the best methods of measuring them. We hope that future studies will use these outcomes to help healthcare professionals improve patient care.
