# Supplementary material for: A core outcome set for airway management research
Source: Anaesthesia. 2025 Nov 7;81(3):373–82. doi: 10.1111/anae.70026 (PMC12893836; doi:10.1111/anae.70026)
Supplement: Supplementary file 2 — Appendix S1. Steering committee, associate principal investigators and collaborators. Appendix S2. COS‐STAR checklist. Appendix S3. Endorsing airway management societies. Appendix S4. Search strategy. Appendix S5. Participant eligibility criteria. Appendix S6. Studies contributing data. Appendix S7. Proposed research agenda. [file ANAE-81-373-s001.docx]

Appendix S1 Steering Committee, Associate Principal Investigators and Collaborators.

**Airway Terminology and Outcome Measures (ATOM) Steering Committee**

Jan Hansel (Principal Investigator); Alexander Fuchs; Kate Rivett; Gillian Radcliffe; Robert Greif; Tim M Cook; Kariem El-Boghdadly (Chief Investigator)

**Airway Terminology and Outcome Measures (ATOM) Associate Principal Investigators**

Katherine Haynes; Benjamin Cornwell; Vinay Tanna; Ahmed Mohamed

**Airway Terminology and Outcome Measures (ATOM) Collaborators**

Andreas Sotiriou; Rosanna Grimes; Vera Bohnenblust; Markus Fally; Ricarda Lippuner; Daniel Perin; David J Brewster; Sheila N Myatra; Ross Hofmeyr; Wenxian Li; Guillermo J Navarro; Gerardo Cortese; Sandeep Sudan

Appendix S2 COS-STAR Checklist

| **SECTION/TOPIC** | **ITEM No.** | **CHECKLIST ITEM** | **LOCATION REPORTED** |
| --- | --- | --- | --- |
| **TITLE/ABSTRACT** | | | |
| Title | 1a | Identify in the title that the paper reports the development of a COS | p. 1 |
| Abstract | 1b | Provide a structured summary | p. 2 |
| **INTRODUCTION** | | | |
| Background and Objectives | 2a | Describe the background and explain the rationale for developing the COS. | p. 3 |
|  | 2b | Describe the specific objectives with reference to developing a COS. | p. 3 |
| Scope | 3a | Describe the health condition(s) and population(s) covered by the COS. | p. 3 |
|  | 3b | Describe the intervention(s) covered by the COS. | p. 3 |
|  | 3c | Describe the setting(s) in which the COS is to be applied. | p. 3 |
| **METHODS** | | | |
| Protocol/Registry Entry | 4 | Indicate where the COS development protocol can be accessed, if available, and/or the study registration details. | p. 4 |
| Participants | 5 | Describe the rationale for stakeholder groups involved in the COS development process, eligibility criteria for participants from each group, and a description of how the individuals involved were identified. | pp. 4-6 |
| Information Sources | 6a | Describe the information sources used to identify an initial list of outcomes. | p. 4 |
|  | 6b | Describe how outcomes were dropped/combined, with reasons (if applicable). | pp. 4-7 |
| Consensus Process | 7 | Describe how the consensus process was undertaken. | pp. 5-6 |
| Outcome Scoring | 8 | Describe how outcomes were scored and how scores were summarised. | pp. 5-7 |
| Consensus Definition | 9a | Describe the consensus definition. | p. 5 |
|  | 9b | Describe the procedure for determining how outcomes were included or excluded from consideration during the consensus process. | pp. 5-7 |
| Ethics and Consent | 10 | Provide a statement regarding the ethics and consent issues for the study. | p. 4 |
| **RESULTS** | | | |
| Protocol Deviations | 11 | Describe any changes from the protocol (if applicable), with reasons, and describe what impact these changes have on the results. | N/A |
| Participants | 12 | Present data on the number and relevant characteristics of the people involved at all stages of COS development. | p. 7;  Table 1 |
| Outcomes | 13a | List all outcomes considered at the start of the consensus process. | p. 7;  Supplement |
|  | 13b | Describe any new outcomes introduced and any outcomes dropped, with reasons, during the consensus process. | pp. 7-8 |
| COS | 14 | List the outcomes in the final COS. | p. 8;  Table 2 |
| **DISCUSSION** | | | |
| Limitations | 15 | Discuss any limitations in the COS development process. | pp. 10-11 |
| Conclusions | 16 | Provide an interpretation of the final COS in the context of other evidence, and implications for future research. | pp. 9-12 |
| **OTHER INFORMATION** | | | |
| Funding | 17 | Describe sources of funding/role of funders. | p. 11 |
| Conflicts of Interest | 18 | Describe any conflicts of interest within the study team and how these were managed. | p. 11 |

Appendix S3 Endorsing airway management societies

| Difficult Airway Society |
| --- |
| Society for Airway Management |
| Safe Airway Society |
| European Airway Management Society |
| All India Difficult Airway Association |
| International Airway Management Society |
| Training in Airway Management Latin America |
| African Airways |

We would like to thank the endorsing societies for facilitating survey dissemination.

Appendix S4 Search strategy

*Original search from Ahmad 2019:*

Ahmad I, Onwochei DN, Muldoon S, Keane O, El-Boghdadly K. Airway management research: a systematic review. *Anaesthesia.* 2019 Feb;74(2):225-236. doi: 10.1111/anae.14471.

Time period: 1 January 2006 to 31 December 2017

Databases searched: MEDLINE, Embase, Web of Science

Included studies: 1505

| 1 | Anaes*.mp. |
| --- | --- |
| 2 | Exp anesthesia/ or exp anesthesiology/ or anesth*.mp |
| 3 | Exp airway/ or airway.mp or exp airway creating device/ |
| 4 | (airway adj3 management).mp [mp=title, abstract, heading word, drug trade name, original title, device manufacturer, drug manufacturer, device trade name, keyword, floating subheading word] |
| 5 | exp intubation/ or intubate*.mp. or exp artificial ventilation/ |
| 6 | videolaryng*.mp or exp laryngoscopy/ or exp videolaryngoscope/ or exp laryngoscope/ or exp endotracheal tube/ or exp endotracheal intubation/ |
| 7 | laryngeal mask.mp or exp laryngeal mask/ |
| 8 | exp supraglottic airway device/ or supraglottic airway.mp. |
| 9 | exp fiber optics/ or fibre-optic*.mp. |
| 10 | exp fiberscope/ or fiber-optic*.mp. |
| 11 | fibreoptic*.mp. |
| 12 | fibreoptic*.mp. |
| 13 | exp oxygenation/ |
| 14 | ventilation.mp. |
| 15 | exp tracheotomy/ or cricothyroid*.mp. |
| 16 | “bag valve mask” .mp. or exp manual emergency ventilator/ |
| 17 | (airway adj3 control).mp [mp=title, abstract, heading word, drug trade name, original title, device manufacturer, drug manufacturer, device trade name, keyword, floating subheading word] |
| 18 | “airway device” .mp. |
| 19 | 1 or 2 or 3 |
| 20 | exp respiration control/ |
| 21 | 4 or 5 or 6 or 7 or 8 or 9 or 10 or 11 or 12 or 13 or 14 or 15 or 16 or 17 or 18 or 20 |
| 22 | 19 and 21 |
| 23 | limit 22 to ((embase or medline) and yr=”2006–Current”) |

*Top-up search*

Time period: 1 January 2018 to 15 November 2023

Databases searched: MEDLINE, Embase

Identified studies: MEDLINE (17,073), Embase (55,300)

Identified studies: 72,323

Following deduplication: 60,023

Included (title/abstract): 2863

| 1 | Anaes*.mp. |
| --- | --- |
| 2 | Exp anesthesia/ or exp anesthesiology/ or anesth*.mp |
| 3 | Exp airway/ or airway.mp or exp airway creating device/ |
| 4 | (airway adj3 management).mp [mp=title, abstract, heading word, drug trade name, original title, device manufacturer, drug manufacturer, device trade name, keyword, floating subheading word] |
| 5 | exp intubation/ or intubate*.mp. or exp artificial ventilation/ |
| 6 | videolaryng*.mp or exp laryngoscopy/ or exp videolaryngoscope/ or exp laryngoscope/ or exp endotracheal tube/ or exp endotracheal intubation/ |
| 7 | laryngeal mask.mp or exp laryngeal mask/ |
| 8 | exp supraglottic airway device/ or supraglottic airway.mp. |
| 9 | exp fiber optics/ or fibre-optic*.mp. |
| 10 | exp fiberscope/ or fiber-optic*.mp. |
| 11 | fibreoptic*.mp. |
| 12 | fibreoptic*.mp. |
| 13 | exp oxygenation/ |
| 14 | ventilation.mp. |
| 15 | exp tracheotomy/ or cricothyroid*.mp. |
| 16 | “bag valve mask” .mp. or exp manual emergency ventilator/ |
| 17 | (airway adj3 control).mp [mp=title, abstract, heading word, drug trade name, original title, device manufacturer, drug manufacturer, device trade name, keyword, floating subheading word] |
| 18 | “airway device” .mp. |
| 19 | 1 or 2 or 3 |
| 20 | exp respiration control/ |
| 21 | 4 or 5 or 6 or 7 or 8 or 9 or 10 or 11 or 12 or 13 or 14 or 15 or 16 or 17 or 18 or 20 |
| 22 | 19 and 21 |
| 23 | limit 22 to ((embase or medline) and yr=”2018–Current”) |

Appendix S5 Participant eligibility criteria

**Patients (NHS service users and international patients)**

- About to undergo airway management for surgery in the elective or emergency setting OR
- Patients who have undergone emergency airway management previously
- AND:
  - Age > 18 years;
  - Able to give informed consent;
  - Any ethnicity and gender;
  - Willing to participate in the study;
  - Speak English language (or other given language if international).

**Clinicians engaged in airway management**

- Anaesthetists, intensivists, emergency physicians, paramedics, operating department practitioners, anaesthetic nurses, surgeons.
- Able to give informed consent.
- No other restrictions will apply.

**Researchers engaged in airway management research**

- Researchers active in the field of airway management.
- At least five publications in a peer-reviewed journal.
- Able to give informed consent.
- No other restrictions will apply.

**Other key stakeholders**

- Representatives of funding bodies, guideline developers, journal editors, industry representatives, inventors.
- Able to give informed consent.
- No other restrictions will apply.

Appendix S6 Studies contributing data

| 1. Noor Zairul, M; Khairul Faizi, A; Norzalina, E; M.N., Zairul; A.K., Faizi; Norzalina, E. Comparison of the ease of insertion of the laryngeal tube VBMTM and laryngeal mask airway during manual in-line neck stabilization. *Medical Journal of Malaysia* 2006. 61: 157-161. |
| --- |
| 1. So, MinHye; Sobue, Kazuya; Arima, Hajime; Morishima, Tetsuro; Fukumoto, Masatoshi; Nakano, Hiroshi; Tsuda, Takako; Katsuya, Hirotada; M., So; K., Sobue; H., Arima; T., Morishima; M., Fukumoto; H., Nakano; T., Tsuda; Katsuya, Hirotada. Flexible, tapered-tip tube facilitates conventional orotracheal Intubation by novice intubators. *Journal of anesthesia* 2006. 20: 344-347. |
| 1. Bertrand, Catherine; Hemery, Francois; Carli, Pierre; Goldstein, Patrick; Espesson, Catherine; Ruttimann, Michel; Macher, Jean Michel; Raffy, Brigitte; Fuster, Patrick; Dolveck, Francois; Rozenberg, Alain; Lecarpentier, Eric; Duvaldestin, Philippe; Saissy, Jean-Marie; Boussignac, Georges; Brochard, Laurent; Grp, Boussignac Study. Constant flow insufflation of oxygen as the sole mode of ventilation during out-of-hospital cardiac arrest. *Intensive care medicine* 2006. 32: 843-851. |
| 1. Hirabayashi, Y. Airway Scope versus Macintosh laryngoscpoe: A manikin study. *Emergency Medicine Journal* 2007. 24: 357-358. |
| 1. Goodman, Evan; Ziegler, E Jane; Douglas, Allen M; E.J., Goodman; E.J., Ziegler; Douglas, Allen M. The PAXpress airway causes more pharyngeal irritation than the reusable laryngeal mask airway. *AANA journal* 2007. 75: 123-125. |
| 1. Maharaj, Ch H; Costello, Jf F; Harte, Bh H; Laffey, Jg G; C.H., Maharaj; J.F., Costello; B.H., Harte; Maharaj, Ch H; Costello, Jf F; Harte, Bh H; Laffey, Jg G. Evaluation of the Airtraq and Macintosh laryngoscopes in patients at increased risk for difficult tracheal Intubation. *Anaesthesia* 2008. 63: 182-188. |
| 1. B., Achen; O.C., Terblanche; Finucane, B T; Achen, B; Terblanche, O C; Finucane, B T. View of the larynx obtained using the Miller blade and paraglossal approach, compared to that with the Macintosh blade. *Anaesthesia and Intensive Care* 2008. 36: 717-721. |
| 1. Asai, Takashi; Enomoto, Yoshiro; Shimizu, Keiko; Shingu, Koh; Okuda, Yasuhisa; T., Asai; Y., Enomoto; K., Shimizu Shingu; K., Shimizu Shingu; Okuda, Yasuhisa. The pentax-AWS video-laryngoscope: The first experience in one hundred patients. *Anesthesia and Analgesia* 2008. 106: 257-259. |
| 1. Hirabayashi, Y; Seo, N; Y., Hirabayashi; Seo, N; Hirabayashi, Y; Seo, N. Airtraq optical laryngoscope: tracheal Intubation by novice laryngoscopists. *Emergency Medicine Journal* 2009. 26: 112-113. |
| 1. E., Marret Malin; J., De Montblanc; Y., Ynineb; E., Marret Malin; Bonnet, F; Malin, E; de Montblanc, J; Ynineb, Y; Marret, E; Bonnet, F. Performance of the Airtraq((TM)) laryngoscope after failed conventional tracheal Intubation: a case series. *Acta Anaesthesiologica Scandinavica* 2009. 53: 858-863. |
| 1. Aneeshkumar, M K; Jones, Terry M; Birchall, Martin A; M.K., Aneeshkumar; T.M., Jones; Birchall, Martin A. A new indicator-guided percutaneous emergency cricothyrotomy device: in vivo study in man. *European archives of oto-rhino-laryngology* 2009. 266: 105-109. |
| 1. C., Bourolias; A., Gkotsis; A., Kontaxakis; Tsoukarelis, Panagiotis; Bourolias, Constantinos; Gkotsis, Antonios; Kontaxakis, Anastasios; Tsoukarelis, Panagiotis. Lidocaine spray vs tetracaine solution for transnasal fiber-optic laryngoscopy. *American Journal of Otolaryngology - Head and Neck Medicine and Surgery* 2010. 31: 114-116. |
| 1. Komatsu, R; Kamata, K; Sessler, D I; Ozaki, M; R., Komatsu; K., Kamata; D.I., Sessler. A comparison of the Airway Scope (R) and McCoy laryngoscope in patients with simulated restricted neck mobility. *Anaesthesia* 2010. 65: 564-568. |
| 1. Sharma, Bimla; Sehgal, Raminder; Sahai, Chand; Sood, Jayashree. PLMA vs. I-gel: A Comparative Evaluation of Respiratory Mechanics in Laparoscopic Cholecystectomy. *Journal of anaesthesiology, clinical pharmacology* 2010. 26: 451-457. |
| 1. Butchart, Ag G; Tjen, C; Garg, A; Young, P; A.G., Butchart; C., Tjen; A., Garg; Butchart, Ag G; Tjen, C; Garg, A; Young, P. Paramedic laryngoscopy in the simulated difficult airway: comparison of the Venner A.P. Advance and GlideScope Ranger video laryngoscopes. *Academic Emergency Medicine* 2011. 18: 692-698. |
| 1. Sanuki, Takuro; Nakatani, Gosuke; Sugioka, Shingo; Daigo, Erina; Kotani, Junichiro; T., Sanuki; G., Nakatani; S., Sugioka; E., Daigo; Kotani, Junichiro. Comparison of the Ambu AuraFlex with the laryngeal mask airway Flexible: a manikin study. *Journal of Oral and Maxillofacial Surgery* 2011. 69: e269-72. |
| 1. Hosten, T; Gurkan, Y; Ozdamar, D; Tekin, M; Solak, M; Toker, K. Comparison of the Laryngeal Mask Airway (CTrach(TM)) and Direct Coupled Interface-Video Laryngoscope for Endotracheal Intubation: a Prospective, Randomized, Clinical Study. *Balkan Medical Journal* 2012. 29: 268-272. |
| 1. S.S., Wahba; T.F., Tammam. Comparative study of awake endotracheal Intubation with Glidescope video laryngoscope versus flexible fiber optic bronchoscope in patients with traumatic cervical spine injury. *Egyptian Journal of Anaesthesia* 2012. 28: 257-260. |
| 1. M.A., Tolon; O.M., Zanaty; W., Shafshak; Tolon, Ma; Zanaty, Om; Shafshak, W; Arida, Ee. Comparative study between the use of Macintosh Laryngoscope and Airtraq in patients with cervical spine immobilization. *Alexandria Journal of Medicine* 2012. 48: 179-185. |
| 1. D., Haske; B., Schempf; G., Gaier; Niederberger, C. Performance of the i-gelTM during pre-hospital cardiopulmonary resuscitation. *Resuscitation* 2013. 84: 1229-1232. |
| 1. Wasem, S; Lazarus, M; Hain, J; Festl, J; Kranke, P; Roewer, N; Lange, M; Smul, Tm M; S., Wasem; M., Lange Lazarus; J., Hain; J., Festl; P., Kranke; N., Roewer; M., Lange Lazarus; Wasem, S; Lazarus, M; Hain, J; Festl, J; Kranke, P; Roewer, N; Lange, M; Smul, Tm M. Comparison of the Airtraq and the Macintosh laryngoscope for double-lumen tube Intubation: a randomised clinical trial. *European journal of anaesthesiology* 2013. 30: 180-186. |
| 1. Saracoglu, Ayten; Dal, Didem; Pehlivan, Gokhan; Gogus, Fevzi Yilmaz. The Professional Experience of Anaesthesiologists in Proper Inflation of Laryngeal Mask and Endotracheal Tube Cuff. *Turkish journal of anaesthesiology and reanimation* 2014. 42: 234-238. |
| 1. S., Dhamotharan; N.R., Singh; S.S., Singh; Singh, M B. Comparative evaluation of fentanyl and midazolam with propofol induction on laryngeal mask airway insertion conditions: A study. *JMS - Journal of Medical Society* 2014. 28: 185-189. |
| 1. A., Peirovifar; A., Mahmoodpoor; S.E.J., Golzari; H., Soleimanpour; Y., Eslampour; Peirovifar, A; Mahmoodpoor, A; Golzari, S E; Soleimanpour, H; Eslampour, Y; Fattahi, V. Efficacy of video-guided laryngoscope in airway management skills of medical students. *Journal of Anaesthesiology Clinical Pharmacology* 2014. 30: 488-491. |
| 1. R., Bhola; S., Bhalla; R., Gupta; I., Singh; Bhola, R; Bhalla, S; Gupta, R; Singh, I; Kumar, S. Tracheal Intubation in patients with cervical spine immobilization: A comparison of McGrath video laryngoscope and Truview EVO2 laryngoscope. *Indian Journal of Anaesthesia* 2014. 58: 269-274. |
| 1. J.K., Chan; I., Ng; J.P., Ang; S.M., Koh; K., Lee; P., Mezzavia; J., Morris; F., Loh; Chan, Jk K; Ng, I; Ang, Jp P; Koh, Sm M; Lee, K; Mezzavia, P; Morris, J; Loh, F; Segal, R; J.K., Chan; I., Ng; J.P., Ang; S.M., Koh; K., Lee; P., Mezzavia; J., Morris; F., Loh. Randomised controlled trial comparing the Ambu aScopeTM2 with a conventional fibreoptic bronchoscope in orotracheal Intubation of anaesthetised adult patients. *Anaesthesia & Intensive Care* 2015. 43: 479-484. |
| 1. Hamp, T; Stumpner, T; Grubhofer, G; Ruetzler, K; Thell, R; Hager, H. Haemodynamic response at double lumen bronchial tube placement - Airtraq vs. MacIntosh laryngoscope, a randomised controlled trial. *Heart Lung & Vessels* 2015. 7: 54-63. |
| 1. A.K., Pasha; K., Farhat; Iqbal, A. Evaluating effectiveness of warming endotracheal tube in blind nasotracheal Intubation in maxillofacial surgery. *Journal of Postgraduate Medical Institute* 2015. 29: 279-283. |
| 1. Akbar, S H; Ooi, J S; S.H., Akbar. Comparison between C-Mac Video-Laryngoscope and Macintosh Direct Laryngoscope During Cervical Spine Immobilization. *Middle* *East Journal of Anesthesiology* 2015. 23: 43-50. |
| 1. Saracoglu, A; Bezen, O; Sengul, T; Ugur, E H; Sener, S; Yuzer, F. Does Video Laryngoscopy Offer Advantages over Direct Laryngoscopy during Cardiopulmonary Resuscitation? *Turk Anestezi Ve Reanimasyon Dergisi* 2015. 43: 263-268. |
| 1. Yoo, Ji Young; Kwak, Hyun Jeong; Lee, Kyung Cheon; Kim, Go Wun; Kim, Jong Yeop; J.Y., Yoo; H.J., Kwak; K.C., Lee; G.W., Kim; Kim, Jong Yeop; Yoo, Ji Young; Kwak, Hyun Jeong; Lee, Kyung Cheon; Kim, Go Wun; Kim, Jong Yeop. Predicted EC50 and EC95 of Remifentanil for Smooth Removal of a Laryngeal Mask Airway Under Propofol Anesthesia. *Yonsei medical journal* 2015. 56: 1128-1133. |
| 1. Mishra, Sandeep Kumar; Sivaraman, B; Balachander, Hemavathy; Naggappa, Mahesh; Parida, Satyen; Bhat, Ravindra R; Yuvaraj, Kotteeswaran. Effect of pneumoperitoneum and Trendelenberg position on oropharyngeal sealing pressure of I-gelTM and ProSeal LMATM in laparoscopic gynecological surgery: A randomized controlled trial. *Anesthesia, essays and researches* 2015. 9: 353-358. |
| 1. S., Chhatrapati; A., Sahu; S.S., Auti; Aswar, S G. Evaluation of the effect of tracheal tube orientation on success of Intubation through intubating laryngeal mask airway. *Journal of Clinical and Diagnostic Research* 2016. 10: U06-U09. |
| 1. Deguchi, S; Komasawa, N; Kido, H; Ueno, T; Minami, T; S., Deguchi; N., Komasawa; H., Kido; T., Ueno; Minami T. AO - Komasawa, Nobuyasu. Impact of pillow height on double-lumen endotracheal tube Intubation with McGRATH MAC: A prospective randomized clinical trial. *Journal of Clinical Anesthesia* 2016. 34: 339-343. |
| 1. Priyanka, A S; Nag, K; Hemanth Kumar, V R; Singh, D R; Kumar, S; Sivashanmugam, T. Comparison of King Vision and Truview Laryngoscope for Postextubation Visualization of Vocal Cord Mobility in Patients Undergoing Thyroid and Major Neck Surgeries: A Randomized Clinical Trial. *Anesth Essays Res* 2017. 11: 238-242. |
| 1. Wu, Caineng; Wei, Jianqi; Cen, Qingyun; Sha, Xuefan; Cai, Qingxiang; Ma, Wuhua; Cao, Ying. Supraglottic jet oxygenation and ventilation-assisted fibre-optic bronchoscope Intubation in patients with difficult airways. *Internal and emergency medicine* 2017. 12: 667-673. |
| 1. S., Prakash; P., Mullick; S., Bhandari; A., Kumar; A.R., Gogia; Singh, Rajvir; Prakash, Smita; Mullick, Parul; Bhandari, Shyam; Kumar, Amitabh; Gogia, Anoop Raj; Singh, Rajvir. Sternomental distance and sternomental displacement as predictors of difficult laryngoscopy and Intubation in adult patients. *Saudi Journal of Anaesthesia* 2017. 11: 273-278. |
| 1. Omur, Dilek; Bayram, Basak; Ozbilgin, Sule; Hanci, Volkan; Kuvaki, Bahar. Comparison of different stylets used for Intubation with the C-MAC D-Bladeg Videolaryngoscope: a randomized controlled study. *Revista brasileira de anestesiologia* 2017. 67: 450-456. |
| 1. Baek, M. S.; Han, M. J.; Huh, J. W.; Lim, C. M.; Koh, Y.; Hong, S. B. Video laryngoscopy versus direct laryngoscopy for first-attempt tracheal intubation in the general ward. *Annals of Intensive Care* 2018. 8(1). |
| 1. Inangil, G.; Deniz, S.; Temircan, S.; Bakal, O.; Sen, H.; Ozkan, S. Validation of tracheal intubation of wire-reinforced endotracheal tube with ultrasonography. *Signa Vitae* 2018. 14(2): 20-23. |
| 1. Mallhi, A. I.; Abbas, N.; Naqvi, S. M. N.; Murtaza, G.; Rafique, M.; Alam, S. S. A comparison of Mallampati classification, thyromental distance and a combination of both to predict difficult intubation. *Anaesthesia, Pain and Intensive Care* 2018. 22(4): 468-473. |
| 1. Siamdoust, S. S.; Rokhtabnak, F.; Motlagh, S. D.; Rahimzadeh, P.; Hassani, V.; Farnaghizad, M. Comparison of the success rate of intubation between the LMA fastrach and AirQ-ILA methods in patients undergoing elective surgery during general anaesthesia. *Anesthesiology and Pain Medicine* 2018. 8(4). |
| 1. Abraham, S.; Himarani, J.; Mary Nancy, S.; Shanmugasundaram, S.; Krishnakumar Raja, V. B. Ultrasound as an Assessment Method in Predicting Difficult Intubation: A Prospective Clinical Study. *Journal of Maxillofacial and Oral Surgery* 2018. 17: 563-569. |
| 1. Cheong, G. P. C.; Kannan, A.; Koh, K. F.; Venkatesan, K.; Seet, E. Prevailing practices in airway management: a prospective single-centre observational study of endotracheal intubation. *Singapore Med J* 2018. 59: 144-149. |
| 1. Lee, Y. C.; Lee, J.; Son, J. D.; Lee, J. Y.; Kim, H. C. Stylet angulation of 70 degrees reduces the time to intubation with the GlideScope R: A prospective randomised trial. *J Int Med Res* 2018. 46: 1428-1438. |
| 1. Ponnusamy, T.; Kundra, P.; Rudingwa, P.; Gopalakrishnan, S. Comparison of laryngeal morbidities with modified reinforced silicone tube intubation guided over a bougie vs. a guidewire: novel assessment with voice analysis. *Anaesthesia* 2018. 73: 730-737. |
| 1. Roh, G. U.; Chae, Y. J.; Lee, Y. B.; Wang, W.; Choi, C. I.; Yi, I. K. Discrimination ability of the endotracheal tube location using real-time palpation during intubation using an endotracheal tube with a preloaded stylet. *Ther Clin Risk Manag* 2018. 14: 1261-1266. |
| 1. Niyogi, S.; Biswas, A.; Chakraborty, I.; Chakraborty, S.; Acharjee, A. Attenuation of haemodynamic responses to laryngoscopy and endotracheal intubation with dexmedetomidine: A comparison between intravenous and intranasal route. *Indian Journal of Anaesthesia* 2019. 63(11): 915-923. |
| 1. Bai, Y.; Xu, Z.; Chandrashekar, M.; St Jacques, P. J.; Liang, Y.; Jiang, Y.; Kla, K. Comparison of a simplified nasal continuous positive airways pressure device with nasal cannula in obese patients undergoing colonoscopy during deep sedation: A randomised clinical trial. *Eur J Anaesthesiol* 2019. 36: 633-640. |
| 1. Birenbaum, A.; Hajage, D.; Roche, S.; Ntouba, A.; Eurin, M.; Cuvillon, P.; Rohn, A.; Compere, V.; Benhamou, D.; Biais, M.; Menut, R.; Benachi, S.; Lenfant, F.; Riou, B. Effect of Cricoid Pressure Compared With a Sham Procedure in the Rapid Sequence Induction of Anesthesia: The IRIS Randomized Clinical Trial. *JAMA Surg* 2019. 154: 42979. |
| 1. Tan, H. S.; Li, S. Y.; Yao, W. Y.; Yuan, Y. J.; Sultana, R.; Han, N. R.; Sia, A. T. H.; Sng, B. L. Association of Mallampati scoring on airway outcomes in women undergoing general anesthesia with Supreme TM laryngeal mask airway in cesarean section. *BMC Anesthesiol* 2019. 19: 122. |
| 1. Tanwar, G.; Singh, U.; Kundra, S.; Chaudhary, A. K.; Kaytal, S.; Grewal, A. Evaluation of airway care score as a criterion for extubation in patients admitted in neurosurgery intensive care unit. *J Anaesthesiol Clin Pharmacol* 2019. 35: 85-91. |
| 1. Zhou, W.; Zhang, D.; Tian, S.; Yang, Y.; Xing, Z.; Ma, R.; Zhou, T.; Bao, T.; Sun, J.; Zhang, Z. Optimal dose of pretreated-dexmedetomidine in fentanyl-induced cough suppression: a prospective randomized controlled trial. *BMC Anesthesiol* 2019. 19: 89. |
| 1. Groombridge, C.; Maini, A.; Olaussen, A.; Kim, Y.; Fitzgerald, M.; Mitra, B.; Smit, D. V. Impact of a targeted bundle of audit with tailored education and an intubation checklist to improve airway management in the emergency department: An integrated time series analysis. *Emergency Medicine Journal* 2020. 37(9): 576-580. |
| 1. Chen, W.; Chen, J.; Wang, H.; Chen, Y. Application of bedside real-time tracheal ultrasonography for confirmation of emergency endotracheal intubation in patients in the intensive care unit. *J Int Med Res* 2020. 48: 3.0006. |
| 1. Dutta, K.; Sriganesh, K.; Chakrabarti, D.; Pruthi, N.; Reddy, M. Cervical Spine Movement During Awake Orotracheal Intubation With Fiberoptic Scope and McGrath Videolaryngoscope in Patients Undergoing Surgery for Cervical Spine Instability: A Randomized Control Trial. *J Neurosurg Anesthesiol* 2020. 32: 249-255. |
| 1. Jung, W.; Kim, J. Factors associated with first-pass success of emergency endotracheal intubation. *Am J Emerg Med* 2020. 38: 109-113. |
| 1. Lakhe, G.; Poudel, H.; Adhikari, K. M. Assessment of Airway Parameters for Predicting Difficult Laryngoscopy and Intubation in a Tertiary Center in Western Nepal. *Journal of Nepal Health Research Council* 2020. 17: 516-520. |
| 1. Lee, J. Y.; Hur, H. J.; Park, H. Y.; Jung, W. S.; Kim, J.; Kwak, H. J. Comparison between video-lighted stylet (Intular Scope TM) and direct laryngoscope for endotracheal intubation in patients with normal airway. *J Int Med Res* 2020. 48: 3.0006. |
| 1. Louro, J.; Dudaryk, R.; Rodriguez, Y.; Dutton, R. P.; Epstein, R. H. Airway management at Level 1 trauma center in the era of video laryngoscopy. *Int J Crit Illn Inj Sci* 2020. 10: 20-24. |
| 1. Lumb, A. B.; Savic, L.; Horsford, M. R.; Hodgson, S. R. Effects of tracheal intubation and tracheal tube position on regional lung ventilation: an observational study. *Anaesthesia* 2020. 75: 359-365. |
| 1. Park, S.; Kim, G. S.; Choi, D. H.; Ko, J. S.; Park, J. B.; Son, Y. H.; Han, S.; Park, J. Comparison of Pulmonary Gas Exchange During Kidney Transplantation: Second-Generation Laryngeal Mask Airway vs Endotracheal Tube. *Transplant Proc* 2020. 52: 1695-1699. |
| 1. Rawal, P.; Shrestha, S. M. The Evaluation of Thyromental Height Test as a Single, Accurate Predictor of Difficult Laryngoscopy. *Journal of Nepal Health Research Council* 2020. 18: 271-276. |
| 1. Won, D.; Chang, J. E.; Kim, H.; Lee, J. M.; Oh, Y.; Hwang, J. Y. Effect of intraoperative neuromuscular blockade on postoperative sore throat and hoarseness in patients undergoing spinal surgery: a prospective observational study. *Nature Scientific Reports* 2020. 10: 14810. |
| 1. Mazzeffi, M. A.; Petrick, K. M.; Magder, L.; Greenwald, B. D.; Darwin, P.; Goldberg, E. M.; Bigeleisen, P.; Chow, J. H.; Anders, M.; Boyd, C. M.; Kaplowitz, J. S.; Sun, K.; Terrin, M.; Rock, P. High-Flow Nasal Cannula Oxygen in Patients Having Anesthesia for Advanced Esophagogastroduodenoscopy: HIFLOW-ENDO, a Randomized Clinical Trial. *Anesthesia and Analgesia* 2021. 132(3): 743-751. |
| 1. Sepmiko, J.; Senapathi, T. G. A.; Wiryana, M.; Kurniyanta, I. P.; Widnyana, I. M. G.; Sutawan, I. B. K. J. The efficacy of O-Mac, patent video laryngoscope, and conventional laryngoscope for intubation in the operating room. *Open Access Macedonian Journal of Medical Sciences* 2021. 9(B): 646-650. |
| 1. Singhal, S.; Kaur, K.; Yadav, P. A study to evaluate the role of experience in acquisition of the skill of orotracheal intubation in adults. *Journal of Anaesthesiology Clinical Pharmacology* 2021. 37(3): 469-474. |
| 1. Tas, G.; Algin, A.; Ozdemir, S.; Erdogan, M. O. Prospective observational study of the endotracheal intubation complications in Emergency Department. *Journal of Experimental and Clinical Medicine (Turkey)* 2021. 38(4): 678-681. |
| 1. Arnold, I.; Alkhouri, H.; Badge, H.; Fogg, T.; McCarthy, S.; Vassiliadis, J. Current airway management practices after a failed intubation attempt in Australian and New Zealand emergency departments. *Emerg Med Australas* 2021. 33: 808-816. |
| 1. Chandy, J.; Pillai, R.; Mathew, A.; Philip, A. V.; George, S. P.; Sahajanandan, R. A randomized clinical trial comparing the King Vision (channeled blade) and the CMAC (D blade) videolaryngoscopes in patients with cervical spine immobilization. *J Anaesthesiol Clin Pharmacol* 2021. 37: 604-609. |
| 1. Cheng, T.; Wang, L. K.; Wu, H. Y.; Yang, X. D.; Zhang, X.; Jiao, L. Shikani Optical Stylet for Awake Nasal Intubation in Patients Undergoing Head and Neck Surgery. *Laryngoscope* 2021. 131: 319-325. |
| 1. Houghton Budd, S.; Alexander-Elborough, E.; Brandon, R.; Fudge, C.; Hardy, S.; Hopkins, L.; Paul, B.; Philips, S.; Thatcher, S.; Winsor, P. Drug-free tracheal intubation by specialist paramedics (critical care) in a United Kingdom ambulance service: a service evaluation. *BMC Emerg Med* 2021. 21: 144. |
| 1. Hsu, P. K.; Lee, Y. Y.; Chuang, L. C.; Ting, C. K.; Tsou, M. Y. Nonintubated versus intubated "one-stage" preoperative localization and thoracoscopic lung resection. *JTCVS Tech* 2021. 10: 517-525. |
| 1. Jaber, S.; Rolle, A.; Godet, T.; Terzi, N.; Riu, B.; Asfar, P.; Bourenne, J.; Ramin, S.; Lemiale, V.; Quenot, J. P.; Guitton, C.; Prudhomme, E.; Quemeneur, C.; Blondonnet, R.; Biais, M.; Muller, L.; Ouattara, A.; Ferrandiere, M.; Saint-Leger, P.; Rimmele, T.; Pottecher, J.; Chanques, G.; Belafia, F.; Chauveton, C.; Huguet, H.; Asehnoune, K.; Futier, E.; Azoulay, E.; Molinari, N.; De Jong, A. Effect of the use of an endotracheal tube and stylet versus an endotracheal tube alone on first-attempt intubation success: a multicentre, randomised clinical trial in 999 patients. *Intensive Care Med* 2021. 47: 653-664. |
| 1. Kim, D.; Park, S.; Kim, J. M.; Choi, G. S.; Kim, G. S. Second generation laryngeal mask airway during laparoscopic living liver donor hepatectomy: a randomized controlled trial. *Nature Scientific Reports* 2021. 11: 3532. |
| 1. Kim, H. J.; Roh, Y.; Yun, S. Y.; Park, W. K.; Kim, H. Y.; Lee, M. H. Comparison of the selection of nasotracheal tube diameter based on the patient's sex or size of the nasal airway: A prospective observational study. *PLoS ONE* 2021. 16: e0248296. |
| 1. Meshram, T. M.; Ramachandran, R.; Trikha, A.; Rewari, V. Haemodynamic responses following orotracheal intubation in patients with hypertension---Macintosh direct laryngoscope versus Glidescope Rvideolaryngoscope. *Indian Journal of Anaesthesia* 2021. 65: 321-327. |
| 1. Murphy, D. L.; Bulger, N. E.; Harrington, B. M.; Skerchak, J. A.; Counts, C. R.; Latimer, A. J.; Yang, B. Y.; Maynard, C.; Rea, T. D.; Sayre, M. R. Fewer tracheal intubation attempts are associated with improved neurologically intact survival following out-of-hospital cardiac arrest. *Resuscitation* 2021. 167: 289-296. |
| 1. Wahdan, A. S.; El-Refai, N. A. R.; Omar, S. H.; Abdel Moneem, S. A.; Mohamed, M. M.; Hussien, M. M. Endotracheal intubation in patients undergoing open abdominal surgery in the lateral position: a comparison between the intubating video stylet and fiberoptic intubating bronchoscopy. *Korean Journal Anesthesiol* 2021. 74: 234-241. |
| 1. Xu, Z.; Yu, H.; Luo, Y.; Ye, Y.; Zhou, C.; Liang, P. A randomized trial to assess the effect of cricoid displacing maneuver on the success rate of blind placement of double-lumen tube and Univent bronchial blocker. *Annals of Palliative Medicine* 2021. 10: 1976-1984. |
| 1. Zengin, M.; Akdagli Ekici, A.; Dogan, G.; Atan, D.; Alagoz, A. The Effect of Different Types of Laryngeal Mask Airways on Sound Quality: A Prospective Randomized Study. *Cureus* 2021. 13: e19056. |
| 1. Zhang, J.; Drakeford, P. A.; Ng, V.; Seng, Z.; Chua, M.; Tan, N.; Mathew, D.; Teoh, W. H. Ventilatory performance of AMBU R AuraGain TM and LMA R Supreme TM in laparoscopic surgery: A randomised controlled trial. *Anaesth Intensive Care* 2021. 49: 395-403. |
| 1. Zheng, J.; Liang, H.; Wang, R.; Zhong, R.; Jiang, S.; Wang, W.; Zhao, Y.; Chen, Z.; Liang, W.; Liu, J.; He, J. Perioperative and long-term outcomes of spontaneous ventilation video-assisted thoracoscopic surgery for non-small cell lung cancer *Translational Lung Cancer Research* 2021. 10: 3875-3887. |
| 1. Fehlmann, C. A.; Chan, M.; Betend, R.; Novotny-Court, F.; Suppan, M.; Savoldelli, G. L.; Suppan, L. Impact of Operator Medical Specialty on Endotracheal Intubation Rates in Prehospital Emergency Medicine-A Retrospective Cohort Study. *Journal of Clinical Medicine* 2022. 11(7). |
| 1. Gulati, S.; Samui, S.; De, A. Comparison of Macintosh, McCoy, Truview EVO2, and King Vision Laryngoscopes for Intubation in Patients with Immobilized Cervical Spine: A Randomized, Controlled Trial. *Bali Journal of Anesthesiology* 2022. 6(2): 108-114. |
| 1. Kandi, S.; KantaPanigrahy, L.; Mishra, J.; Patel, P. K.; Jena, P.; Dash, A. Comparison between Polyvinyl Chloride and Flexometallic Endotracheal Tube for Blind Tracheal Intubation through I-gel: A Randomised Clinical Study. *Journal of Clinical and Diagnostic Research* 2022. 16(7): UC50-UC54. |
| 1. Kumar, A.; Kumar, R.; Avinash, R. Comparison of Efficacy between Baska Mask and I-GEL (Supraglottic Airway Devices) in Patients Undergoing Elective Surgery. *International Journal of Pharmaceutical and Clinical Research* 2022. 14(10): 411-418. |
| 1. Hussain, D.; Kundal, R.; Kumar, A.; Sabharwal, N. An Analysis of the Comparative Efficacy Between a Third-Generation and a Second-Generation Supraglottic Airway Device in Patients Undergoing Laparoscopic Cholecystectomy. *Cureus* 2022. 14: e22592. |
| 1. Jiang, J.; Wang, Z.; Xu, Q.; Chen, Q.; Lu, W. Development of a nomogram for prediction of postoperative sore throat in patients under general anaesthesia: a single-centre, prospective, observational study. *BMJ Open* 2022. 12: e059084. |
| 1. Maughan, E. F.; Rotman, A.; Rouhani, M. J.; Thong, G.; Poncia, J.; Myatt, J.; Al Yaghchi, C.; Sandhu, G. Suspension laryngoscopy experiences in a tertiary airway service: A prospective study of 150 procedures. *Clin Otolaryngol* 2022. 47: 52-60. |
| 1. Mohseni, M.; Farahmand Rad, R.; Jafarian, A. A.; Zarisfi, A. H.; Masoudi, N. The Effect of Softening of Endotracheal Tubes on the Decrement of Postoperative Hoarseness and Sore Throat. *Anesthesiology and Pain Medicine* 2022. 12: e123910. |
| 1. Tan, N. E.; Yoong, K. P. Y.; Yahya, H. M. F. Use of HEAVEN criteria for predicting difficult intubation in the emergency department. *Clinical and Experimental Emergency Medicine* 2022. 9: 29-35. |
| 1. De, A.; Dwivedi, D.; Verma, R. N.; Nayani, R.; Mateen, M. A.; Bhatia, J. S. A comparative evaluation of LMA GastroTM versus Gastro Laryngeal Tube for airway management in patients undergoing endoscopic retrograde cholangiopancreatography under general anesthesia: A randomized control study. *Trends in Anaesthesia and Critical Care* 2023. 52. |
| 1. Nagraj, S.; Gandhi, P. S.; Saxena, D.; Saha, A.; Sharma, R. Comparison between Three Insertion Techniques for Supraglottic Airway Device I-Gel Placement: Standard, Rotation and Reverse in Terms of Insertion Characteristics & Success Rate. *International Journal of Toxicological and Pharmacological Research* 2023. 13(4): 250-256. |
| 1. Rajashree, S. M.; Singh, D.; Raksha, M. H.; Ashwini, A. Difficult laryngoscopy based on Cormac Lehane grading: Determinants. *Journal of Cardiovascular Disease Research* 2023. 14(3): 1192-1195. |
| 1. Sjoblom, A.; Hedberg, M.; Johansson, S.; Henningsson, R.; Soumpasis, I.; Lafrenz, H.; Tornberg, D.; Lodenius, A.; Fagerlund, M. J. Pre-oxygenation using high-flow nasal oxygen in parturients undergoing caesarean section in general anaesthesia: A prospective, multi-centre, pilot study. *Acta Anaesthesiologica Scandinavica* 2023. 67(8): 1028-1036. |
| 1. Soenarto, R. F.; Nugroho, A. M.; Ramlan, A. A. W.; Pratama, B. Head elevation during preoxygenation can delay desaturation time: A randomized-controlled trial. *Trends in Anaesthesia and Critical Care* 2023. 50. |
| 1. Sony, S.; Krishnamurthy, J.; Reddy, K. N.; Motiani, P.; Shekhar, S. Comparison of Normal Saline and Alkalinized 2% Lignocaine to Reduce Emergence Phenomenon and Post-Intubation Morbidities: A Prospective, Double-Blind, Randomized Study. *Cureus* 2023. 15: e33910. |
| 1. Win, M.; Erkalp, K.; Demirgan, S.; Ozcan, F. G.; Sevdi, M. S.; Selcan, A. Comparison of the patients with diabetes mellitus using either insulin or oral antidiabetic drug in terms of difficult laryngoscopy: A randomized controlled study Niger J Clin Pract 2023. 26: 1423-1429. |
| 1. Yan, C. L.; Zhang, Y. Q.; Chen, Y.; Qv, Z. Y.; Zuo, M. Z. To compare the influence of blind insertion and up-down optimized glottic exposure manoeuvre on oropharyngeal leak pressure using SaCoVLM TM video laryngeal mask among patients undergoing general anesthesia. *J Clin Monit Comput* 2023. 37: 593-598. |

Appendix S7 Proposed research agenda

| 1 | Difficult airway: measurement instruments for difficult laryngoscopy |
| --- | --- |
| 2 | Difficult airway: thresholds for defining difficult laryngoscopy |
| 3 | First attempt success without complications: is a maximum time cut-off required when absence of complications is built into the outcome? |
| 4 | Reporting serious adverse events as a core outcome: necessary or redundant? |
| 5 | Cardiac arrest: which events can be attributed to airway management? |
| 6 | Cardiac arrest: what is the optimal timepoint for assessing cardiac arrest as an outcome in airway management studies? |
| 7 | Pulmonary complications as a composite outcome: which complications are relevant and important to patients and clinicians for airway management research? |
| 8 | Patient-reported outcome measures in airway management research |
