# Supplementary material for: A core outcome set for airway management research
Source: Anaesthesia. 2025 Nov 7;81(3):373–82. doi: 10.1111/anae.70026 (PMC12893836; doi:10.1111/anae.70026)
Supplement: Supplementary file 3 — Figure S1. Modified PRISMA flow diagram. Figure S2. Individual outcome voting results grouped by stakeholder group (round 1). Figure S3. Individual outcome voting results grouped by stakeholder group (round 2). Figure S4. Summary of votes according to stakeholder group in rounds 1 and 2. [file ANAE-81-373-s002.docx]

Figure S1 Modified PRISMA flow diagram

Generated using *PRISMA2020* Shiny app [1].

**Reference**

1. Haddaway NR, Page MJ, Pritchard CC, McGuinness LA. Prisma2020: An r package and shiny app for producing Prisma 2020-compliant flow diagrams, with interactivity for optimised digital transparency and open synthesis. *Campbell Systematic Reviews* 2022; **18:** e1230. 10.1002/cl2.1230

Figure S2 Individual outcome voting results grouped by stakeholder group (Round 1)


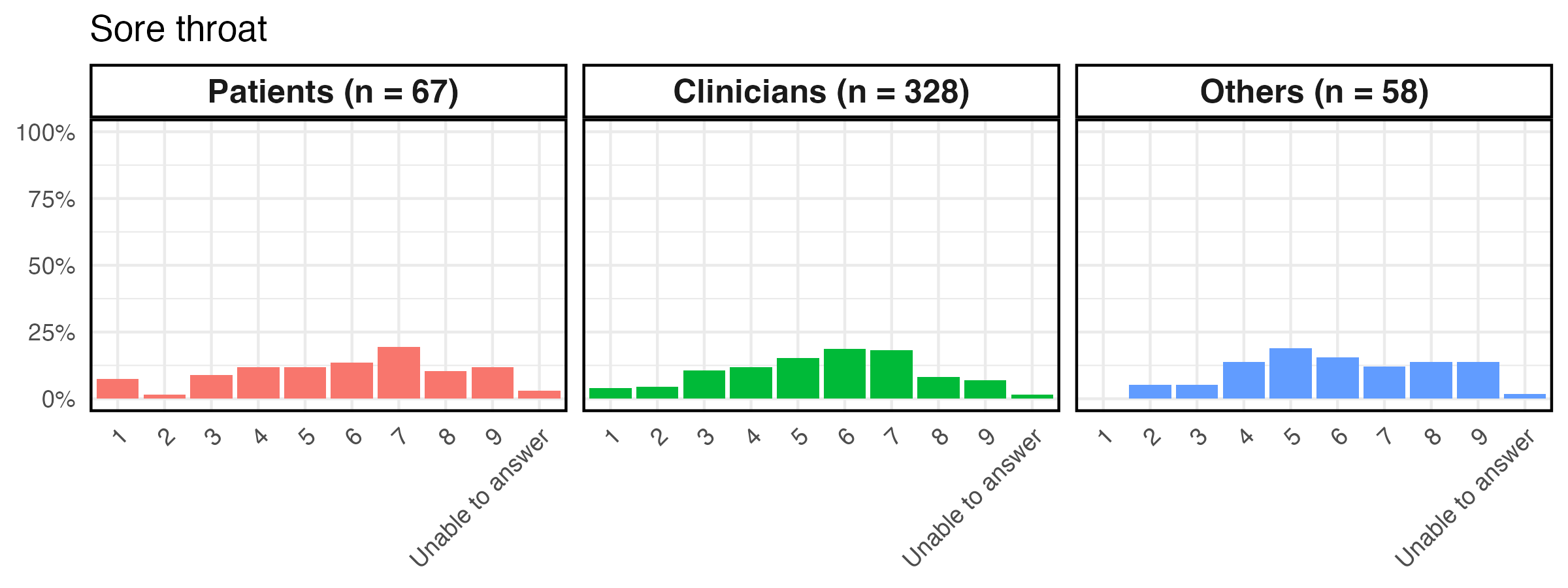

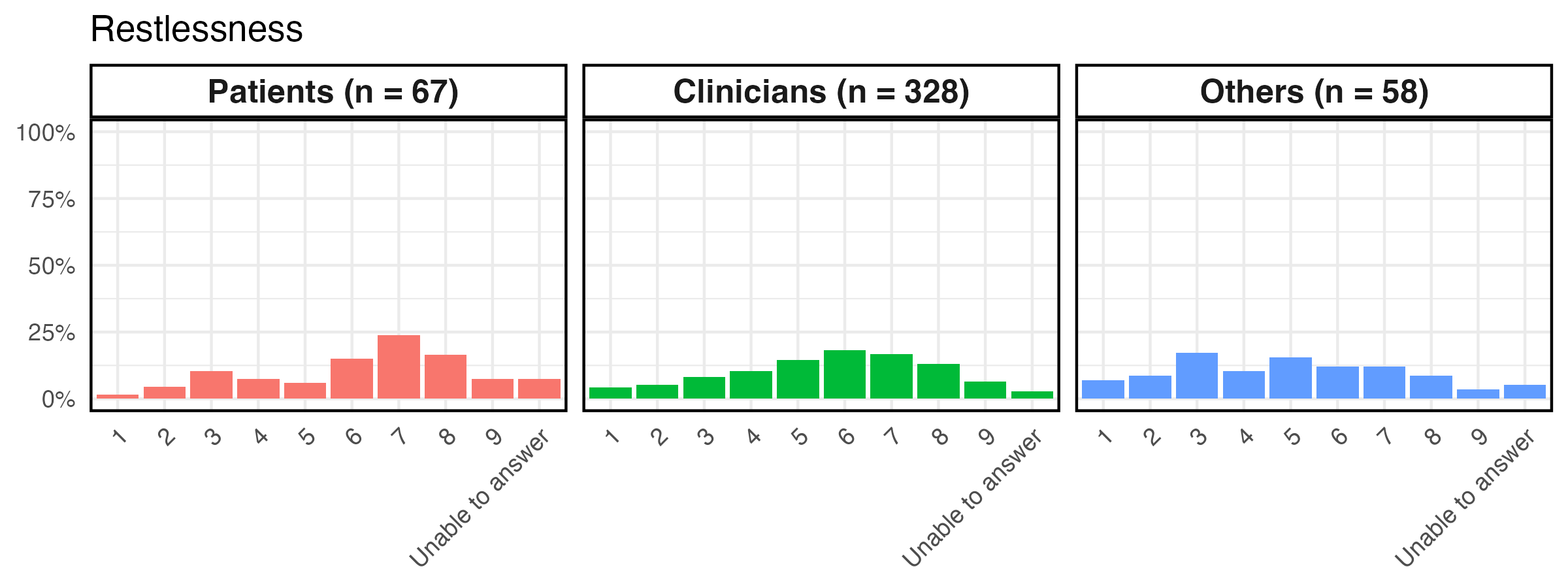


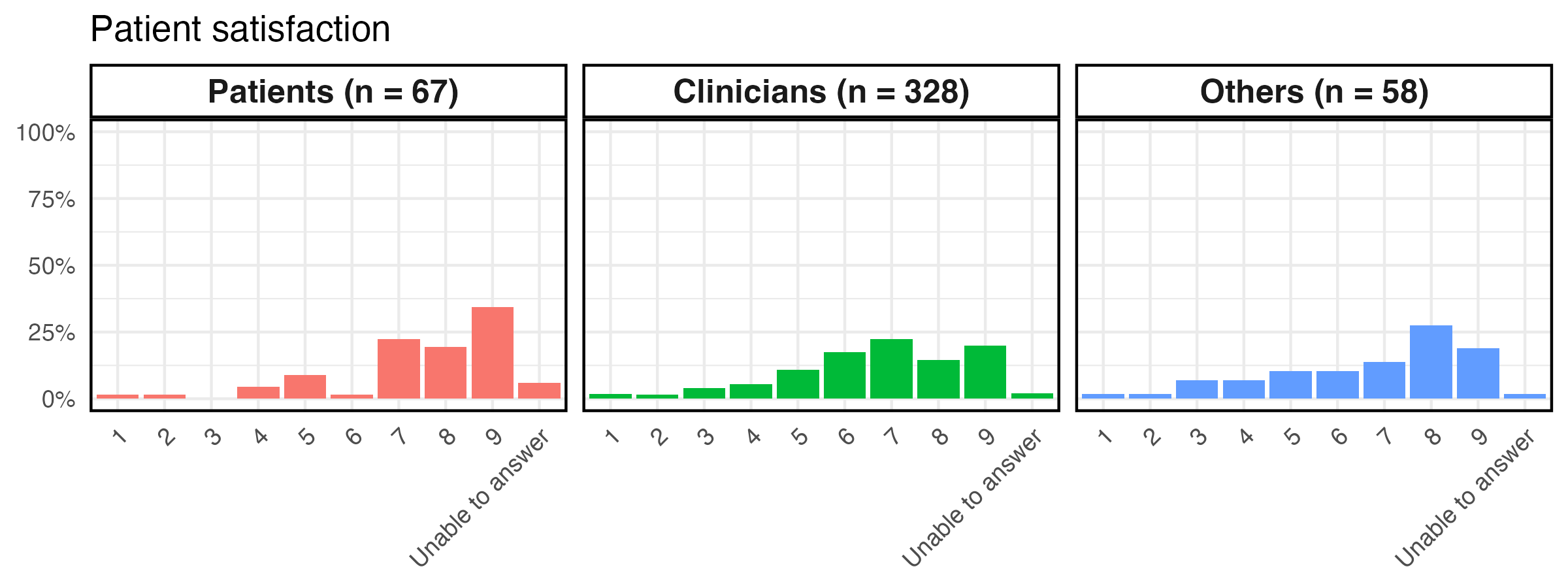

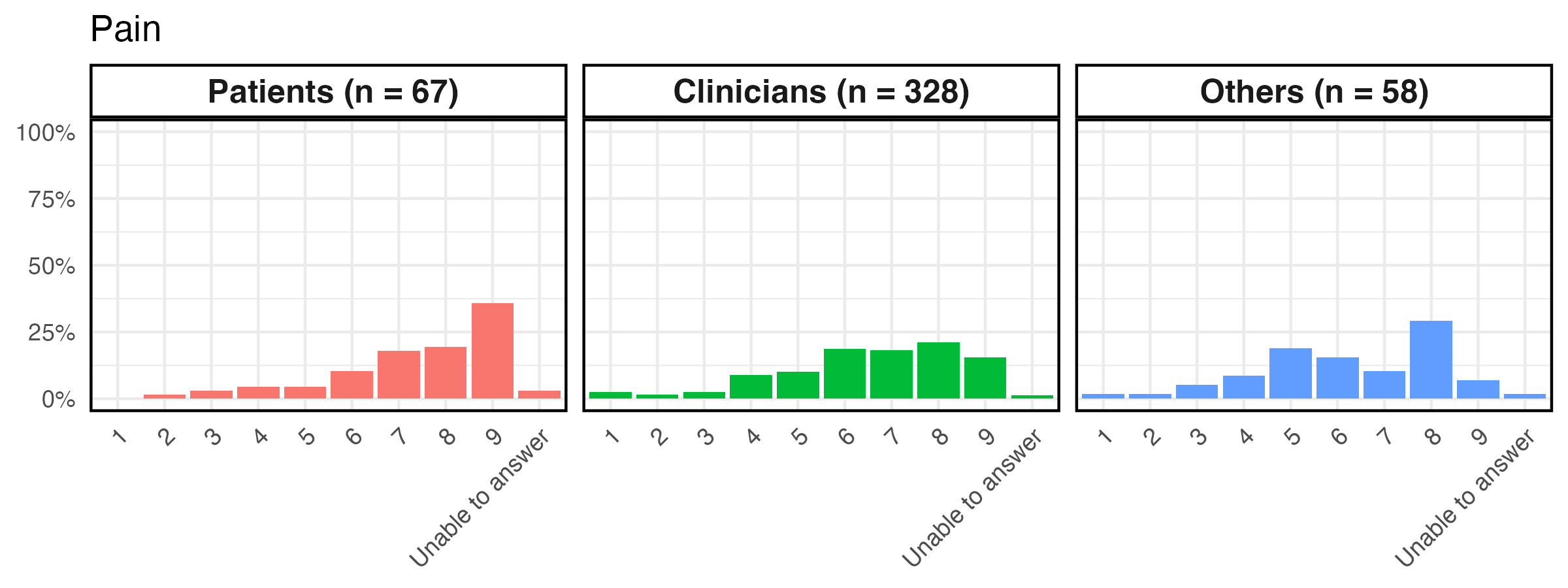

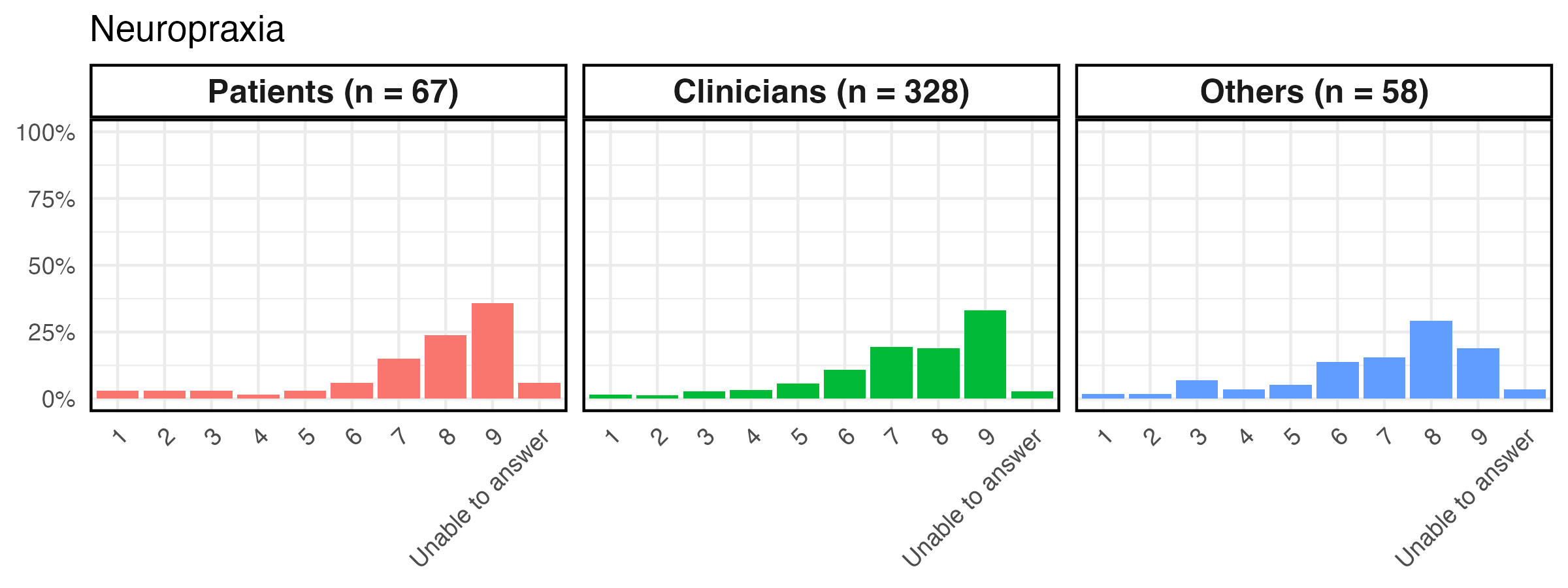

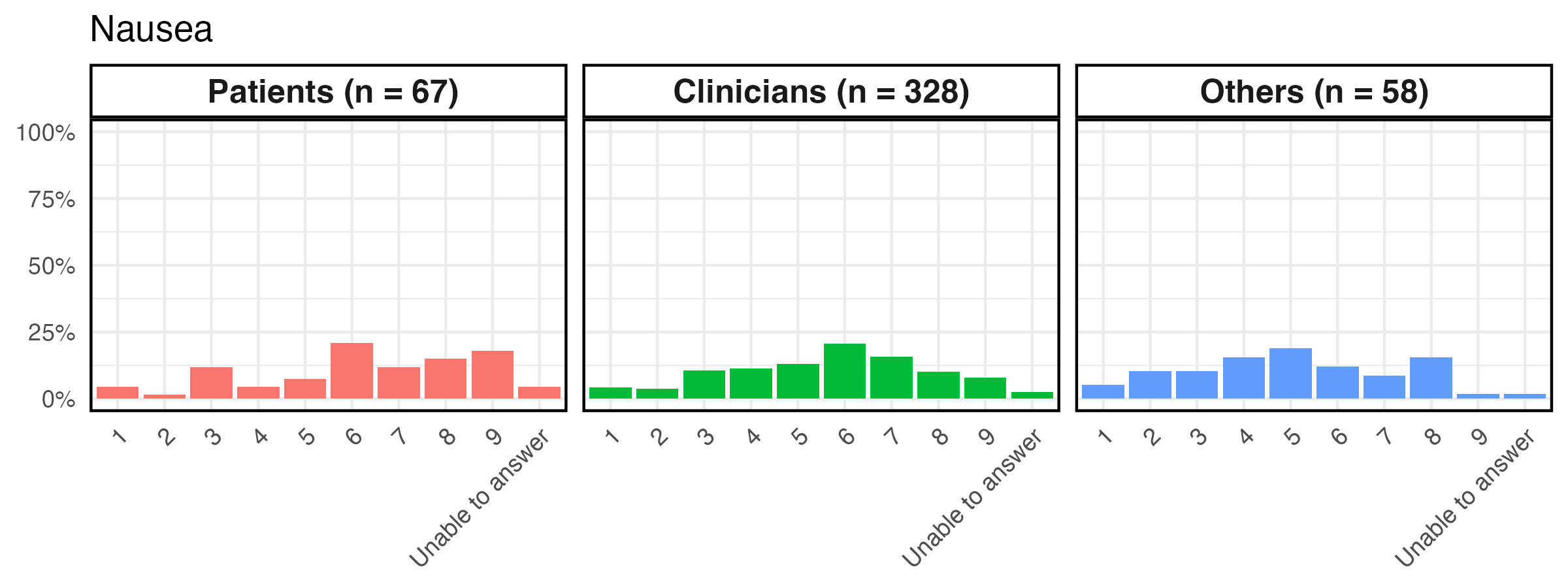

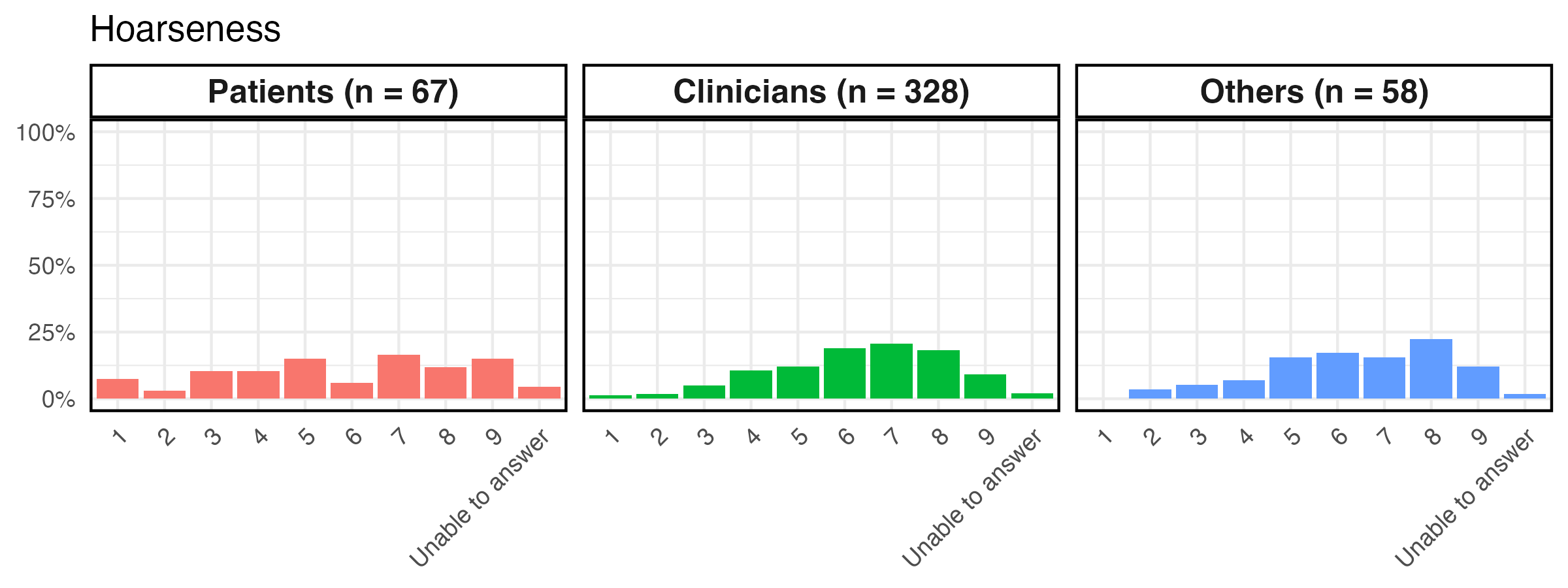

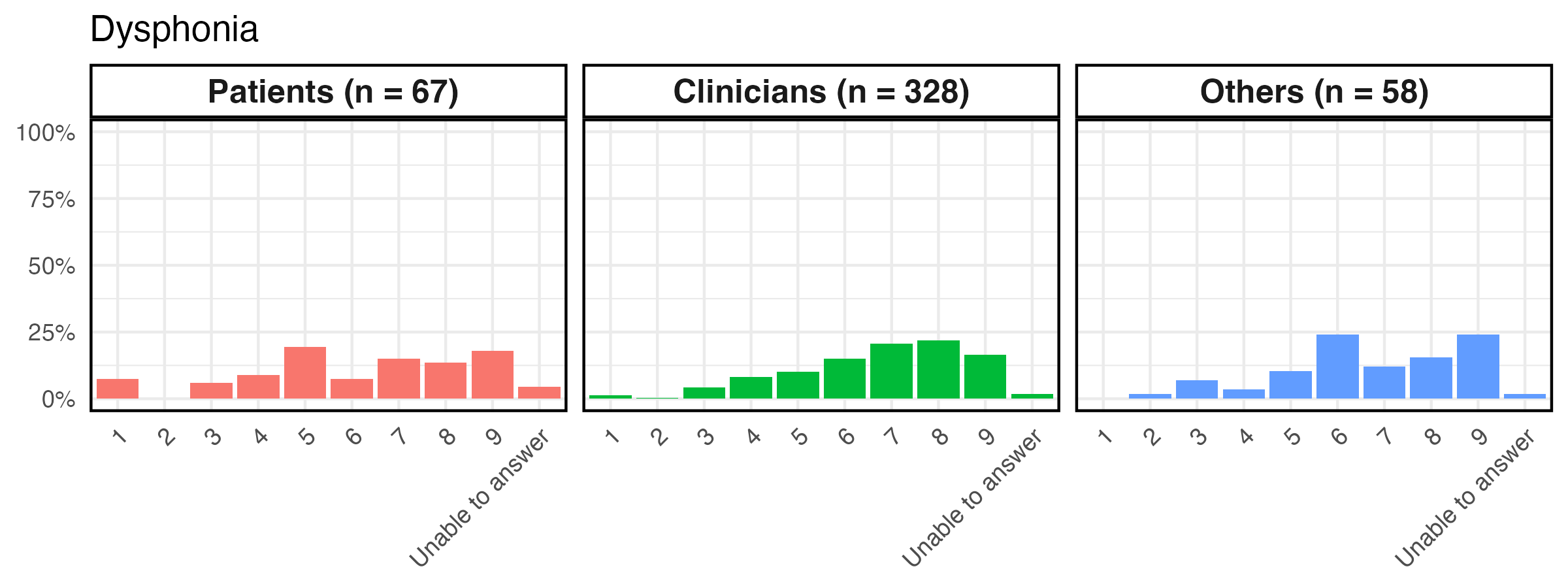

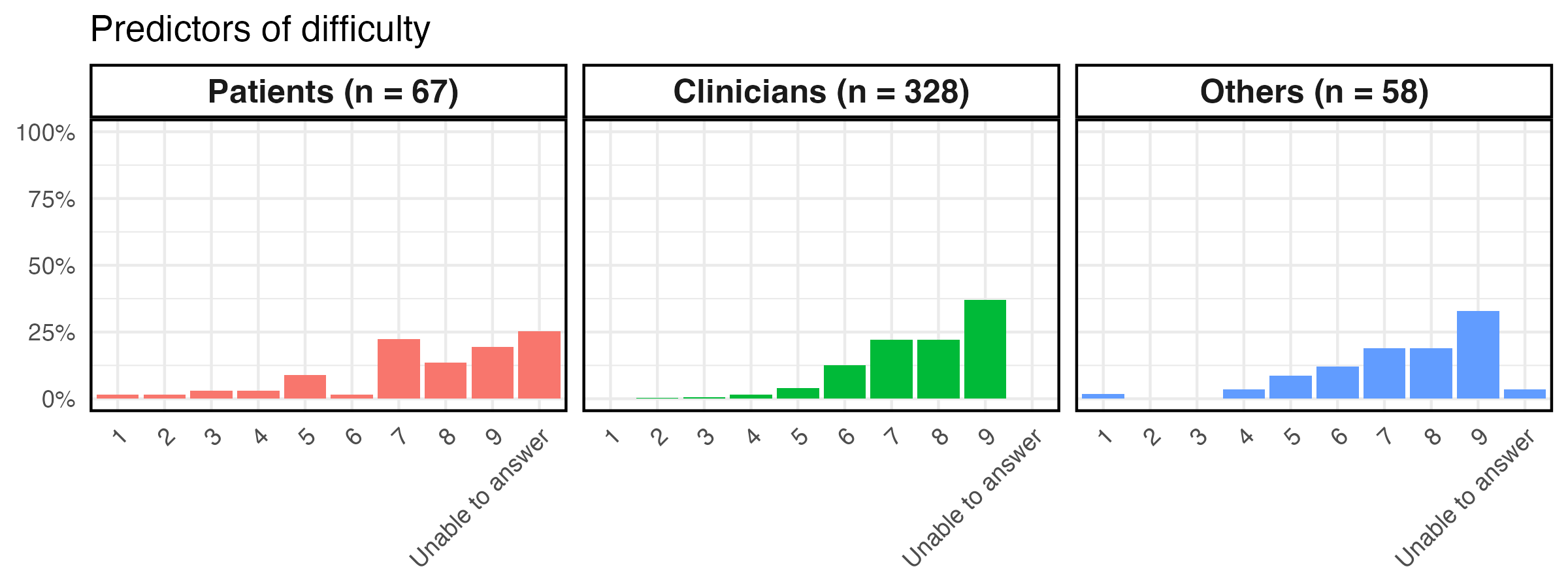

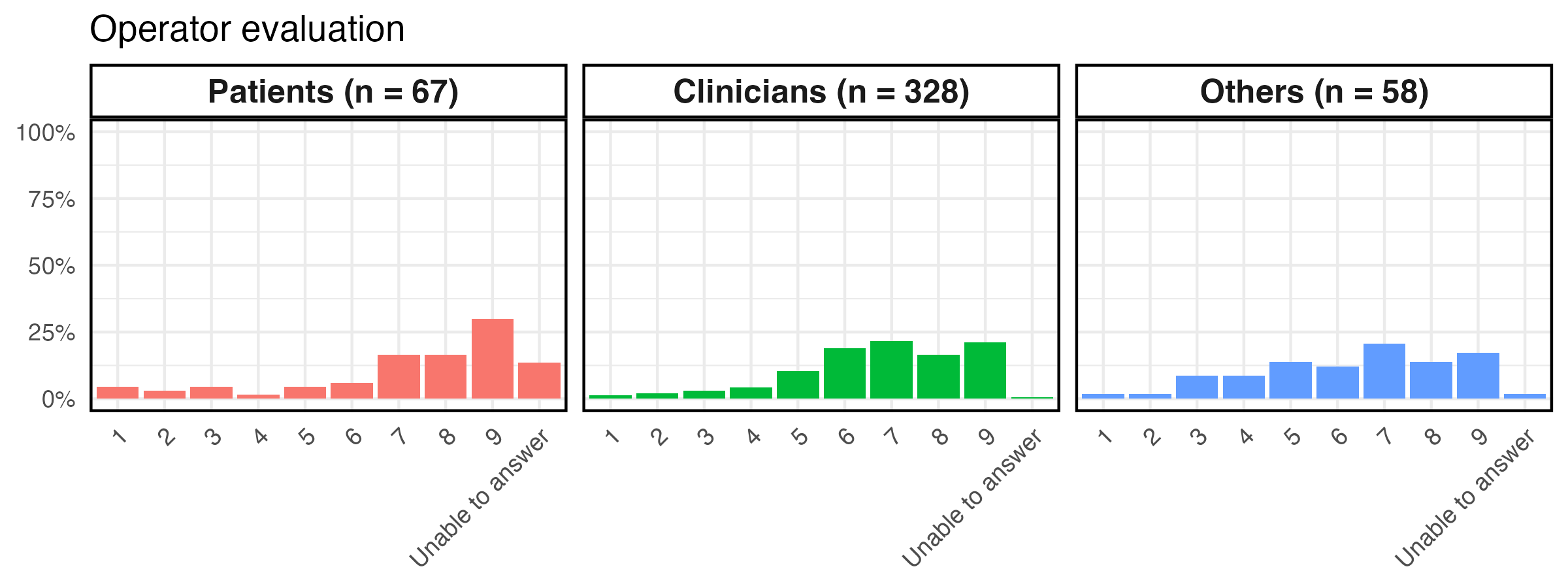

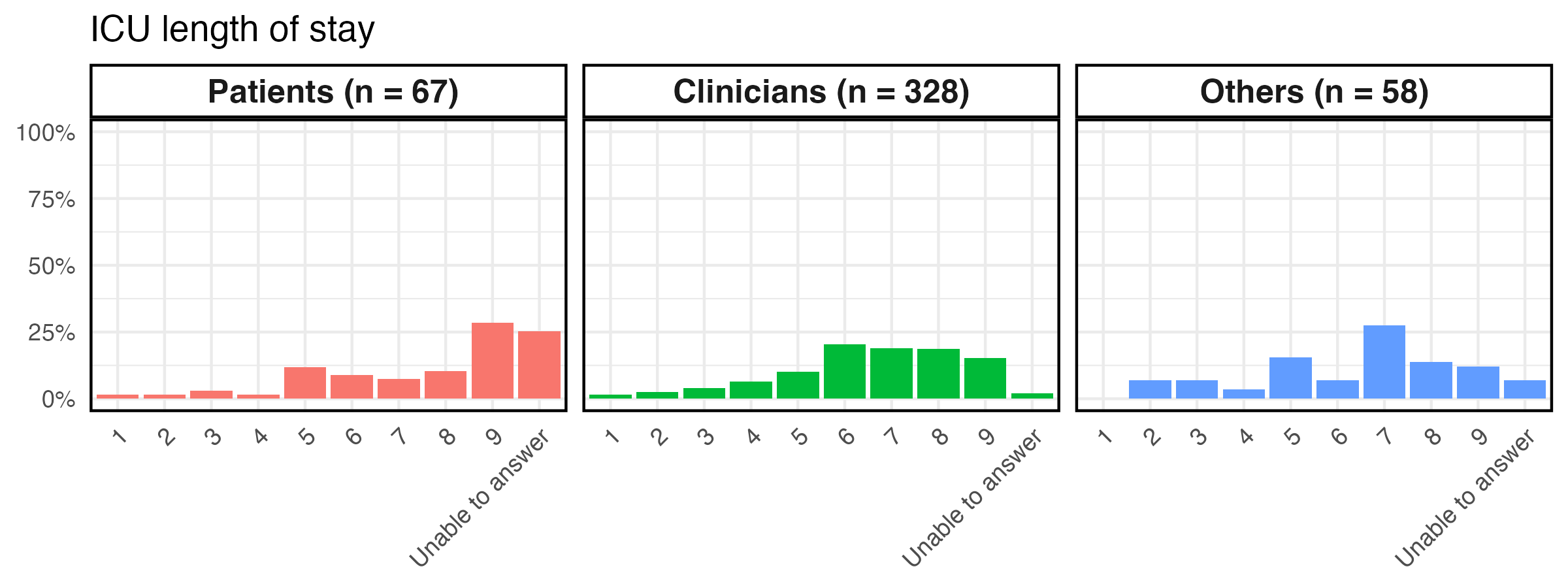

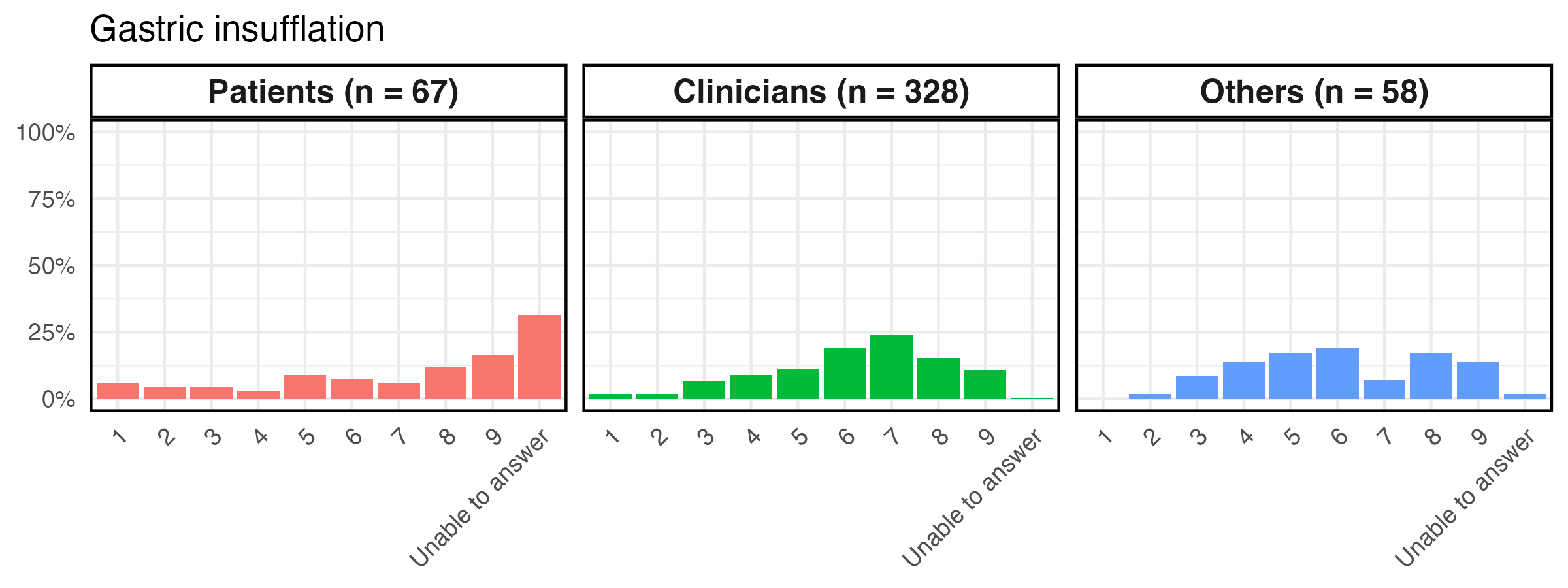

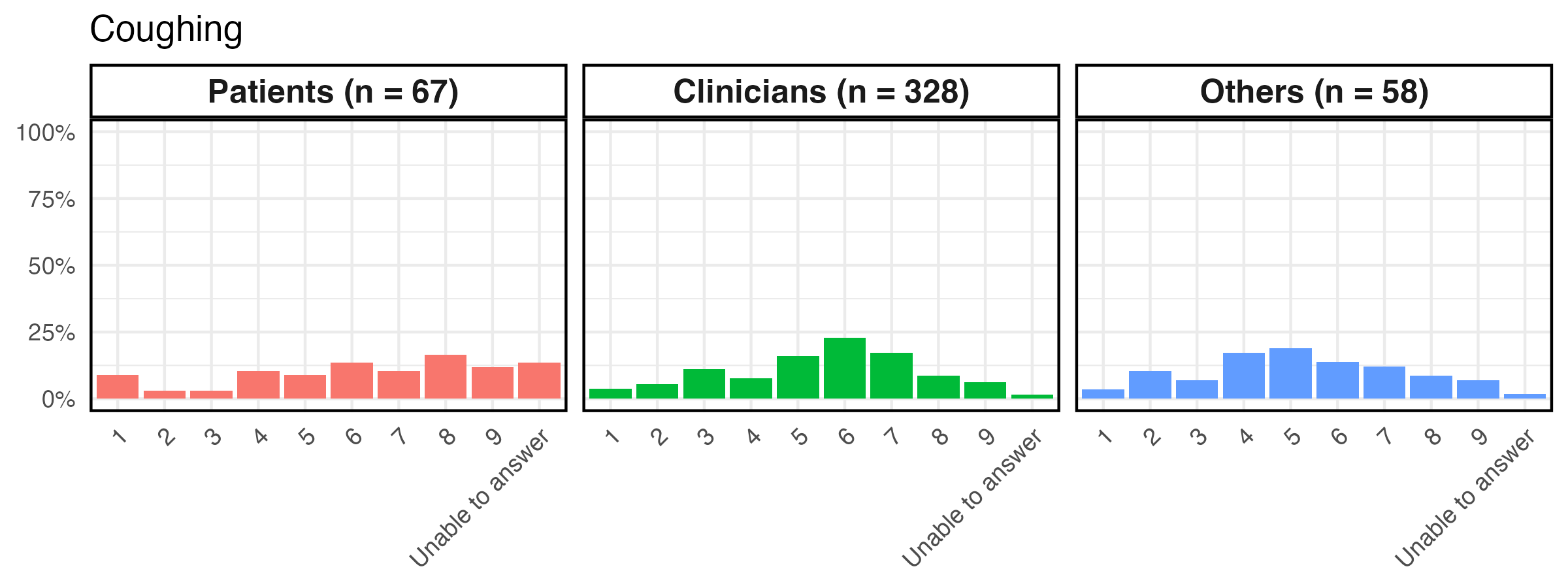

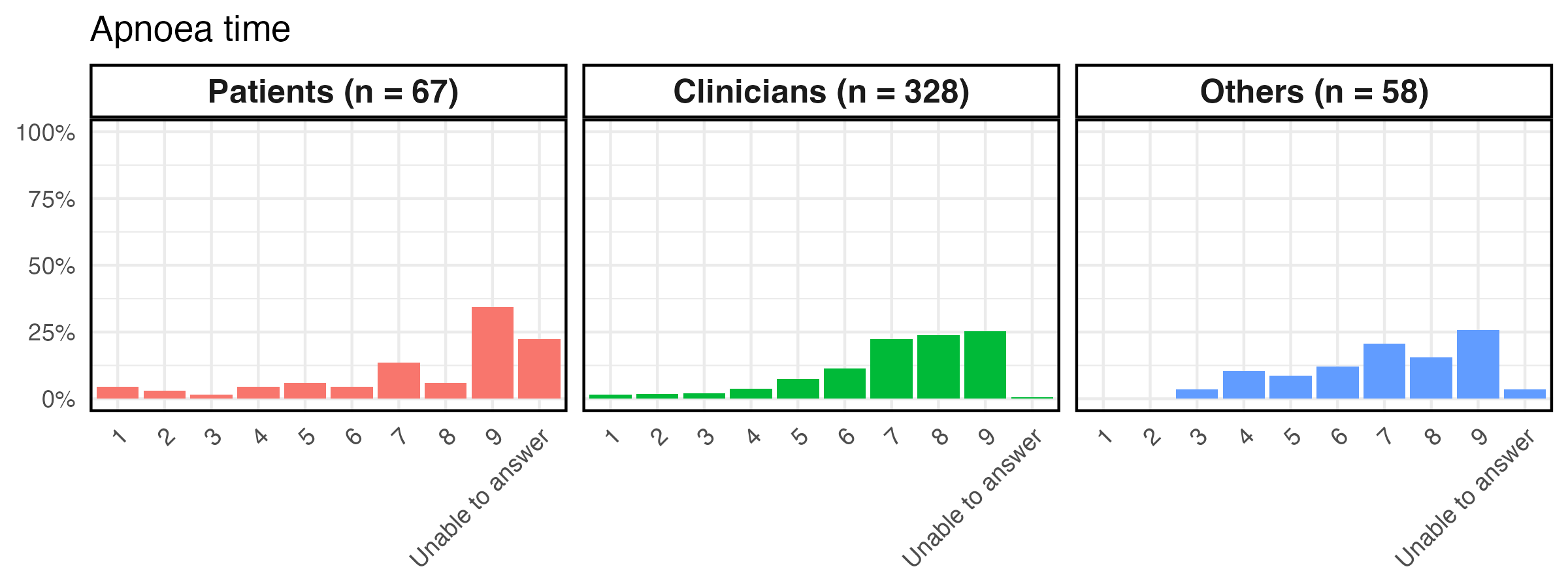

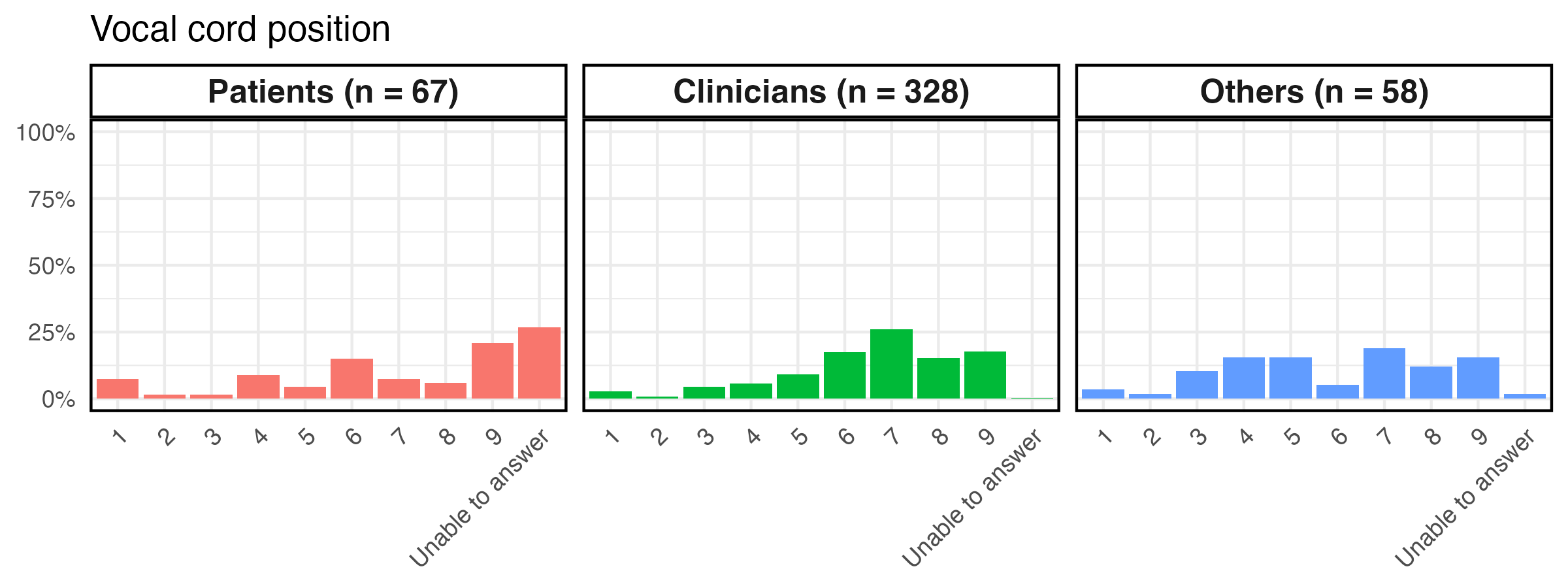

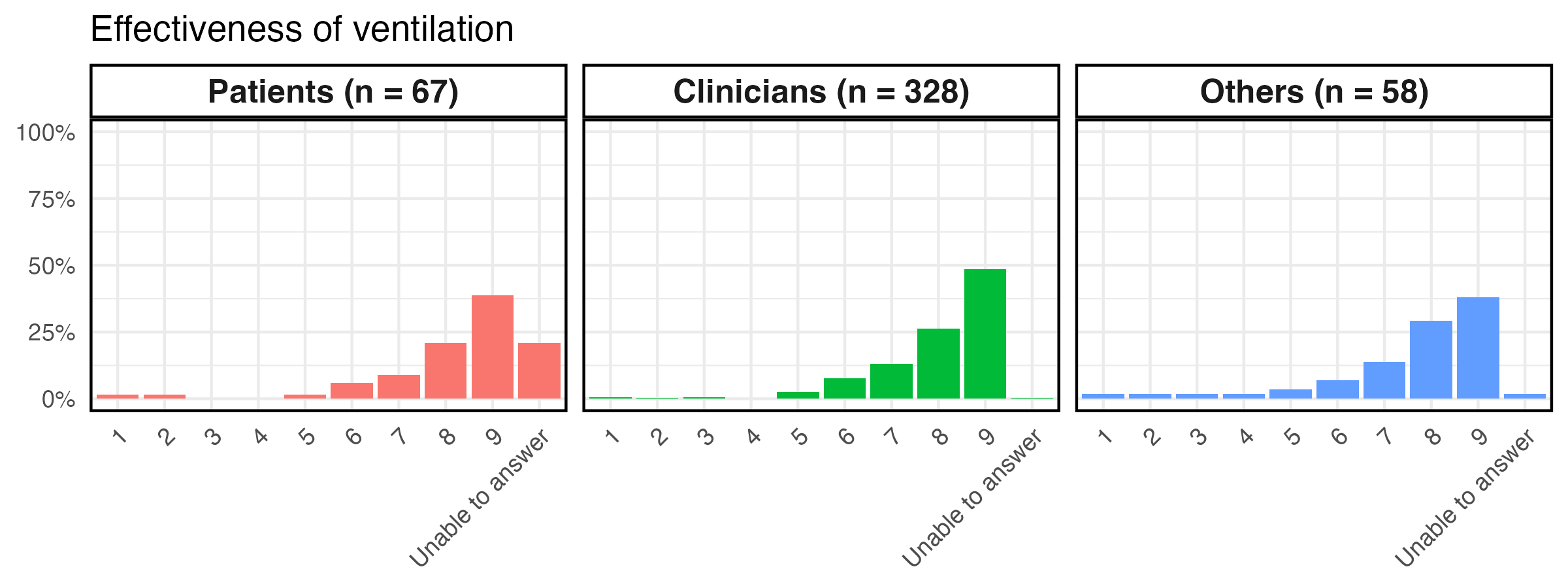

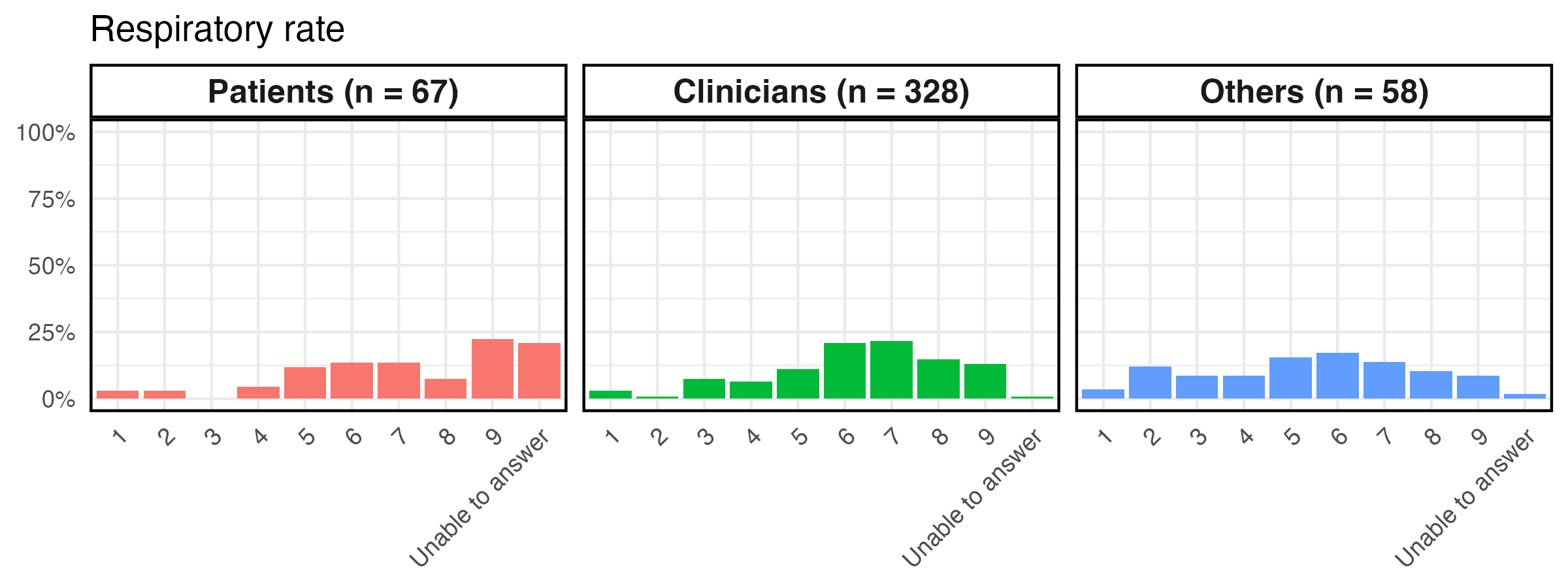

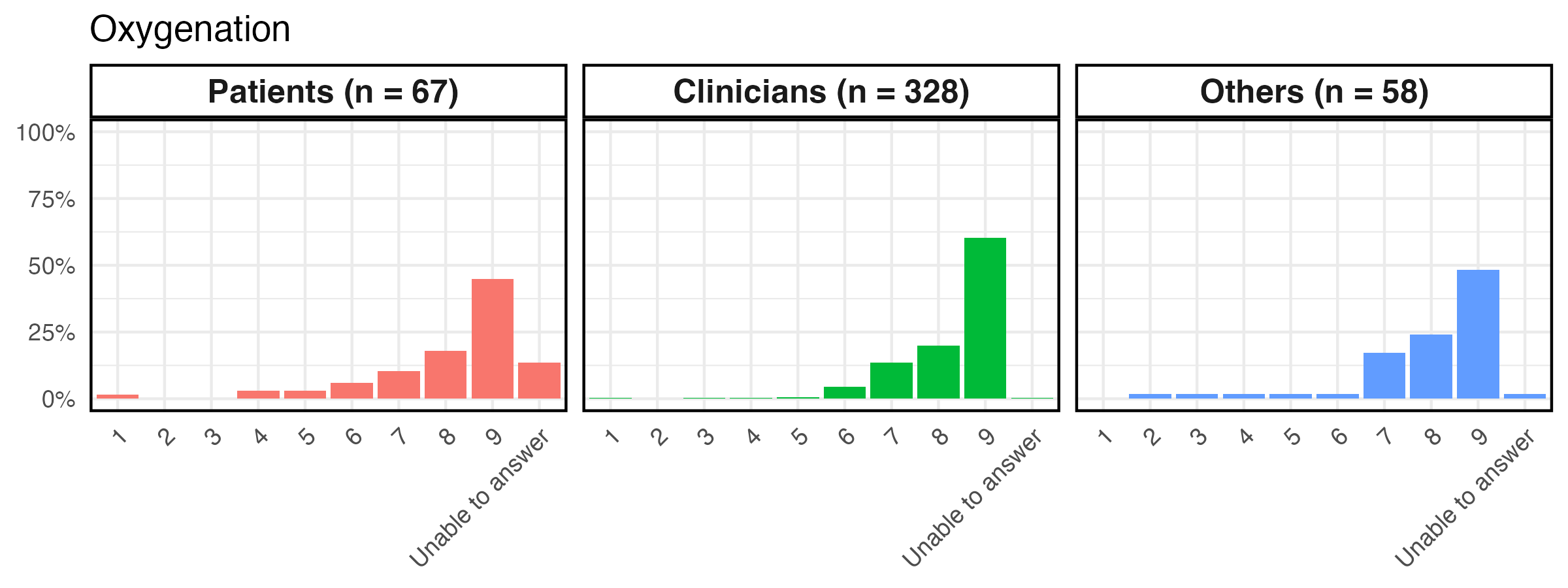

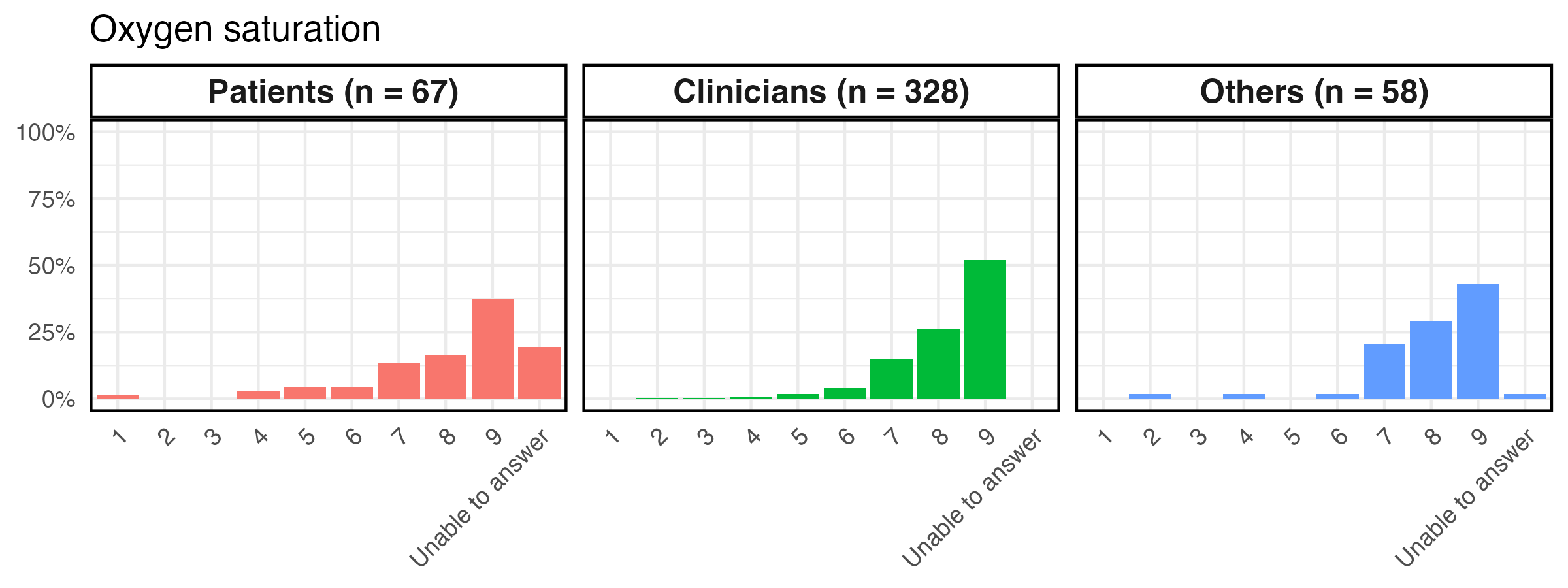

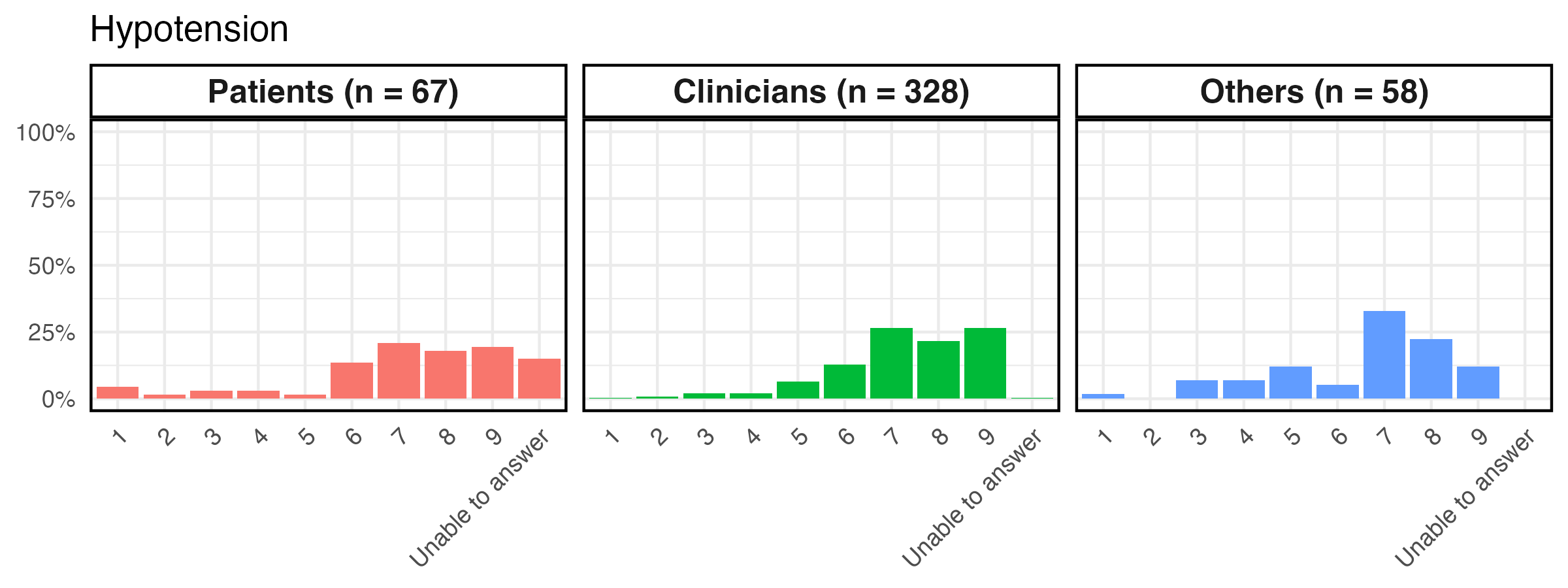

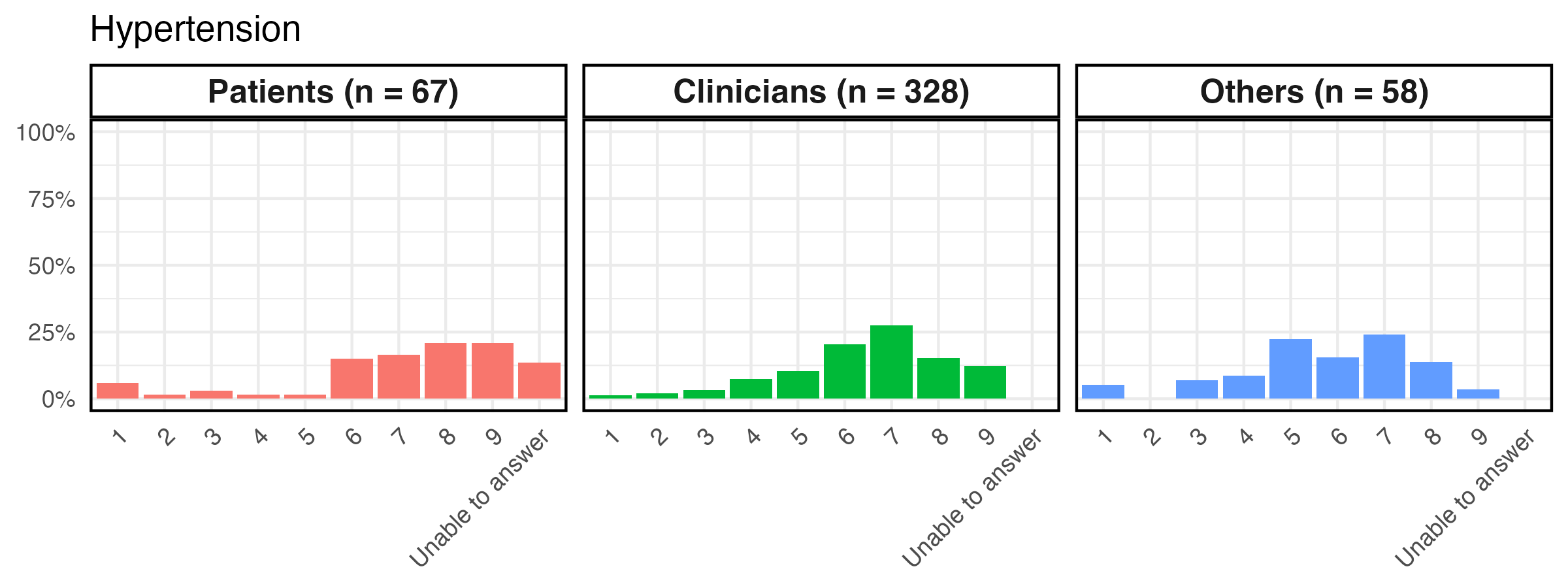

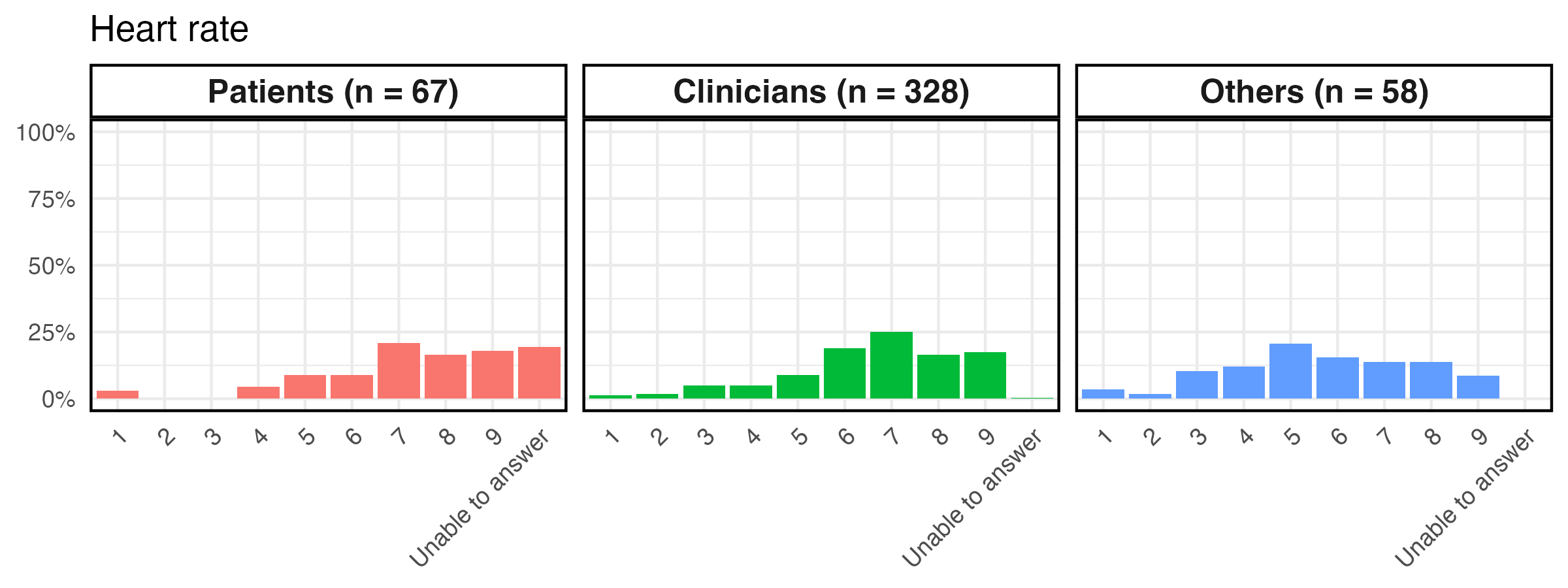

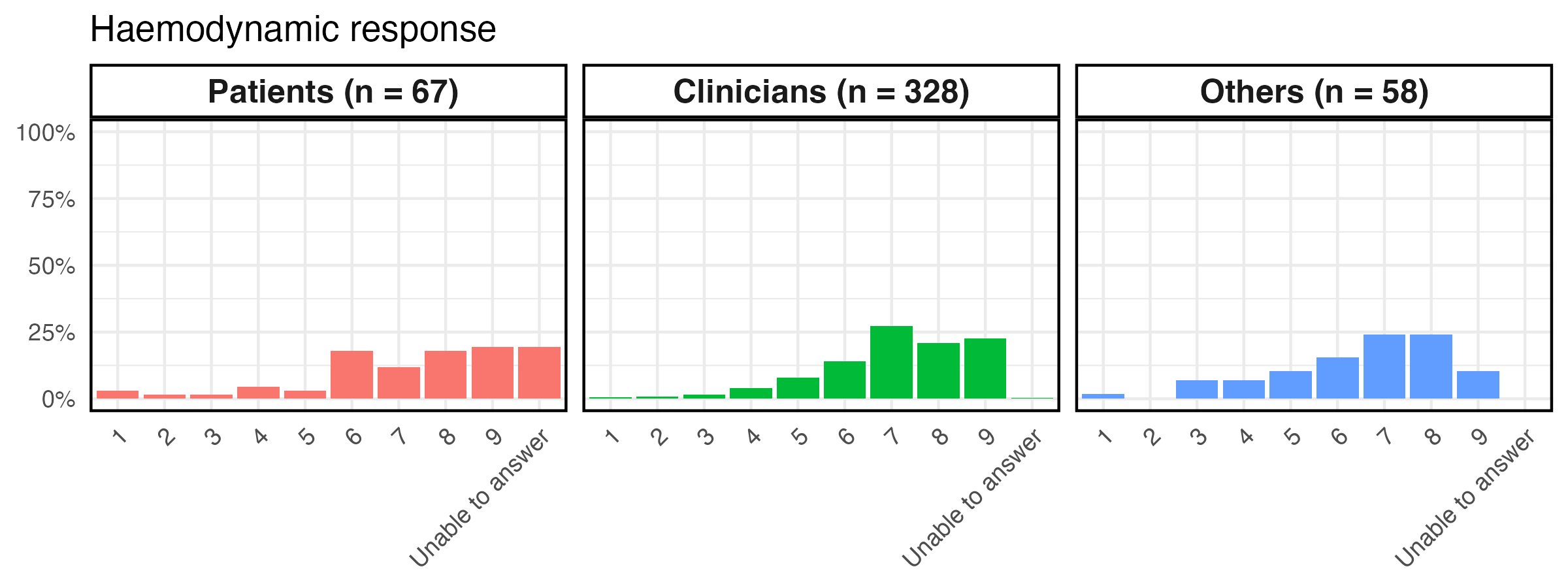

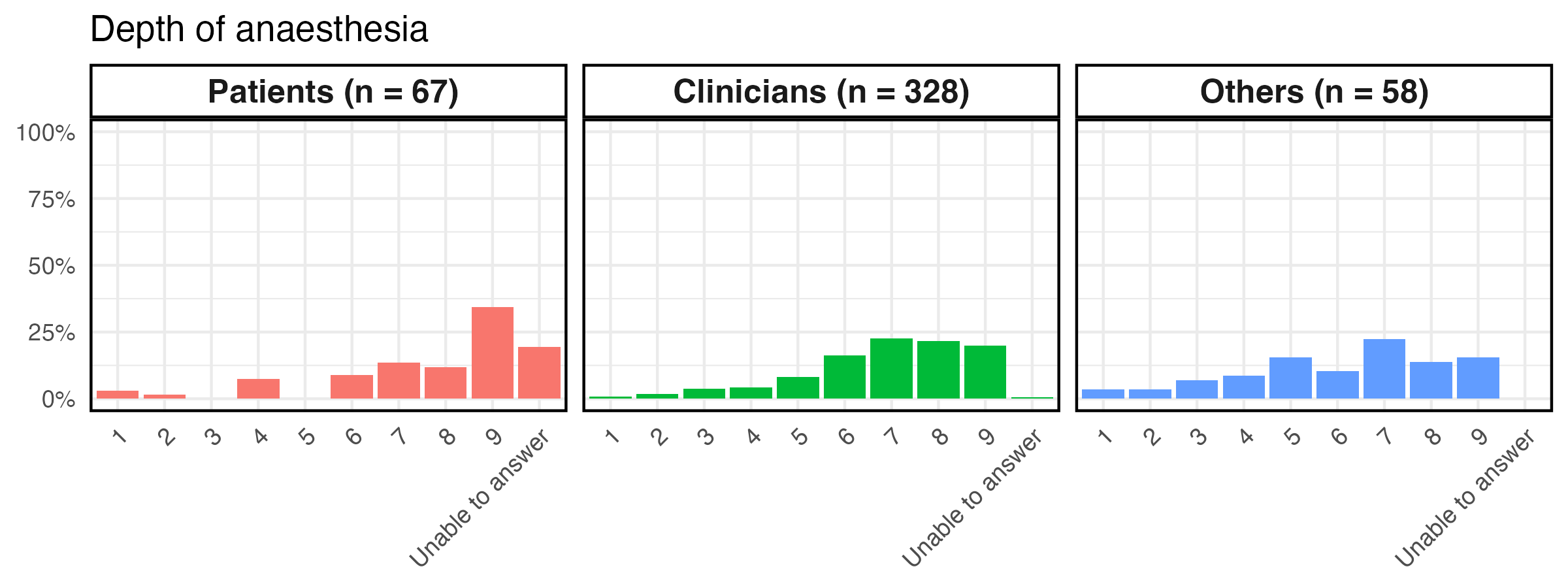

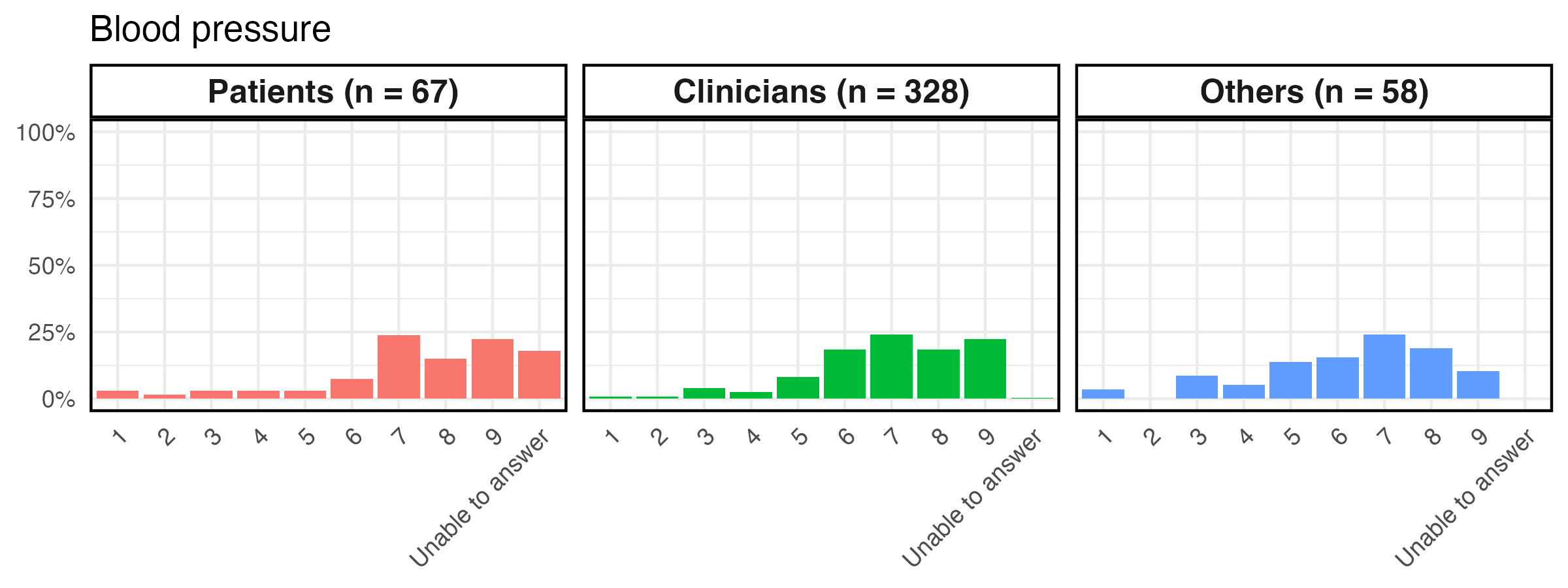

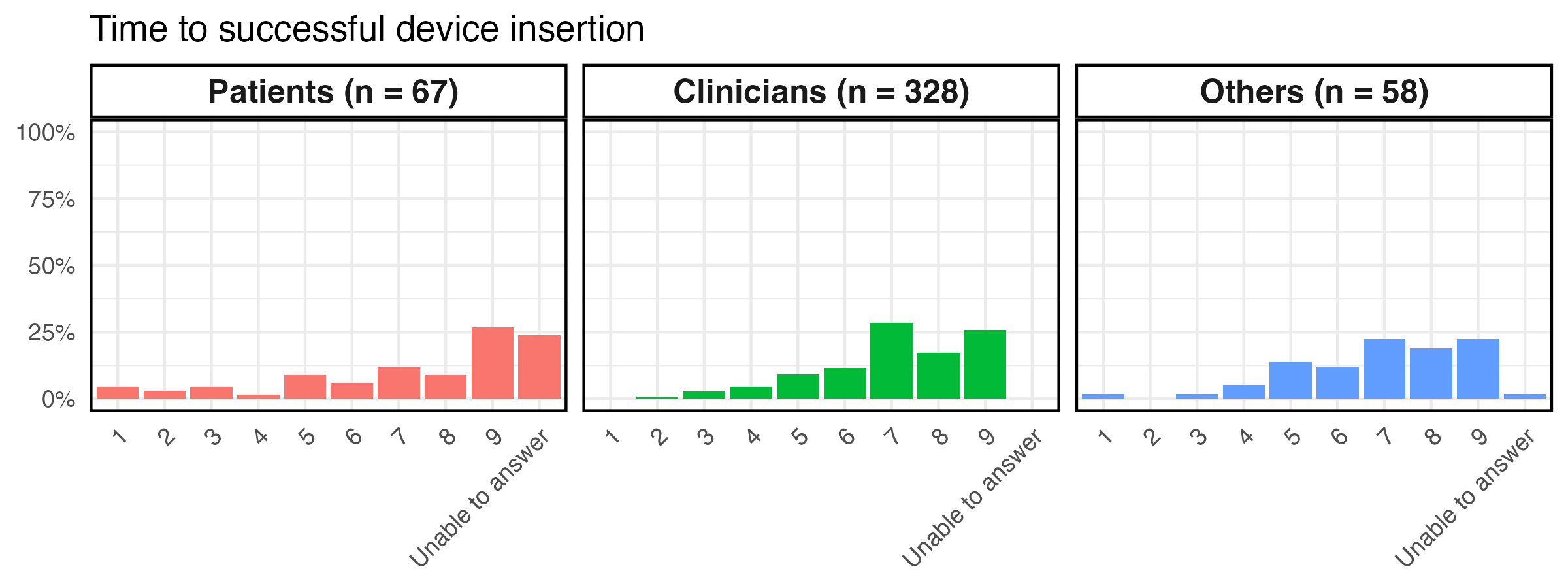

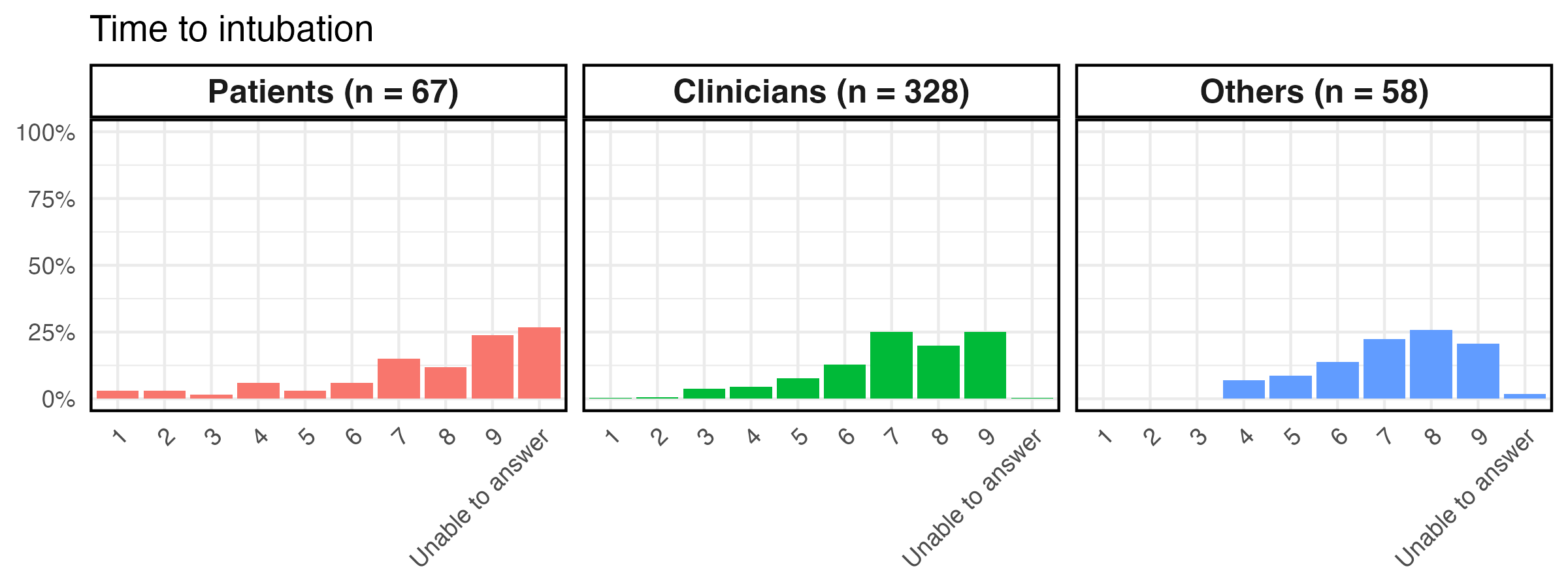

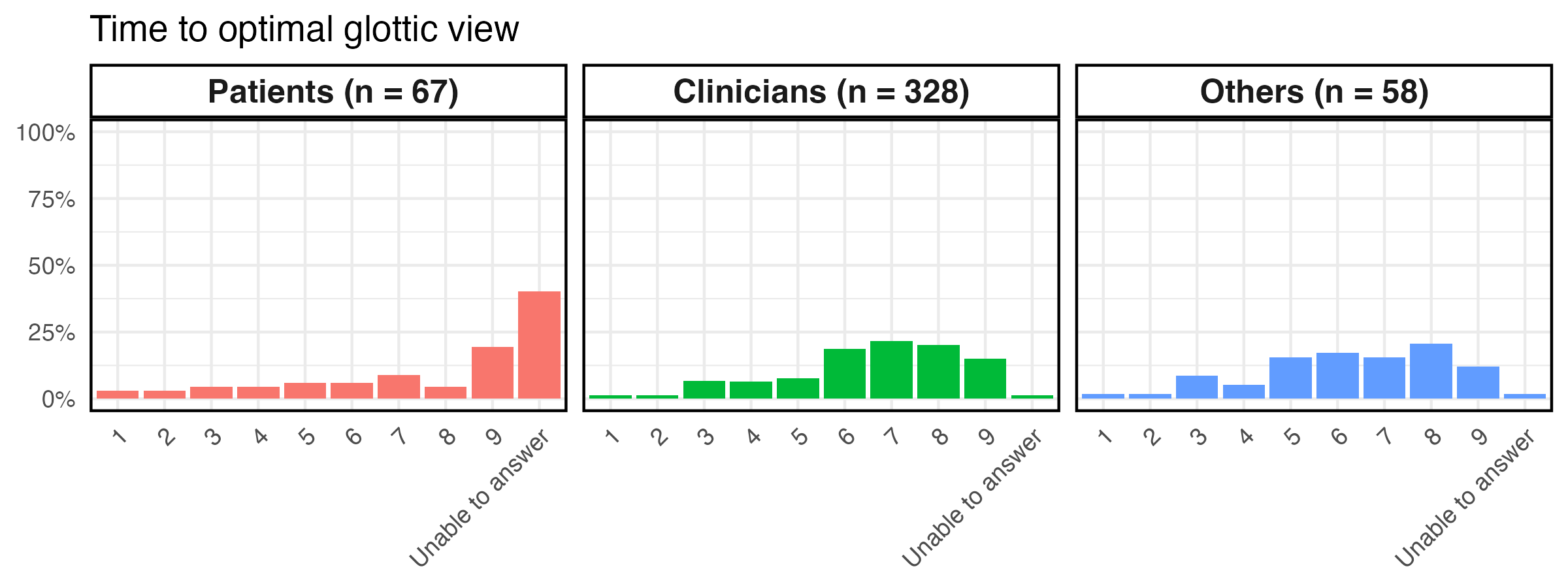

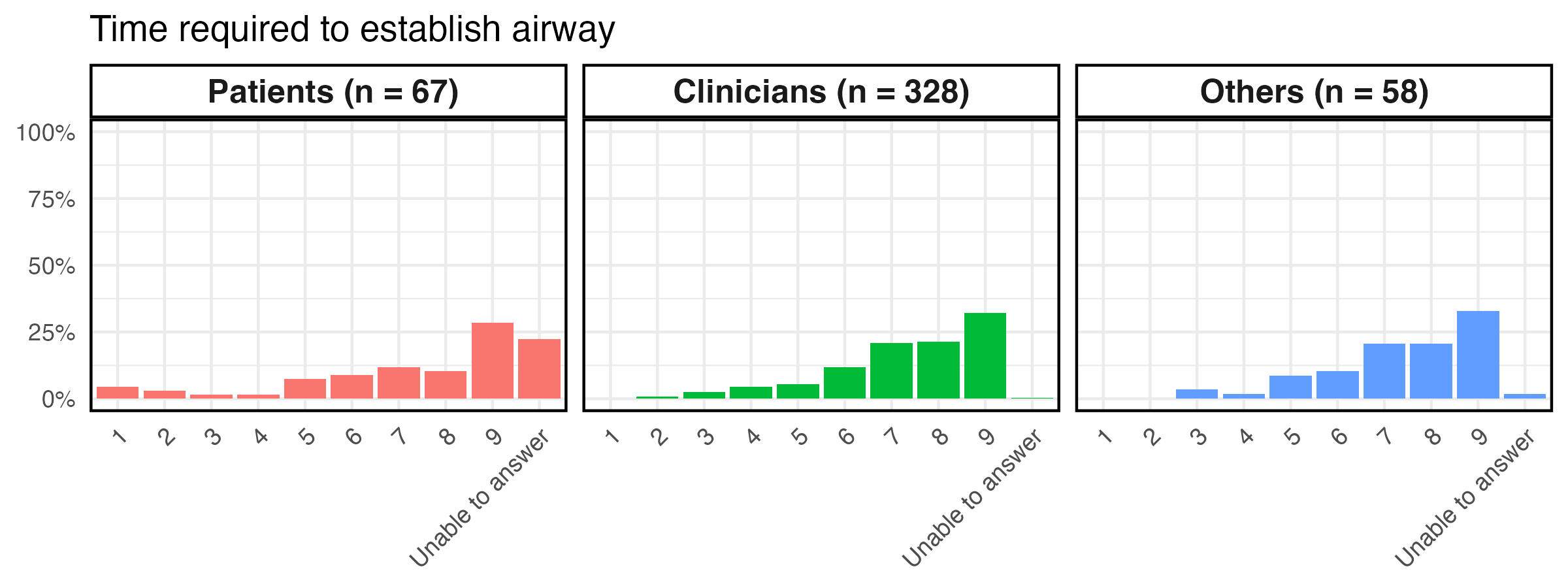

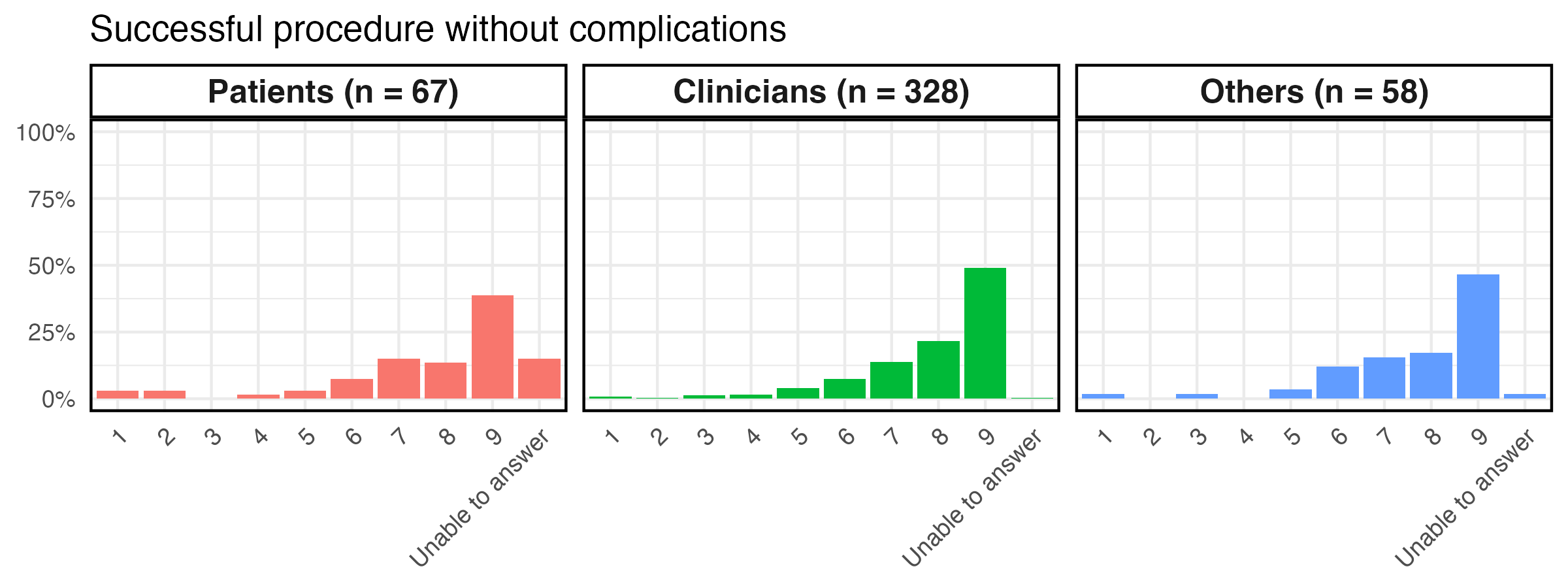

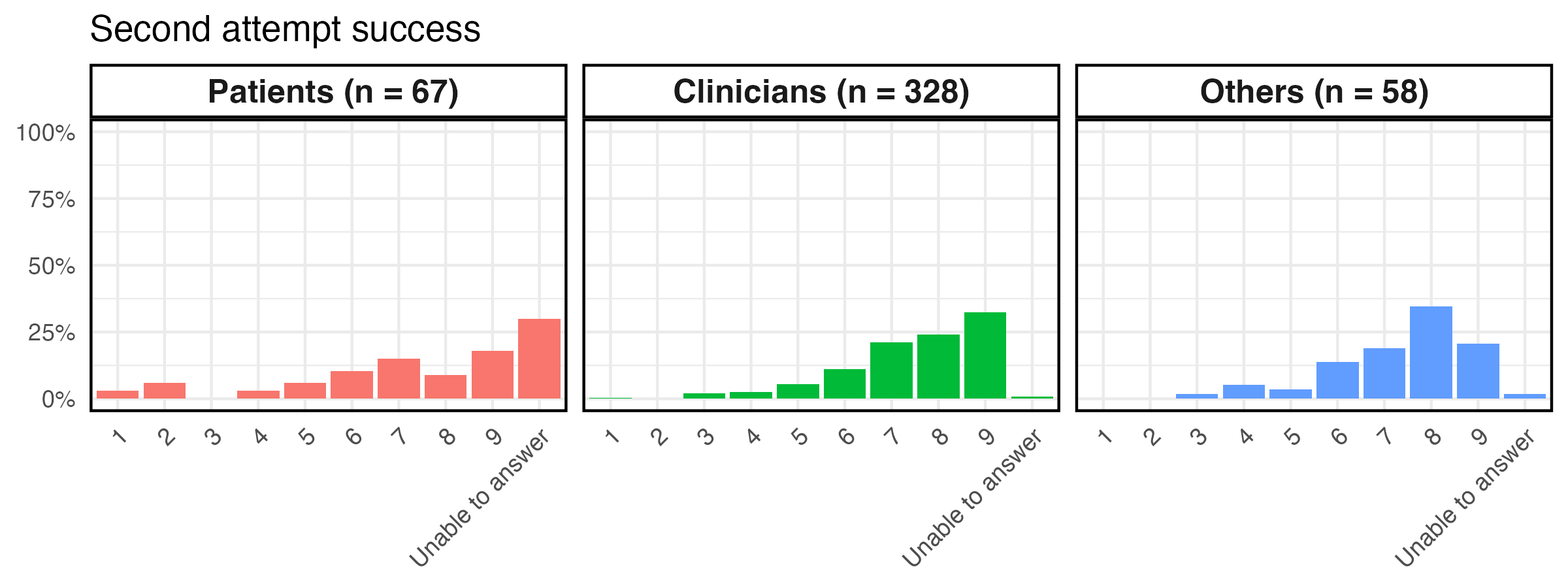

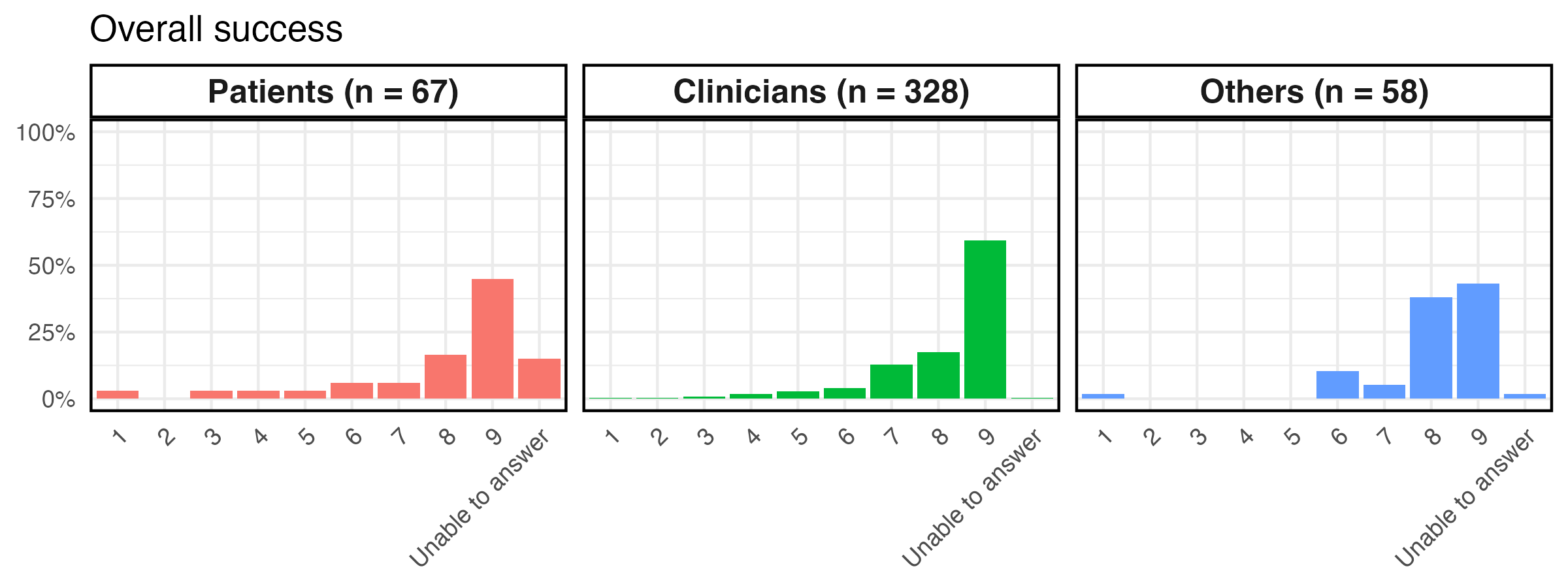

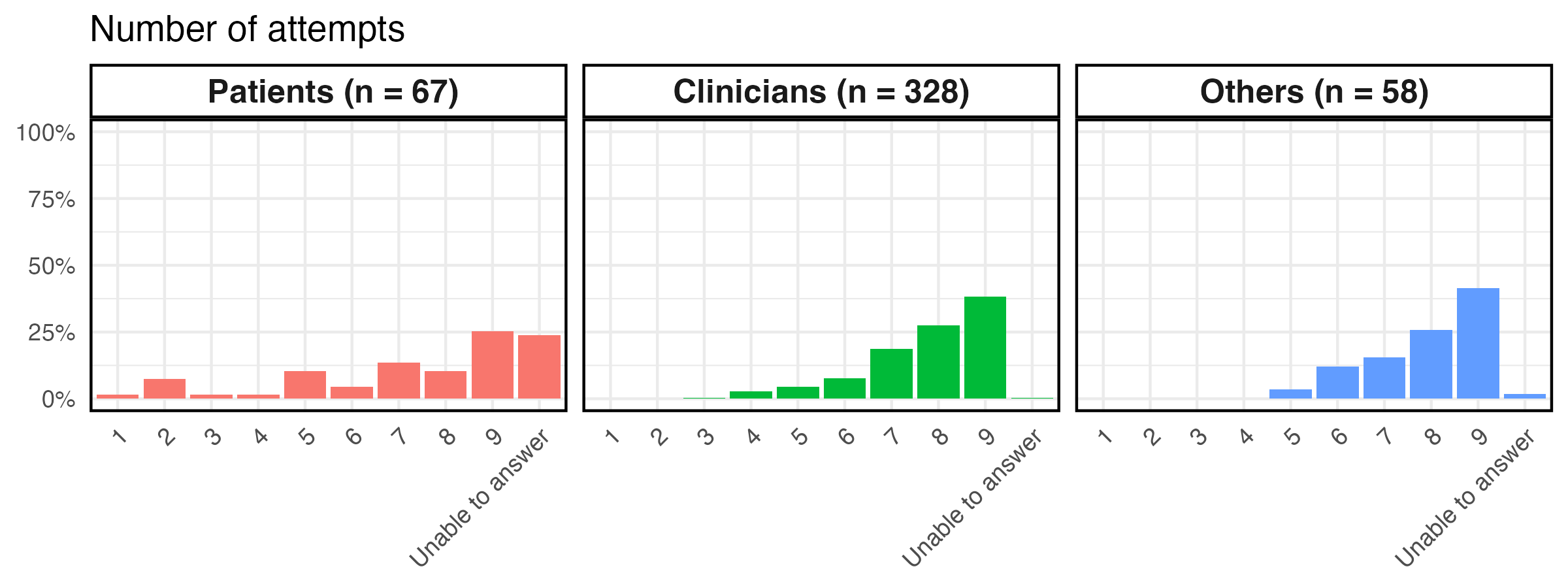

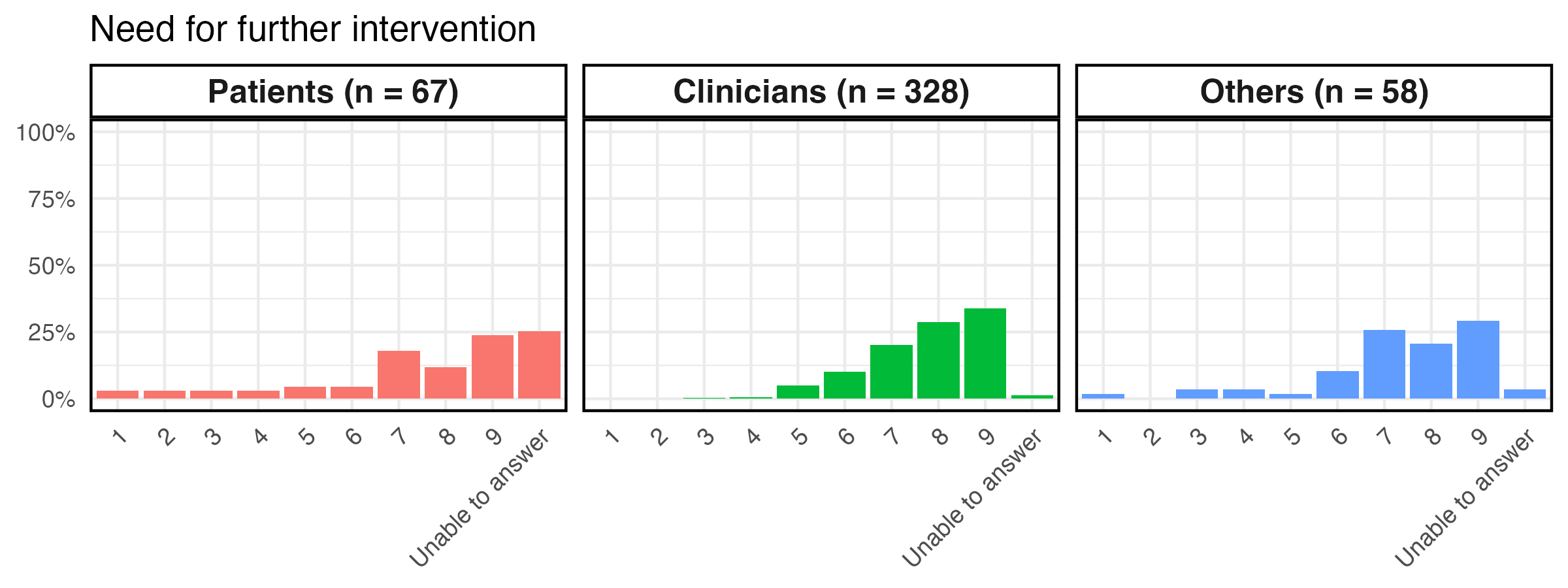

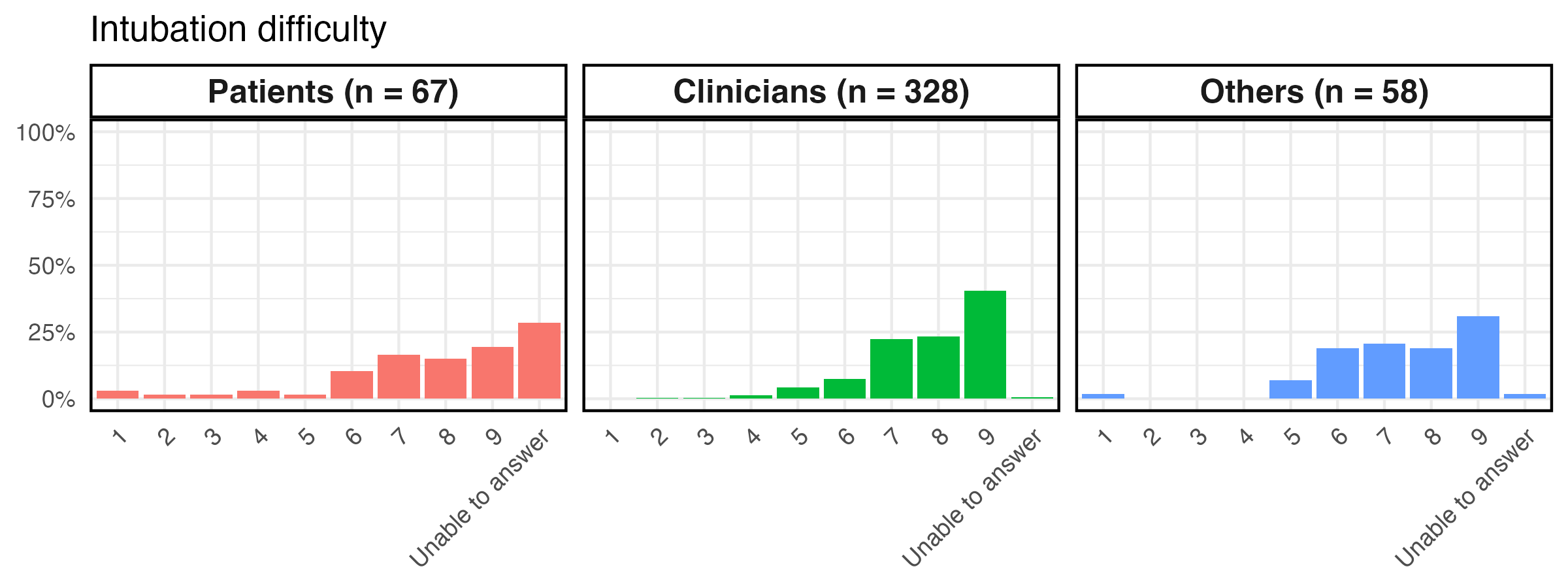

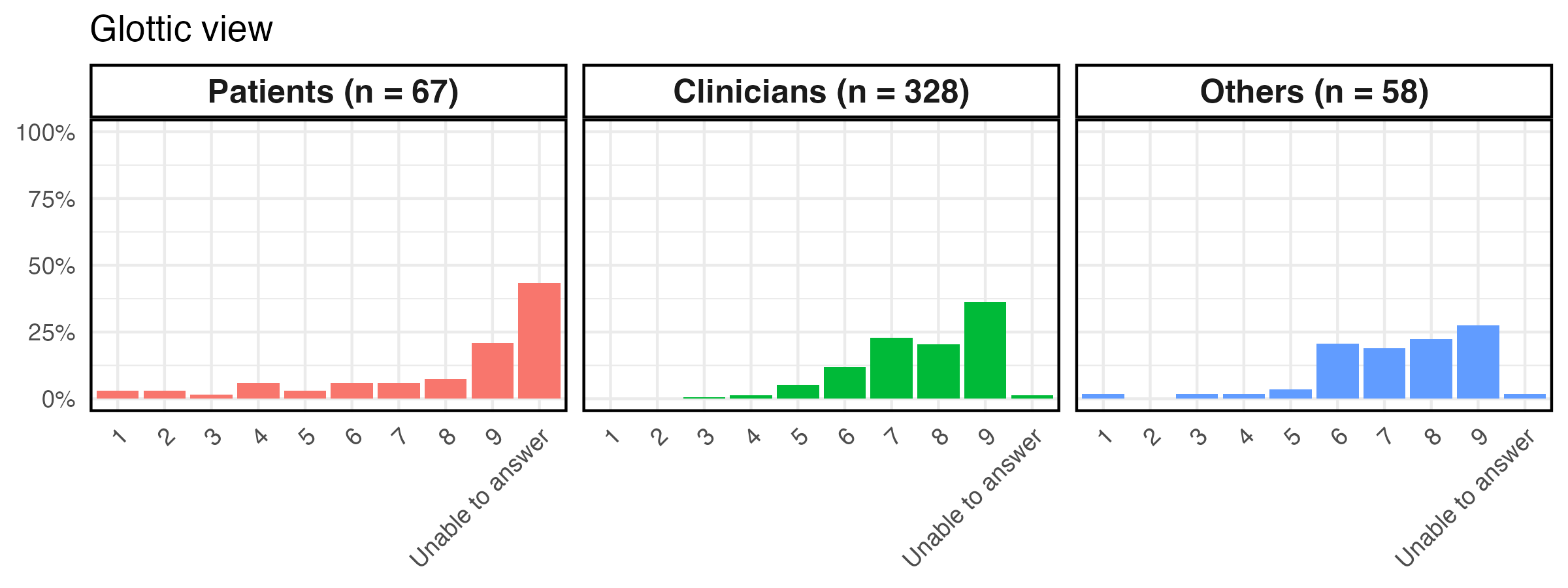

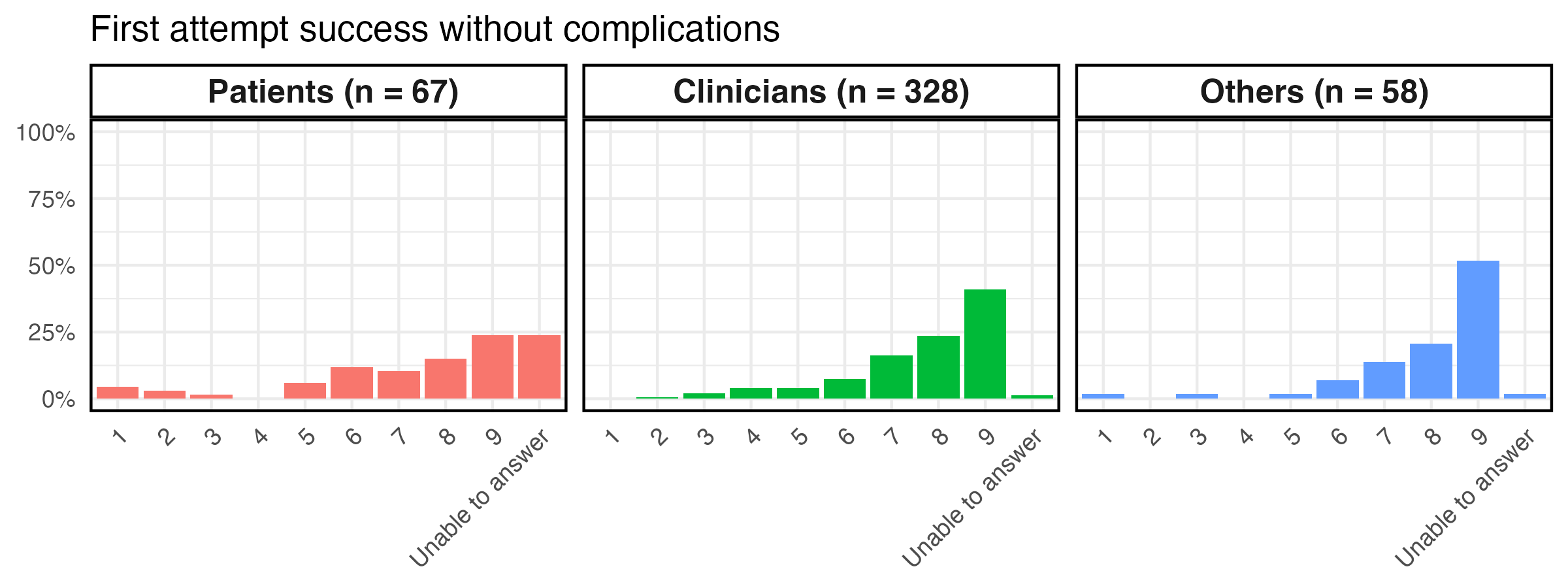

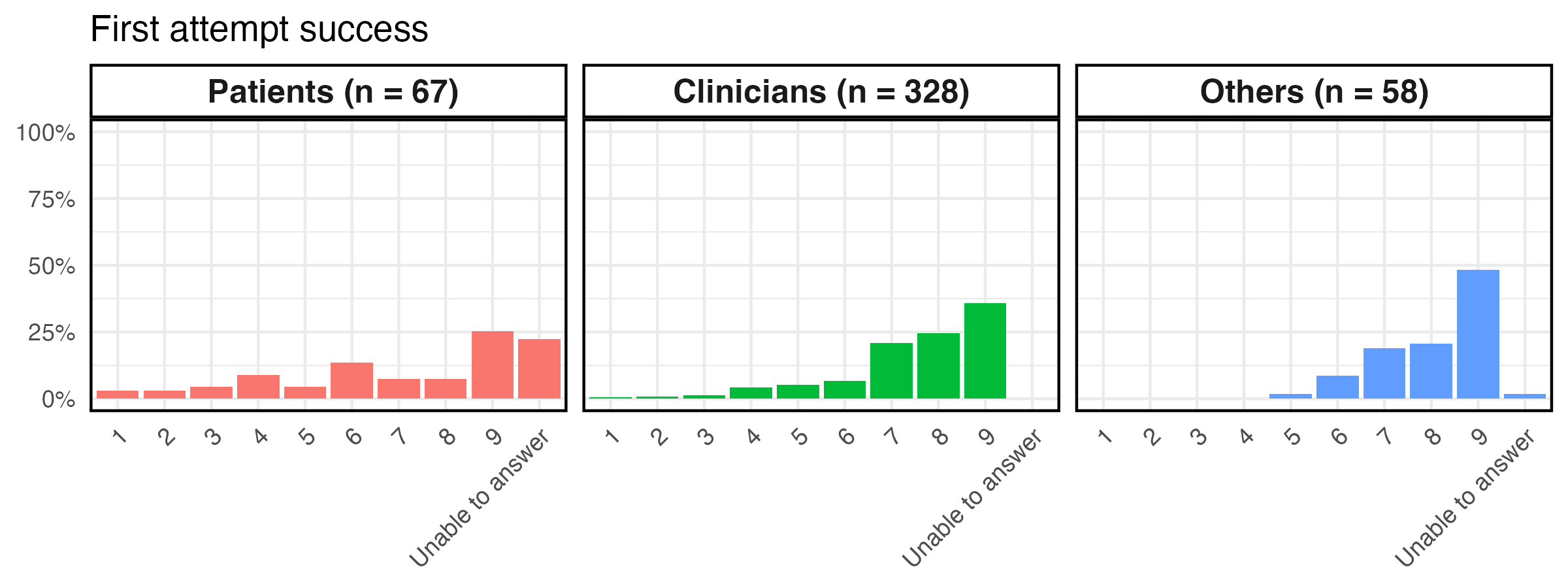

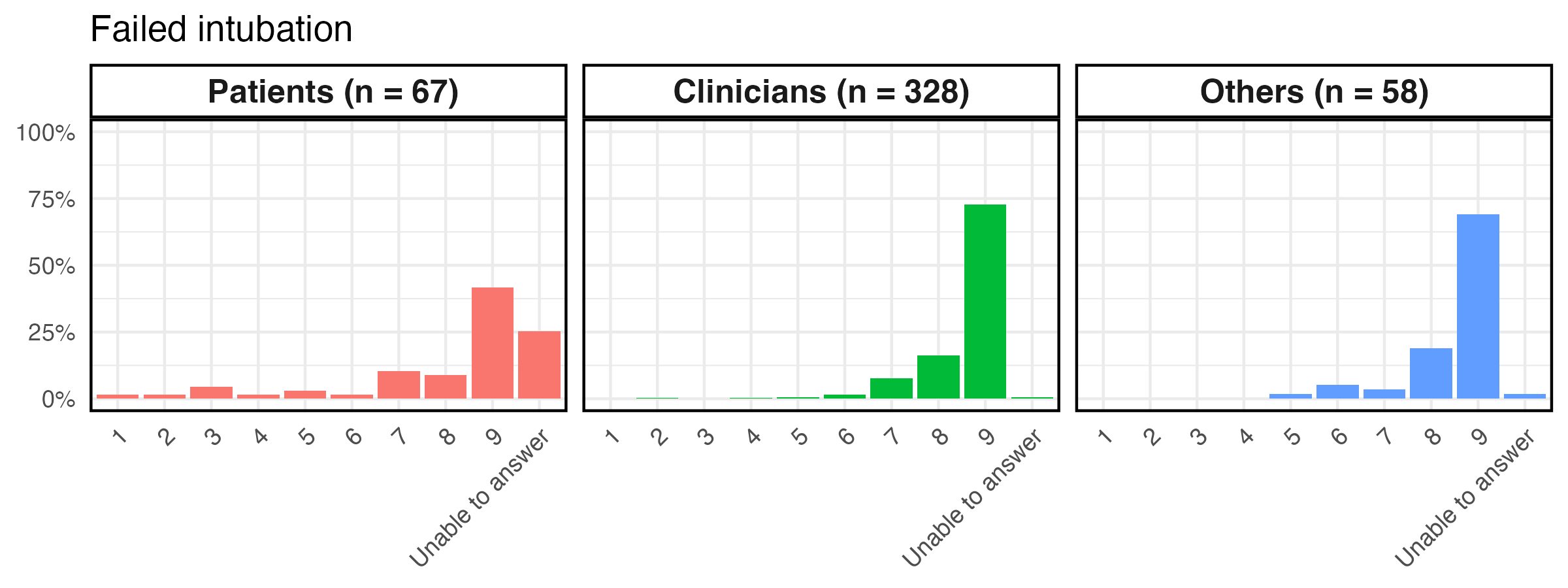

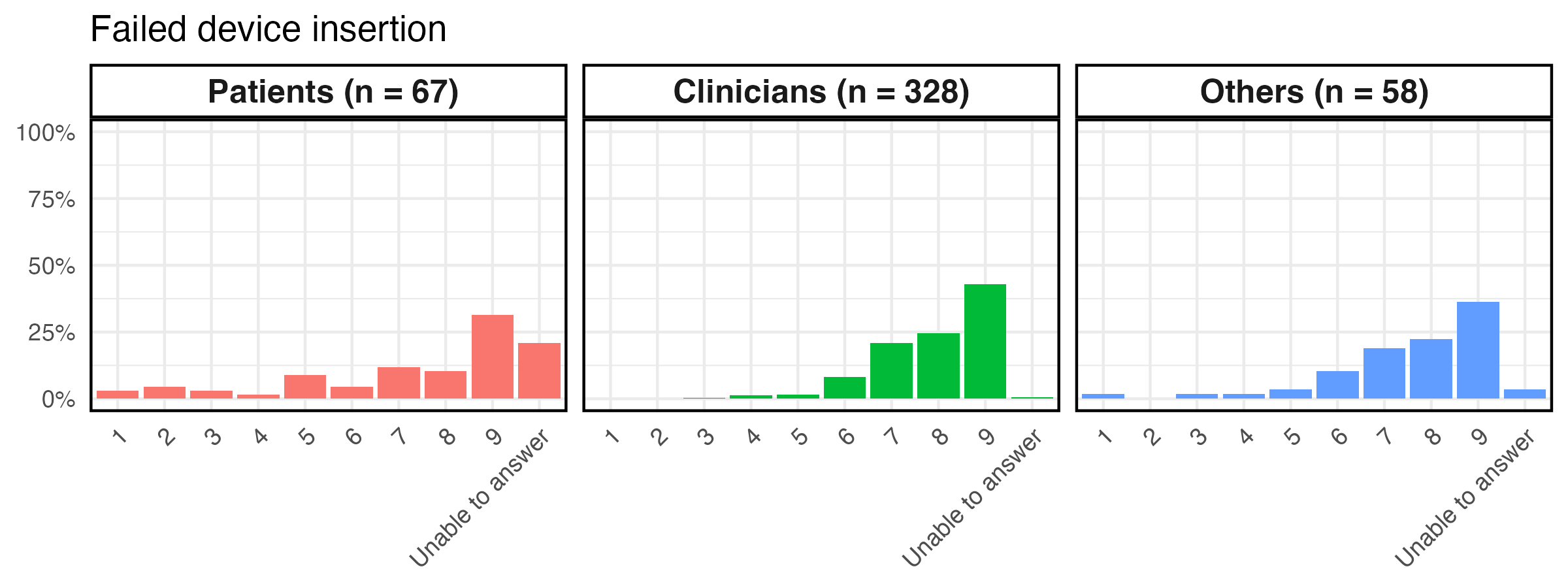

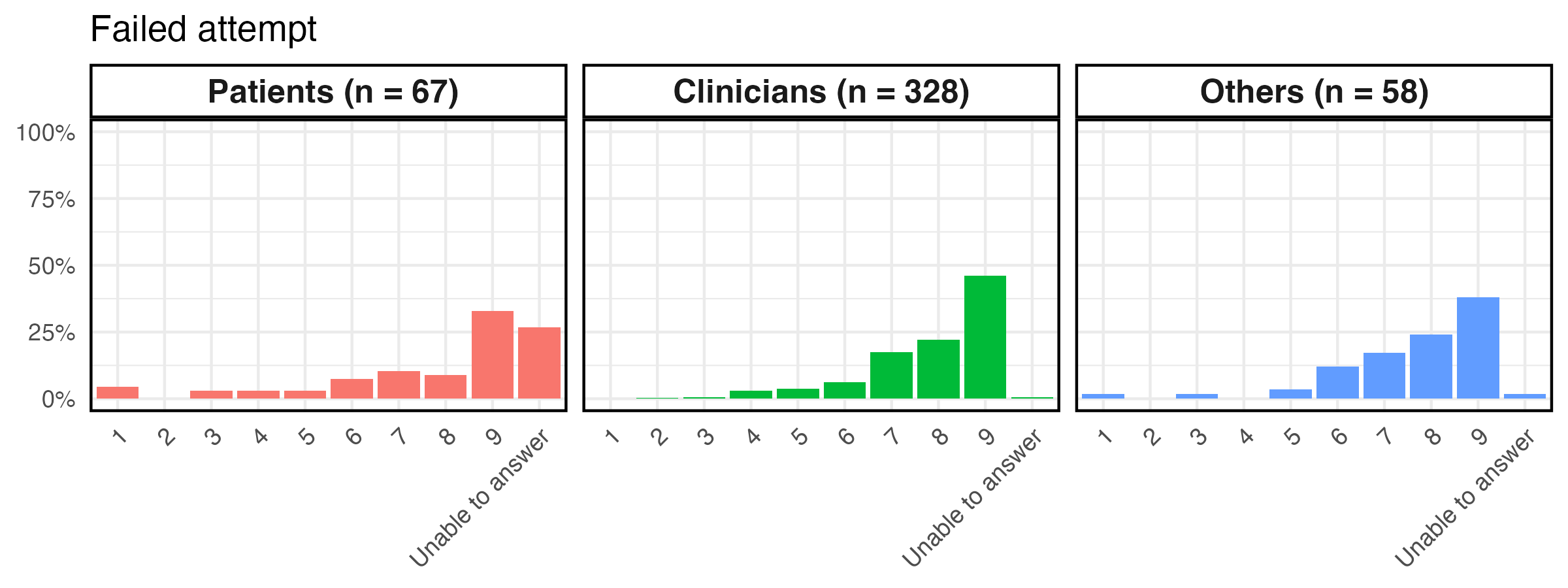

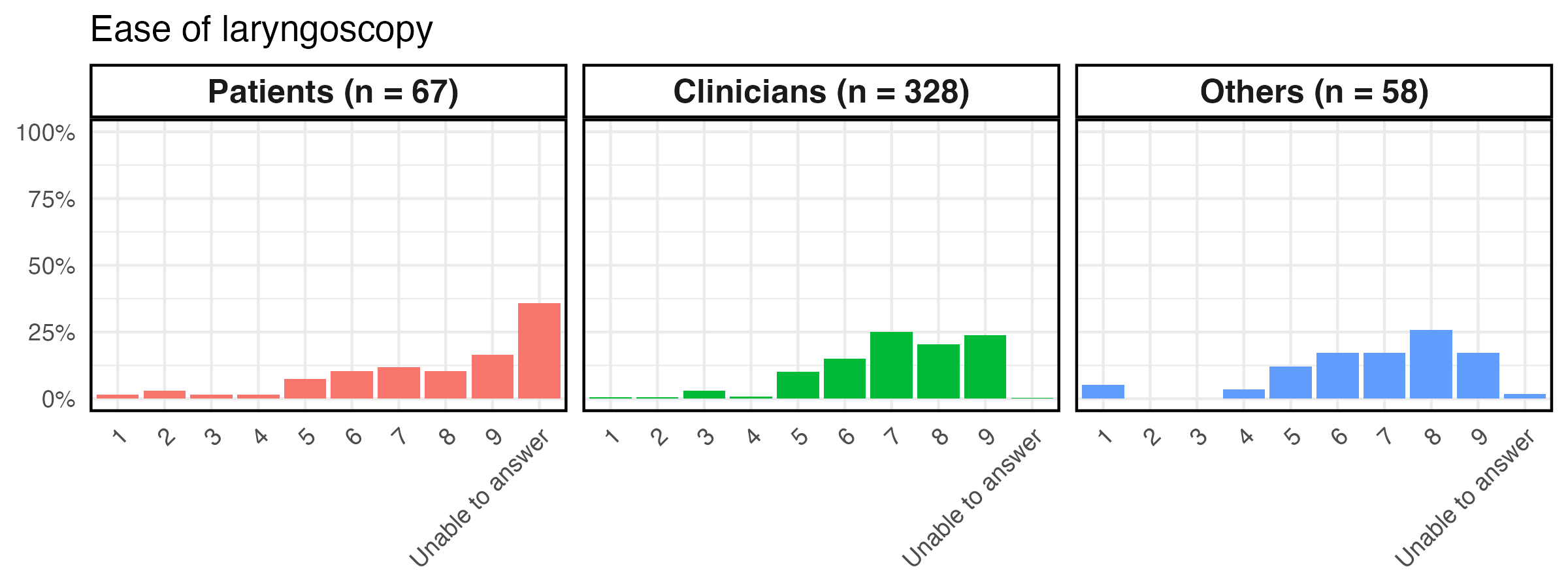

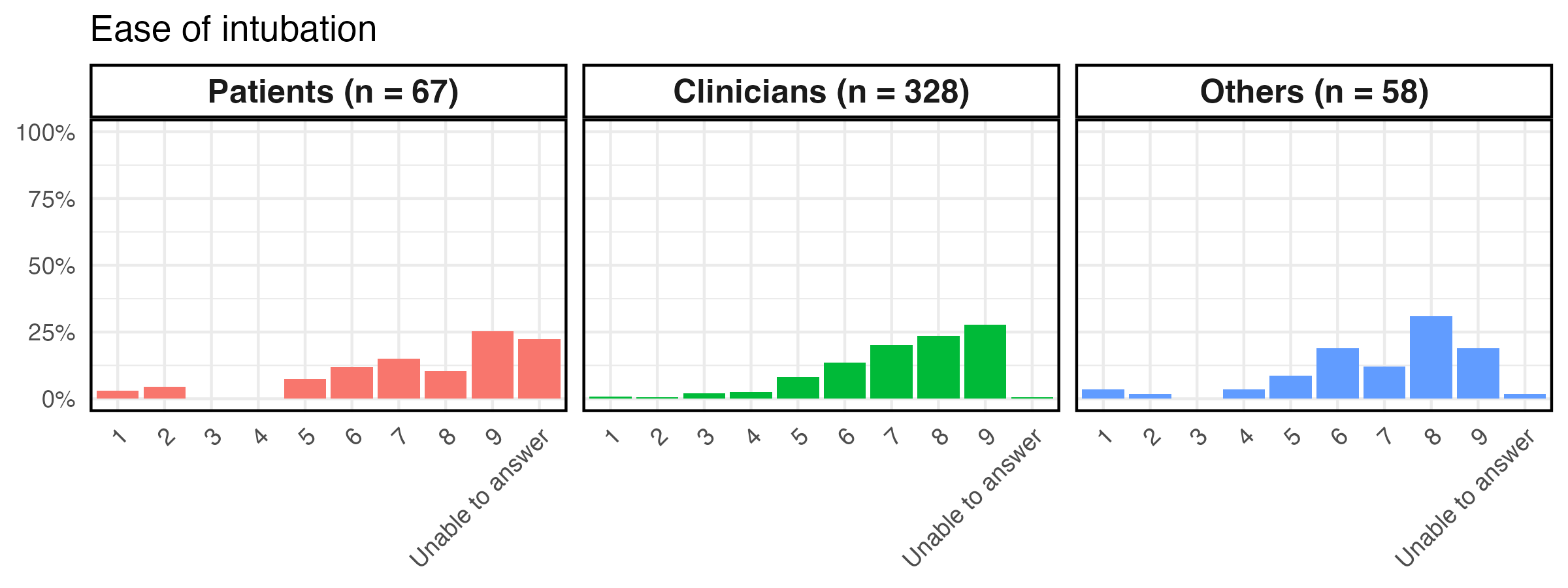

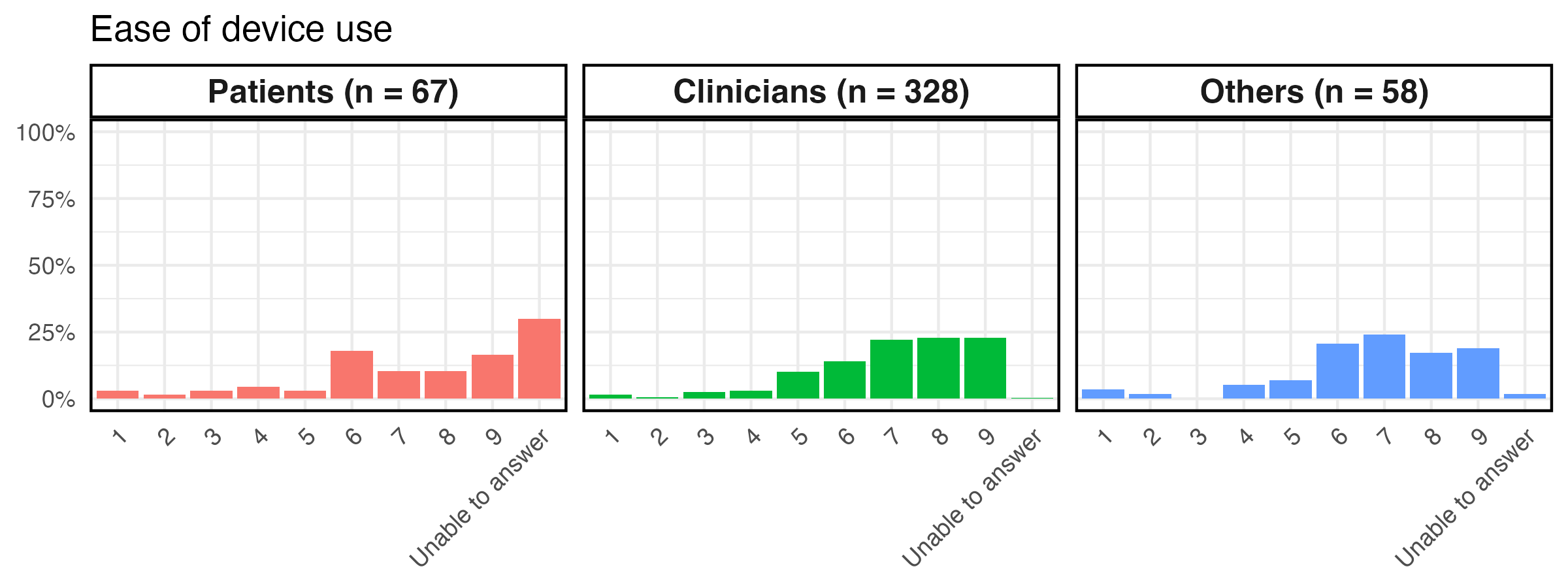

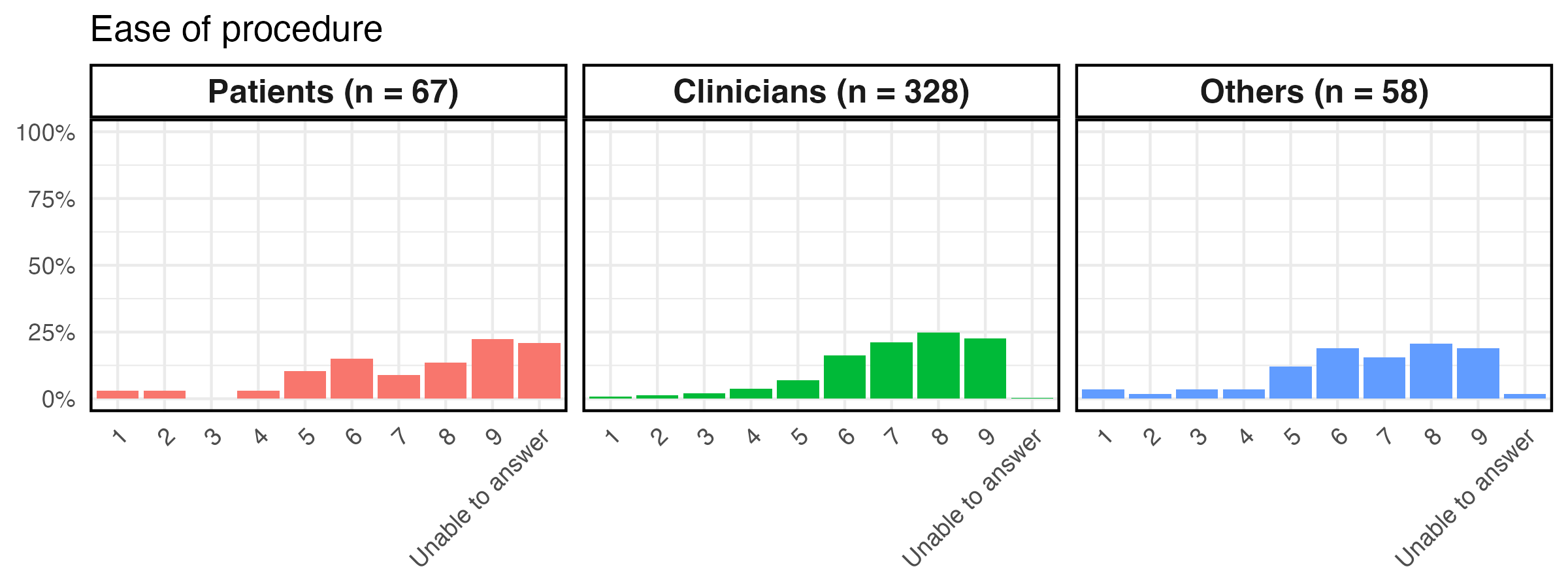

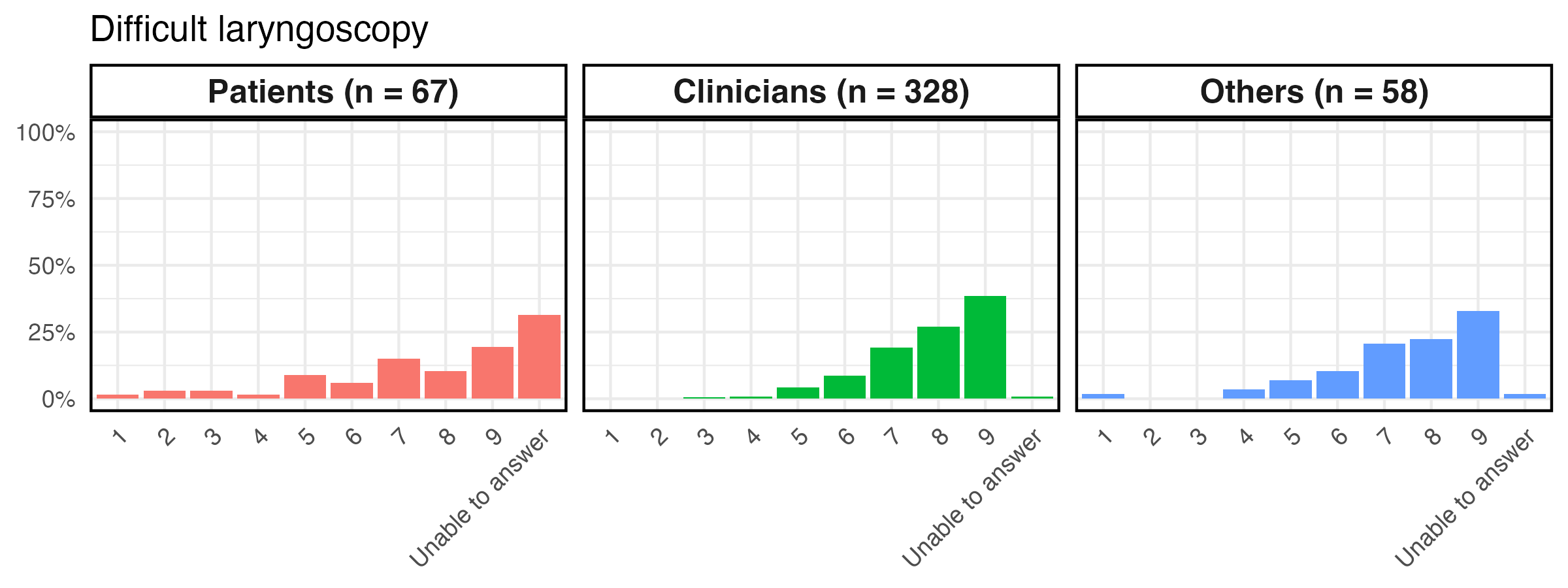

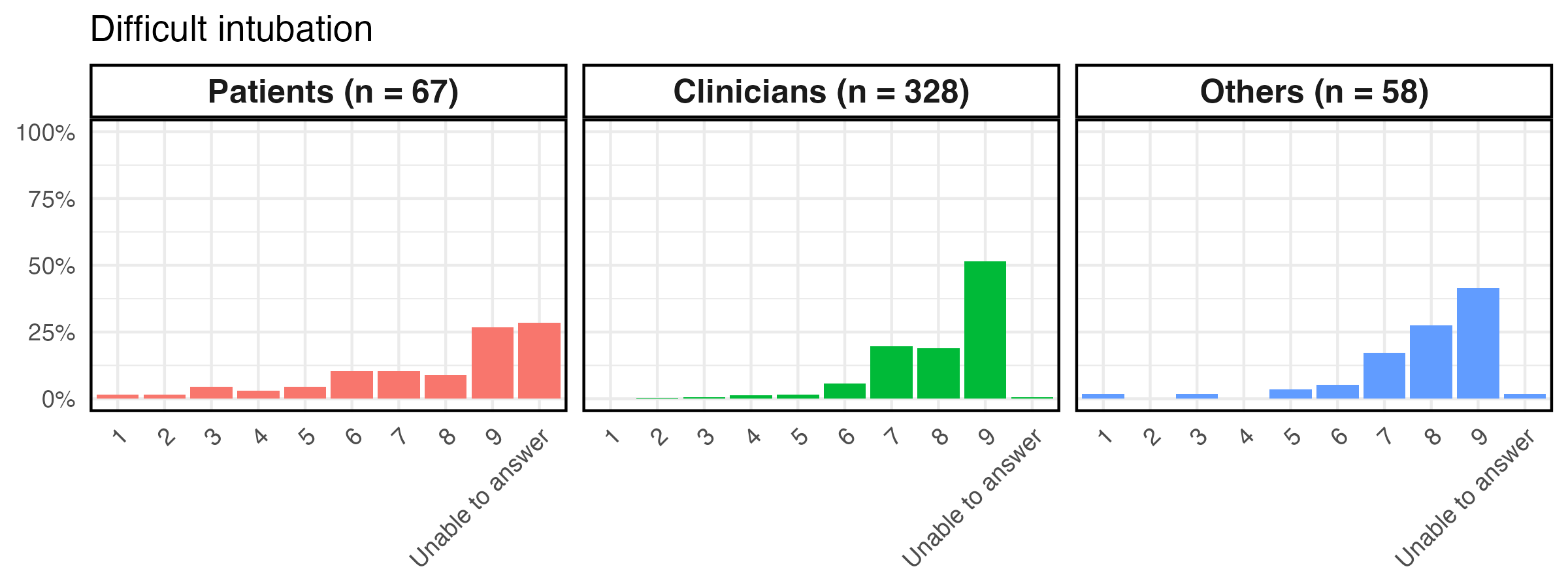

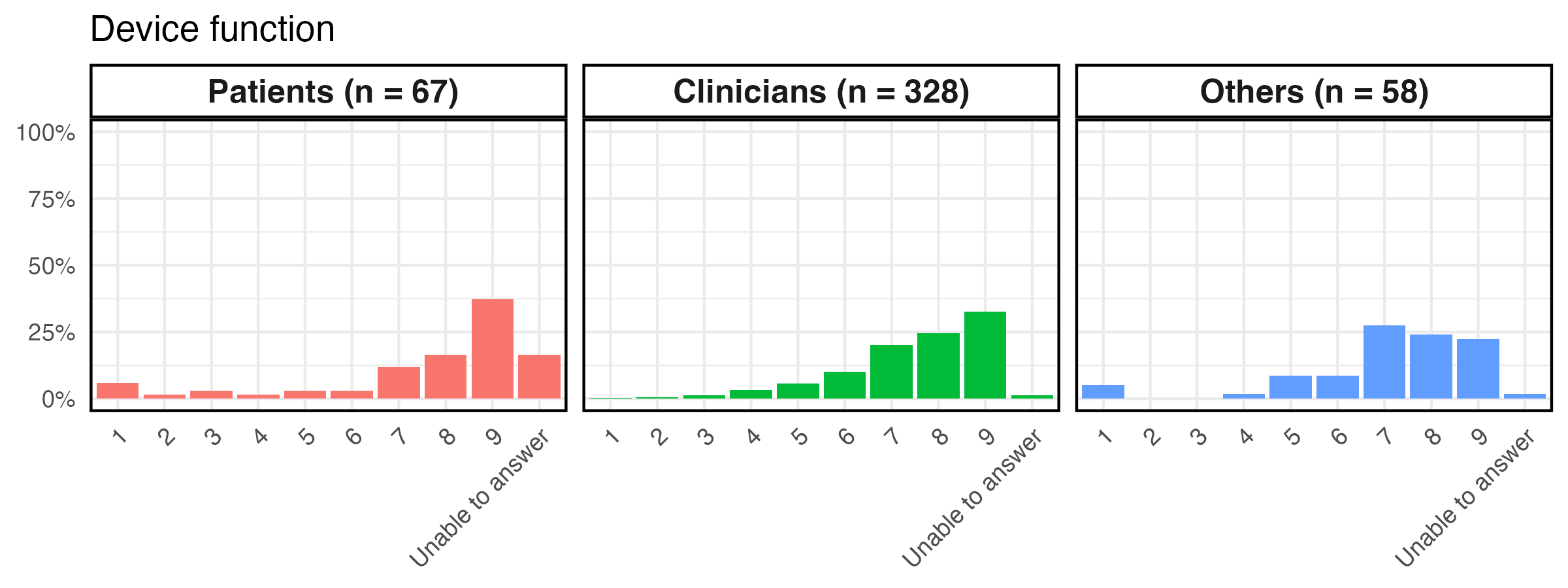

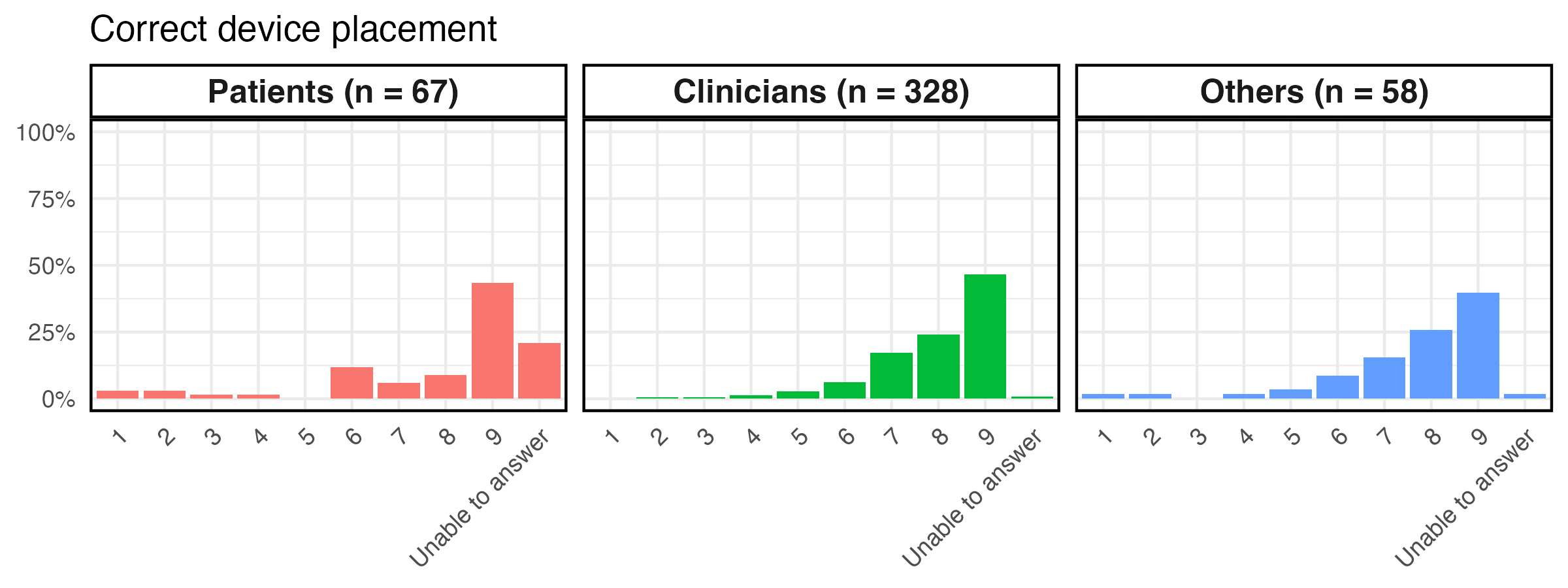

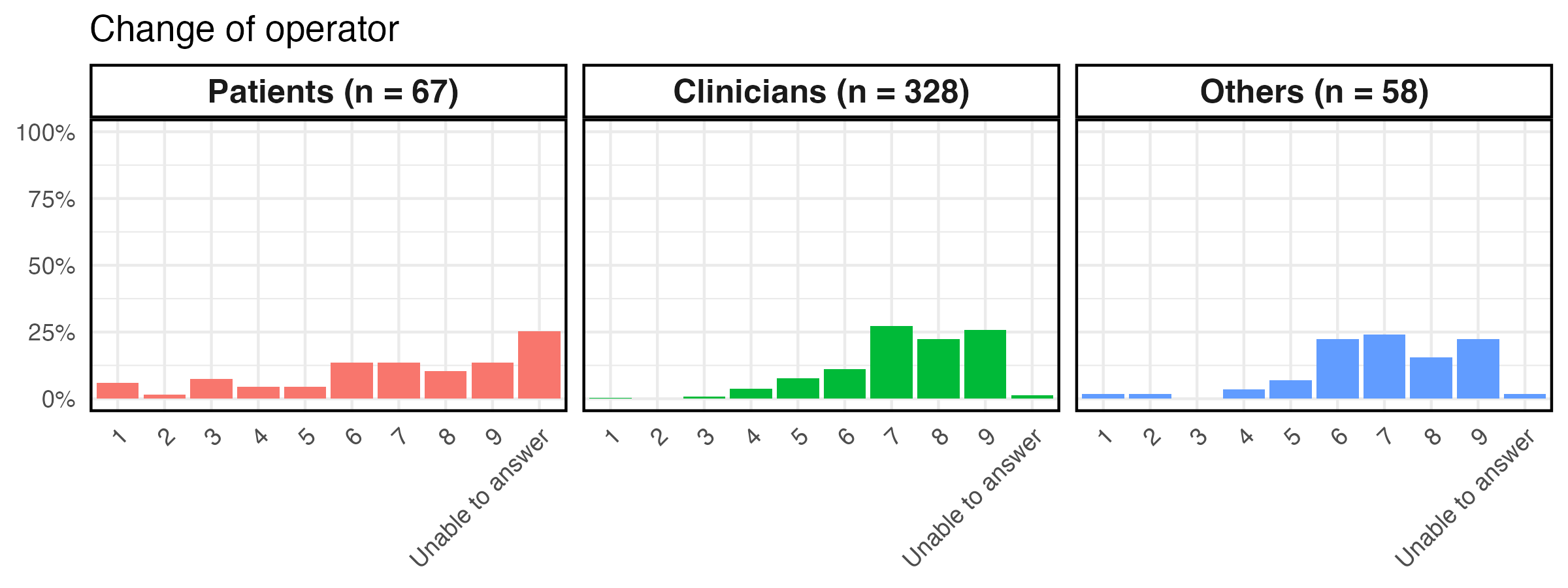

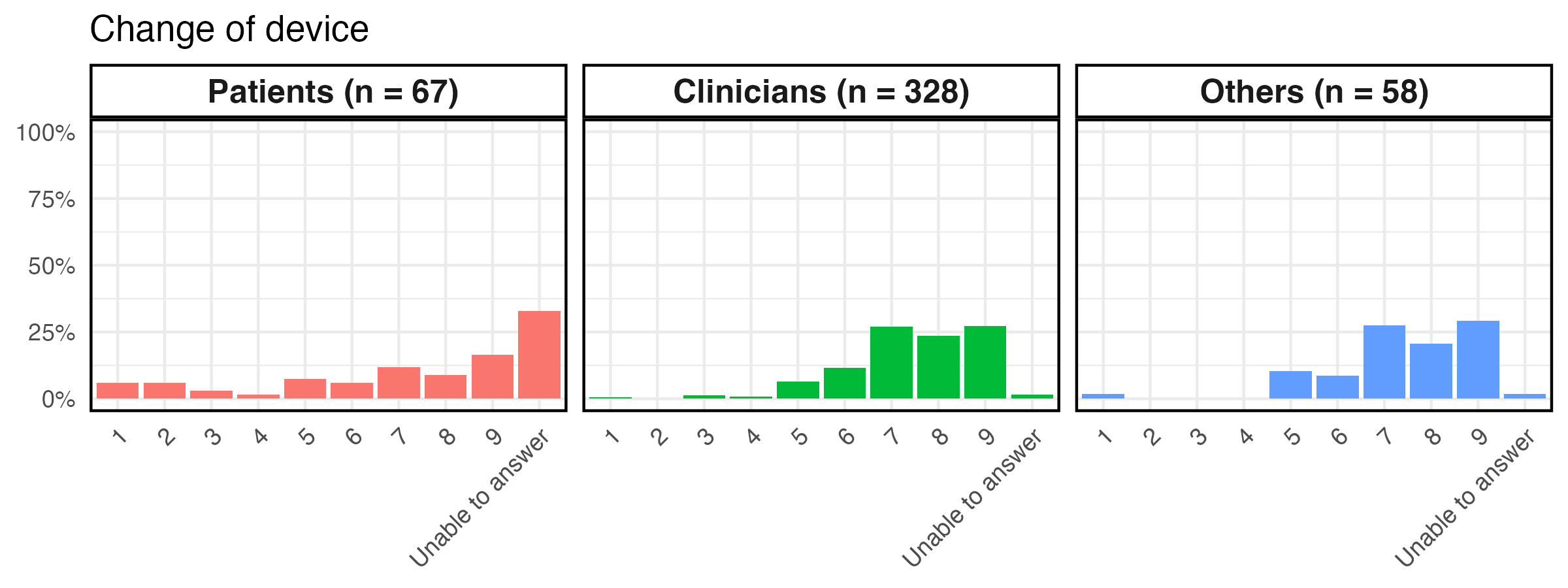

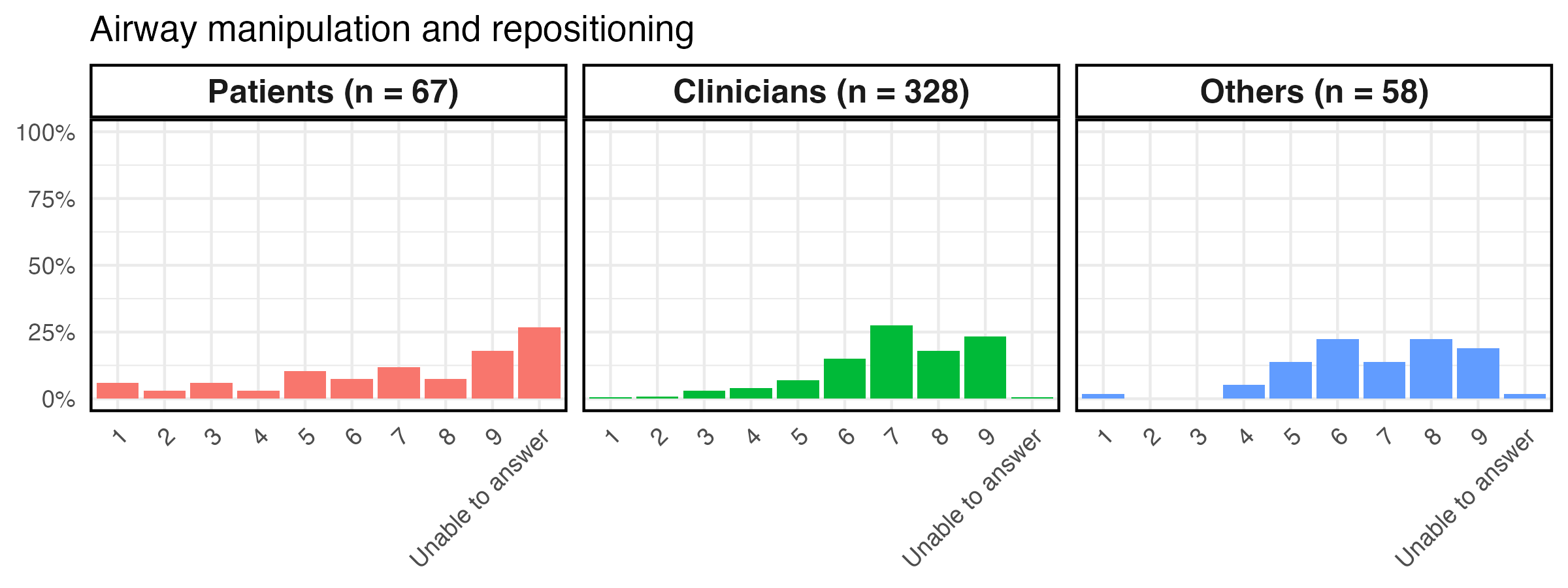

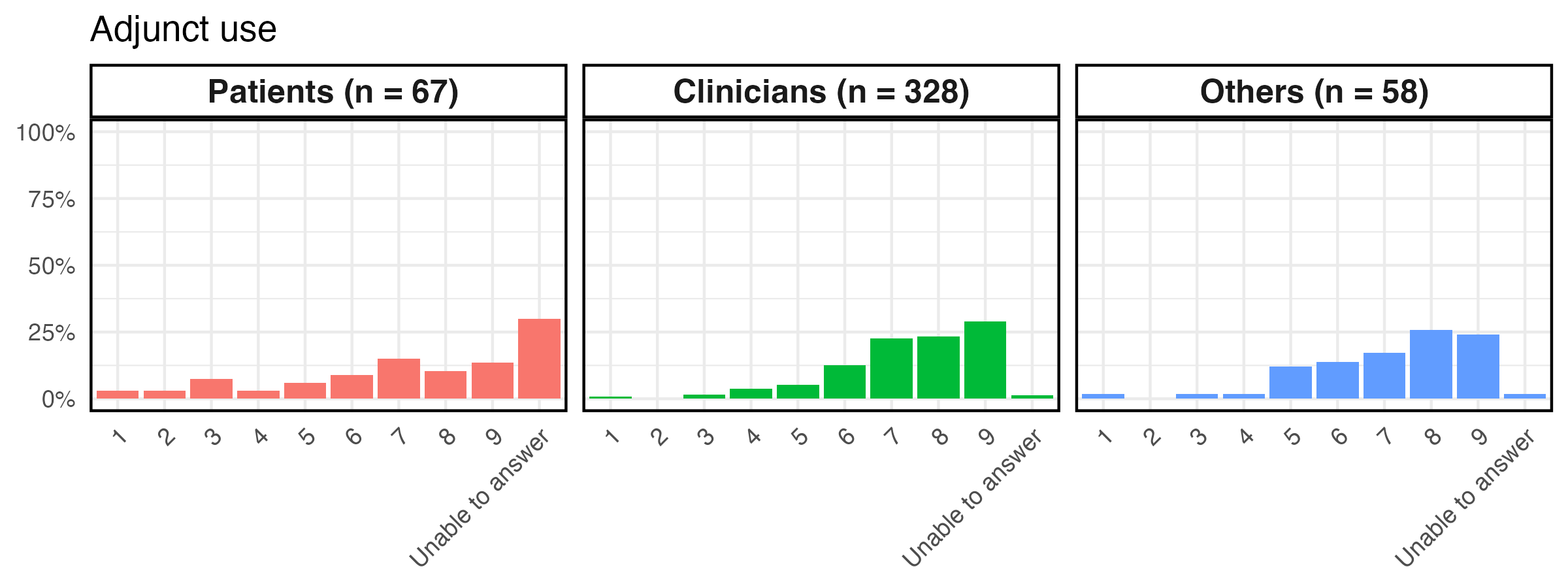

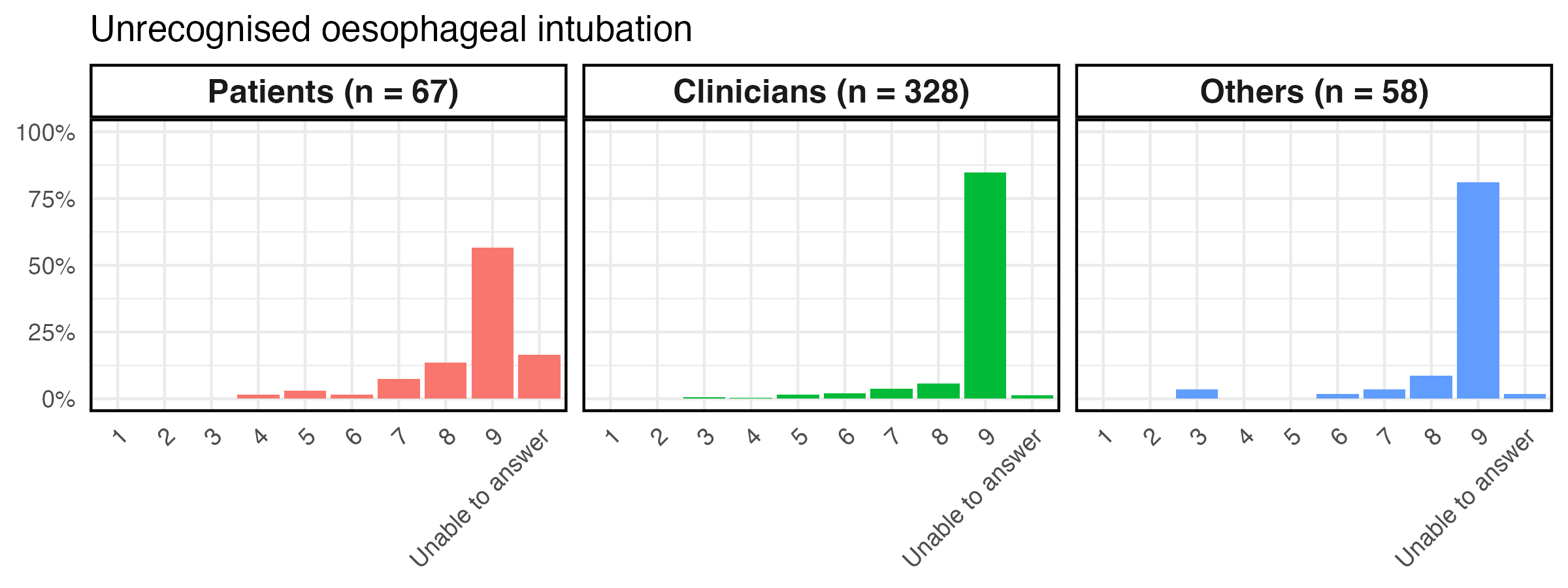

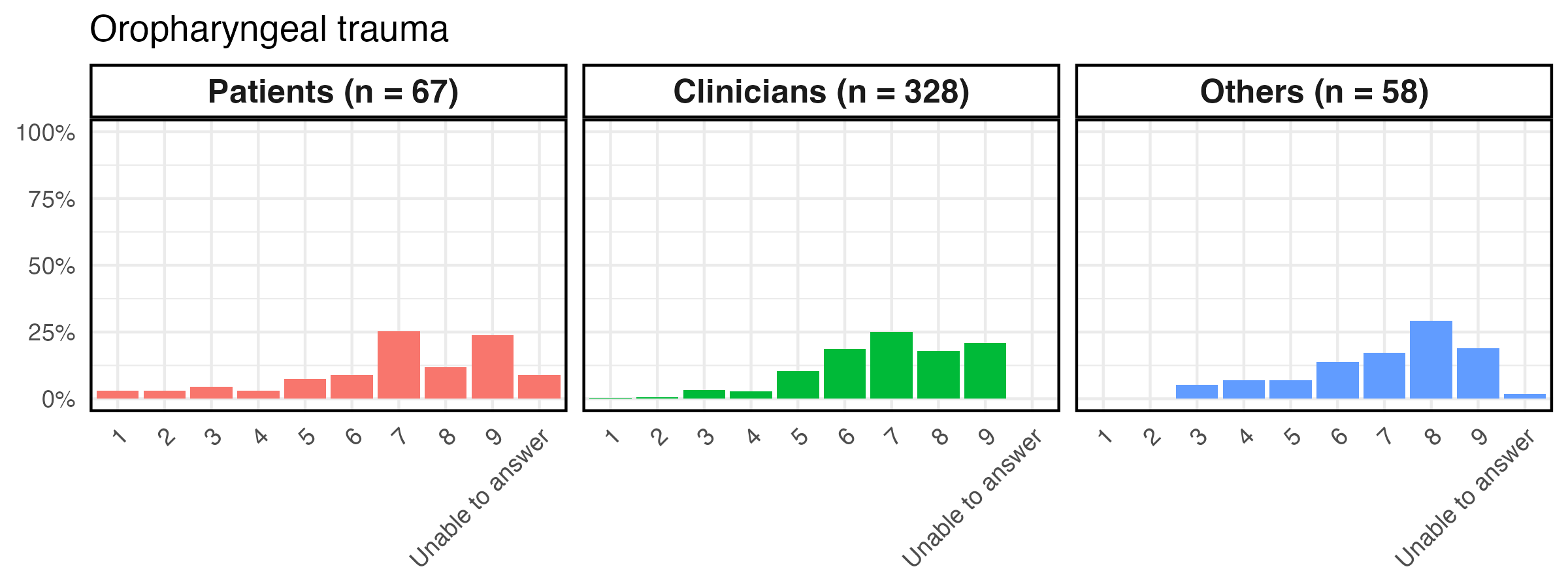

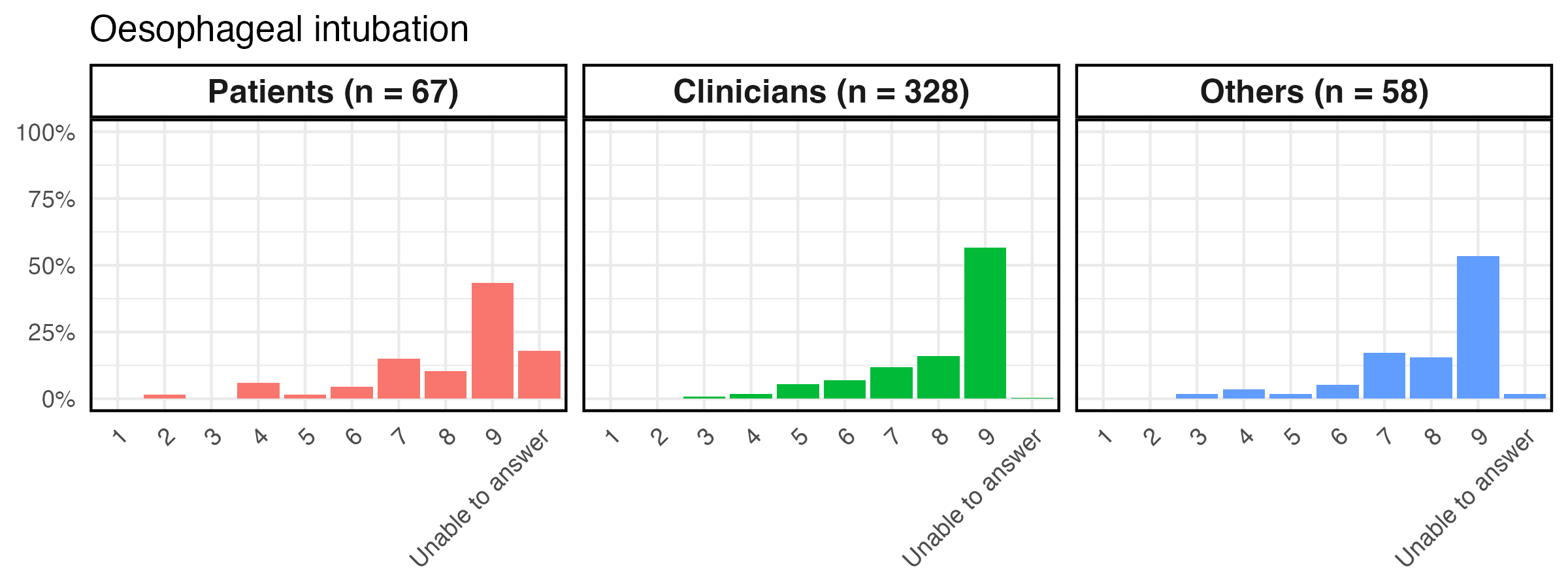

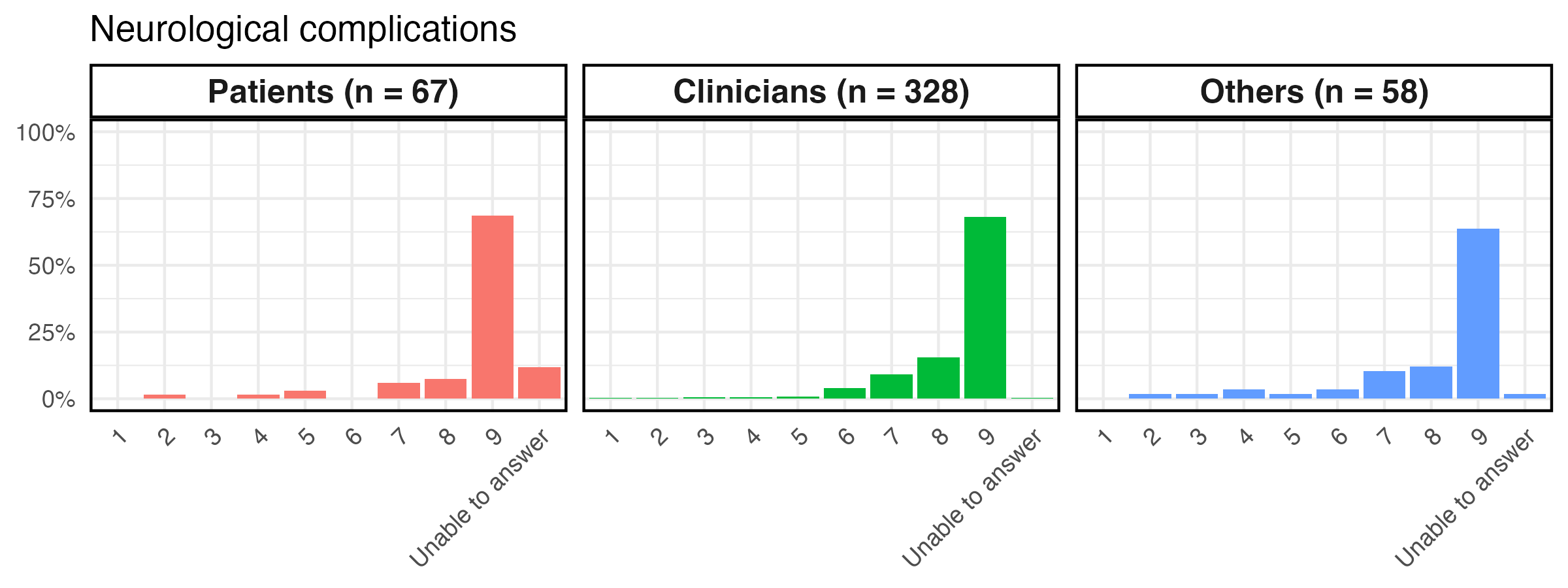

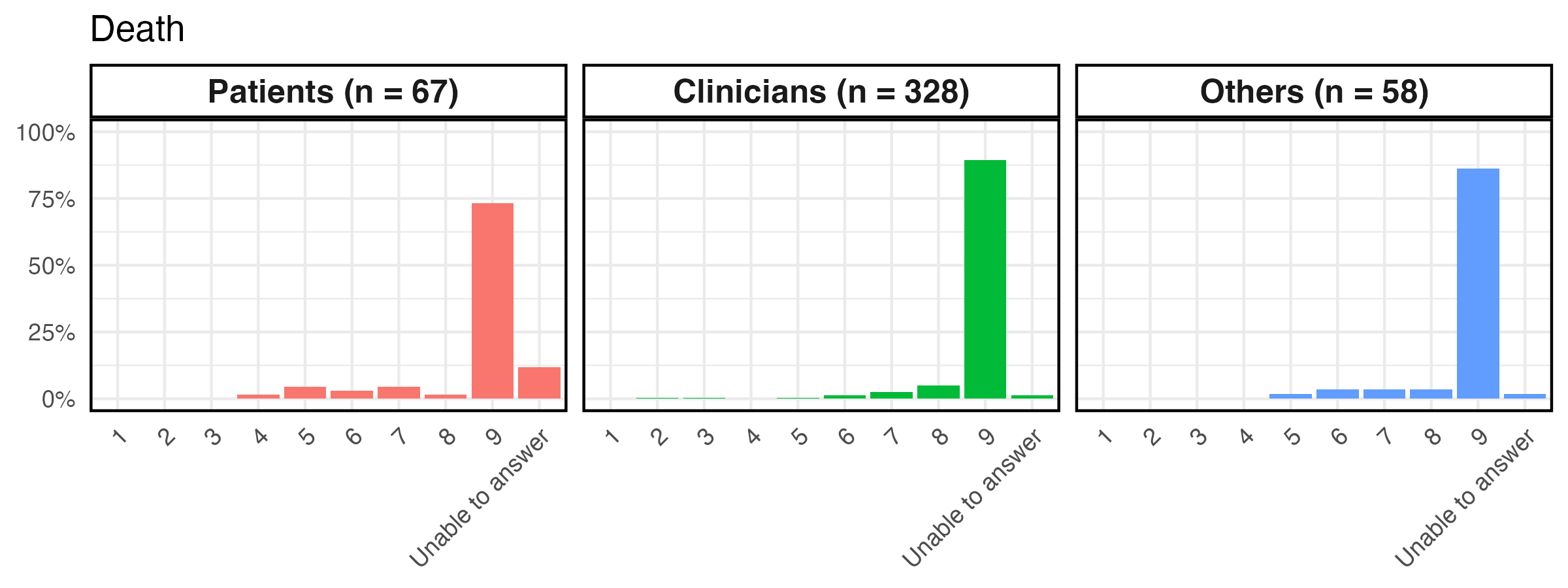

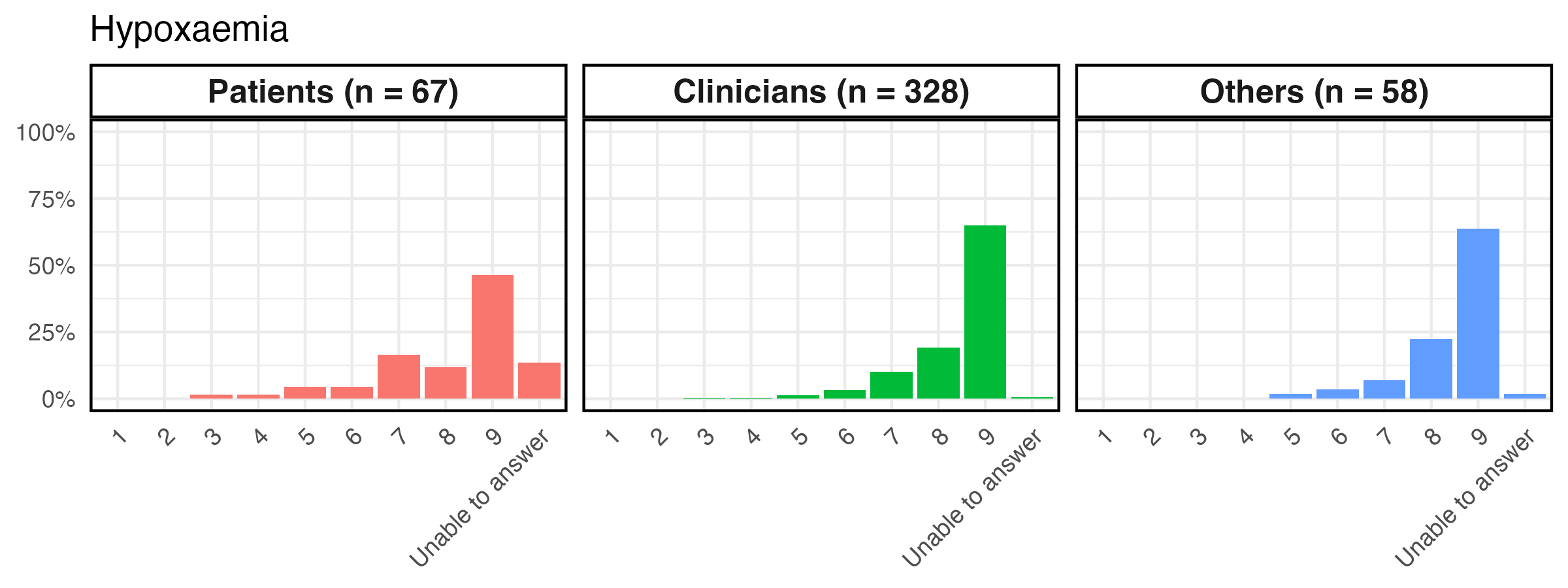

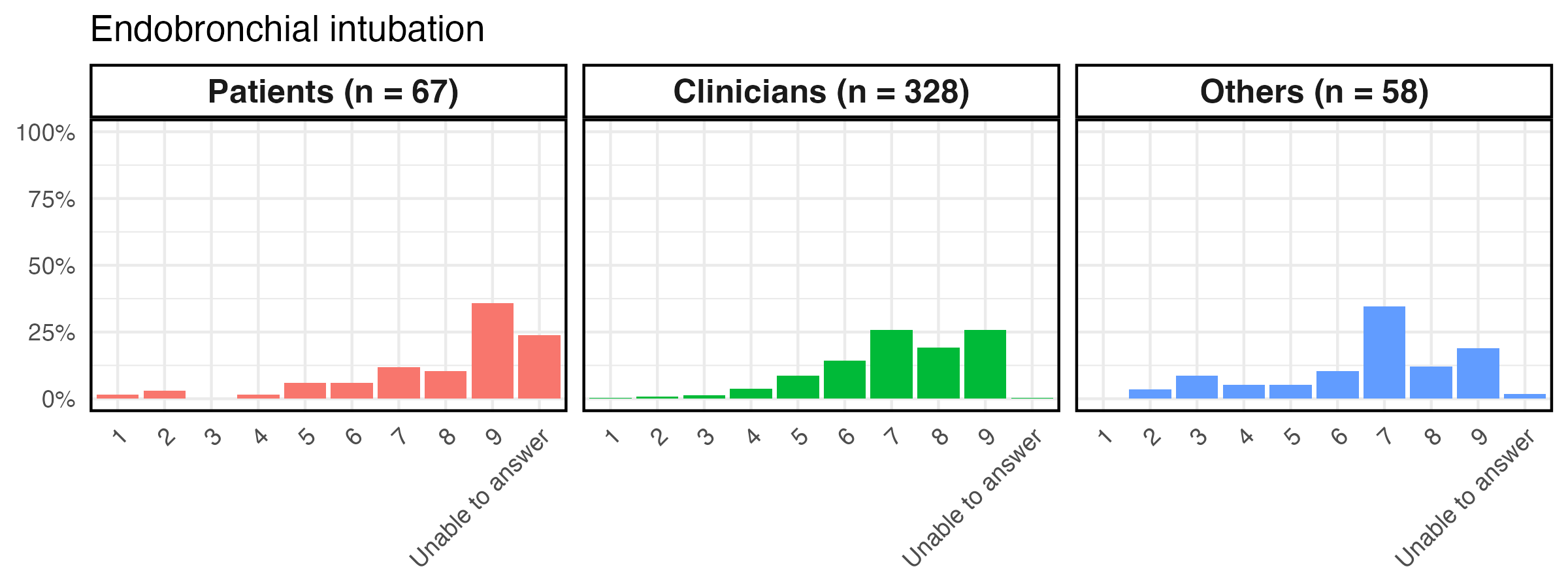

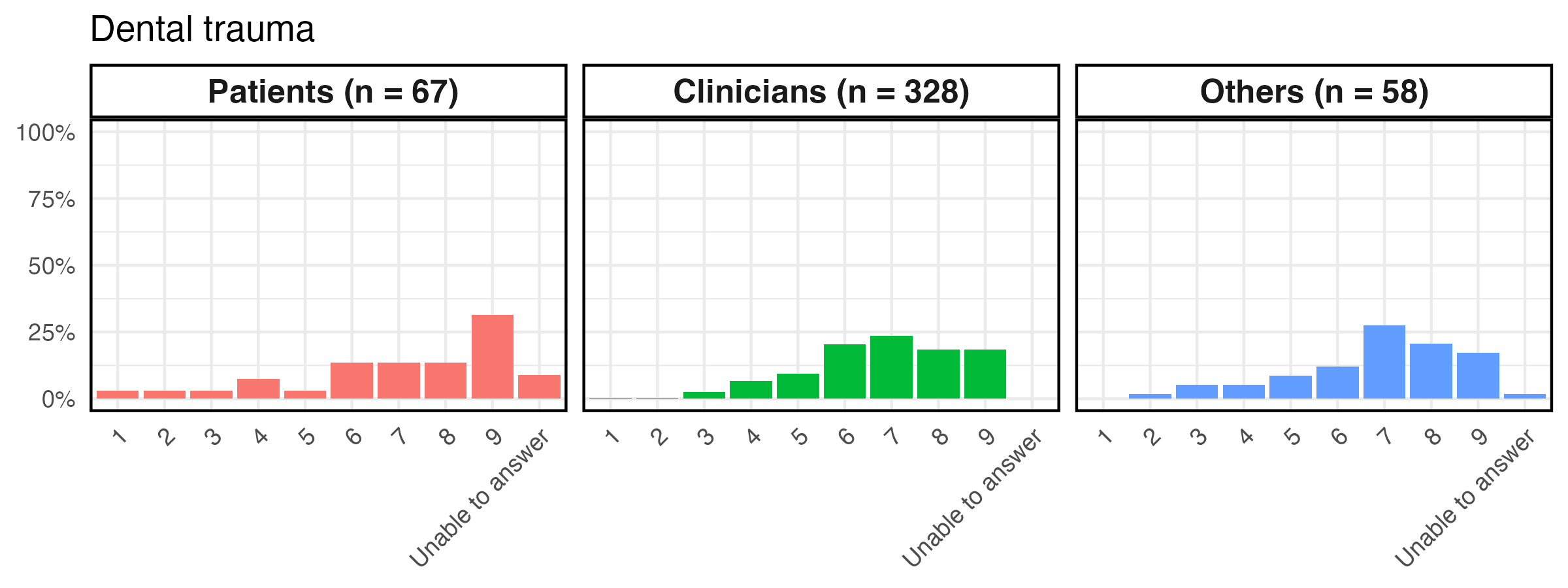

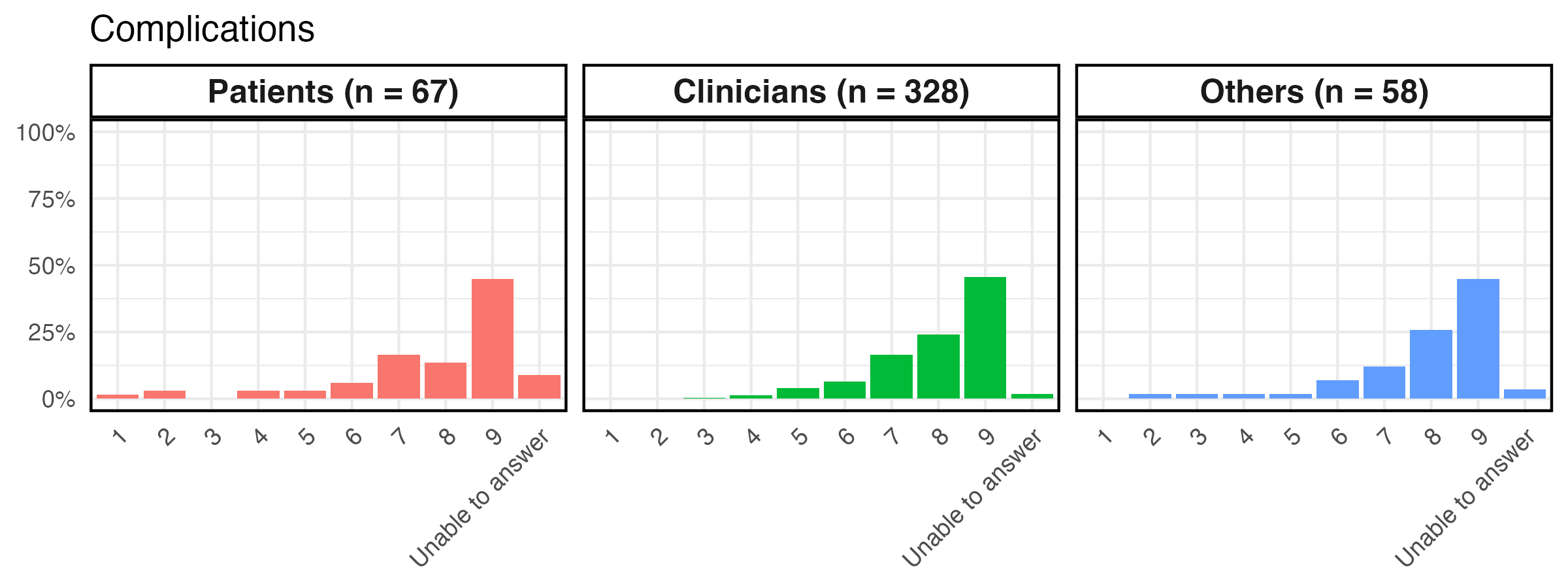

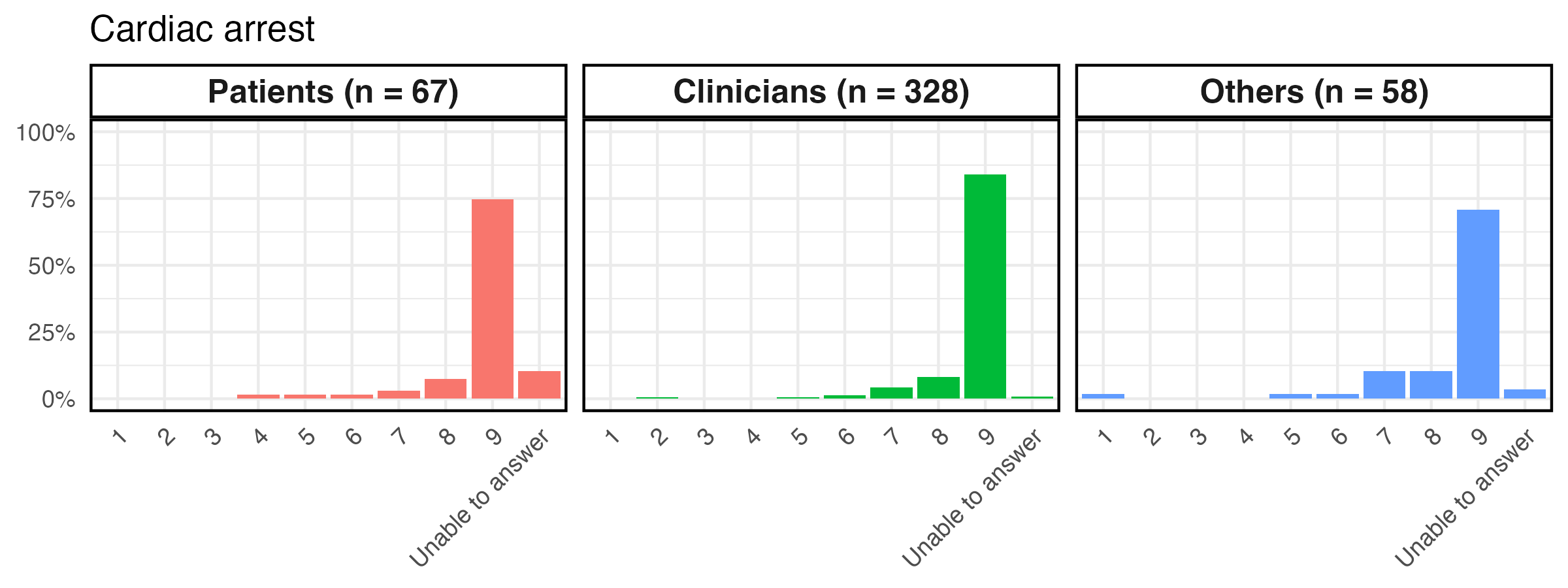

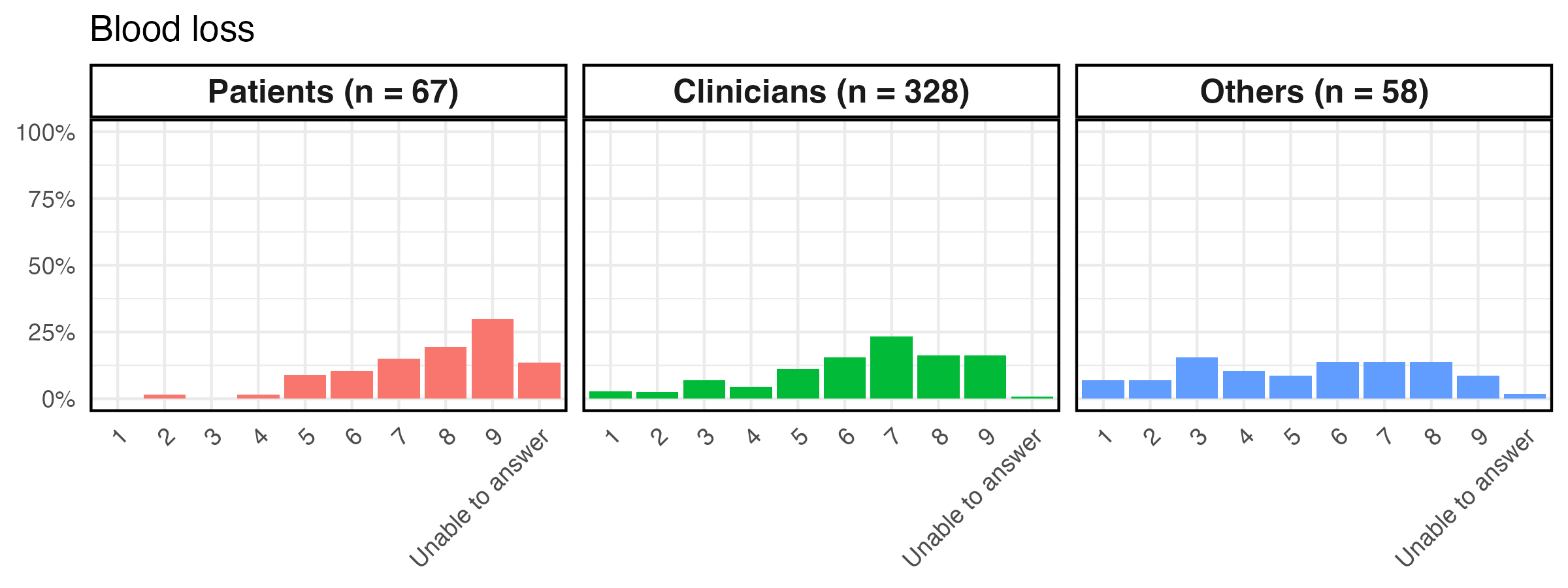

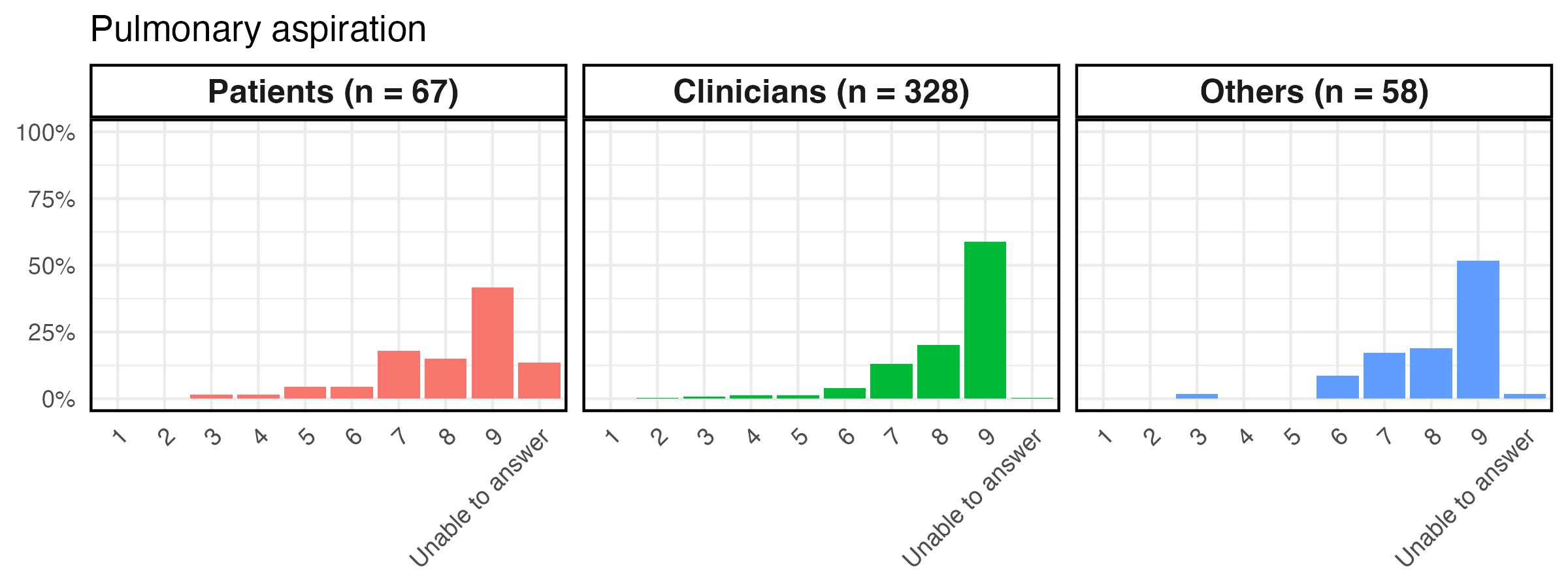

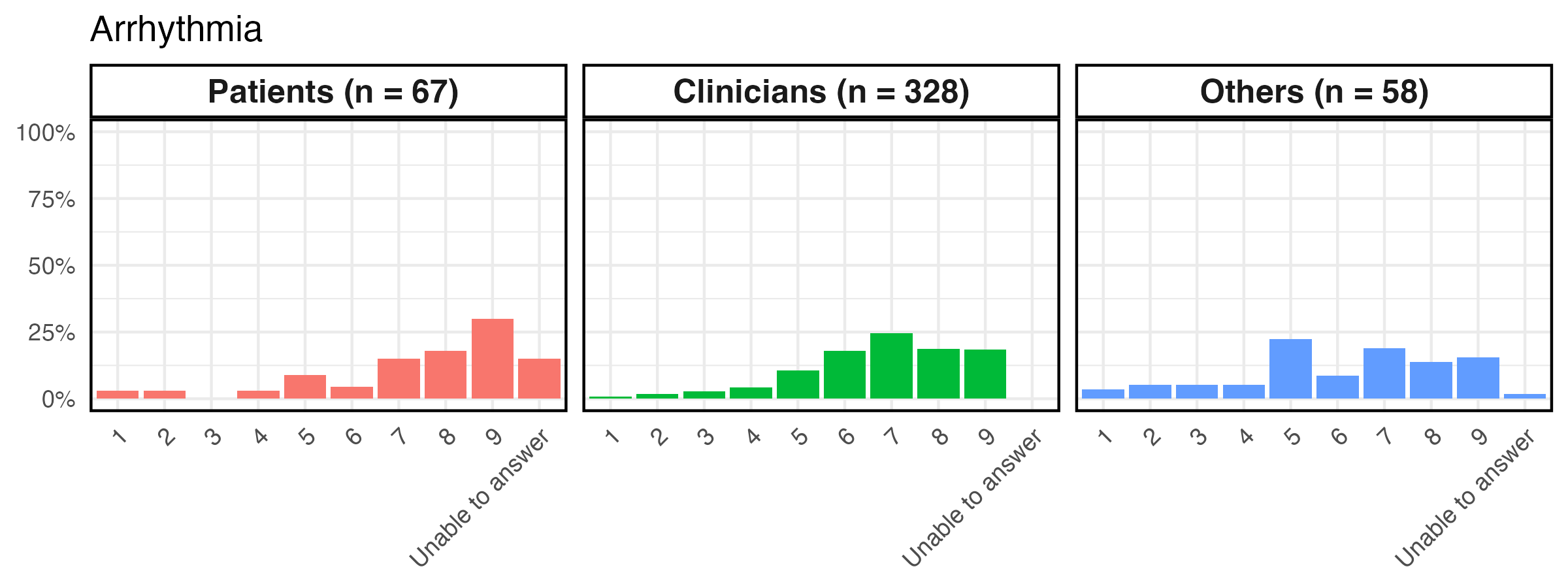

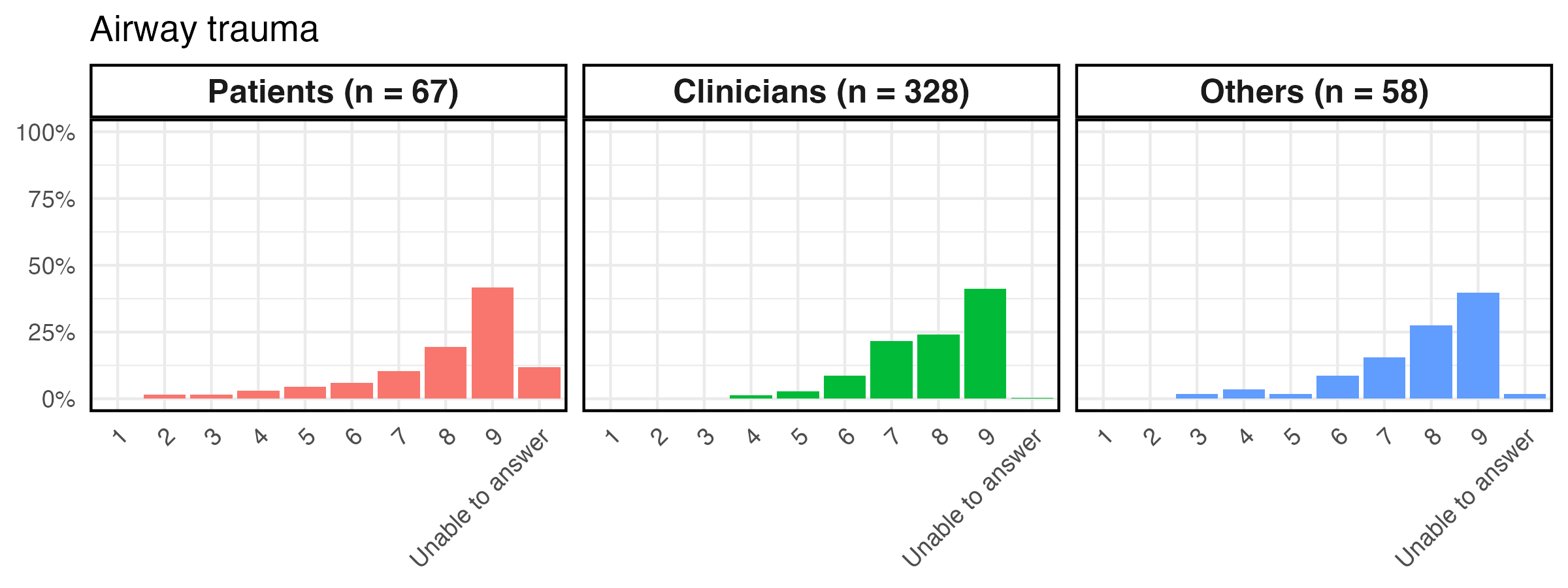

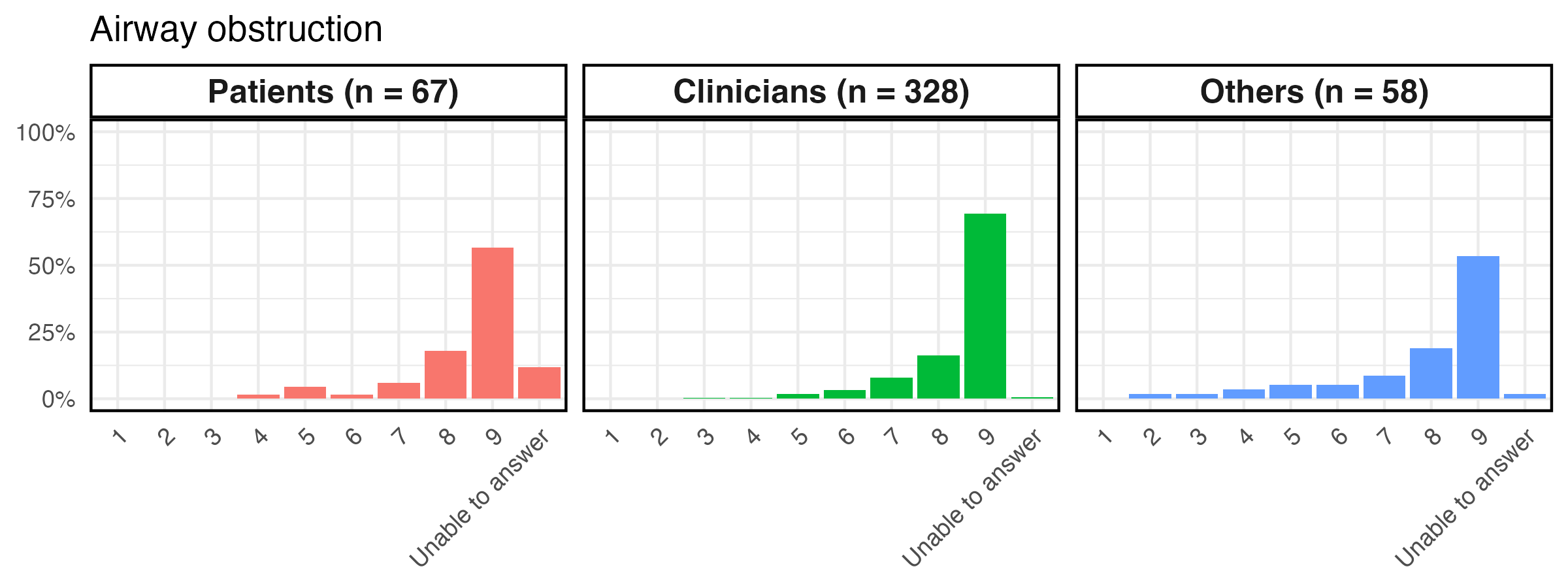


Figure S3 Individual outcome voting results grouped by stakeholder group (Round 2)


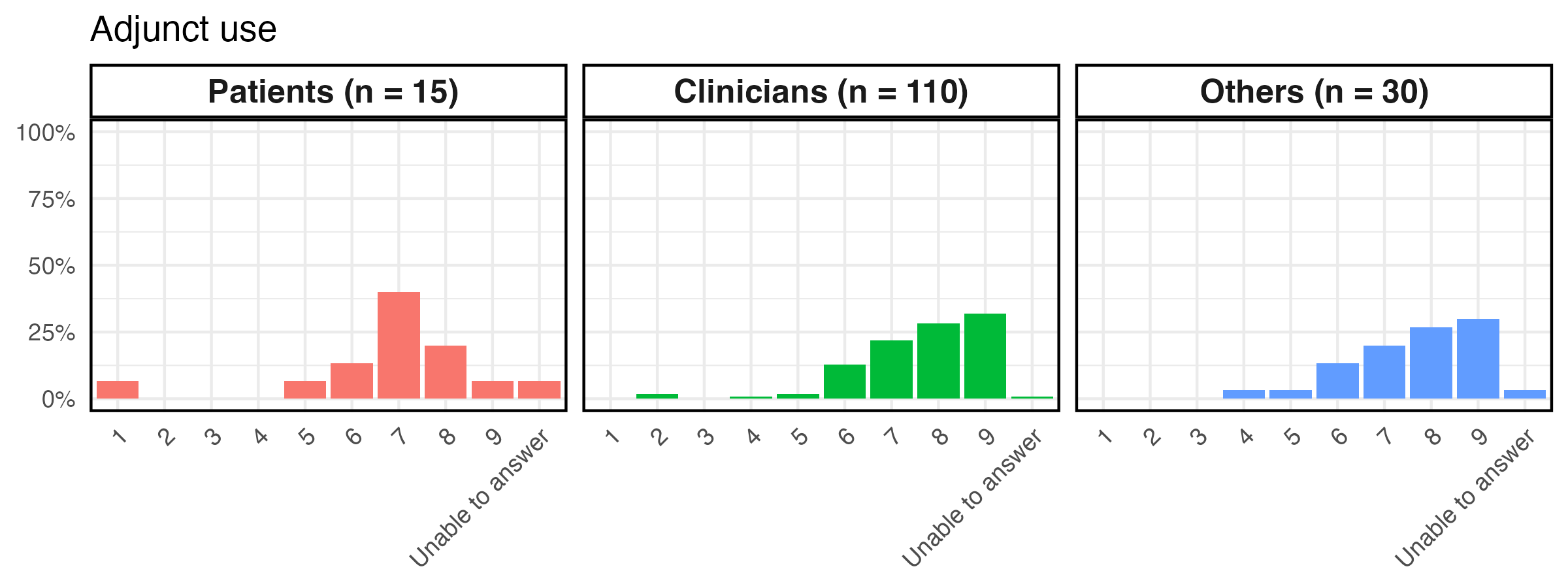

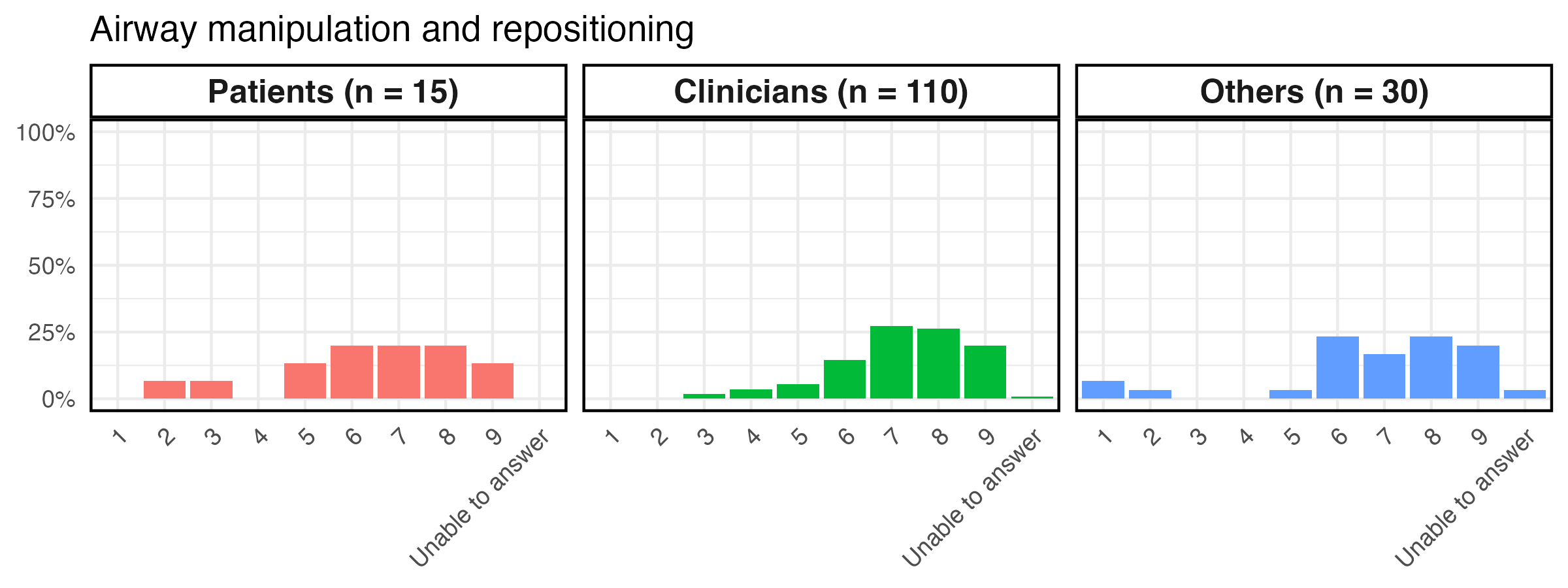

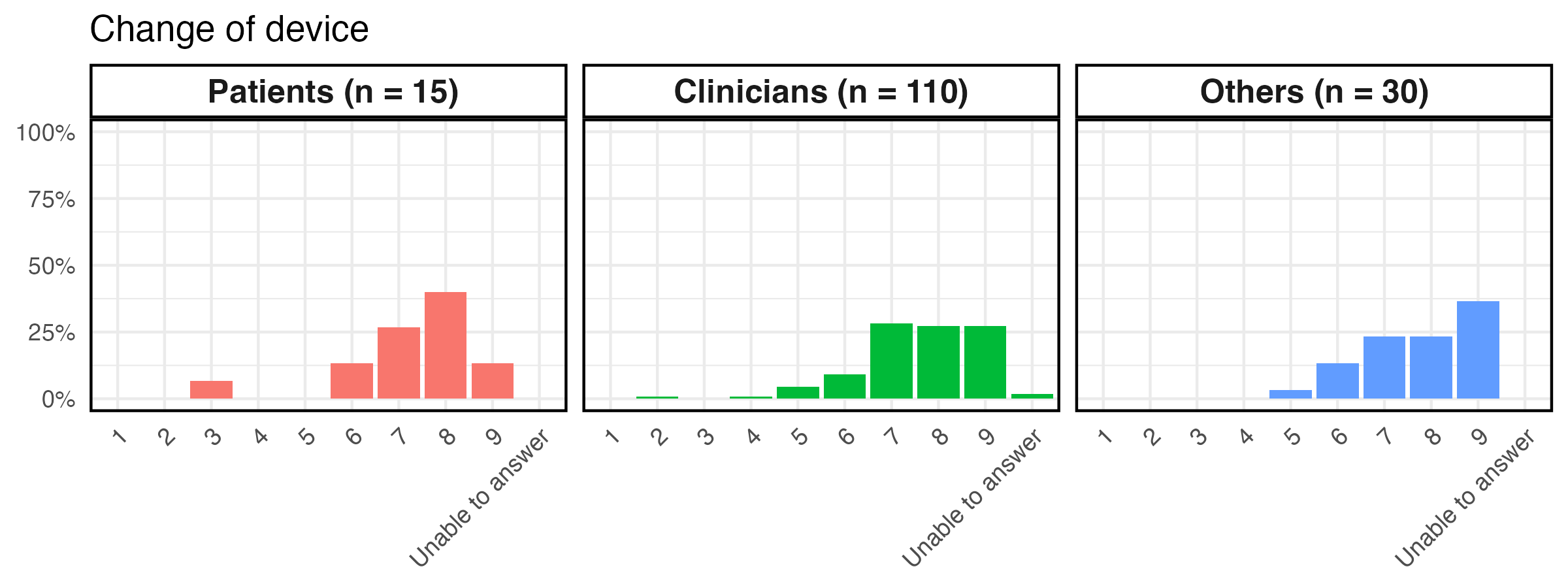

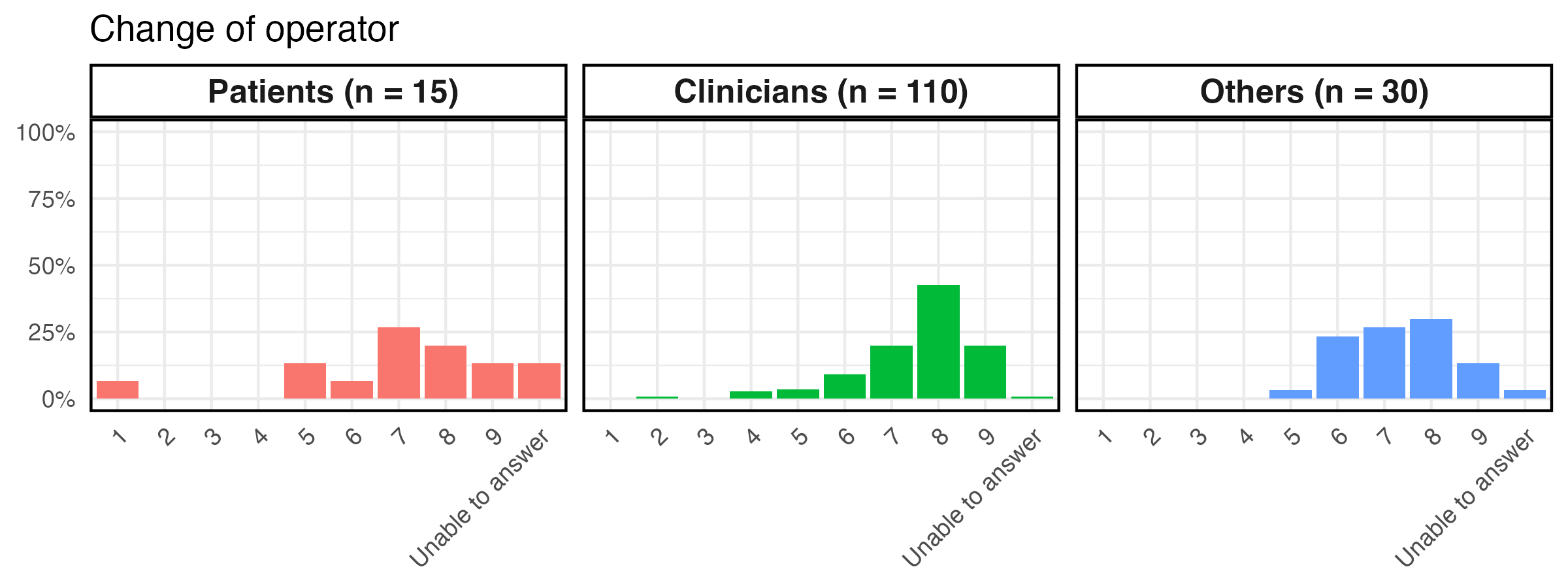

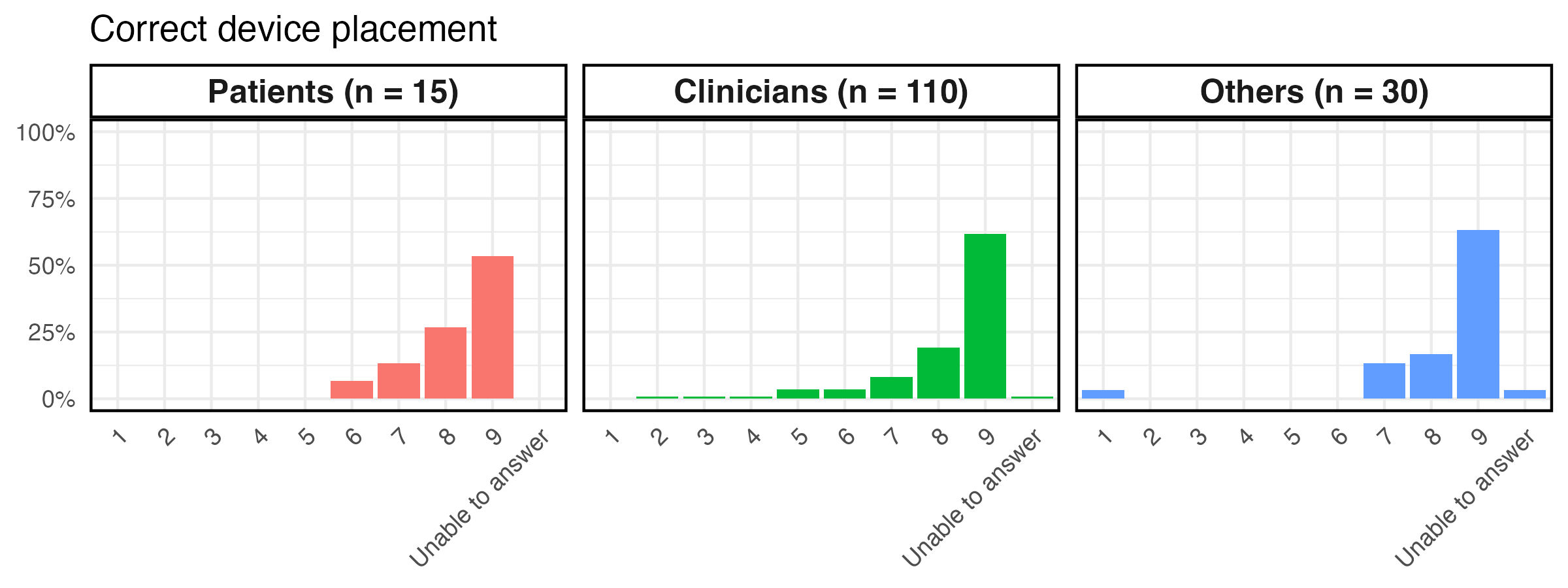

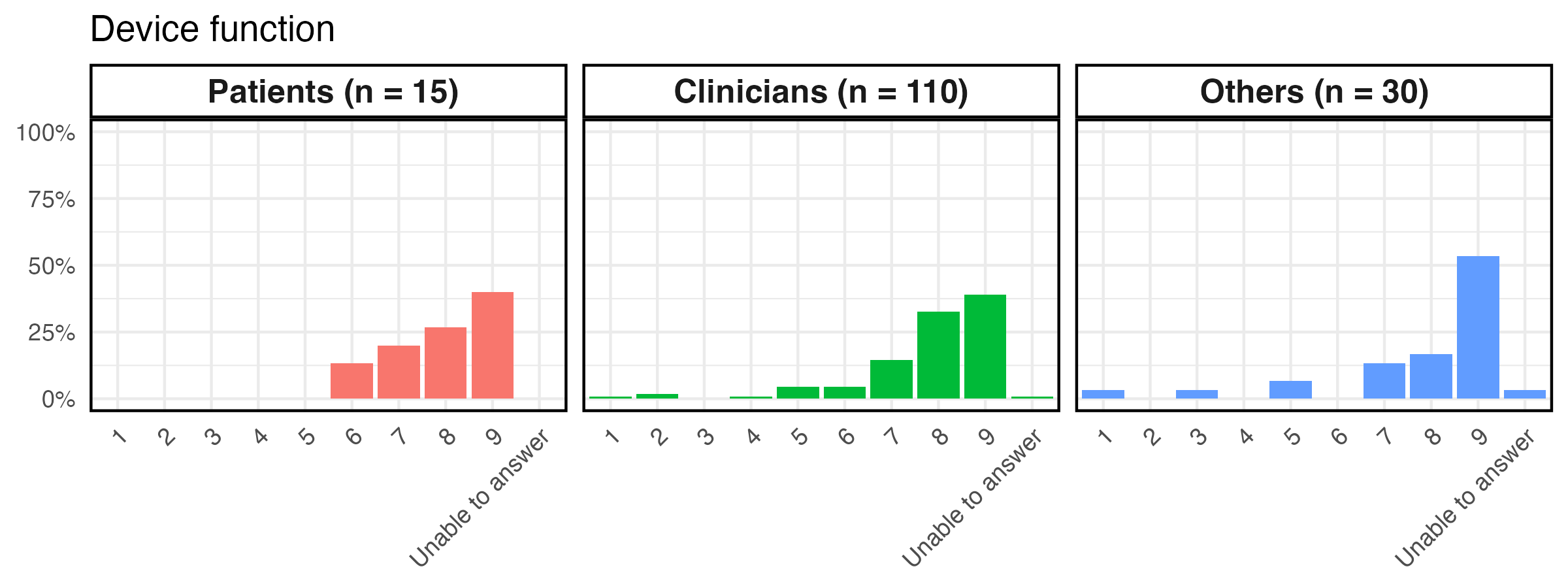

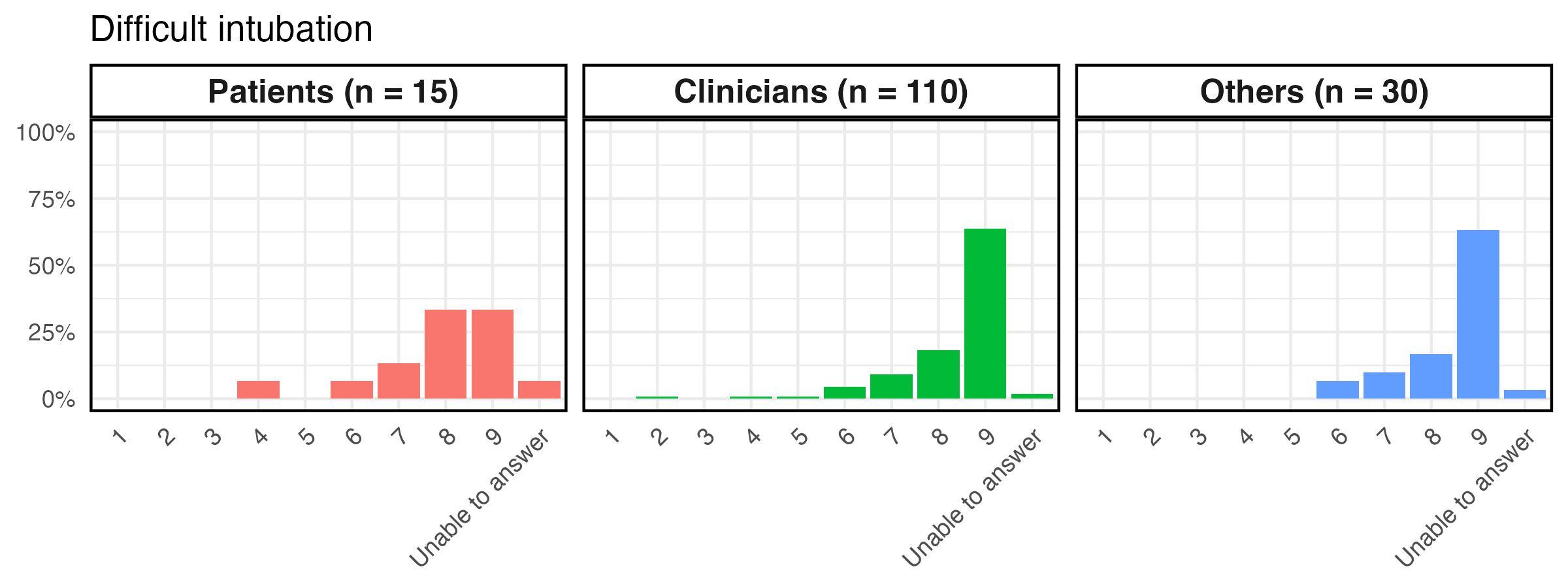

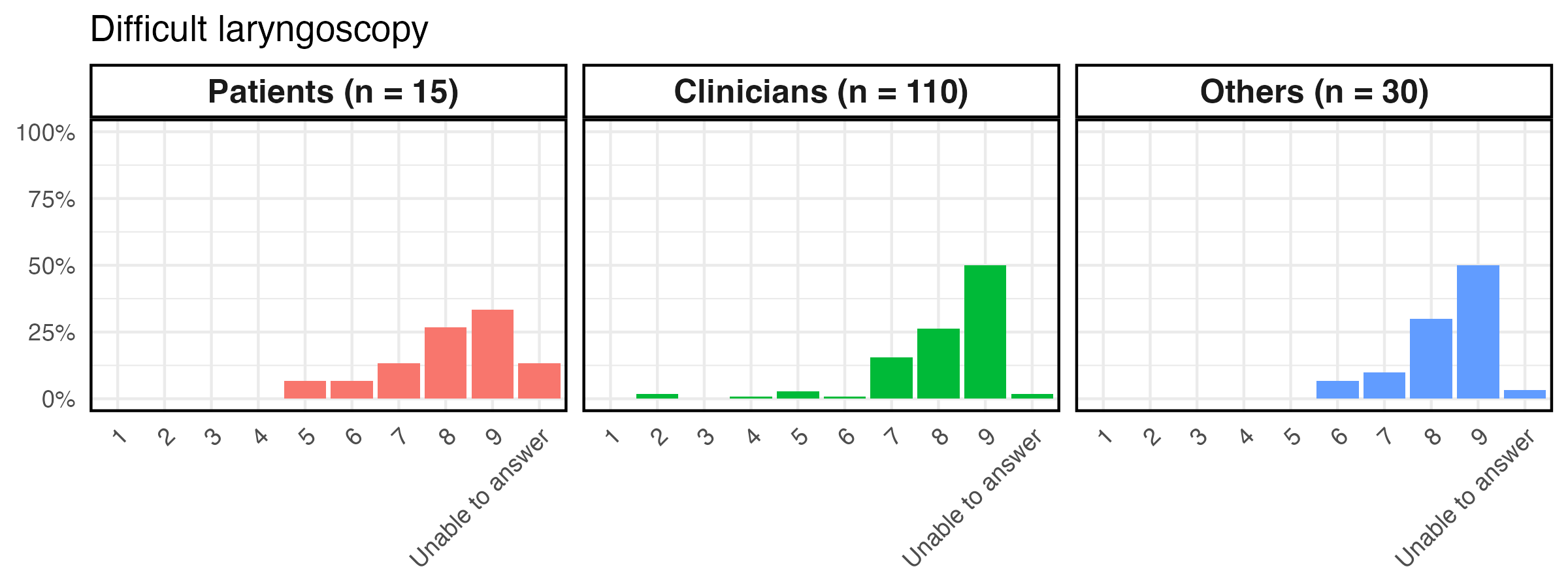

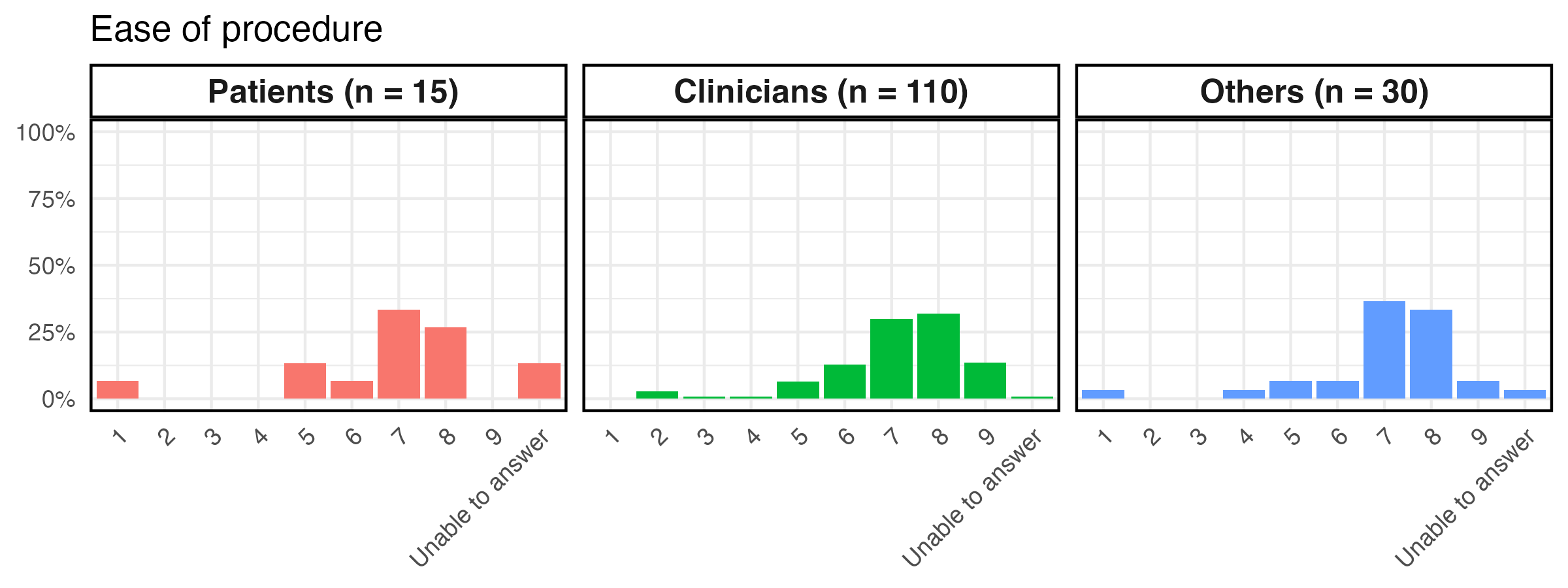

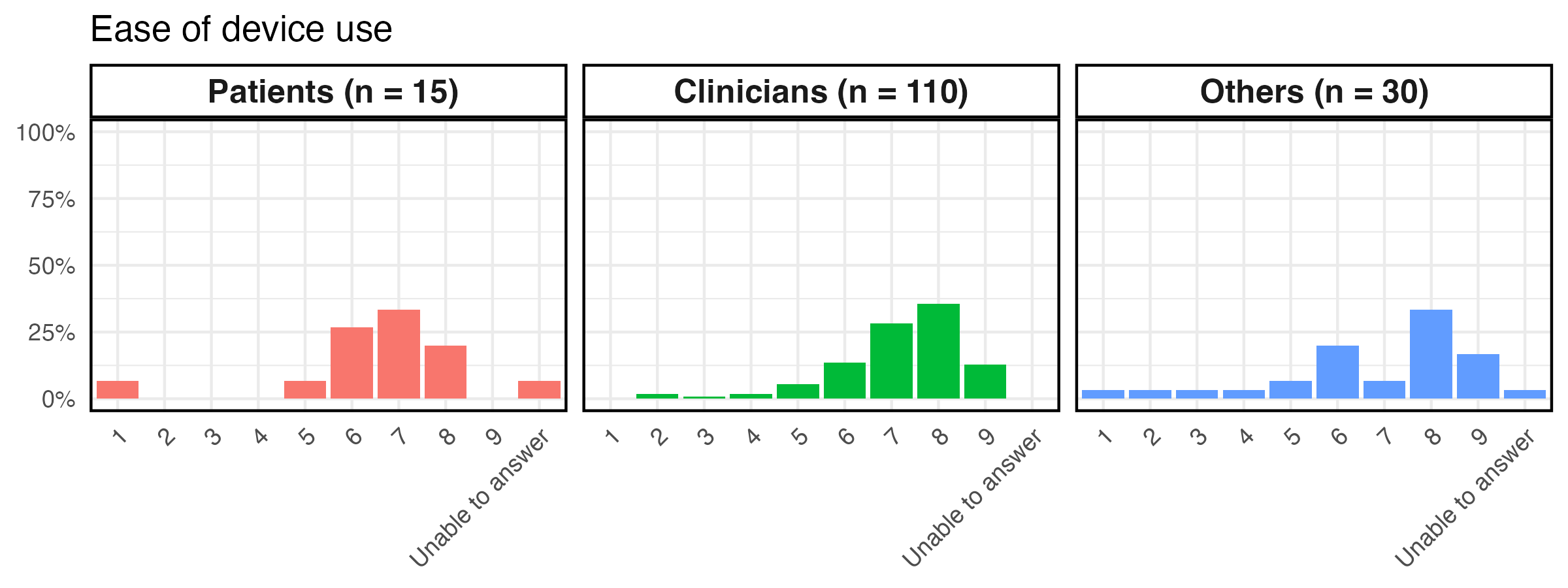

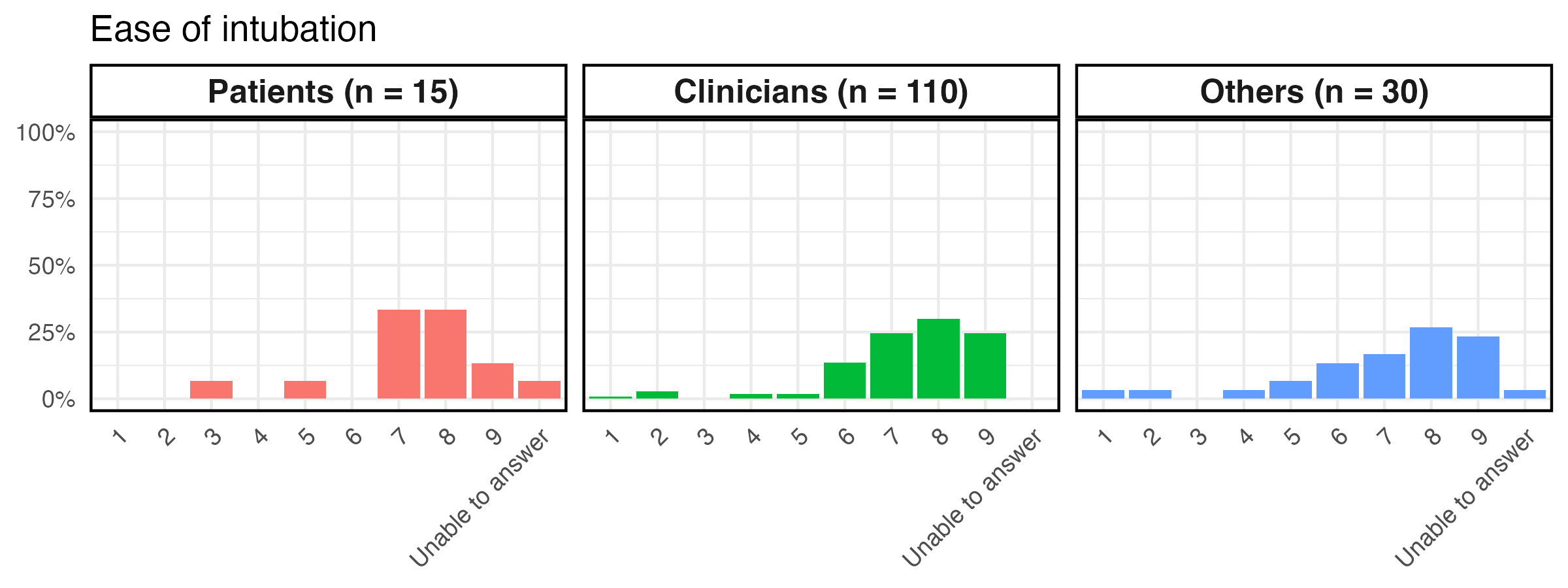

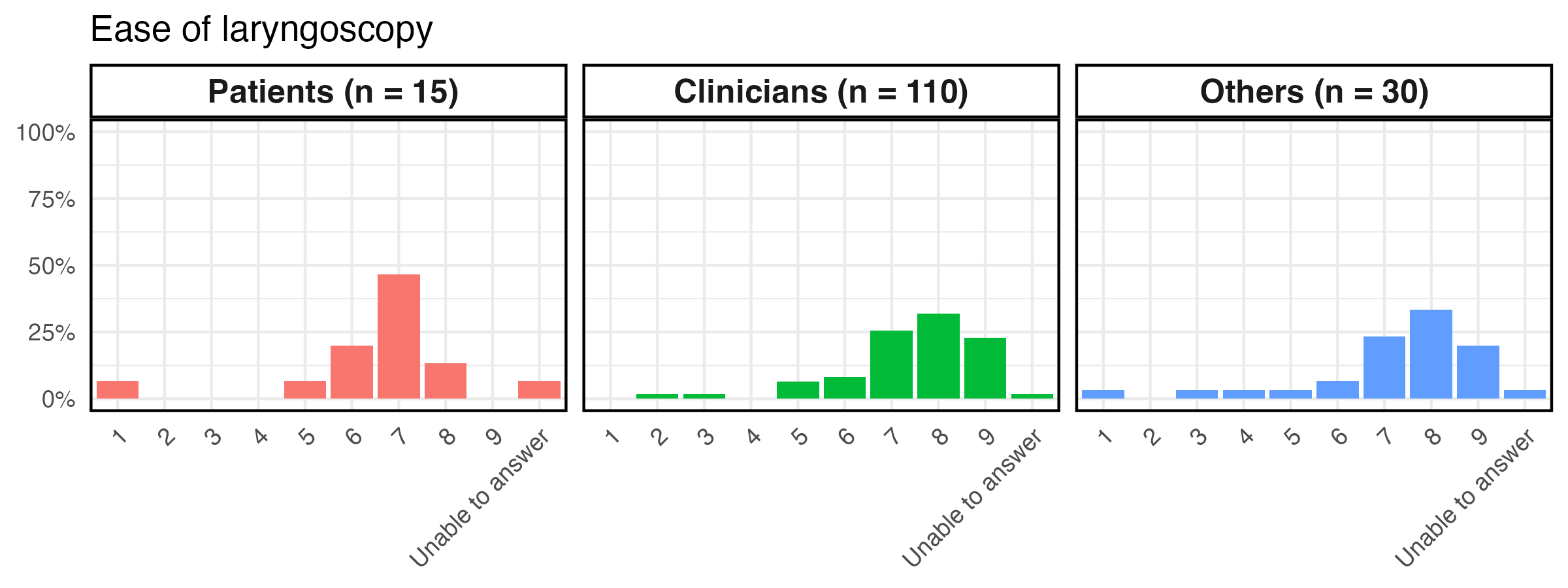

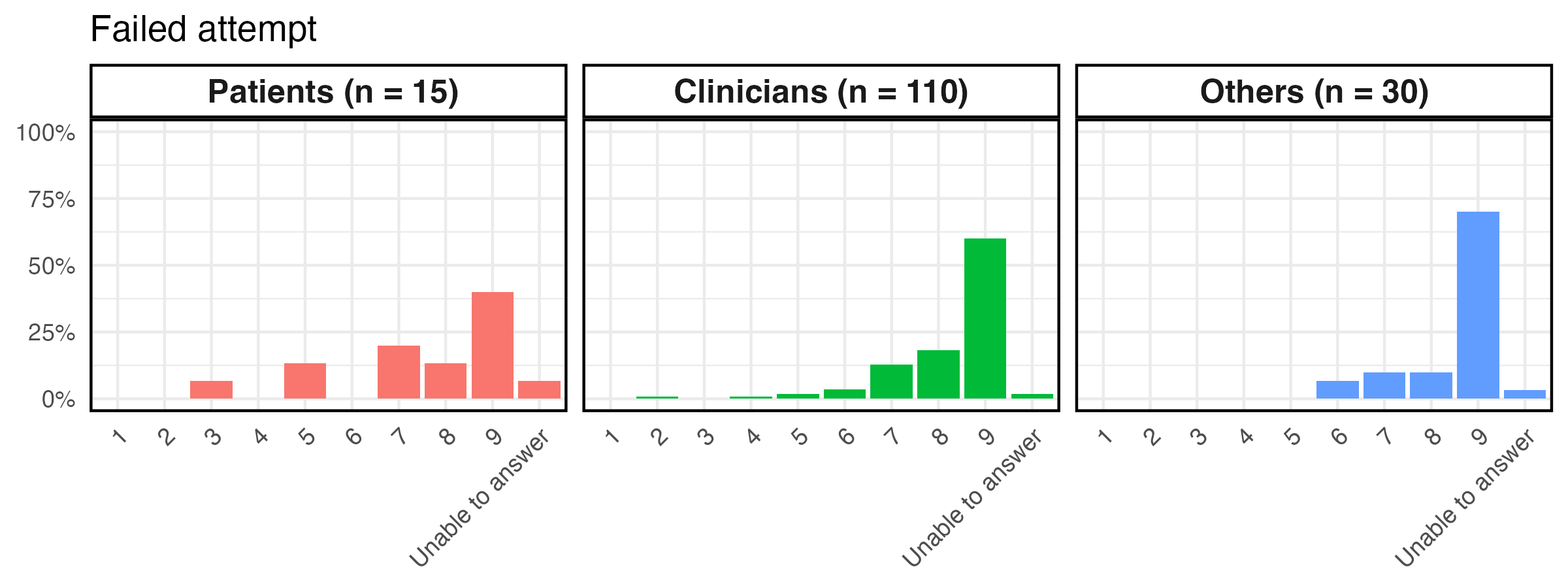

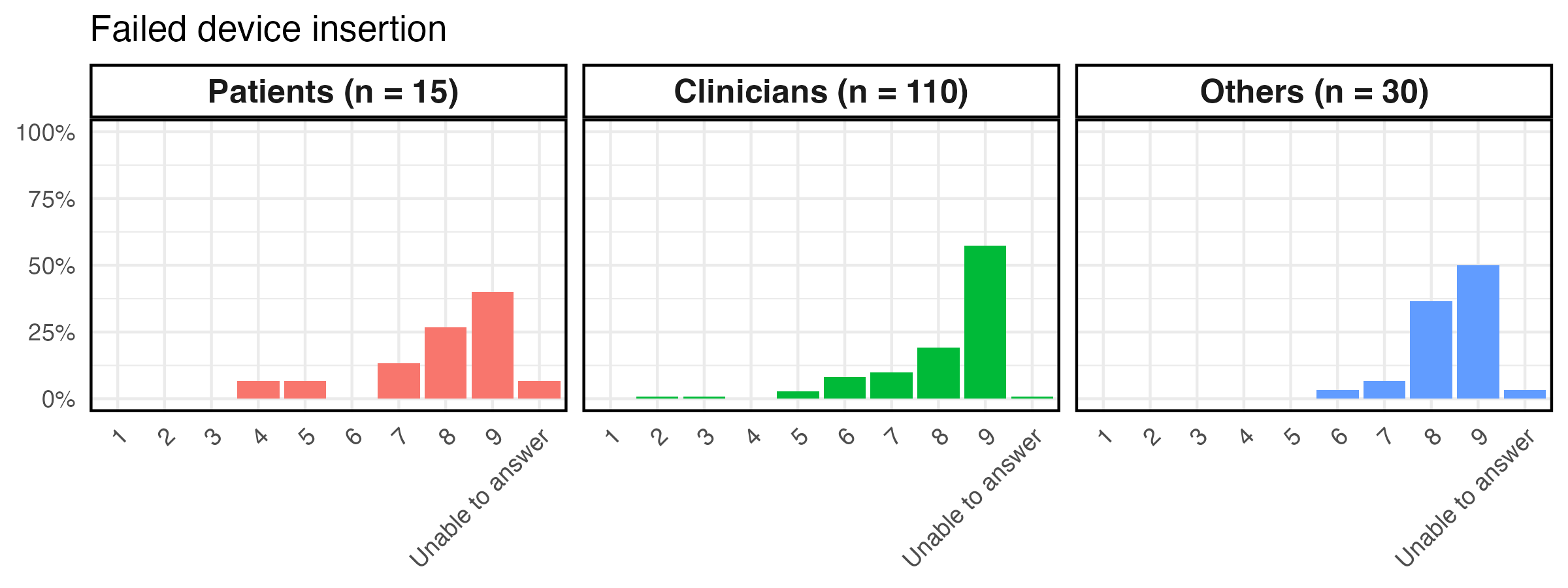

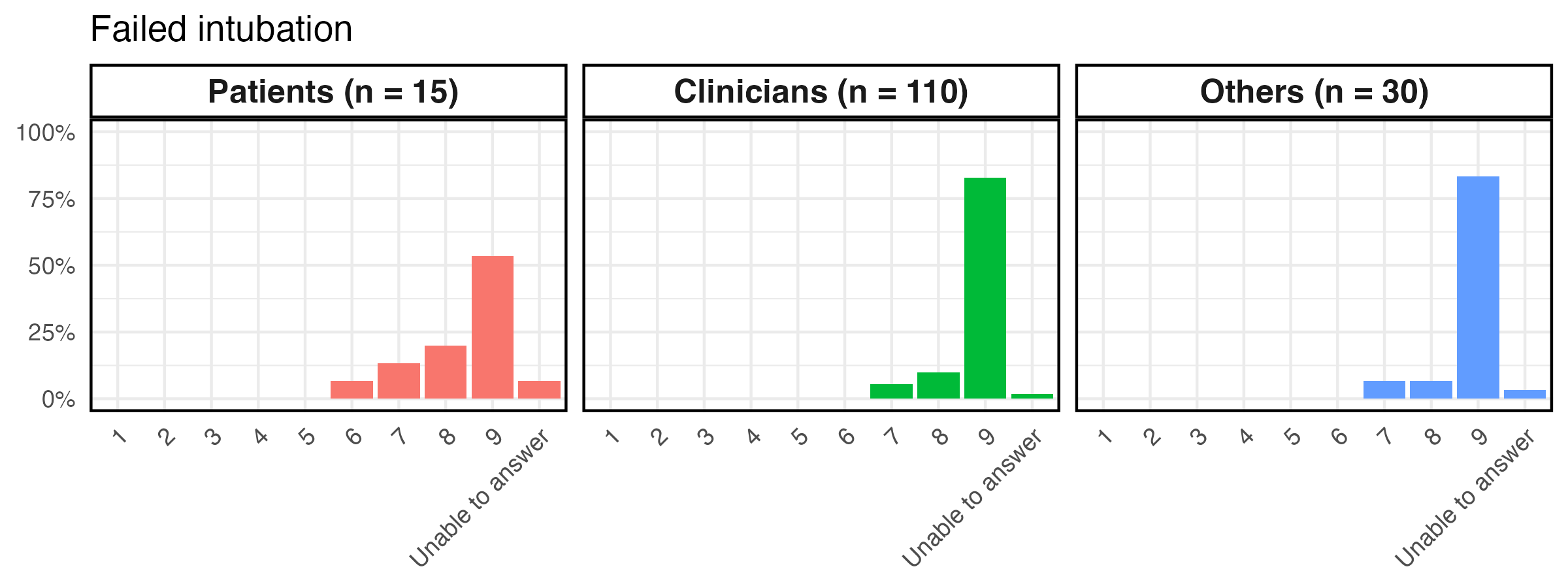

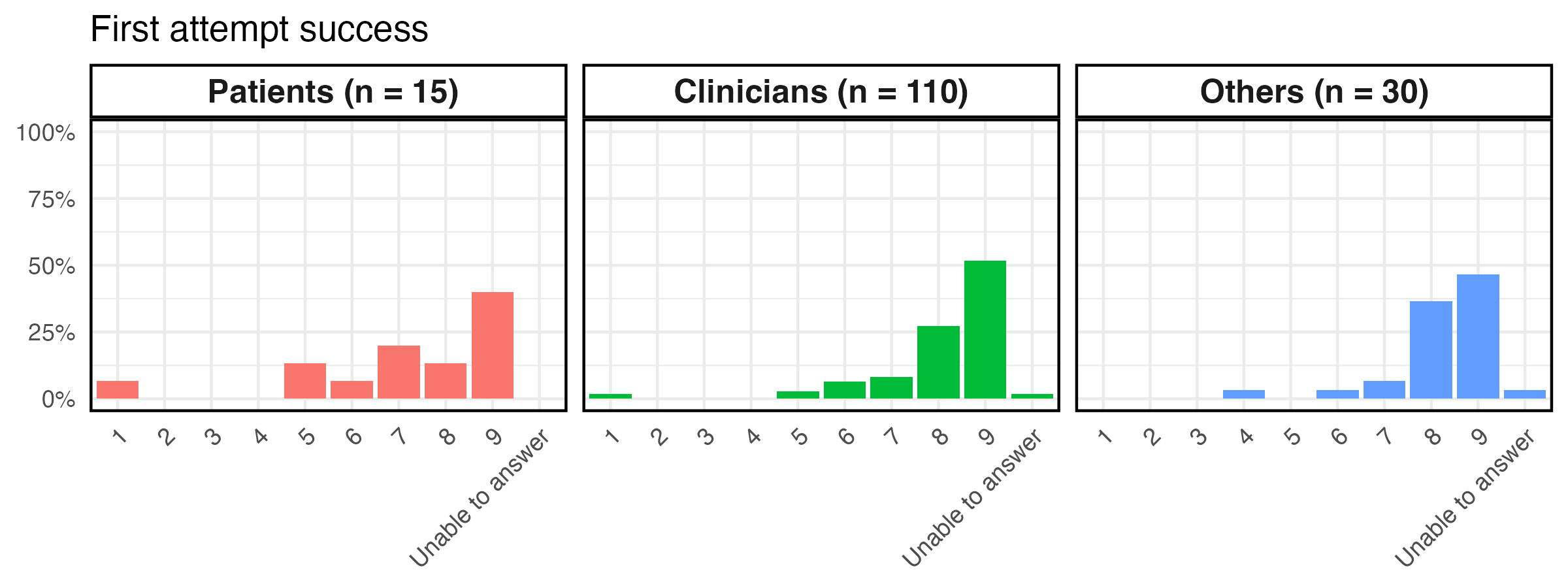

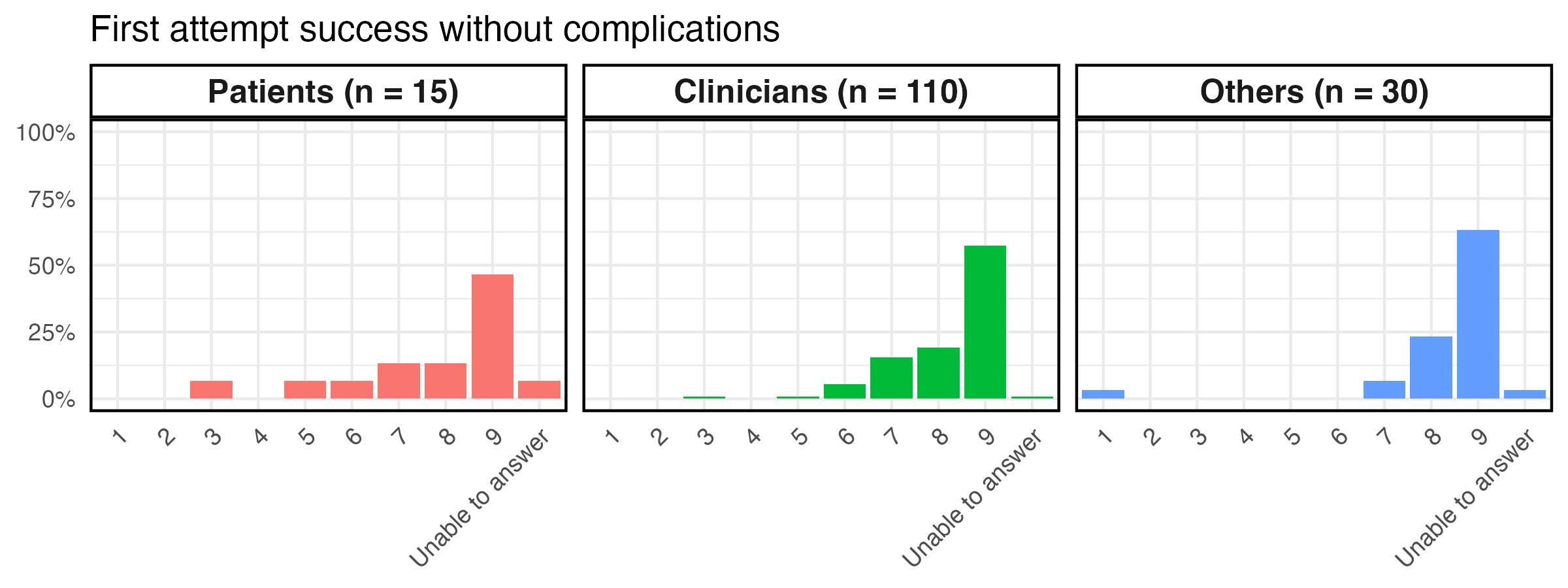

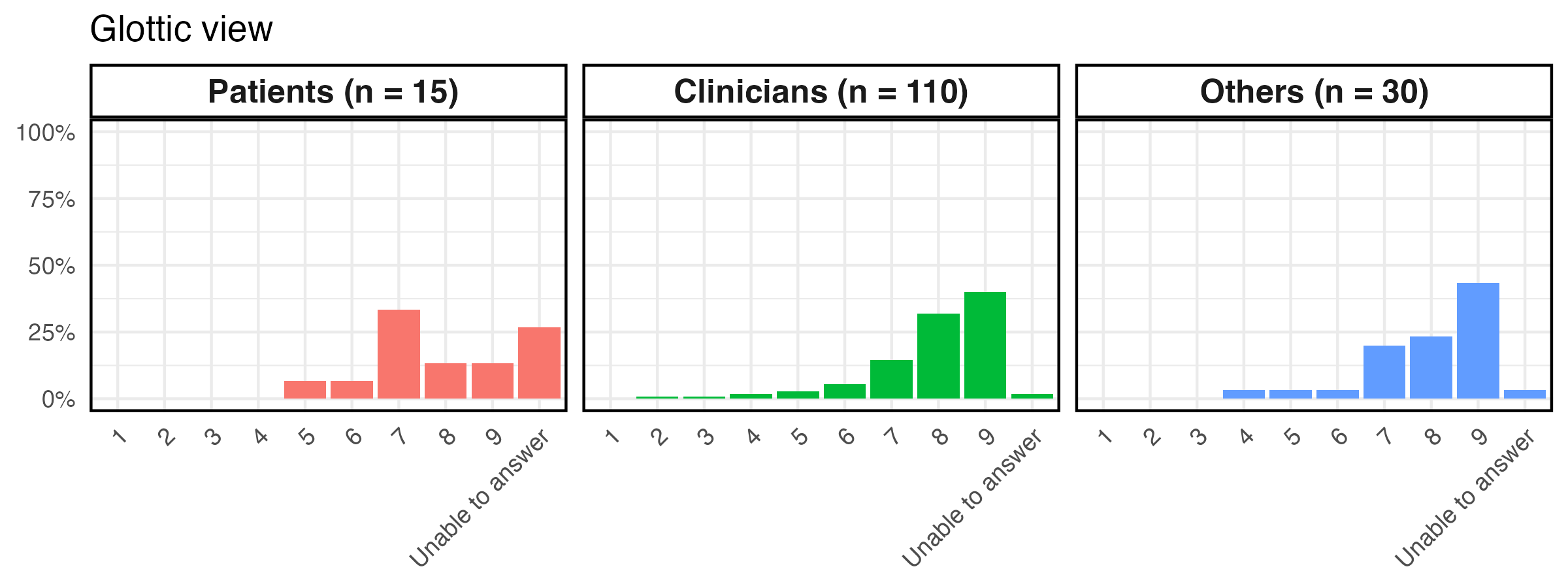

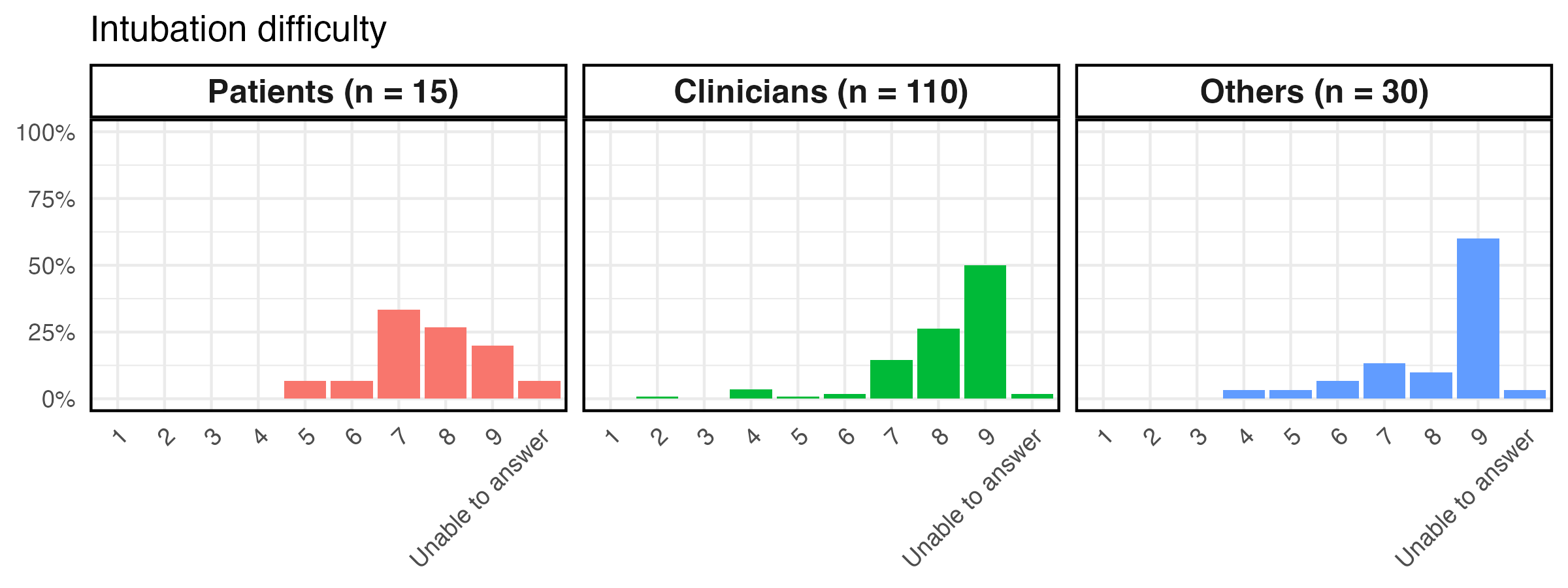

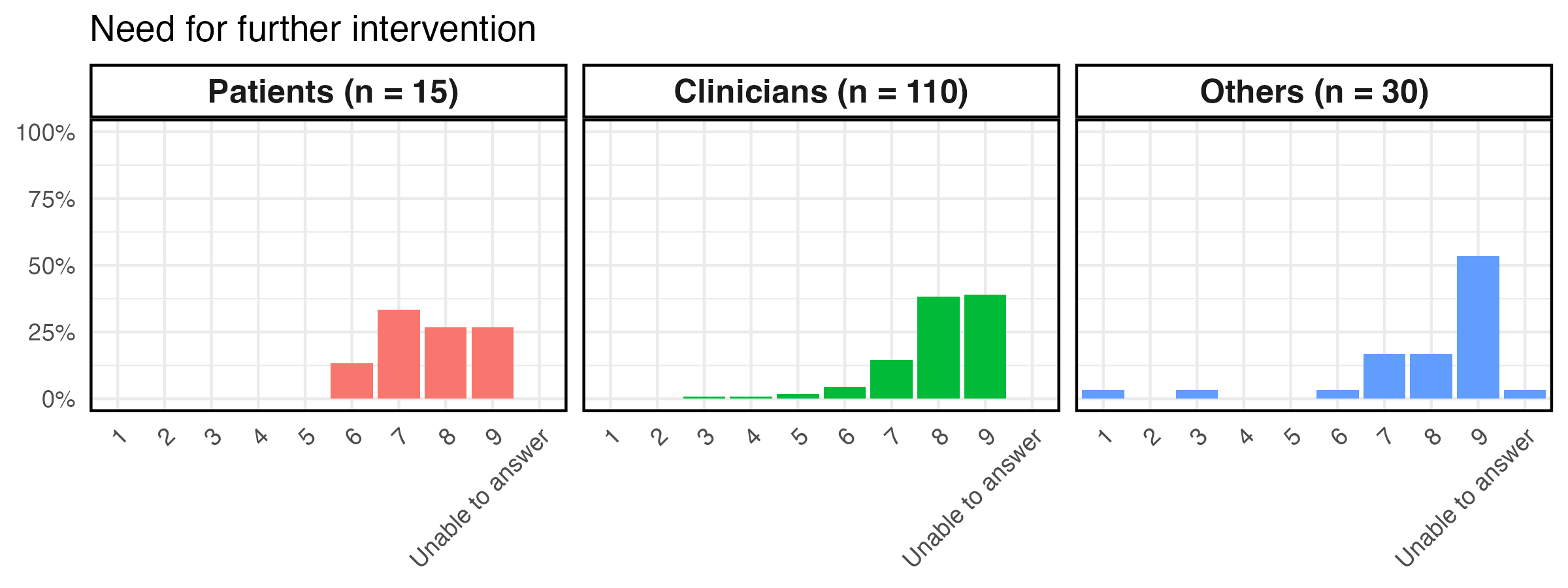

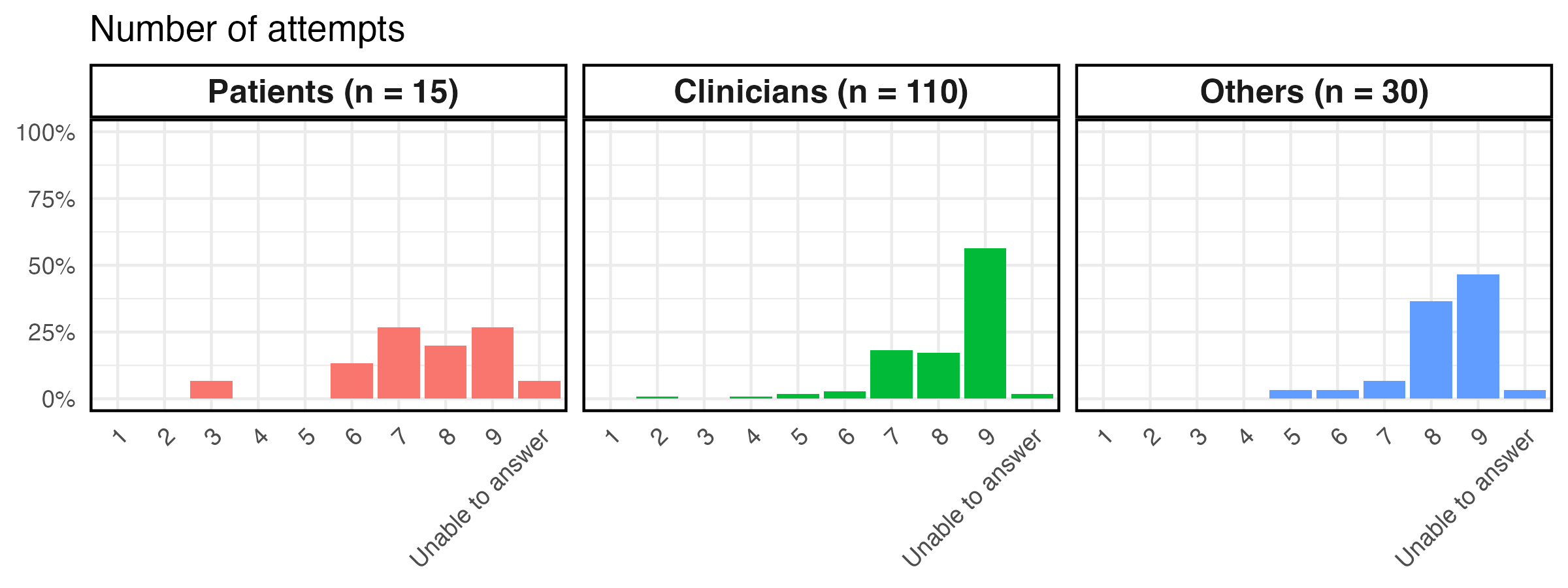

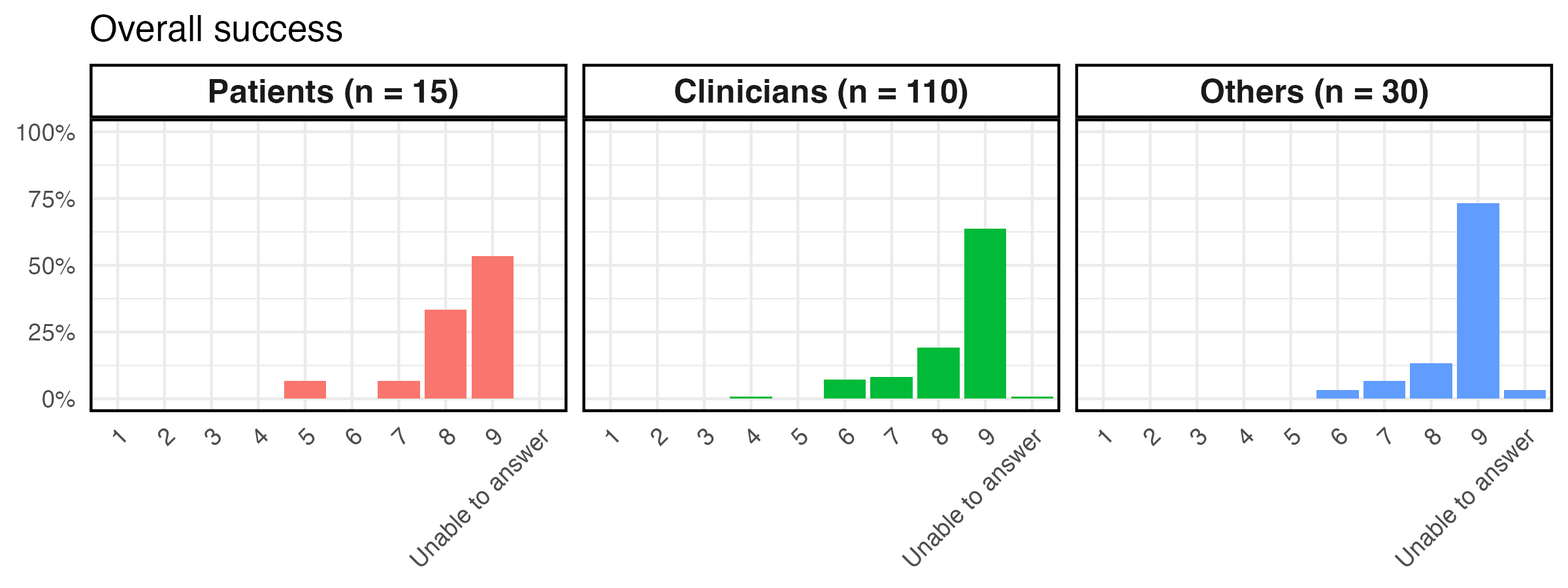

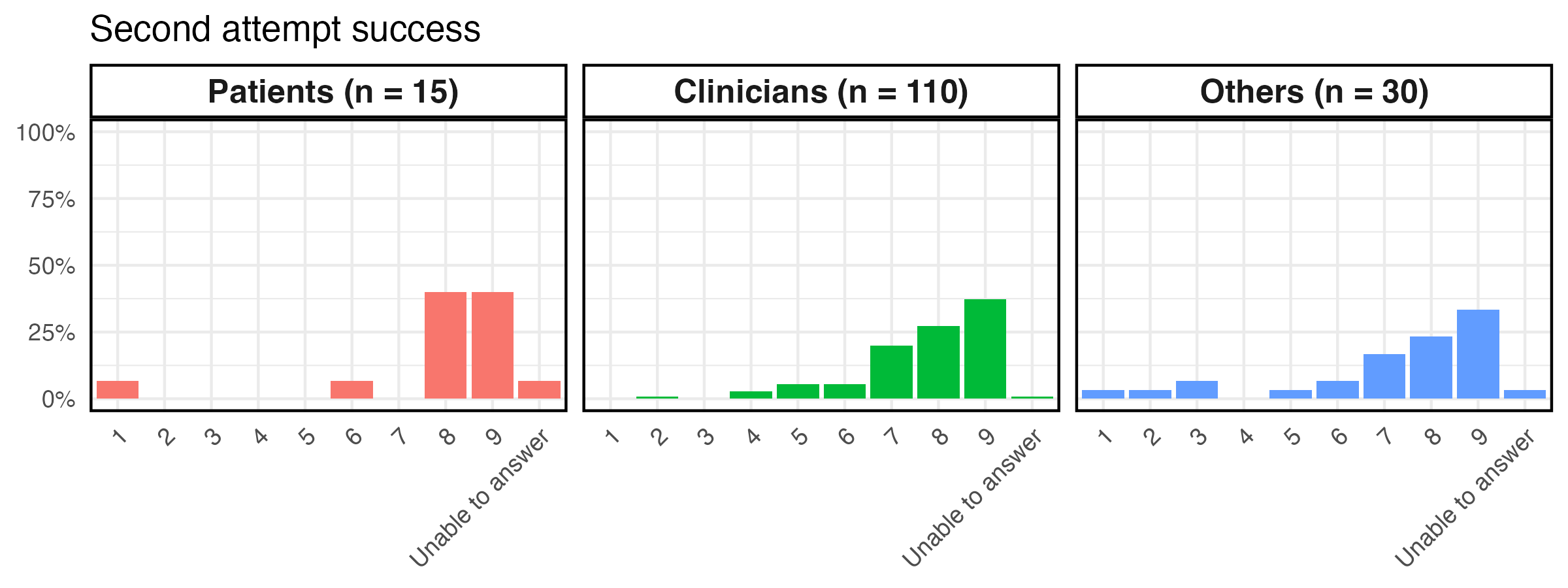

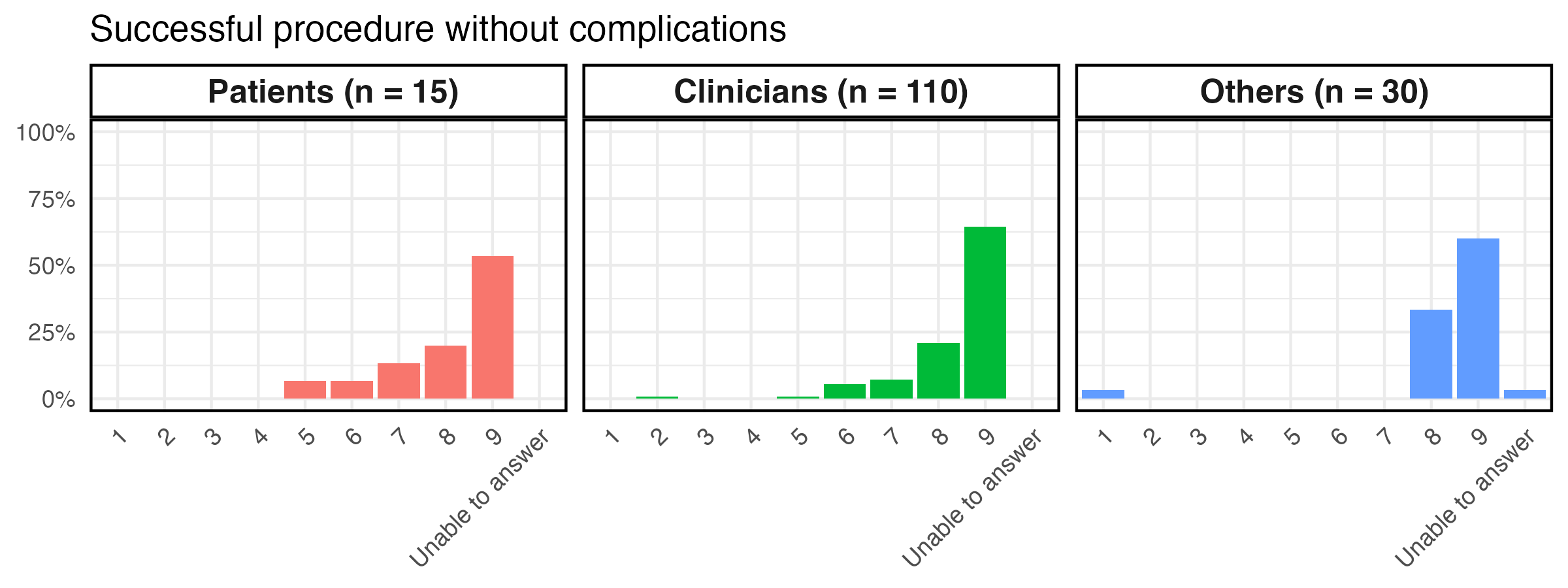

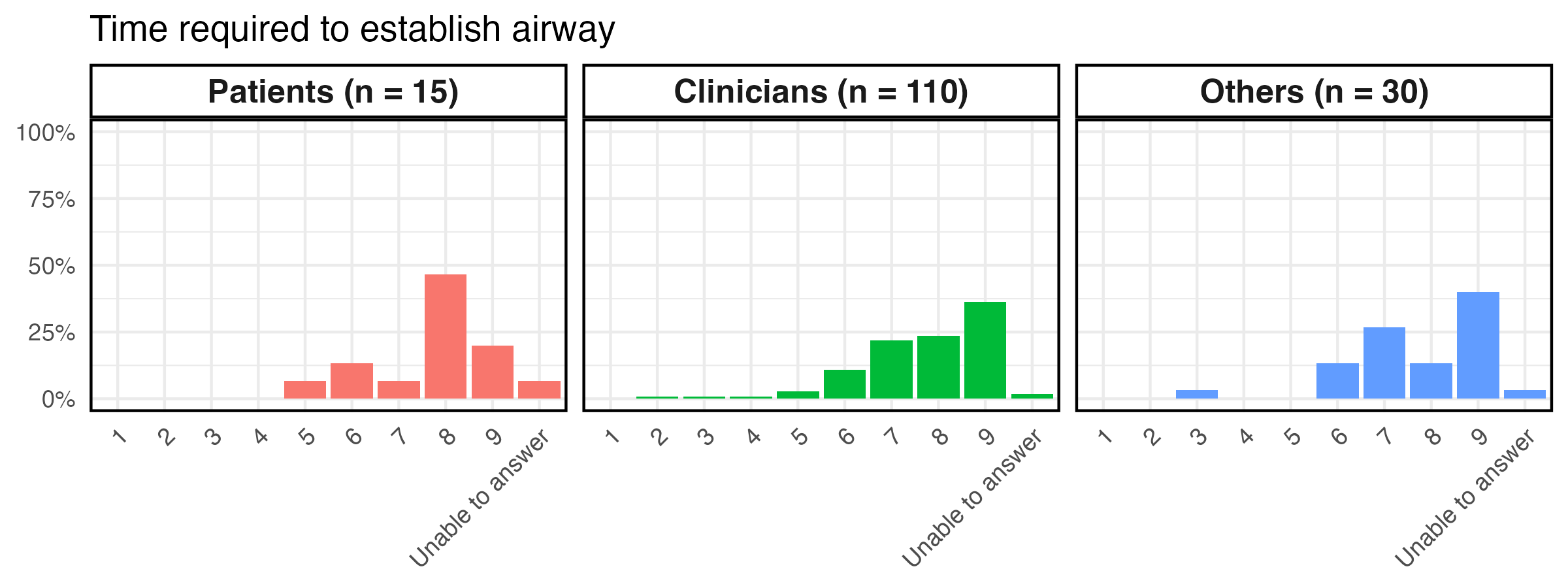

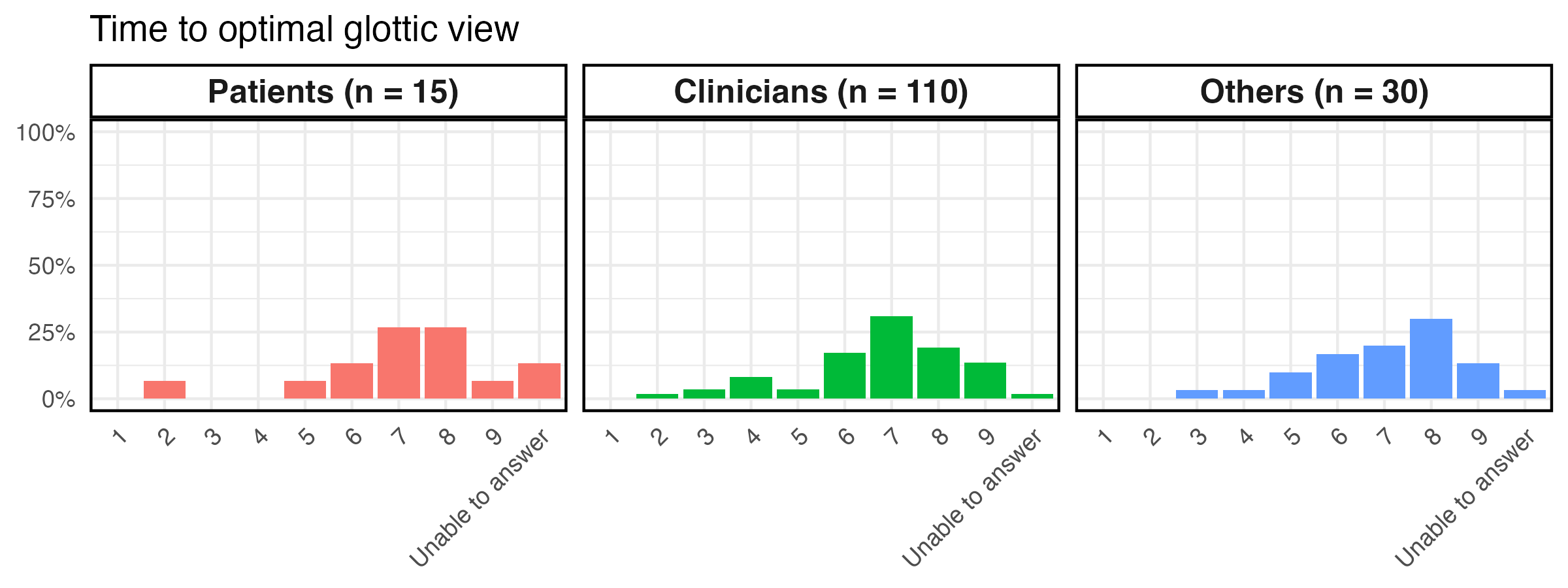

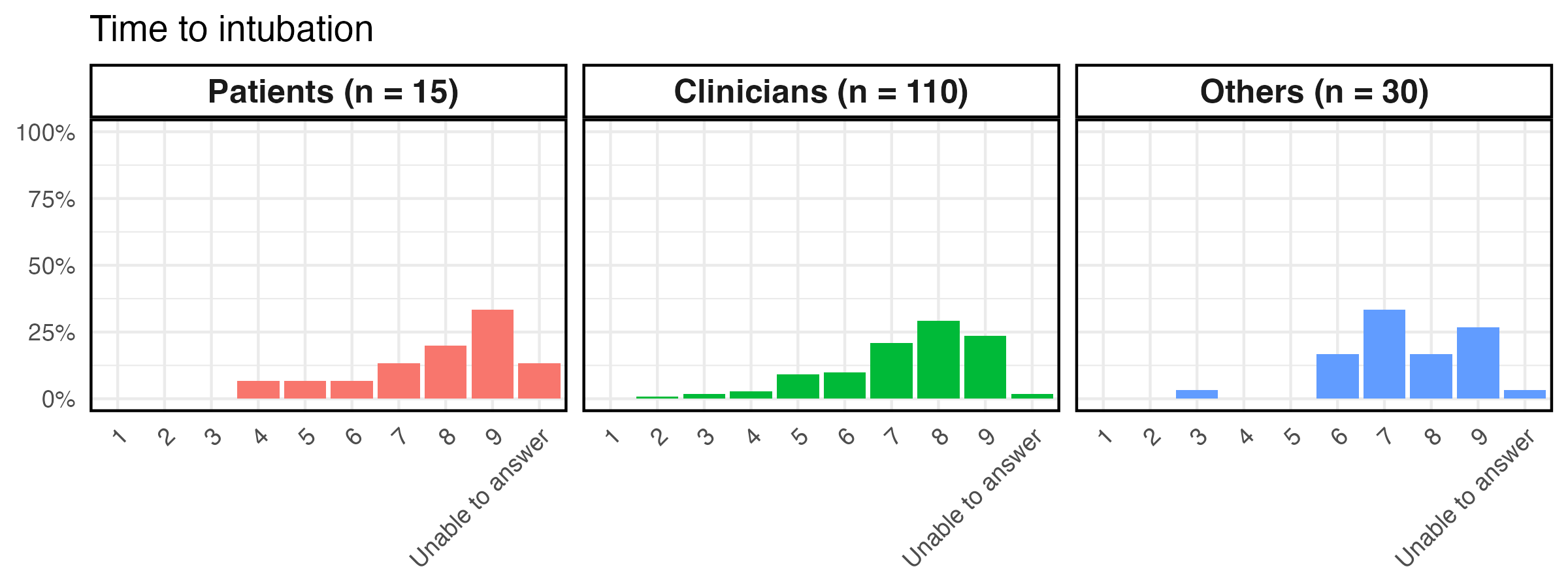

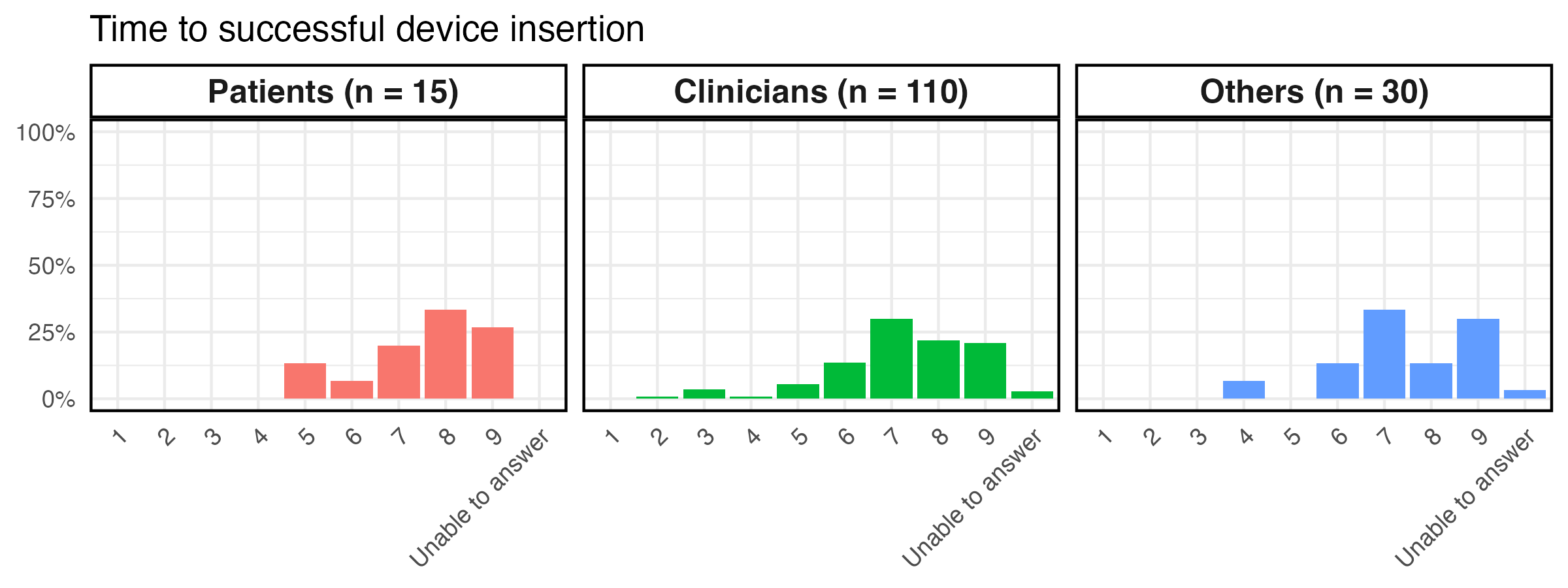

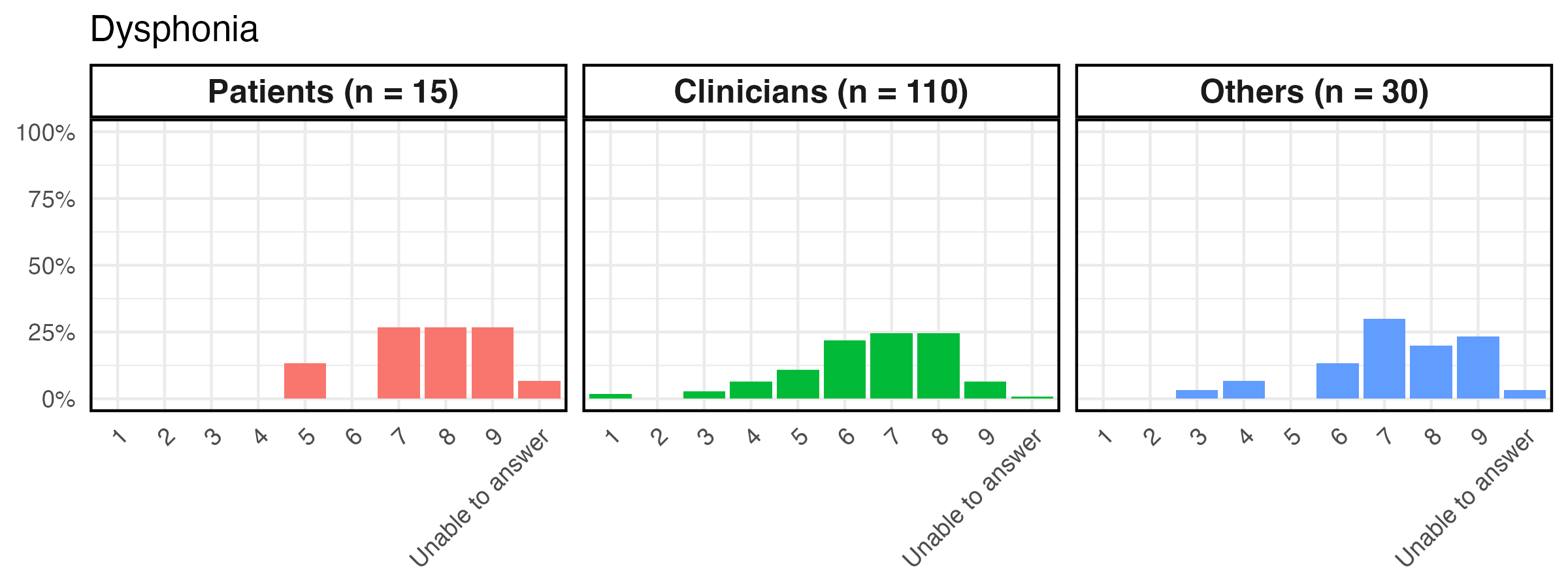

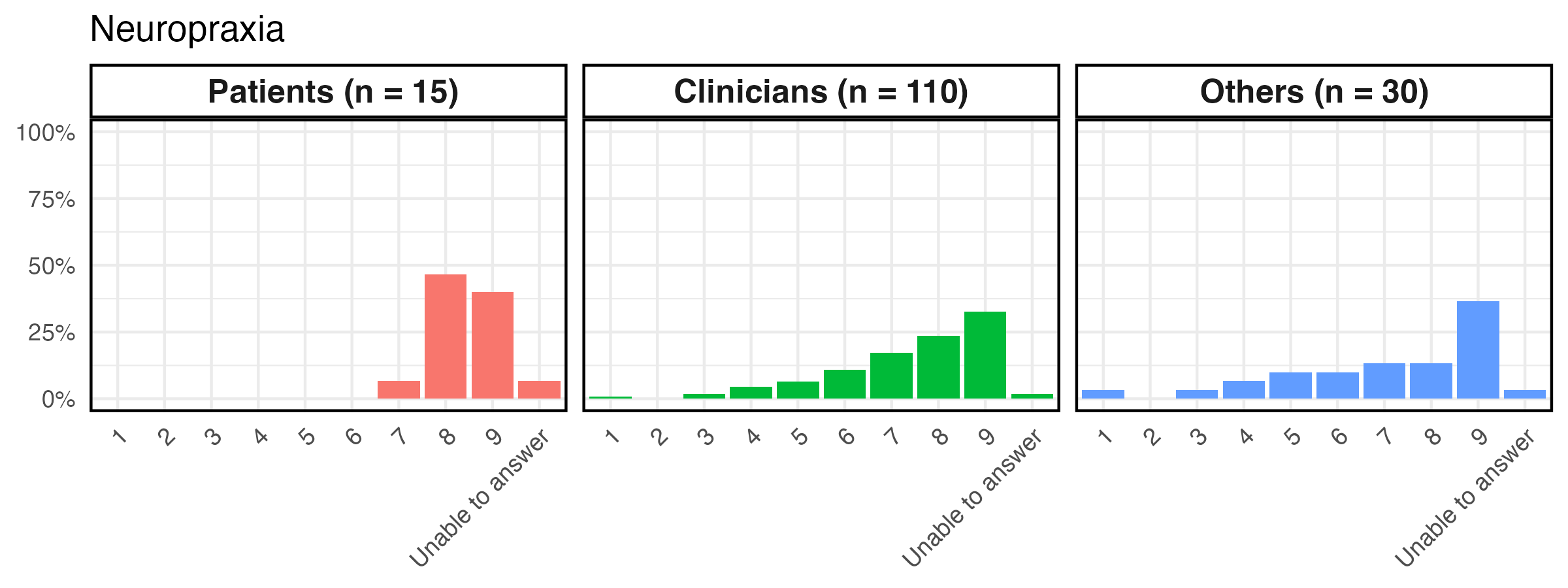

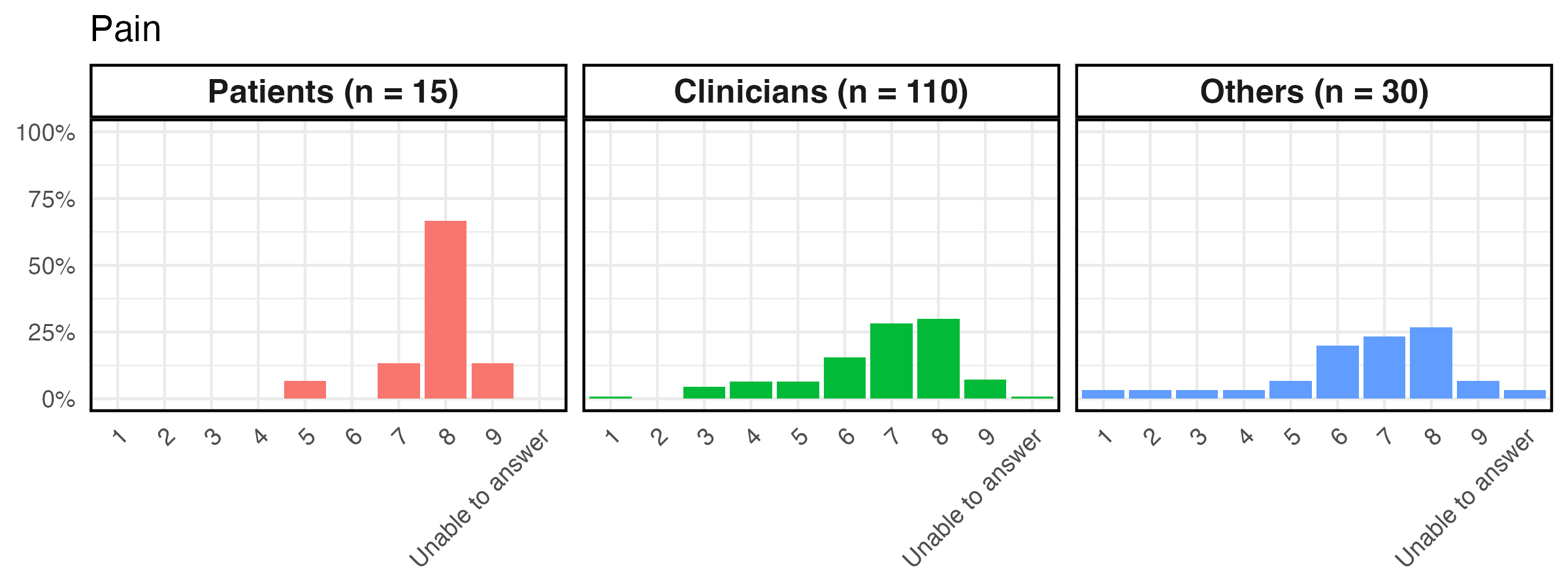

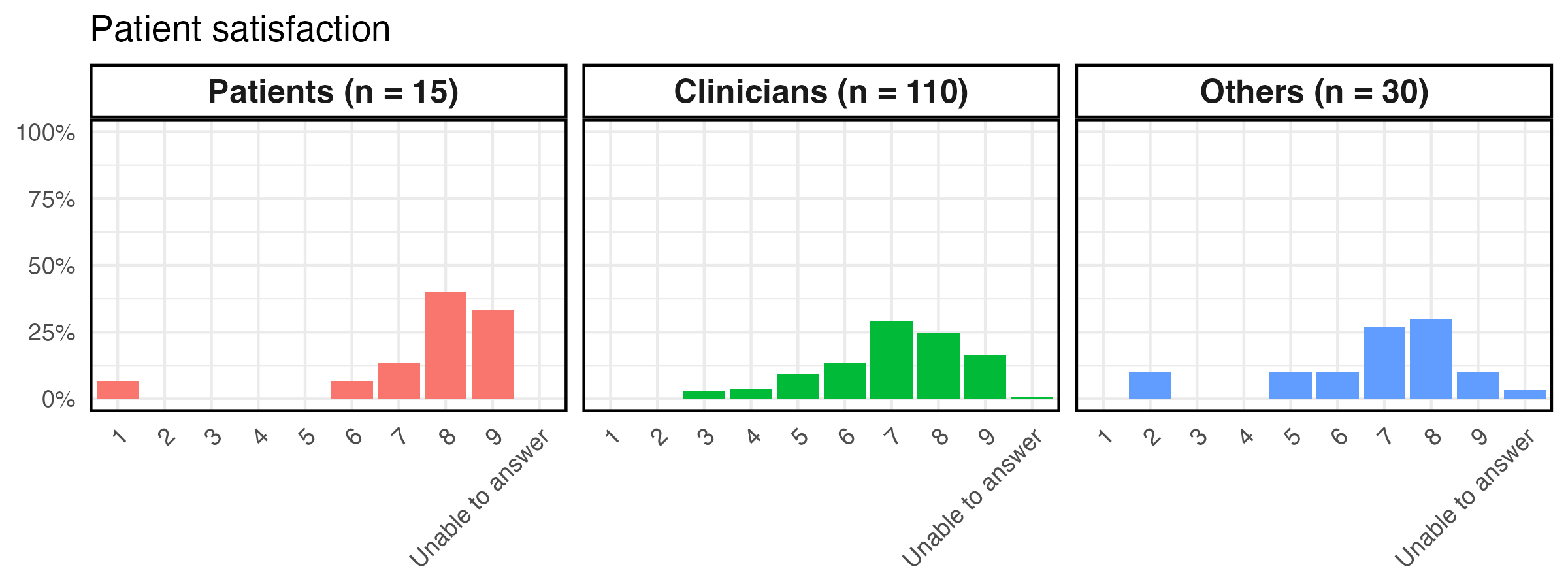


Figure S4 Summary of votes according to stakeholder group in Rounds 1 and 2
