# Supplementary material for: A core outcome set for airway management research
Source: Anaesthesia. 2025 Nov 7;81(3):373–82. doi: 10.1111/anae.70026 (PMC12893836; doi:10.1111/anae.70026)
Supplement: Supplementary file 4 — Table S1. Extracted outcomes. Table S2. Longlisted outcomes. Table S3. Modifications to outcomes following survey rounds. Table S4. Virtual consensus panel voting results for individual outcomes. Table S5. Voting results on proposed modifications to included outcomes. Table S6. Virtual consensus panel voting results for outcome measurement instruments. Table S7. Detailed information on outcomes and outcome measurement instruments. Table S8. Plain language summary of included outcomes. [file ANAE-81-373-s003.docx]

Table S1 Extracted verbatim outcomes with corresponding amalgamations and domain classifications

| **Verbatim outcome** | **Amalgamated outcome** | **Domain (ATOM)** | **Outcome Domain (COMET)** |
| --- | --- | --- | --- |
| Number of patients requiring manipulation or additional propofol for achieving effective airway | Additional interventions | 1: Procedural effectiveness | 32: Delivery of care |
| Use of bougie | Adjunct use | 1: Procedural effectiveness | 32: Delivery of care |
| Number of alternative techniques used | Change of device | 1: Procedural effectiveness | 32: Delivery of care |
| Need for alternative intubation technique | Change of device | 1: Procedural effectiveness | 32: Delivery of care |
| Change of laryngoscope or blade | Change of device | 1: Procedural effectiveness | 32: Delivery of care |
| Number of operators | Change of operator | 1: Procedural effectiveness | 32: Delivery of care |
| Change of operator | Change of operator | 1: Procedural effectiveness | 32: Delivery of care |
| Square wave capnograph | Confirmation of tracheal intubation | 1: Procedural effectiveness | 32: Delivery of care |
| DL tube correct in left main bronchus | Correct device placement | 1: Procedural effectiveness | 32: Delivery of care |
| Adequate placement of the LMA | Correct device placement | 1: Procedural effectiveness | 32: Delivery of care |
| Optimal insertion of the device | Correct device placement | 1: Procedural effectiveness | 32: Delivery of care |
| Device portability | Device function | 1: Procedural effectiveness | 32: Delivery of care |
| Overall preference for device | Device function | 1: Procedural effectiveness | 32: Delivery of care |
| Leak | Device function | 1: Procedural effectiveness | 32: Delivery of care |
| Average cuff pressures | Device function | 1: Procedural effectiveness | 32: Delivery of care |
| Fogging of camera | Device function | 1: Procedural effectiveness | 32: Delivery of care |
| Leak airway pressure (LAP) | Device function | 1: Procedural effectiveness | 32: Delivery of care |
| Oropharyngeal leak pressure | Device function | 1: Procedural effectiveness | 32: Delivery of care |
| Oropharyngeal leak pressure | Device function | 1: Procedural effectiveness | 32: Delivery of care |
| Absence of an audible leak with peak airway pressure 20cm H2O during manual ventilation | Device function | 1: Procedural effectiveness | 32: Delivery of care |
| Audible leak | Device function | 1: Procedural effectiveness | 32: Delivery of care |
| Oropharyngeal seal pressure | Device function | 1: Procedural effectiveness | 32: Delivery of care |
| TT cuff pressure | Device function | 1: Procedural effectiveness | 32: Delivery of care |
| Oropharyngeal leak pressure | Device function | 1: Procedural effectiveness | 32: Delivery of care |
| Seal pressure | Device function | 1: Procedural effectiveness | 32: Delivery of care |
| Airway seal pressure | Device function | 1: Procedural effectiveness | 32: Delivery of care |
| Oropharyngeal leak pressure | Device function | 1: Procedural effectiveness | 32: Delivery of care |
| Device failure | Device function | 1: Procedural effectiveness | 32: Delivery of care |
| Intra-operative device performance | Device function | 1: Procedural effectiveness | 32: Delivery of care |
| Leak volume | Device function | 1: Procedural effectiveness | 32: Delivery of care |
| Oropharyngeal leak pressure | Device function | 1: Procedural effectiveness | 32: Delivery of care |
| Difficulty of airway placement | Difficult device placement | 1: Procedural effectiveness | 32: Delivery of care |
| Difficult intubation | Difficult intubation | 1: Procedural effectiveness | 32: Delivery of care |
| Difficult intubation | Difficult intubation | 1: Procedural effectiveness | 32: Delivery of care |
| Difficult intubation | Difficult intubation | 1: Procedural effectiveness | 32: Delivery of care |
| Difficult tracheal intubation | Difficult intubation | 1: Procedural effectiveness | 32: Delivery of care |
| Difficult intubation | Difficult intubation | 1: Procedural effectiveness | 32: Delivery of care |
| Difficult laryngoscopy | Difficult laryngoscopy | 1: Procedural effectiveness | 32: Delivery of care |
| Difficult laryngoscopy | Difficult laryngoscopy | 1: Procedural effectiveness | 32: Delivery of care |
| Difficult laryngoscopy | Difficult laryngoscopy | 1: Procedural effectiveness | 32: Delivery of care |
| Dysphagia | Difficulty swallowing | 1: Procedural effectiveness | 8: Gastrointestinal outcomes |
| Anaesthesia time | Duration of anaesthesia | 1: Procedural effectiveness | 32: Delivery of care |
| Incidence and duration of the interventions | Duration of interventions | 1: Procedural effectiveness | 32: Delivery of care |
| Time taken for laryngoscopy during extubation | Duration of laryngoscopy during extubation | 1: Procedural effectiveness | 32: Delivery of care |
| Ease of insertion | Ease of device use | 1: Procedural effectiveness | 32: Delivery of care |
| Ease of device use | Ease of device use | 1: Procedural effectiveness | 32: Delivery of care |
| Ease of insertion | Ease of device use | 1: Procedural effectiveness | 32: Delivery of care |
| Ease of insertion | Ease of device use | 1: Procedural effectiveness | 32: Delivery of care |
| Ease of SGA insertion | Ease of device use | 1: Procedural effectiveness | 32: Delivery of care |
| LMA insertion condition | Ease of device use | 1: Procedural effectiveness | 32: Delivery of care |
| Ease of SGA insertion | Ease of device use | 1: Procedural effectiveness | 32: Delivery of care |
| Ease of SGA insertion | Ease of device use | 1: Procedural effectiveness | 32: Delivery of care |
| Ease of device usage | Ease of device use | 1: Procedural effectiveness | 32: Delivery of care |
| Ease of insertion | Ease of device use | 1: Procedural effectiveness | 32: Delivery of care |
| Ease of insertion | Ease of device use | 1: Procedural effectiveness | 32: Delivery of care |
| Ease of insertion | Ease of device use | 1: Procedural effectiveness | 32: Delivery of care |
| Ease of use | Ease of device use | 1: Procedural effectiveness | 32: Delivery of care |
| Ease of device insertion | Ease of device use | 1: Procedural effectiveness | 32: Delivery of care |
| Ease of insertion | Ease of device use | 1: Procedural effectiveness | 32: Delivery of care |
| Ease of intubation | Ease of intubation | 1: Procedural effectiveness | 32: Delivery of care |
| Ease of intubation | Ease of intubation | 1: Procedural effectiveness | 32: Delivery of care |
| Ease of intubation | Ease of intubation | 1: Procedural effectiveness | 32: Delivery of care |
| Ease of intubation | Ease of intubation | 1: Procedural effectiveness | 32: Delivery of care |
| Ease of laryngoscope blade insertion | Ease of laryngoscopy | 1: Procedural effectiveness | 32: Delivery of care |
| Easy laryngoscopy | Ease of laryngoscopy | 1: Procedural effectiveness | 32: Delivery of care |
| Emergence time | Emergence time | 1: Procedural effectiveness | 32: Delivery of care |
| Unsuccessful attempt | Failed attempt | 1: Procedural effectiveness | 32: Delivery of care |
| Malposition | Failed attempt | 1: Procedural effectiveness | 32: Delivery of care |
| Failed SGA insertion | Failed device insertion | 1: Procedural effectiveness | 32: Delivery of care |
| Failed insertion | Failed device insertion | 1: Procedural effectiveness | 32: Delivery of care |
| Failed SGA insertion | Failed device insertion | 1: Procedural effectiveness | 32: Delivery of care |
| Failed intubation | Failed intubation | 1: Procedural effectiveness | 32: Delivery of care |
| Failed intubation | Failed intubation | 1: Procedural effectiveness | 32: Delivery of care |
| Failed tracheal intubation | Failed intubation | 1: Procedural effectiveness | 32: Delivery of care |
| Reason for failed attempts | Failed intubation | 1: Procedural effectiveness | 32: Delivery of care |
| Failed intubation | Failed intubation | 1: Procedural effectiveness | 32: Delivery of care |
| Failed intubation | Failed intubation | 1: Procedural effectiveness | 32: Delivery of care |
| Failed intubation | Failed intubation | 1: Procedural effectiveness | 32: Delivery of care |
| Failed intubation | Failed intubation | 1: Procedural effectiveness | 32: Delivery of care |
| Failed scope intubation | Failed intubation | 1: Procedural effectiveness | 32: Delivery of care |
| Failed intubation | Failed intubation | 1: Procedural effectiveness | 32: Delivery of care |
| Unsuccessful intubation | Failed intubation | 1: Procedural effectiveness | 32: Delivery of care |
| Failed intubation | Failed intubation | 1: Procedural effectiveness | 32: Delivery of care |
| Failed intubation | Failed intubation | 1: Procedural effectiveness | 32: Delivery of care |
| Failed intubation | Failed intubation | 1: Procedural effectiveness | 32: Delivery of care |
| Failed intubation | Failed intubation | 1: Procedural effectiveness | 32: Delivery of care |
| Failed attempt | Failed intubation | 1: Procedural effectiveness | 32: Delivery of care |
| Failed intubation | Failed intubation | 1: Procedural effectiveness | 32: Delivery of care |
| Failed intubation | Failed intubation | 1: Procedural effectiveness | 32: Delivery of care |
| Impossible intubation | Failed intubation | 1: Procedural effectiveness | 32: Delivery of care |
| Failed intubation | Failed intubation | 1: Procedural effectiveness | 32: Delivery of care |
| Failed intubation | Failed intubation | 1: Procedural effectiveness | 32: Delivery of care |
| Failed intubation | Failed intubation | 1: Procedural effectiveness | 32: Delivery of care |
| Failed intubation | Failed intubation | 1: Procedural effectiveness | 32: Delivery of care |
| Successful tracheal intubation on the first attempt | First attempt success | 1: Procedural effectiveness | 32: Delivery of care |
| Success rate with first insertion | First attempt success | 1: Procedural effectiveness | 32: Delivery of care |
| First attempts success rate | First attempt success | 1: Procedural effectiveness | 32: Delivery of care |
| First attempts success rate | First attempt success | 1: Procedural effectiveness | 32: Delivery of care |
| First attempts success rate | First attempt success | 1: Procedural effectiveness | 32: Delivery of care |
| First attempts success rate | First attempt success | 1: Procedural effectiveness | 32: Delivery of care |
| First attempts success rate | First attempt success | 1: Procedural effectiveness | 32: Delivery of care |
| 1st time success rate | First attempt success | 1: Procedural effectiveness | 32: Delivery of care |
| ETI success at first attempt | First attempt success | 1: Procedural effectiveness | 32: Delivery of care |
| First-attempt success of intubation | First attempt success | 1: Procedural effectiveness | 32: Delivery of care |
| First attempt success rate of intubation | First attempt success | 1: Procedural effectiveness | 32: Delivery of care |
| Success on first pass | First attempt success | 1: Procedural effectiveness | 32: Delivery of care |
| First-pass success rate | First attempt success | 1: Procedural effectiveness | 32: Delivery of care |
| First attempt intubation success | First attempt success | 1: Procedural effectiveness | 32: Delivery of care |
| First-pass success rate | First attempt success | 1: Procedural effectiveness | 32: Delivery of care |
| First-time success rate | First attempt success | 1: Procedural effectiveness | 32: Delivery of care |
| Success/failure of intubation at the first attempt | First attempt success | 1: Procedural effectiveness | 32: Delivery of care |
| First pass success | First attempt success | 1: Procedural effectiveness | 32: Delivery of care |
| First attempt success of intubation | First attempt success | 1: Procedural effectiveness | 32: Delivery of care |
| First attempt correct positioning of DLT or blocker | First attempt success | 1: Procedural effectiveness | 32: Delivery of care |
| Glottic view | Glottic view | 1: Procedural effectiveness | 32: Delivery of care |
| Percentage of the vocal cords visible when performing laryngoscopy | Glottic view | 1: Procedural effectiveness | 32: Delivery of care |
| Glottic view | Glottic view | 1: Procedural effectiveness | 32: Delivery of care |
| Grading of the glottis view | Glottic view | 1: Procedural effectiveness | 32: Delivery of care |
| Glottic view | Glottic view | 1: Procedural effectiveness | 32: Delivery of care |
| Laryngoscopy grade | Glottic view | 1: Procedural effectiveness | 32: Delivery of care |
| Fibreoptic view | Glottic view | 1: Procedural effectiveness | 32: Delivery of care |
| View quality | Glottic view | 1: Procedural effectiveness | 32: Delivery of care |
| Cormack-Lehane grade | Glottic view | 1: Procedural effectiveness | 32: Delivery of care |
| Glottic view | Glottic view | 1: Procedural effectiveness | 32: Delivery of care |
| Percentage of glottic opening score | Glottic view | 1: Procedural effectiveness | 32: Delivery of care |
| Laryngoscopy view | Glottic view | 1: Procedural effectiveness | 32: Delivery of care |
| Glottic view | Glottic view | 1: Procedural effectiveness | 32: Delivery of care |
| Cormack and Lehane grade | Glottic view | 1: Procedural effectiveness | 32: Delivery of care |
| Pogo | Glottic view | 1: Procedural effectiveness | 32: Delivery of care |
| Laryngoscopic view | Glottic view | 1: Procedural effectiveness | 32: Delivery of care |
| Cormack–Lehane grade | Glottic view | 1: Procedural effectiveness | 32: Delivery of care |
| Glottic view | Glottic view | 1: Procedural effectiveness | 32: Delivery of care |
| Grade of laryngoscopy view | Glottic view | 1: Procedural effectiveness | 32: Delivery of care |
| Pogo | Glottic view | 1: Procedural effectiveness | 32: Delivery of care |
| Cormack–Lehane grade | Glottic view | 1: Procedural effectiveness | 32: Delivery of care |
| POGO score | Glottic view | 1: Procedural effectiveness | 32: Delivery of care |
| Cormack-lehane | Glottic view | 1: Procedural effectiveness | 32: Delivery of care |
| Glottis exposure grading | Glottic view | 1: Procedural effectiveness | 32: Delivery of care |
| Difficulty of intubation | Intubation difficulty | 1: Procedural effectiveness | 32: Delivery of care |
| IDS score | Intubation difficulty | 1: Procedural effectiveness | 32: Delivery of care |
| Intubation difficulty scale score | Intubation difficulty | 1: Procedural effectiveness | 32: Delivery of care |
| Grade of difficulty | Intubation difficulty | 1: Procedural effectiveness | 32: Delivery of care |
| Difficulty of intubation | Intubation difficulty | 1: Procedural effectiveness | 32: Delivery of care |
| Difficulty of intubation | Intubation difficulty | 1: Procedural effectiveness | 32: Delivery of care |
| Subjective difficulty of laryngoscopy | Intubation difficulty | 1: Procedural effectiveness | 32: Delivery of care |
| Intubation difficulty | Intubation difficulty | 1: Procedural effectiveness | 32: Delivery of care |
| IDS score | Intubation difficulty | 1: Procedural effectiveness | 32: Delivery of care |
| Intubation difficulty | Intubation difficulty | 1: Procedural effectiveness | 32: Delivery of care |
| Operator-assessed difficult intubation | Intubation difficulty | 1: Procedural effectiveness | 32: Delivery of care |
| Intubation difficulty | Intubation difficulty | 1: Procedural effectiveness | 32: Delivery of care |
| Number of optimisation manoeuvres to aid tracheal intubation | Manoeuvres | 1: Procedural effectiveness | 32: Delivery of care |
| Adjustment manoeuvres | Manoeuvres | 1: Procedural effectiveness | 32: Delivery of care |
| Number of patients who required manoeuvres for a good glottic view | Manoeuvres | 1: Procedural effectiveness | 32: Delivery of care |
| Number of optimization manoeuvres required | Manoeuvres | 1: Procedural effectiveness | 32: Delivery of care |
| Need for external laryngeal manipulation | Manoeuvres | 1: Procedural effectiveness | 32: Delivery of care |
| Optimisation manoeuvres (BURP, laryngeal manoeuvre,...) | Manoeuvres | 1: Procedural effectiveness | 32: Delivery of care |
| Optimisation manoeuvres (BURP, laryngeal manoeuvre,...) | Manoeuvres | 1: Procedural effectiveness | 32: Delivery of care |
| Optimisation manoeuvres (BURP, laryngeal manoeuvre,...) | Manoeuvres | 1: Procedural effectiveness | 32: Delivery of care |
| Optimisation manoeuvres (BURP, laryngeal manoeuvre,...) | Manoeuvres | 1: Procedural effectiveness | 32: Delivery of care |
| Manoeuvres | Manoeuvres | 1: Procedural effectiveness | 32: Delivery of care |
| Need for manoeuvres | Manoeuvres | 1: Procedural effectiveness | 32: Delivery of care |
| Chin lift | Manoeuvres | 1: Procedural effectiveness | 32: Delivery of care |
| Jaw thrust | Manoeuvres | 1: Procedural effectiveness | 32: Delivery of care |
| Use of external laryngeal manipulation, stylet or bougie | Manoeuvres | 1: Procedural effectiveness | 32: Delivery of care |
| External laryngeal manipulation | Manoeuvres | 1: Procedural effectiveness | 32: Delivery of care |
| Performance of BURP | Manoeuvres | 1: Procedural effectiveness | 32: Delivery of care |
| Abandonment of technique | Need for further intervention | 1: Procedural effectiveness | 36: Need for further intervention |
| Use of tools / techniques | Need for further intervention | 1: Procedural effectiveness | 36: Need for further intervention |
| Need for mask ventilation | Need for further intervention | 1: Procedural effectiveness | 36: Need for further intervention |
| Different airway equipment requirement | Need for further intervention | 1: Procedural effectiveness | 36: Need for further intervention |
| Number of attempts needed | Number of attempts | 1: Procedural effectiveness | 32: Delivery of care |
| Number of attempts | Number of attempts | 1: Procedural effectiveness | 32: Delivery of care |
| Number of attempts until successful endotracheal intubation | Number of attempts | 1: Procedural effectiveness | 32: Delivery of care |
| Number of intubation attempts | Number of attempts | 1: Procedural effectiveness | 32: Delivery of care |
| Number of attempts | Number of attempts | 1: Procedural effectiveness | 32: Delivery of care |
| Intubating attempts | Number of attempts | 1: Procedural effectiveness | 32: Delivery of care |
| Number of intubation attempts | Number of attempts | 1: Procedural effectiveness | 32: Delivery of care |
| Number of insertion attempts required | Number of attempts | 1: Procedural effectiveness | 32: Delivery of care |
| Number of attempts | Number of attempts | 1: Procedural effectiveness | 32: Delivery of care |
| Intubation attempts | Number of attempts | 1: Procedural effectiveness | 32: Delivery of care |
| Number of intubation attempts | Number of attempts | 1: Procedural effectiveness | 32: Delivery of care |
| Number of scope intubation attempts | Number of attempts | 1: Procedural effectiveness | 32: Delivery of care |
| Number of attempts | Number of attempts | 1: Procedural effectiveness | 32: Delivery of care |
| Number of intubation attempts | Number of attempts | 1: Procedural effectiveness | 32: Delivery of care |
| Number of attempts | Number of attempts | 1: Procedural effectiveness | 32: Delivery of care |
| Number of SGA insertion attempts | Number of attempts | 1: Procedural effectiveness | 32: Delivery of care |
| Number of attempts | Number of attempts | 1: Procedural effectiveness | 32: Delivery of care |
| Number of attempts | Number of attempts | 1: Procedural effectiveness | 32: Delivery of care |
| Number of laryngoscopy | Number of attempts | 1: Procedural effectiveness | 32: Delivery of care |
| Number of attempts | Number of attempts | 1: Procedural effectiveness | 32: Delivery of care |
| Number of attempts | Number of attempts | 1: Procedural effectiveness | 32: Delivery of care |
| Intubation attempts | Number of attempts | 1: Procedural effectiveness | 32: Delivery of care |
| Number of attempts | Number of attempts | 1: Procedural effectiveness | 32: Delivery of care |
| Number of attempts | Number of attempts | 1: Procedural effectiveness | 32: Delivery of care |
| Number of ETI attempts | Number of attempts | 1: Procedural effectiveness | 32: Delivery of care |
| Rate of ETI attempted | Number of attempts | 1: Procedural effectiveness | 32: Delivery of care |
| Number of attempts | Number of attempts | 1: Procedural effectiveness | 32: Delivery of care |
| Number of attempts | Number of attempts | 1: Procedural effectiveness | 32: Delivery of care |
| Attempts | Number of attempts | 1: Procedural effectiveness | 32: Delivery of care |
| Number of attempts | Number of attempts | 1: Procedural effectiveness | 32: Delivery of care |
| Number intubation attempts | Number of attempts | 1: Procedural effectiveness | 32: Delivery of care |
| Intubation attempt | Number of attempts | 1: Procedural effectiveness | 32: Delivery of care |
| Number of attempts | Number of attempts | 1: Procedural effectiveness | 32: Delivery of care |
| Numbers of insertion attempts | Number of attempts | 1: Procedural effectiveness | 32: Delivery of care |
| Number of attempts of intubation | Number of attempts | 1: Procedural effectiveness | 32: Delivery of care |
| Number of attempts of laryngoscopy | Number of attempts | 1: Procedural effectiveness | 32: Delivery of care |
| Number intubation attempts | Number of attempts | 1: Procedural effectiveness | 32: Delivery of care |
| Number of failures | Number of attempts | 1: Procedural effectiveness | 32: Delivery of care |
| Number of attempts | Number of attempts | 1: Procedural effectiveness | 32: Delivery of care |
| Number intubation attempts | Number of attempts | 1: Procedural effectiveness | 32: Delivery of care |
| Intubation attempt | Number of attempts | 1: Procedural effectiveness | 32: Delivery of care |
| Insertion attempts | Number of attempts | 1: Procedural effectiveness | 32: Delivery of care |
| Number of laryngoscopy attempts | Number of attempts | 1: Procedural effectiveness | 32: Delivery of care |
| Rate of successful intubation | Overall success | 1: Procedural effectiveness | 32: Delivery of care |
| Successful tracheal intubation | Overall success | 1: Procedural effectiveness | 32: Delivery of care |
| Success rate of intubation | Overall success | 1: Procedural effectiveness | 32: Delivery of care |
| Procedural success | Overall success | 1: Procedural effectiveness | 32: Delivery of care |
| Overall intubation success rate | Overall success | 1: Procedural effectiveness | 32: Delivery of care |
| Rates of successful device insertion | Overall success | 1: Procedural effectiveness | 32: Delivery of care |
| Success rate with last insertion | Overall success | 1: Procedural effectiveness | 32: Delivery of care |
| Success rates | Overall success | 1: Procedural effectiveness | 32: Delivery of care |
| Rate of successful placement of the ETT in the trachea | Overall success | 1: Procedural effectiveness | 32: Delivery of care |
| Insertion success | Overall success | 1: Procedural effectiveness | 32: Delivery of care |
| Success of procedure | Overall success | 1: Procedural effectiveness | 32: Delivery of care |
| DLT tube passage through the glottis | Overall success | 1: Procedural effectiveness | 32: Delivery of care |
| Performance in anticipated difficult airway | Overall success | 1: Procedural effectiveness | 32: Delivery of care |
| Overall ETI success rate | Overall success | 1: Procedural effectiveness | 32: Delivery of care |
| Appropriate placement of I-gel | Overall success | 1: Procedural effectiveness | 32: Delivery of care |
| Proper device placement | Overall success | 1: Procedural effectiveness | 32: Delivery of care |
| Success rate | Overall success | 1: Procedural effectiveness | 32: Delivery of care |
| Overall success rate | Overall success | 1: Procedural effectiveness | 32: Delivery of care |
| Overall success rate | Overall success | 1: Procedural effectiveness | 32: Delivery of care |
| Successful intubation attempt | Overall success | 1: Procedural effectiveness | 32: Delivery of care |
| Successful endotracheal intubation | Overall success | 1: Procedural effectiveness | 32: Delivery of care |
| Successful LMA insertion | Overall success | 1: Procedural effectiveness | 32: Delivery of care |
| Success rate | Overall success | 1: Procedural effectiveness | 32: Delivery of care |
| Successful sacovlm™ insertion | Overall success | 1: Procedural effectiveness | 32: Delivery of care |
| Procedural time | Procedure time | 1: Procedural effectiveness | 32: Delivery of care |
| Postoperative reintubation | Re-intubation | 1: Procedural effectiveness | 36: Need for further intervention |
| Need for re-use of the laryngoscope blade | Re-use of device | 1: Procedural effectiveness | 36: Need for further intervention |
| Ventilation of the right lung | Right lung ventilation | 1: Procedural effectiveness | 22: Respiratory, thoracic and mediastinal outcomes |
| Successful tracheal intubation on the second attempt | Second attempt success | 1: Procedural effectiveness | 32: Delivery of care |
| Success within two attempts | Second attempt success | 1: Procedural effectiveness | 32: Delivery of care |
| Successful removal of the LMA | Successful removal of device | 1: Procedural effectiveness | 32: Delivery of care |
| Extubation time | Time to extubation | 1: Procedural effectiveness | 32: Delivery of care |
| Elapsed time from anaesthesia induction to first airway intervention | Time to first airway intervention | 1: Procedural effectiveness | 32: Delivery of care |
| Time required for intubation | Time to intubation | 1: Procedural effectiveness | 32: Delivery of care |
| Duration of tracheal intubation | Time to intubation | 1: Procedural effectiveness | 32: Delivery of care |
| Duration of tracheal intubation procedure | Time to intubation | 1: Procedural effectiveness | 32: Delivery of care |
| Duration of intubation | Time to intubation | 1: Procedural effectiveness | 32: Delivery of care |
| Time for tracheal intubation | Time to intubation | 1: Procedural effectiveness | 32: Delivery of care |
| Time to secure the airway | Time to intubation | 1: Procedural effectiveness | 32: Delivery of care |
| Intubation time | Time to intubation | 1: Procedural effectiveness | 32: Delivery of care |
| Time to announcement of best view | Time to intubation | 1: Procedural effectiveness | 32: Delivery of care |
| Time to secure tracheal intubation | Time to intubation | 1: Procedural effectiveness | 32: Delivery of care |
| Total intubation time | Time to intubation | 1: Procedural effectiveness | 32: Delivery of care |
| Intubating time | Time to intubation | 1: Procedural effectiveness | 32: Delivery of care |
| Duration of intubation procedure | Time to intubation | 1: Procedural effectiveness | 32: Delivery of care |
| Time taken to insert the tube | Time to intubation | 1: Procedural effectiveness | 32: Delivery of care |
| Time to intubation | Time to intubation | 1: Procedural effectiveness | 32: Delivery of care |
| Time to intubation | Time to intubation | 1: Procedural effectiveness | 32: Delivery of care |
| Scope intubation times | Time to intubation | 1: Procedural effectiveness | 32: Delivery of care |
| Time to intubation | Time to intubation | 1: Procedural effectiveness | 32: Delivery of care |
| Time required for successful intubation | Time to intubation | 1: Procedural effectiveness | 32: Delivery of care |
| Time required for intubation | Time to intubation | 1: Procedural effectiveness | 32: Delivery of care |
| Time required for intubation | Time to intubation | 1: Procedural effectiveness | 32: Delivery of care |
| Intubation time | Time to intubation | 1: Procedural effectiveness | 32: Delivery of care |
| Time to intubation | Time to intubation | 1: Procedural effectiveness | 32: Delivery of care |
| Time to intubation | Time to intubation | 1: Procedural effectiveness | 32: Delivery of care |
| Time to intubation | Time to intubation | 1: Procedural effectiveness | 32: Delivery of care |
| Time taken for intubation | Time to intubation | 1: Procedural effectiveness | 32: Delivery of care |
| Time required for intubation | Time to intubation | 1: Procedural effectiveness | 32: Delivery of care |
| Intubation time | Time to intubation | 1: Procedural effectiveness | 32: Delivery of care |
| Time to intubation | Time to intubation | 1: Procedural effectiveness | 32: Delivery of care |
| Time for intubation through SGA | Time to intubation | 1: Procedural effectiveness | 32: Delivery of care |
| Time to intubation | Time to intubation | 1: Procedural effectiveness | 32: Delivery of care |
| Intubation time | Time to intubation | 1: Procedural effectiveness | 32: Delivery of care |
| Duration of intubation procedure | Time to intubation | 1: Procedural effectiveness | 32: Delivery of care |
| Intubation time | Time to intubation | 1: Procedural effectiveness | 32: Delivery of care |
| Time for tube insertion | Time to intubation | 1: Procedural effectiveness | 32: Delivery of care |
| Total time for intubation | Time to intubation | 1: Procedural effectiveness | 32: Delivery of care |
| Time taken for intubation | Time to intubation | 1: Procedural effectiveness | 32: Delivery of care |
| Time of insertion | Time to intubation | 1: Procedural effectiveness | 32: Delivery of care |
| Time to intubation | Time to intubation | 1: Procedural effectiveness | 32: Delivery of care |
| Time to intubation | Time to intubation | 1: Procedural effectiveness | 32: Delivery of care |
| Time to intubation | Time to intubation | 1: Procedural effectiveness | 32: Delivery of care |
| Time to effective airway placement | Time to intubation | 1: Procedural effectiveness | 32: Delivery of care |
| Time to intubation | Time to intubation | 1: Procedural effectiveness | 32: Delivery of care |
| Intubation duration | Time to intubation | 1: Procedural effectiveness | 32: Delivery of care |
| Successful intubation duration | Time to intubation | 1: Procedural effectiveness | 32: Delivery of care |
| Time to obtain a good glottic view | Time to optimal glottic view | 1: Procedural effectiveness | 32: Delivery of care |
| Duration of laryngoscopy | Time to optimal glottic view | 1: Procedural effectiveness | 32: Delivery of care |
| Time to glottic visualisation | Time to optimal glottic view | 1: Procedural effectiveness | 32: Delivery of care |
| Time for laryngoscopy | Time to optimal glottic view | 1: Procedural effectiveness | 32: Delivery of care |
| Time for visualization of the glottis | Time to optimal glottic view | 1: Procedural effectiveness | 32: Delivery of care |
| Time required to successfully insert the airway device | Time to successful device insertion | 1: Procedural effectiveness | 32: Delivery of care |
| Time required for successful device insertion | Time to successful device insertion | 1: Procedural effectiveness | 32: Delivery of care |
| Insertion time of SGA | Time to successful device insertion | 1: Procedural effectiveness | 32: Delivery of care |
| Supraglottic airway insertion time | Time to successful device insertion | 1: Procedural effectiveness | 32: Delivery of care |
| Time for SGA placement | Time to successful device insertion | 1: Procedural effectiveness | 32: Delivery of care |
| Time for device insertion | Time to successful device insertion | 1: Procedural effectiveness | 32: Delivery of care |
| Time taken for DLT or blocker | Time to successful device insertion | 1: Procedural effectiveness | 32: Delivery of care |
| Reposition times by bronchoscopy during surgery | Time to successful device insertion | 1: Procedural effectiveness | 32: Delivery of care |
| Time to device insertion | Time to successful device insertion | 1: Procedural effectiveness | 32: Delivery of care |
| Incorrect placement of tracheal tube | Tracheal tube misplacement | 1: Procedural effectiveness | 38: Adverse events/effects |
| pH <7.3 on arterial blood gas analysis (ABGA) | Acidosis | 2: Physiology | 22: Respiratory, thoracic and mediastinal outcomes |
| Changes in mean arterial pressure | Blood pressure | 2: Physiology | 3: Cardiac outcomes |
| Mean arterial blood pressure | Blood pressure | 2: Physiology | 3: Cardiac outcomes |
| Mean arterial blood pressure | Blood pressure | 2: Physiology | 3: Cardiac outcomes |
| Blood pressure | Blood pressure | 2: Physiology | 3: Cardiac outcomes |
| Systolic blood pressure | Blood pressure | 2: Physiology | 3: Cardiac outcomes |
| Diastolic blood pressure | Blood pressure | 2: Physiology | 3: Cardiac outcomes |
| Mean arterial pressure | Blood pressure | 2: Physiology | 3: Cardiac outcomes |
| Systolic blood pressure | Blood pressure | 2: Physiology | 3: Cardiac outcomes |
| Diastolic blood pressure | Blood pressure | 2: Physiology | 3: Cardiac outcomes |
| Mean arterial pressure | Blood pressure | 2: Physiology | 3: Cardiac outcomes |
| Changes in mean arterial pressure | Blood pressure | 2: Physiology | 3: Cardiac outcomes |
| Systolic blood pressure | Blood pressure | 2: Physiology | 3: Cardiac outcomes |
| Diastolic blood pressure | Blood pressure | 2: Physiology | 3: Cardiac outcomes |
| Lowest mean arterial pressure | Blood pressure | 2: Physiology | 3: Cardiac outcomes |
| Blood pressure | Blood pressure | 2: Physiology | 3: Cardiac outcomes |
| Mean arterial blood pressure | Blood pressure | 2: Physiology | 3: Cardiac outcomes |
| Mean blood pressure | Blood pressure | 2: Physiology | 3: Cardiac outcomes |
| Mean arterial pressure during intubation. | Blood pressure | 2: Physiology | 3: Cardiac outcomes |
| Mean blood pressure | Blood pressure | 2: Physiology | 3: Cardiac outcomes |
| Systolic blood pressure | Blood pressure | 2: Physiology | 3: Cardiac outcomes |
| Diastolic blood pressure | Blood pressure | 2: Physiology | 3: Cardiac outcomes |
| MAP | Blood pressure | 2: Physiology | 3: Cardiac outcomes |
| Bradycardia | Bradycardia | 2: Physiology | 3: Cardiac outcomes |
| Bradycardia | Bradycardia | 2: Physiology | 3: Cardiac outcomes |
| Bradycardia | Bradycardia | 2: Physiology | 3: Cardiac outcomes |
| Bradycardia | Bradycardia | 2: Physiology | 3: Cardiac outcomes |
| Bronchospasm | Bronchospasm | 2: Physiology | 22: Respiratory, thoracic and mediastinal outcomes |
| Limb and head movement during SGA insertion | Depth of anaesthesia | 2: Physiology | 17: Nervous system outcomes |
| Bis-spectral index monitoring | Depth of anaesthesia | 2: Physiology | 17: Nervous system outcomes |
| Sedation score | Depth of anaesthesia | 2: Physiology | 17: Nervous system outcomes |
| Glasgow coma score | Depth of anaesthesia | 2: Physiology | 17: Nervous system outcomes |
| Haemodynamic response during laryngoscopy | Haemodynamic response | 2: Physiology | 3: Cardiac outcomes |
| Stress response during laryngoscopy | Haemodynamic response | 2: Physiology | 3: Cardiac outcomes |
| Hemodynamic changes | Haemodynamic response | 2: Physiology | 3: Cardiac outcomes |
| Haemodynamic response | Haemodynamic response | 2: Physiology | 3: Cardiac outcomes |
| Haemodynamic 'parameters' | Haemodynamic response | 2: Physiology | 3: Cardiac outcomes |
| Haemodynamic variables | Haemodynamic response | 2: Physiology | 3: Cardiac outcomes |
| Haemodynamic variables | Haemodynamic response | 2: Physiology | 3: Cardiac outcomes |
| Hemodynamic parameters | Haemodynamic response | 2: Physiology | 3: Cardiac outcomes |
| Haemodynamic response | Haemodynamic response | 2: Physiology | 3: Cardiac outcomes |
| Changes in heart rate | Heart rate | 2: Physiology | 3: Cardiac outcomes |
| Heart rate | Heart rate | 2: Physiology | 3: Cardiac outcomes |
| Heart rate | Heart rate | 2: Physiology | 3: Cardiac outcomes |
| Heart rate | Heart rate | 2: Physiology | 3: Cardiac outcomes |
| Heart rate | Heart rate | 2: Physiology | 3: Cardiac outcomes |
| Heart rate | Heart rate | 2: Physiology | 3: Cardiac outcomes |
| Heart rate | Heart rate | 2: Physiology | 3: Cardiac outcomes |
| Lowest heart rate | Heart rate | 2: Physiology | 3: Cardiac outcomes |
| Tachycardia | Heart rate | 2: Physiology | 3: Cardiac outcomes |
| Heart rate | Heart rate | 2: Physiology | 3: Cardiac outcomes |
| Heart rate | Heart rate | 2: Physiology | 3: Cardiac outcomes |
| Heart rate | Heart rate | 2: Physiology | 3: Cardiac outcomes |
| HR | Heart rate | 2: Physiology | 3: Cardiac outcomes |
| Hypertension | Hypertension | 2: Physiology | 3: Cardiac outcomes |
| Hypertension | Hypertension | 2: Physiology | 3: Cardiac outcomes |
| Hypotension | Hypotension | 2: Physiology | 3: Cardiac outcomes |
| Hypotension | Hypotension | 2: Physiology | 3: Cardiac outcomes |
| Hypotension | Hypotension | 2: Physiology | 3: Cardiac outcomes |
| Hypotension | Hypotension | 2: Physiology | 3: Cardiac outcomes |
| Hypotension | Hypotension | 2: Physiology | 3: Cardiac outcomes |
| Oxygen saturation | Oxygen saturation | 2: Physiology | 22: Respiratory, thoracic and mediastinal outcomes |
| Oxygen saturation | Oxygen saturation | 2: Physiology | 22: Respiratory, thoracic and mediastinal outcomes |
| Oxygen saturation | Oxygen saturation | 2: Physiology | 22: Respiratory, thoracic and mediastinal outcomes |
| Oxygen saturation | Oxygen saturation | 2: Physiology | 22: Respiratory, thoracic and mediastinal outcomes |
| Oxygen saturation | Oxygen saturation | 2: Physiology | 22: Respiratory, thoracic and mediastinal outcomes |
| Lowest SpO2 | Oxygen saturation | 2: Physiology | 22: Respiratory, thoracic and mediastinal outcomes |
| Differences in SpO2 | Oxygen saturation | 2: Physiology | 22: Respiratory, thoracic and mediastinal outcomes |
| Oxygen saturation | Oxygen saturation | 2: Physiology | 22: Respiratory, thoracic and mediastinal outcomes |
| Pao2 | Oxygen saturation | 2: Physiology | 22: Respiratory, thoracic and mediastinal outcomes |
| Lowest peripheral oxygen saturation | Oxygen saturation | 2: Physiology | 22: Respiratory, thoracic and mediastinal outcomes |
| Oxygen saturation of less than 90% for 5 min on oxygen supplementation with flow mask with fio2 0.5 | Oxygen saturation | 2: Physiology | 22: Respiratory, thoracic and mediastinal outcomes |
| SpO2 | Oxygen saturation | 2: Physiology | 22: Respiratory, thoracic and mediastinal outcomes |
| Effectiveness of oxygenation | Oxygenation | 2: Physiology | 22: Respiratory, thoracic and mediastinal outcomes |
| Highest fraction of inspired oxygen | Oxygenation | 2: Physiology | 22: Respiratory, thoracic and mediastinal outcomes |
| Ratio of partial pressure of oxygen to fractional inspired oxygen | Oxygenation | 2: Physiology | 22: Respiratory, thoracic and mediastinal outcomes |
| Loss of pharyngeal tone noted by loss of gagging to suction catheter or marked stridor | Pharyngeal tone | 2: Physiology | 17: Nervous system outcomes |
| Respiratory parameters | Respiratory parameters | 2: Physiology | 22: Respiratory, thoracic and mediastinal outcomes |
| Respiratory rate | Respiratory rate | 2: Physiology | 22: Respiratory, thoracic and mediastinal outcomes |
| Respiratory rate | Respiratory rate | 2: Physiology | 22: Respiratory, thoracic and mediastinal outcomes |
| Respiratory rate | Respiratory rate | 2: Physiology | 22: Respiratory, thoracic and mediastinal outcomes |
| Respiratory rate of more than 40 breaths/min accompanied by accessory muscle use or paradoxical breathing pattern | Respiratory rate | 2: Physiology | 22: Respiratory, thoracic and mediastinal outcomes |
| Neurological response during laryngoscopy | Sympathetic response | 2: Physiology | 17: Nervous system outcomes |
| Ventilation | Ventilation | 2: Physiology | 22: Respiratory, thoracic and mediastinal outcomes |
| Changes in etCO2 | Ventilation | 2: Physiology | 22: Respiratory, thoracic and mediastinal outcomes |
| Changes in minute ventilation | Ventilation | 2: Physiology | 22: Respiratory, thoracic and mediastinal outcomes |
| Changes in peak airway pressure | Ventilation | 2: Physiology | 22: Respiratory, thoracic and mediastinal outcomes |
| Changes in respiratory compliance | Ventilation | 2: Physiology | 22: Respiratory, thoracic and mediastinal outcomes |
| Changes in respiratory resistance | Ventilation | 2: Physiology | 22: Respiratory, thoracic and mediastinal outcomes |
| Changes in work of breathing | Ventilation | 2: Physiology | 22: Respiratory, thoracic and mediastinal outcomes |
| Ability to provide effective ventilation with the iGel during continuous chest compressions | Ventilation | 2: Physiology | 22: Respiratory, thoracic and mediastinal outcomes |
| End-tidal carbon dioxide value | Ventilation | 2: Physiology | 22: Respiratory, thoracic and mediastinal outcomes |
| Measures of ventilation | Ventilation | 2: Physiology | 22: Respiratory, thoracic and mediastinal outcomes |
| End tidal volume | Ventilation | 2: Physiology | 22: Respiratory, thoracic and mediastinal outcomes |
| EtCO2 | Ventilation | 2: Physiology | 22: Respiratory, thoracic and mediastinal outcomes |
| Peak airway pressure | Ventilation | 2: Physiology | 22: Respiratory, thoracic and mediastinal outcomes |
| Ventilation efficiency | Ventilation | 2: Physiology | 22: Respiratory, thoracic and mediastinal outcomes |
| Ventilation efficiency | Ventilation | 2: Physiology | 22: Respiratory, thoracic and mediastinal outcomes |
| Inspired tidal volume | Ventilation | 2: Physiology | 22: Respiratory, thoracic and mediastinal outcomes |
| Expired tidal volume | Ventilation | 2: Physiology | 22: Respiratory, thoracic and mediastinal outcomes |
| Peak airway pressure | Ventilation | 2: Physiology | 22: Respiratory, thoracic and mediastinal outcomes |
| Hypercarbia | Ventilation | 2: Physiology | 22: Respiratory, thoracic and mediastinal outcomes |
| Movement of the chest wall | Ventilation | 2: Physiology | 22: Respiratory, thoracic and mediastinal outcomes |
| Mask ventilation | Ventilation | 2: Physiology | 22: Respiratory, thoracic and mediastinal outcomes |
| End-tidal oxygen and carbon dioxide concentrations | Ventilation | 2: Physiology | 22: Respiratory, thoracic and mediastinal outcomes |
| Minute ventilation | Ventilation | 2: Physiology | 22: Respiratory, thoracic and mediastinal outcomes |
| Tidal volume | Ventilation | 2: Physiology | 22: Respiratory, thoracic and mediastinal outcomes |
| PaCO2 | Ventilation | 2: Physiology | 22: Respiratory, thoracic and mediastinal outcomes |
| Tidal volume on a ventilator | Ventilation | 2: Physiology | 22: Respiratory, thoracic and mediastinal outcomes |
| Highest PEEP | Ventilation | 2: Physiology | 22: Respiratory, thoracic and mediastinal outcomes |
| EtCO2 | Ventilation | 2: Physiology | 22: Respiratory, thoracic and mediastinal outcomes |
| Minute ventilation 2 h after the insertion of airway device | Ventilation | 2: Physiology | 22: Respiratory, thoracic and mediastinal outcomes |
| PaCO2 | Ventilation | 2: Physiology | 22: Respiratory, thoracic and mediastinal outcomes |
| Peak inspiratory pressures | Ventilation | 2: Physiology | 22: Respiratory, thoracic and mediastinal outcomes |
| Plateau airway pressures | Ventilation | 2: Physiology | 22: Respiratory, thoracic and mediastinal outcomes |
| Difference in paCO2 at 2h after insertion of airway devices | Ventilation | 2: Physiology | 22: Respiratory, thoracic and mediastinal outcomes |
| Regional ventilation delay | Ventilation | 2: Physiology | 22: Respiratory, thoracic and mediastinal outcomes |
| Centre of ventilation | Ventilation | 2: Physiology | 22: Respiratory, thoracic and mediastinal outcomes |
| Expired tidal volume | Ventilation | 2: Physiology | 22: Respiratory, thoracic and mediastinal outcomes |
| PaCO2 | Ventilation | 2: Physiology | 22: Respiratory, thoracic and mediastinal outcomes |
| Peak inspiratory pressure | Ventilation | 2: Physiology | 22: Respiratory, thoracic and mediastinal outcomes |
| Driving pressure | Ventilation | 2: Physiology | 22: Respiratory, thoracic and mediastinal outcomes |
| Tidal volume | Ventilation | 2: Physiology | 22: Respiratory, thoracic and mediastinal outcomes |
| Partial pressure of carbon dioxide (PaCO2) >60 mmHg | Ventilation | 2: Physiology | 22: Respiratory, thoracic and mediastinal outcomes |
| Surgical assessment of lung isolation | Ventilation | 2: Physiology | 22: Respiratory, thoracic and mediastinal outcomes |
| Vocal cord mobility | Vocal cord position | 2: Physiology | 32: Delivery of care |
| Position of vocal cords | Vocal cord position | 2: Physiology | 32: Delivery of care |
| Adverse events | Adverse events | 3: Adverse events | 38: Adverse events/effects |
| Postoperative airway complications | Airway complications | 3: Adverse events | 22: Respiratory, thoracic and mediastinal outcomes |
| Trauma to the airway | Airway trauma | 3: Adverse events | 22: Respiratory, thoracic and mediastinal outcomes |
| Traumatic complications | Airway trauma | 3: Adverse events | 22: Respiratory, thoracic and mediastinal outcomes |
| Traumatic injuries related to the tracheal intubation: mucosal bleeding, laryngeal, tracheal, mediastinal or oesophageal injuries | Airway trauma | 3: Adverse events | 22: Respiratory, thoracic and mediastinal outcomes |
| Arrhythmia | Arrhythmia | 3: Adverse events | 3: Cardiac outcomes |
| Dysrhythmia | Arrhythmia | 3: Adverse events | 3: Cardiac outcomes |
| Arrhythmia | Arrhythmia | 3: Adverse events | 3: Cardiac outcomes |
| Corrected QT interval | Arrhythmia | 3: Adverse events | 3: Cardiac outcomes |
| Aspiration | Aspiration | 3: Adverse events | 22: Respiratory, thoracic and mediastinal outcomes |
| Regurgitation and aspiration of stomach contents | Aspiration | 3: Adverse events | 22: Respiratory, thoracic and mediastinal outcomes |
| Incidence of pulmonary aspiration | Aspiration | 3: Adverse events | 22: Respiratory, thoracic and mediastinal outcomes |
| Operator-reported aspiration | Aspiration | 3: Adverse events | 22: Respiratory, thoracic and mediastinal outcomes |
| Pulmonary aspiration | Aspiration | 3: Adverse events | 22: Respiratory, thoracic and mediastinal outcomes |
| Pulmonary aspiration | Aspiration | 3: Adverse events | 22: Respiratory, thoracic and mediastinal outcomes |
| Presence of clinical aspiration | Aspiration | 3: Adverse events | 22: Respiratory, thoracic and mediastinal outcomes |
| Frequency of suspected aspiration pneumonia | Aspiration pneumonia | 3: Adverse events | 22: Respiratory, thoracic and mediastinal outcomes |
| Bleeding | Blood loss | 3: Adverse events | 13: Injury and poisoning outcomes |
| Blood loss | Blood loss | 3: Adverse events | 13: Injury and poisoning outcomes |
| Bronchus injury | Bronchial injury | 3: Adverse events | 38: Adverse events/effects |
| Cardiac arrest | Cardiac arrest | 3: Adverse events | 38: Adverse events/effects |
| Cardiac arrest | Cardiac arrest | 3: Adverse events | 38: Adverse events/effects |
| Cardiac arrest | Cardiac arrest | 3: Adverse events | 38: Adverse events/effects |
| Severe [cardiovascular] collapse | Circulatory collapse | 3: Adverse events | 3: Cardiac outcomes |
| Serious complications | Complications | 3: Adverse events | 38: Adverse events/effects |
| Problems | Complications | 3: Adverse events | 38: Adverse events/effects |
| Complication of intubation | Complications | 3: Adverse events | 38: Adverse events/effects |
| Complications or side effects of drug | Complications | 3: Adverse events | 38: Adverse events/effects |
| Adverse events | Complications | 3: Adverse events | 38: Adverse events/effects |
| Airway complications | Complications | 3: Adverse events | 38: Adverse events/effects |
| Complications of intubation | Complications | 3: Adverse events | 38: Adverse events/effects |
| Complications during intubation | Complications | 3: Adverse events | 38: Adverse events/effects |
| Complications | Complications | 3: Adverse events | 38: Adverse events/effects |
| Intubation-related complications | Complications | 3: Adverse events | 38: Adverse events/effects |
| Side effects related to anaesthesia were documented | Complications | 3: Adverse events | 38: Adverse events/effects |
| Complications | Complications | 3: Adverse events | 38: Adverse events/effects |
| Complications during intubation | Complications | 3: Adverse events | 38: Adverse events/effects |
| Complication of intubation | Complications | 3: Adverse events | 38: Adverse events/effects |
| Complications of intubation | Complications | 3: Adverse events | 38: Adverse events/effects |
| Non pulmonary complications | Complications | 3: Adverse events | 38: Adverse events/effects |
| Intubation complication | Complications | 3: Adverse events | 38: Adverse events/effects |
| Complications of intubation | Complications | 3: Adverse events | 38: Adverse events/effects |
| Complications of intubation | Complications | 3: Adverse events | 38: Adverse events/effects |
| Postoperative complications | Complications | 3: Adverse events | 38: Adverse events/effects |
| Death | Death | 3: Adverse events | 1: Mortality/survival |
| Dental trauma | Dental trauma | 3: Adverse events | 38: Adverse events/effects |
| Dental trauma | Dental trauma | 3: Adverse events | 38: Adverse events/effects |
| Damage to teeth | Dental trauma | 3: Adverse events | 38: Adverse events/effects |
| Dental injury | Dental trauma | 3: Adverse events | 38: Adverse events/effects |
| Dental/mucosa/lip trauma | Dental trauma | 3: Adverse events | 38: Adverse events/effects |
| Dental trauma | Dental trauma | 3: Adverse events | 38: Adverse events/effects |
| Dental trauma | Dental trauma | 3: Adverse events | 38: Adverse events/effects |
| Damage to teeth | Dental trauma | 3: Adverse events | 38: Adverse events/effects |
| Endobronchial intubation | Endobronchial intubation | 3: Adverse events | 38: Adverse events/effects |
| Endobronchial intubation | Endobronchial intubation | 3: Adverse events | 38: Adverse events/effects |
| Traumatic epistaxis | Epistaxis | 3: Adverse events | 22: Respiratory, thoracic and mediastinal outcomes |
| Episodes of oxygen desaturation | Hypoxaemia | 3: Adverse events | 22: Respiratory, thoracic and mediastinal outcomes |
| Hypoxia | Hypoxaemia | 3: Adverse events | 22: Respiratory, thoracic and mediastinal outcomes |
| Hypoxia | Hypoxaemia | 3: Adverse events | 22: Respiratory, thoracic and mediastinal outcomes |
| Oxygen desaturation | Hypoxaemia | 3: Adverse events | 22: Respiratory, thoracic and mediastinal outcomes |
| Desaturation during procedure | Hypoxaemia | 3: Adverse events | 22: Respiratory, thoracic and mediastinal outcomes |
| Hypoxia <92% | Hypoxaemia | 3: Adverse events | 22: Respiratory, thoracic and mediastinal outcomes |
| Desaturation | Hypoxaemia | 3: Adverse events | 22: Respiratory, thoracic and mediastinal outcomes |
| Hypoxia | Hypoxaemia | 3: Adverse events | 22: Respiratory, thoracic and mediastinal outcomes |
| Hypoxaemia | Hypoxaemia | 3: Adverse events | 22: Respiratory, thoracic and mediastinal outcomes |
| Hypoxia | Hypoxaemia | 3: Adverse events | 22: Respiratory, thoracic and mediastinal outcomes |
| Hypoxia | Hypoxaemia | 3: Adverse events | 22: Respiratory, thoracic and mediastinal outcomes |
| Hypoxaemia | Hypoxaemia | 3: Adverse events | 22: Respiratory, thoracic and mediastinal outcomes |
| Oxygen desaturation | Hypoxaemia | 3: Adverse events | 22: Respiratory, thoracic and mediastinal outcomes |
| Arterial oxygen desaturation | Hypoxaemia | 3: Adverse events | 22: Respiratory, thoracic and mediastinal outcomes |
| Severe hypoxaemia | Hypoxaemia | 3: Adverse events | 22: Respiratory, thoracic and mediastinal outcomes |
| Desaturation (hypoxaemia) | Hypoxaemia | 3: Adverse events | 22: Respiratory, thoracic and mediastinal outcomes |
| Desaturation (hypoxaemia) | Hypoxaemia | 3: Adverse events | 22: Respiratory, thoracic and mediastinal outcomes |
| Arterial pressure of oxygen (PaO2) <60 mmHg on an arterial blood gas | Hypoxaemia | 3: Adverse events | 22: Respiratory, thoracic and mediastinal outcomes |
| Oxygen desaturation (hypoxaemia) | Hypoxaemia | 3: Adverse events | 22: Respiratory, thoracic and mediastinal outcomes |
| Laryngeal trauma | Laryngeal trauma | 3: Adverse events | 22: Respiratory, thoracic and mediastinal outcomes |
| Laryngospasm | Laryngospasm | 3: Adverse events | 22: Respiratory, thoracic and mediastinal outcomes |
| Mortality | Mortality | 3: Adverse events | 1: Mortality/survival |
| 28-day mortality | Mortality | 3: Adverse events | 1: Mortality/survival |
| 90-d mortality | Mortality | 3: Adverse events | 1: Mortality/survival |
| Death | Mortality | 3: Adverse events | 1: Mortality/survival |
| Placement of surgical airway | Need for surgical airway | 3: Adverse events | 36: Need for further intervention |
| Post intubation neurological assessment | Neurological complications | 3: Adverse events | 17: Nervous system outcomes |
| Change in motor power following intubation | Neurological complications | 3: Adverse events | 17: Nervous system outcomes |
| Modified Glasgow outcome score | Neurological complications | 3: Adverse events | 17: Nervous system outcomes |
| Oesophageal intubation | Oesophageal intubation | 3: Adverse events | 38: Adverse events/effects |
| Erroneous oesophageal intubation | Oesophageal intubation | 3: Adverse events | 38: Adverse events/effects |
| Frequency of oesophageal intubation | Oesophageal intubation | 3: Adverse events | 38: Adverse events/effects |
| Oesophageal intubation | Oesophageal intubation | 3: Adverse events | 38: Adverse events/effects |
| Oesophageal intubation | Oesophageal intubation | 3: Adverse events | 38: Adverse events/effects |
| Oesophageal intubation | Oesophageal intubation | 3: Adverse events | 38: Adverse events/effects |
| Oesophageal intubation | Oesophageal intubation | 3: Adverse events | 38: Adverse events/effects |
| Oesophageal intubation | Oesophageal intubation | 3: Adverse events | 38: Adverse events/effects |
| Oesophageal intubation | Oesophageal intubation | 3: Adverse events | 38: Adverse events/effects |
| Oesophageal intubation | Oesophageal intubation | 3: Adverse events | 38: Adverse events/effects |
| Oesophageal intubation | Oesophageal intubation | 3: Adverse events | 38: Adverse events/effects |
| Oesophageal intubation | Oesophageal intubation | 3: Adverse events | 38: Adverse events/effects |
| Oesophageal intubation | Oesophageal intubation | 3: Adverse events | 38: Adverse events/effects |
| Blood on the airway device | Oropharyngeal trauma | 3: Adverse events | 22: Respiratory, thoracic and mediastinal outcomes |
| Oropharyngeal trauma | Oropharyngeal trauma | 3: Adverse events | 22: Respiratory, thoracic and mediastinal outcomes |
| Bleeding from the oropharynx | Oropharyngeal trauma | 3: Adverse events | 22: Respiratory, thoracic and mediastinal outcomes |
| Lip injury | Oropharyngeal trauma | 3: Adverse events | 22: Respiratory, thoracic and mediastinal outcomes |
| Mucosal trauma | Oropharyngeal trauma | 3: Adverse events | 22: Respiratory, thoracic and mediastinal outcomes |
| Evaluation of potential trauma | Oropharyngeal trauma | 3: Adverse events | 22: Respiratory, thoracic and mediastinal outcomes |
| Oral trauma | Oropharyngeal trauma | 3: Adverse events | 22: Respiratory, thoracic and mediastinal outcomes |
| Airway trauma | Oropharyngeal trauma | 3: Adverse events | 22: Respiratory, thoracic and mediastinal outcomes |
| Airway trauma | Oropharyngeal trauma | 3: Adverse events | 22: Respiratory, thoracic and mediastinal outcomes |
| Trauma to the airway | Oropharyngeal trauma | 3: Adverse events | 22: Respiratory, thoracic and mediastinal outcomes |
| Gum trauma | Oropharyngeal trauma | 3: Adverse events | 22: Respiratory, thoracic and mediastinal outcomes |
| Tongue trauma | Oropharyngeal trauma | 3: Adverse events | 22: Respiratory, thoracic and mediastinal outcomes |
| Tissue damage | Oropharyngeal trauma | 3: Adverse events | 22: Respiratory, thoracic and mediastinal outcomes |
| Airway trauma | Oropharyngeal trauma | 3: Adverse events | 22: Respiratory, thoracic and mediastinal outcomes |
| Airway trauma | Oropharyngeal trauma | 3: Adverse events | 22: Respiratory, thoracic and mediastinal outcomes |
| Soft tissue trauma | Oropharyngeal trauma | 3: Adverse events | 22: Respiratory, thoracic and mediastinal outcomes |
| Epistaxis | Oropharyngeal trauma | 3: Adverse events | 22: Respiratory, thoracic and mediastinal outcomes |
| Incidence of larynx injury and blood on the ETT | Oropharyngeal trauma | 3: Adverse events | 22: Respiratory, thoracic and mediastinal outcomes |
| Mucosal injury | Oropharyngeal trauma | 3: Adverse events | 22: Respiratory, thoracic and mediastinal outcomes |
| Postoperative pulmonary complication | Pulmonary complications | 3: Adverse events | 22: Respiratory, thoracic and mediastinal outcomes |
| Regurgitation | Regurgitation | 3: Adverse events | 22: Respiratory, thoracic and mediastinal outcomes |
| Survival to ICU discharge | Survival | 3: Adverse events | 1: Mortality/survival |
| Survival | Survival | 3: Adverse events | 1: Mortality/survival |
| Disease free survival | Survival | 3: Adverse events | 1: Mortality/survival |
| Agitation | Agitation | 4: Patient-reported outcomes | 21: Psychiatric outcomes |
| Coughing and gagging during SGA insertion | Coughing | 4: Patient-reported outcomes | 22: Respiratory, thoracic and mediastinal outcomes |
| Bucking | Coughing | 4: Patient-reported outcomes | 22: Respiratory, thoracic and mediastinal outcomes |
| Coughing | Coughing | 4: Patient-reported outcomes | 22: Respiratory, thoracic and mediastinal outcomes |
| Onset of cough | Coughing | 4: Patient-reported outcomes | 22: Respiratory, thoracic and mediastinal outcomes |
| Severity of cough | Coughing | 4: Patient-reported outcomes | 22: Respiratory, thoracic and mediastinal outcomes |
| Coughing | Coughing | 4: Patient-reported outcomes | 22: Respiratory, thoracic and mediastinal outcomes |
| Hoarseness | Hoarseness | 4: Patient-reported outcomes | 22: Respiratory, thoracic and mediastinal outcomes |
| Hoarseness | Hoarseness | 4: Patient-reported outcomes | 22: Respiratory, thoracic and mediastinal outcomes |
| Hoarseness | Hoarseness | 4: Patient-reported outcomes | 22: Respiratory, thoracic and mediastinal outcomes |
| Hoarseness | Hoarseness | 4: Patient-reported outcomes | 22: Respiratory, thoracic and mediastinal outcomes |
| Hoarseness | Hoarseness | 4: Patient-reported outcomes | 22: Respiratory, thoracic and mediastinal outcomes |
| Hoarseness | Hoarseness | 4: Patient-reported outcomes | 22: Respiratory, thoracic and mediastinal outcomes |
| Hoarseness | Hoarseness | 4: Patient-reported outcomes | 22: Respiratory, thoracic and mediastinal outcomes |
| Hoarseness | Hoarseness | 4: Patient-reported outcomes | 22: Respiratory, thoracic and mediastinal outcomes |
| Hoarseness | Hoarseness | 4: Patient-reported outcomes | 22: Respiratory, thoracic and mediastinal outcomes |
| Hoarseness | Hoarseness | 4: Patient-reported outcomes | 22: Respiratory, thoracic and mediastinal outcomes |
| Nausea | Nausea | 4: Patient-reported outcomes | 8: Gastrointestinal outcomes |
| PONV | Nausea | 4: Patient-reported outcomes | 8: Gastrointestinal outcomes |
| Nausea | Nausea | 4: Patient-reported outcomes | 8: Gastrointestinal outcomes |
| Use of postoperative analgesic agents | Pain | 4: Patient-reported outcomes | 17: Nervous system outcomes |
| Pain intensity during procedure | Pain | 4: Patient-reported outcomes | 17: Nervous system outcomes |
| Painful nose | Pain | 4: Patient-reported outcomes | 17: Nervous system outcomes |
| Pain | Pain | 4: Patient-reported outcomes | 17: Nervous system outcomes |
| Total intraoperative morphine consumption | Pain relief requirement | 4: Patient-reported outcomes | 17: Nervous system outcomes |
| Patient discomfort | Patient comfort | 4: Patient-reported outcomes | 32: Delivery of care |
| Position during surgery | Patient position | 4: Patient-reported outcomes | 32: Delivery of care |
| Patient satisfaction | Patient satisfaction | 4: Patient-reported outcomes | 32: Delivery of care |
| Patient satisfaction with the intubation process | Patient satisfaction | 4: Patient-reported outcomes | 32: Delivery of care |
| Restlessness score | Restlessness | 4: Patient-reported outcomes | 17: Nervous system outcomes |
| Restlessness | Restlessness | 4: Patient-reported outcomes | 17: Nervous system outcomes |
| Sore throat | Sore throat | 4: Patient-reported outcomes | 22: Respiratory, thoracic and mediastinal outcomes |
| Presence of pharyngeal pain in the post-anaesthesia care unit | Sore throat | 4: Patient-reported outcomes | 22: Respiratory, thoracic and mediastinal outcomes |
| Severity of pharyngeal irritation the next day | Sore throat | 4: Patient-reported outcomes | 22: Respiratory, thoracic and mediastinal outcomes |
| Upper airway discomfort | Sore throat | 4: Patient-reported outcomes | 22: Respiratory, thoracic and mediastinal outcomes |
| Sore throat | Sore throat | 4: Patient-reported outcomes | 22: Respiratory, thoracic and mediastinal outcomes |
| Sore throat | Sore throat | 4: Patient-reported outcomes | 22: Respiratory, thoracic and mediastinal outcomes |
| Postoperative sore throat | Sore throat | 4: Patient-reported outcomes | 22: Respiratory, thoracic and mediastinal outcomes |
| Pharyngeal pain / Sore throat | Sore throat | 4: Patient-reported outcomes | 22: Respiratory, thoracic and mediastinal outcomes |
| Sore throat | Sore throat | 4: Patient-reported outcomes | 22: Respiratory, thoracic and mediastinal outcomes |
| Sore throat | Sore throat | 4: Patient-reported outcomes | 22: Respiratory, thoracic and mediastinal outcomes |
| Sore throat | Sore throat | 4: Patient-reported outcomes | 22: Respiratory, thoracic and mediastinal outcomes |
| Postoperative sore throat | Sore throat | 4: Patient-reported outcomes | 22: Respiratory, thoracic and mediastinal outcomes |
| Sore throat | Sore throat | 4: Patient-reported outcomes | 22: Respiratory, thoracic and mediastinal outcomes |
| Sore throat | Sore throat | 4: Patient-reported outcomes | 22: Respiratory, thoracic and mediastinal outcomes |
| Postoperative sore throat | Sore throat | 4: Patient-reported outcomes | 22: Respiratory, thoracic and mediastinal outcomes |
| Sore throat | Sore throat | 4: Patient-reported outcomes | 22: Respiratory, thoracic and mediastinal outcomes |
| Pharyngo-laryngeal complaints | Sore throat | 4: Patient-reported outcomes | 22: Respiratory, thoracic and mediastinal outcomes |
| Sore throat | Sore throat | 4: Patient-reported outcomes | 22: Respiratory, thoracic and mediastinal outcomes |
| Sore throat | Sore throat | 4: Patient-reported outcomes | 22: Respiratory, thoracic and mediastinal outcomes |
| Sore throat | Sore throat | 4: Patient-reported outcomes | 22: Respiratory, thoracic and mediastinal outcomes |
| Sore throat | Sore throat | 4: Patient-reported outcomes | 22: Respiratory, thoracic and mediastinal outcomes |
| Sore throat | Sore throat | 4: Patient-reported outcomes | 22: Respiratory, thoracic and mediastinal outcomes |
| Vomiting | Vomiting | 4: Patient-reported outcomes | 8: Gastrointestinal outcomes |
| Acoustic voice analysis | Acoustic voice analysis | 5: Other outcomes | 9: General outcomes |
| Admission to ICU | Admission to ICU | 5: Other outcomes | 35: Hospital |
| Airway care score | Airway care score | 5: Other outcomes | 32: Delivery of care |
| Apnoea time | Apnoea time | 5: Other outcomes | 22: Respiratory, thoracic and mediastinal outcomes |
| Non-hypoxic apnoea time | Apnoea time | 5: Other outcomes | 22: Respiratory, thoracic and mediastinal outcomes |
| Auscultation | Auscultation | 5: Other outcomes | 22: Respiratory, thoracic and mediastinal outcomes |
| Laryngoscope blades used | Blade used | 5: Other outcomes | 32: Delivery of care |
| Degree of cervical spine motion | Cervical spine motion | 5: Other outcomes | 15: Musculoskeletal and connective tissue outcomes |
| Chest tube duration | Chest tube treatment duration | 5: Other outcomes | 22: Respiratory, thoracic and mediastinal outcomes |
| Interruption of cricoid pressure | Cricoid pressure | 5: Other outcomes | 32: Delivery of care |
| Extubation responses | Extubation response | 5: Other outcomes | 22: Respiratory, thoracic and mediastinal outcomes |
| Gastroenterologist’s feedback | Feedback from other clinician | 5: Other outcomes | 32: Delivery of care |
| Intensity of lifting force applied during laryngoscopy | Force during laryngoscopy | 5: Other outcomes | 32: Delivery of care |
| Amount and pH of gastric aspirate | Gastric aspirate | 5: Other outcomes | 8: Gastrointestinal outcomes |
| Gastric insufflation | Gastric insufflation | 5: Other outcomes | 8: Gastrointestinal outcomes |
| Gastric inflation | Gastric insufflation | 5: Other outcomes | 8: Gastrointestinal outcomes |
| Gastroesophageal drainage tub position | Gastric tube position | 5: Other outcomes | 8: Gastrointestinal outcomes |
| Global inhomogeneity | Global inhomogeneity | 5: Other outcomes | 9: General outcomes |
| Global rating scale score | Global rating scale score | 5: Other outcomes | 9: General outcomes |
| Head and neck flexion or extension | Head and neck position | 5: Other outcomes | 32: Delivery of care |
| Head and neck position | Head and neck position | 5: Other outcomes | 32: Delivery of care |
| HEAVEN criteria | HEAVEN criteria | 5: Other outcomes | 9: General outcomes |
| Rate of hospital admission | Hospital admission | 5: Other outcomes | 35: Hospital |
| ICU length of stay | ICU length of stay | 5: Other outcomes | 35: Hospital |
| ICU-free days within the first28-days since intubation | ICU length of stay | 5: Other outcomes | 35: Hospital |
| Intubation episode | Intubation episode | 5: Other outcomes | 9: General outcomes |
| Intubation supervision rate | Intubation supervision rate | 5: Other outcomes | 32: Delivery of care |
| Intubation technique | Intubation technique | 5: Other outcomes | 32: Delivery of care |
| Intubator experience | Intubator experience | 5: Other outcomes | 32: Delivery of care |
| Expert intubator | Intubator experience | 5: Other outcomes | 32: Delivery of care |
| Novice intubator | Intubator experience | 5: Other outcomes | 32: Delivery of care |
| Length of hospital stay | Length of hospital stay | 5: Other outcomes | 35: Hospital |
| Length of ICU stay | Length of ICU stay | 5: Other outcomes | 35: Hospital |
| Apgar score | Neonatal outcomes | 5: Other outcomes | 20: Reproductive system and breast outcomes |
| Neonatal weight | Neonatal outcomes | 5: Other outcomes | 20: Reproductive system and breast outcomes |
| Umbilical venous pH | Neonatal outcomes | 5: Other outcomes | 20: Reproductive system and breast outcomes |
| Node resection | Node resection | 5: Other outcomes | 9: General outcomes |
| Operative time | Operative time | 5: Other outcomes | 32: Delivery of care |
| Subjective endpoints | Operator evaluation | 5: Other outcomes | 32: Delivery of care |
| Subjective endpoints of device evaluation (easy to learn, easy to use) | Operator evaluation | 5: Other outcomes | 32: Delivery of care |
| Patient related factors (haemodynamics, complications) | Other | 5: Other outcomes | 9: General outcomes |
| pH of SLMA laryngeal surface | pH of device surface | 5: Other outcomes | 32: Delivery of care |
| Phonetic analysis | Phonetic analysis | 5: Other outcomes | 9: General outcomes |
| Place of intervention | Place of intervention | 5: Other outcomes | 9: General outcomes |
| Jaw opening | Predictors of difficulty | 5: Other outcomes | 32: Delivery of care |
| Anticipated difficult airway | Predictors of difficulty | 5: Other outcomes | 32: Delivery of care |
| Thyromental distance | Predictors of difficulty | 5: Other outcomes | 32: Delivery of care |
| Airway assessment | Predictors of difficulty | 5: Other outcomes | 32: Delivery of care |
| Airway assessment | Predictors of difficulty | 5: Other outcomes | 32: Delivery of care |
| Thyromental distance | Predictors of difficulty | 5: Other outcomes | 32: Delivery of care |
| Modified Mallampati | Predictors of difficulty | 5: Other outcomes | 32: Delivery of care |
| Sternomental distance | Predictors of difficulty | 5: Other outcomes | 32: Delivery of care |
| Thyromental distance | Predictors of difficulty | 5: Other outcomes | 32: Delivery of care |
| High Mallampati | Predictors of difficulty | 5: Other outcomes | 32: Delivery of care |
| Low Mallampati | Predictors of difficulty | 5: Other outcomes | 32: Delivery of care |
| Proximal-cuff-to-tip distance | Proximal-cuff-to-tip distance | 5: Other outcomes | 9: General outcomes |
| Appropriate removal of rigid stylet | Removal of adjunct | 5: Other outcomes | 32: Delivery of care |
| Rate of return of spontaneous circulation | Return of spontaneous circulation | 5: Other outcomes | 3: Cardiac outcomes |
| Time of intervention (night, weekend) | Time of intervention | 5: Other outcomes | 32: Delivery of care |
| Invasive ventilator-free days within the first 28-days since intubation | Ventilator-free days | 5: Other outcomes | 35: Hospital |
| Intubations performed with VL as a primary or rescue technique | Videolaryngoscopy as primary approach | 5: Other outcomes | 32: Delivery of care |

Table S2 Longlisted outcomes

| Adjunct use | Haemodynamic response |
| --- | --- |
| Airway manipulation and repositioning | Heart rate |
| Airway obstruction | Hoarseness |
| Airway trauma | Hypertension |
| Apnoea time | Hypotension |
| Arrhythmia | Hypoxaemia |
| Blood loss | ICU length of stay |
| Blood pressure | Intubation difficulty |
| Cardiac arrest | Nausea |
| Change of device | Need for further intervention |
| Change of operator | Neurological complications |
| Complications | Neuropraxia |
| Correct device placement | Number of attempts |
| Coughing | Oesophageal intubation |
| Death | Operator evaluation |
| Dental trauma | Oropharyngeal trauma |
| Depth of anaesthesia | Overall success |
| Device function | Oxygen saturation |
| Difficult intubation | Oxygenation |
| Difficult laryngoscopy | Pain |
| Dysphonia | Patient satisfaction |
| Ease of device use | Predictors of difficulty |
| Ease of intubation | Pulmonary aspiration |
| Ease of laryngoscopy | Respiratory rate |
| Ease of procedure | Restlessness |
| Effectiveness of ventilation | Second attempt success |
| Endobronchial intubation | Sore throat |
| Failed attempt | Successful procedure without complications |
| Failed device insertion | Time required to establish airway |
| Failed intubation | Time to intubation |
| First attempt success | Time to optimal glottic view |
| First attempt success without complications | Time to successful device insertion |
| Gastric insufflation | Unrecognised oesophageal intubation |
| Glottic view | Vocal cord position |

Table S3 Modifications to outcomes following survey rounds

| **Original wording following Round 2 survey** | **Amended wording** | **Rationale** |
| --- | --- | --- |
| Accidental awareness under general anaesthesia | Accidental awareness during general anaesthesia | Renamed to Accidental awareness during general anaesthesia |
| Airway obstruction | Effectiveness of ventilation | Amalgamated into effectiveness of ventilation |
| Blood pressure | Dropped | Dropped as captured by hypotension and hypertension |
| Correct device placement | Ease of procedure | Amalgamated into ease of procedure |
| Depth of anaesthesia | Dropped | Captured by awareness, therefore dropped |
| Difficult intubation | Difficult airway | Reframed as difficult airway to ensure broader applicability |
| Difficult laryngoscopy | Difficult airway | Amalgamated into difficult airway |
| Ease of device use | Ease of procedure | Amalgamated into ease of procedure |
| Ease of intubation | Ease of procedure | Amalgamated into ease of procedure |
| Ease of laryngoscopy | Ease of procedure | Amalgamated into ease of procedure |
| Endobronchial intubation | Bronchial intubation | Renamed to more specific term |
| Failed attempt | Overall success | Inverted to overall success (successful procedure) |
| Failed device insertion | Overall success | Inverted to overall success (successful procedure) |
| Failed intubation | Failed tracheal intubation | Renamed to more specific term |
| Haemodynamic response | Dropped | Dropped as captured by hypotension and hypertension |
| Heart rate | Dropped | Dropped as not an outcome, rather a haemodynamic variable |
| ICU length of stay | ICU admission | Renamed to ICU admission as prerequisite - original outcome specific to patients already admitted to ICU |
| Intubation difficulty | Difficult airway | Amalgamated into difficult airway |
| Neuropraxia | Neurological complications | Amalgamated into neurological complications |
| Oesophageal intubation | Unrecognised oesophageal intubation | Merged with unrecognised oesophageal intubation as more clinically relevant |
| Operator evaluation | Dropped | Dropped as vague outcome definition and captured by ease of procedure |
| Oropharyngeal trauma | Airway trauma | Amalgamated with airway trauma |
| Oxygen saturation | Oxygenation | Amalgamated with oxygenation |
| Pain | Pain related to airway management | Renamed for specificity to airway management |
| Post-operative cardiac complications | Cardiac complications | Removed postoperative as airway management can occur outside the peri-operative setting |
| Post-operative pulmonary complications | Pulmonary complications | Removed postoperative as airway management can occur outside the peri-operative setting |
| Predictors of difficulty | Dropped | Dropped as not an outcome, rather a predictor variable |
| Second attempt success | Number of attempts | Amalgamated into number of attempts |
| Successful procedure without complications | Overall success without complications | Renamed to align with Overall success outcome |
| Time to device competency | Device competency | Removed time from term as most often not assessed by time, rather the number of procedures required |
| Time to intubation | Time required to establish airway | Amalgamated into Time required to establish airway |
| Time to optimal glottic view | Time to glottic view | Renamed as term 'optimal' too subjective |
| Time to successful device insertion | Time required to establish airway | Amalgamated into Time required to establish airway |

Table S4 Virtual consensus panel voting results for individual outcomes

| **Outcome domain** | **Outcome** | **Stakeholder decision in previous round** | **Steering Committee suggestion** | **Panel votes** | **Decision** |
| --- | --- | --- | --- | --- | --- |
| Adverse events | Death | **Include** | **Include** | **100%** | **Include** |
| Adverse events | Cardiac arrest | **Include** | **Include** | **98%** | **Include** |
| Adverse events | Complications | **Include** | **Include** | **85%** | **Include** |
| Adverse events | Pulmonary complications | **Include** | **Include** | **92%** | **Include** |
| Adverse events | Neurological complications | **Include** | **Include** | **77%** | **Include** |
| Adverse events | Airway trauma | **Include** | **Include** | **92%** | **Include** |
| Adverse events | Unrecognised oesophageal intubation | **Include** | **Include** | **89%** | **Include** |
| Adverse events | Pulmonary aspiration | **Include** | **Include** | **89%** | **Include** |
| Procedural success | First attempt success without complications | **Include** | **Include** | **98%** | **Include** |
| Procedural success | Overall success without complications | **Include** | **Include** | **82%** | **Include** |
| Procedural success | Difficult airway | **Include** | **Include** | **95%** | **Include** |
| Other | Hypoxaemia | **Include** | **Include** | **91%** | **Include** |
| Adverse events | Cardiac complications | **Indeterminate** | **Include** | **59%** | **Exclude** |
| Adverse events | Dental trauma | **Indeterminate** | **Include** | **45%** | **Exclude** |
| Adverse events | Bronchial intubation | **Indeterminate** | **Include** | **40%** | **Exclude** |
| Adverse events | Arrhythmia | **Indeterminate** | **Include** | **32%** | **Exclude** |
| Adverse events | Hypertension | **Indeterminate** | **Include** | **21%** | **Exclude** |
| Adverse events | Hypotension | **Indeterminate** | **Include** | **49%** | **Exclude** |
| Adverse events | Accidental awareness during general anaesthesia | **Indeterminate** | **Exclude** | **7%** | **Exclude** |
| Adverse events | Dysphonia | **Indeterminate** | **Exclude** | **18%** | **Exclude** |
| Adverse events | Front-of-neck airway | **Include** | **Exclude** | **49%** | **Exclude** |
| Adverse events | ICU admission | **Indeterminate** | **Exclude** | **27%** | **Exclude** |
| Adverse events | Blood loss | **Indeterminate** | **Exclude** | **2%** | **Exclude** |
| Procedural success | First attempt success | **Include** | **Exclude** | **52%** | **Exclude** |
| Procedural success | Overall success | **Include** | **Exclude** | **48%** | **Exclude** |
| Procedural success | Number of attempts | **Include** | **Exclude** | **48%** | **Exclude** |
| Procedural success | Failed tracheal intubation | **Include** | **Exclude** | **32%** | **Exclude** |
| Procedural success | Need for further intervention | **Include** | **Exclude** | **11%** | **Exclude** |
| Procedural success | Glottic view | **Indeterminate** | **Exclude** | **39%** | **Exclude** |
| Procedural success | Time required to establish airway | **Include** | **Exclude** | **19%** | **Exclude** |
| Procedural success | Time to glottic view | **Indeterminate** | **Exclude** | **13%** | **Exclude** |
| Procedural success | Device competency | **Indeterminate** | **Exclude** | **0%** | **Exclude** |
| Procedural success | Change of operator | **Indeterminate** | **Exclude** | **9%** | **Exclude** |
| Procedural success | Change of device | **Include** | **Exclude** | **16%** | **Exclude** |
| Procedural success | Ease of procedure | **Include** | **Exclude** | **2%** | **Exclude** |
| Procedural success | Device function | **Include** | **Exclude** | **0%** | **Exclude** |
| Procedural success | Vocal cord position | **Indeterminate** | **Exclude** | **0%** | **Exclude** |
| Procedural success | Airway manipulation and repositioning | **Indeterminate** | **Exclude** | **5%** | **Exclude** |
| Procedural success | Adjunct use | **Indeterminate** | **Exclude** | **11%** | **Exclude** |
| Patient-reported outcomes | Pain related to airway management | **Indeterminate** | **Exclude** | **5%** | **Exclude** |
| Patient-reported outcomes | Patient satisfaction | **Indeterminate** | **Include** | **28%** | **Exclude** |
| Other | Oxygenation | **Include** | **Exclude** | **9%** | **Exclude** |
| Other | Apnoea time | **Indeterminate** | **Exclude** | **11%** | **Exclude** |
| Other | Effectiveness of ventilation | **Include** | **Exclude** | **20%** | **Exclude** |

Table S5 Voting results on proposed modifications to included outcomes

| **Outcome** | Death | | | |
| --- | --- | --- | --- | --- |
| **Vote** | All-cause vs airway-related mortality | | | |
| **Total votes** | 28 | | | |
| **Responses** | **Airway-related** | All-cause |  |  |
|  | **21** | 7 |  |  |
| **Summary** | **75%** | 25% |  |  |
| **Recommendation** | **Strong** | | | |

| **Outcome** | Death | | | |
| --- | --- | --- | --- | --- |
| **Vote** | We do not recommend a specific timepoint for mortality in this outcome set | | | |
| **Total votes** | 30 | | | |
| **Responses** | **Agree** | Disagree |  |  |
|  | **27** | 3 |  |  |
| **Summary** | **90%** | 10% |  |  |
| **Recommendation** | **Strong** | | | |

| **Outcome** | Cardiac arrest | | | |
| --- | --- | --- | --- | --- |
| **Vote** | Timing: cardiac arrest within 24 hours of airway management event | | | |
| **Total votes** | 31 | | | |
| **Responses** | **Yes** | No |  |  |
|  | **16** | 15 |  |  |
| **Summary** | **52%** | 48% |  |  |
| **Recommendation** | **Interim recommendation – further research required** | | | |

| **Outcome** | Complications | | | |
| --- | --- | --- | --- | --- |
| **Vote** | Change to serious complications (definition as stated) or remove and merge into domain (adverse events) | | | |
| **Total votes** | 35 | | | |
| **Responses** | **Change to Serious complications** | Remove and merge into domain | Neither |  |
|  | **25** | 8 | 2 |  |
| **Summary** | **71%** | 23% | 6% |  |
| **Recommendation** | **Interim recommendation – further research required** | | | |

| **Outcome** | Neurological complications | | | |
| --- | --- | --- | --- | --- |
| **Vote** | Remove peripheral nerve palsy from definition | | | |
| **Total votes** | 35 | | | |
| **Responses** | **Yes** | No |  |  |
|  | **28** | 7 |  |  |
| **Summary** | **80%** | 20% |  |  |
| **Recommendation** | **Strong** | | | |

| **Outcome** | Unrecognised oesophageal intubation | | | |
| --- | --- | --- | --- | --- |
| **Vote** | Definition vote: Unrecognised oesophageal intubation | | | |
| **Total votes** | 36 | | | |
| **Responses** | **Agree** | Disagree |  |  |
|  | **34** | 2 |  |  |
| **Summary** | **94%** | 6% |  |  |
| **Recommendation** | **Strong** | | | |

| **Outcome** | Hypoxaemia | | | |
| --- | --- | --- | --- | --- |
| **Vote** | Definition vote: Hypoxaemia | | | |
| **Total votes** | 36 | | | |
| **Responses** | **Agree** | Disagree |  |  |
|  | **30** | 6 |  |  |
| **Summary** | **83%** | 17% |  |  |
| **Recommendation** | **Strong** | | | |

| **Outcome** | Pulmonary aspiration | | | |
| --- | --- | --- | --- | --- |
| **Vote** | Vote on updated instrument: clinical, radiological or bronchoscopic evidence of entry of gastric contents into trachea and/or lungs | | | |
| **Total votes** | 37 | | | |
| **Responses** | **Yes** | No |  |  |
|  | **37** | 0 |  |  |
| **Summary** | **100%** |  |  |  |
| **Recommendation** | **Strong** | | | |

| **Outcome** | Pulmonary aspiration | | | |
| --- | --- | --- | --- | --- |
| **Vote** | Definition vote: Propose to combine with 'Pulmonary complications' | | | |
| **Total votes** | 37 | | | |
| **Responses** | Agree | Disagree |  |  |
|  | 32 | 5 |  |  |
| **Summary** | 86% | 14% |  |  |
| **Recommendation** | **Strong** | | | |

| **Outcome** | First attempt success without complications | | | |
| --- | --- | --- | --- | --- |
| **Vote** | First attempt success without complications time cut-off (90 seconds vs 120 seconds) | | | |
| **Total votes** | 33 | | | |
| **Responses** | **120 seconds** | 90 seconds |  |  |
|  | **22** | 11 |  |  |
| **Summary** | **67%** | 33% |  |  |
| **Recommendation** | **Interim recommendation – further research required** | | | |

| **Outcome** | First attempt success without complications | | | |
| --- | --- | --- | --- | --- |
| **Vote** | Definition vote: First attempt success without complications | | | |
| **Total votes** | 31 | | | |
| **Responses** | **Agree** | Disagree |  |  |
|  | **26** | 5 |  |  |
| **Summary** | **84%** | 16% |  |  |
| **Recommendation** | **Strong** | | | |

| **Outcome** | Overall success without complications | | | |
| --- | --- | --- | --- | --- |
| **Vote** | Definition vote: Overall success without complications | | | |
| **Total votes** | 33 | | | |
| **Responses** | **Agree** | Disagree |  |  |
|  | **32** | 1 |  |  |
| **Summary** | **97%** | 3% |  |  |
| **Recommendation** | **Strong** | | | |

| **Outcome** | Difficult airway | | | |
| --- | --- | --- | --- | --- |
| **Vote** | Include emergency front-of-neck airway into composite definition | | | |
| **Total votes** | 33 | | | |
| **Responses** | **Agree** | Disagree |  |  |
|  | **32** | 1 |  |  |
| **Summary** | **97%** | 3% |  |  |
| **Recommendation** | **Strong** | | | |

| **Outcome** | Difficult airway | | | |
| --- | --- | --- | --- | --- |
| **Vote** | Definition vote: Difficult airway | | | |
| **Total votes** | 32 | | | |
| **Responses** | **Agree** | Disagree |  |  |
|  | **29** | 3 |  |  |
| **Summary** | **91%** | 9% |  |  |
| **Recommendation** | **Strong** | | | |

| **Outcome** | Difficult airway | | | |
| --- | --- | --- | --- | --- |
| **Vote** | Outcome measurement instrument sub-definition: difficult laryngoscopy – change to | | | |
| **Total votes** | 35 | | | |
| **Responses** | modified Cormack-Lehane ≥2b | **modified Cormack-Lehane ≥2b or vocal cords not visible** | vocal cords not visible |  |
|  | 5 | **17** | 13 |  |
| **Summary** | 14% | **49%** | 37% |  |
| **Recommendation** | **No recommendation can be made** | | | |

Table S6 Virtual consensus panel voting results for outcome measurement instruments

| **Outcome** | **Outcome measurement instrument** | **Total** | **Yes** | **No** | **%** | **Recommendation** |
| --- | --- | --- | --- | --- | --- | --- |
| Death | Death certificate or hospital record; no single timepoint proposed. | 31 | 30 | 1 | 97% | **Strong** |
| Cardiac arrest | Need for five or more chest compressions and/or defibrillation. | 33 | 24 | 9 | 73% | **Interim** |
| Serious complications | No single outcome measurement instrument proposed. | 35 | 32 | 3 | 91% | **Strong** |
| Pulmonary complications | Atelectasis: detected on imaging (e.g. computed tomography, chest radiograph, ultrasound). Pulmonary aspiration: clinical, radiological or bronchoscopic evidence of entry of gastric contents into trachea and/or lungs. Acute respiratory distress syndrome: diagnosed using the 2023 global definition. | 35 | 26 | 9 | 74% | **Interim** |
| Neurological complications | Brain damage: confirmed evidence of hypoxic-ischaemic brain injury following procedure. Spinal cord injury: confirmed clinical diagnosis. | 36 | 34 | 2 | 94% | **Strong** |
| Airway trauma | No single outcome measurement instrument proposed. | 36 | 36 | 0 | 100% | **Strong** |
| Unrecognised oesophageal intubation | No single outcome measurement instrument proposed. | 37 | 35 | 2 | 95% | **Strong** |
| Hypoxaemia | Measured with continuous pulse oximetry for at least 30 minutes following commencement of study intervention. Record the lowest values. Report episodes of hypoxaemia (S_p_O_2_ <90%) and severe hypoxaemia (S_p_O_2_ <80%). | 36 | 34 | 2 | 94% | **Strong** |
| First attempt success without complications | Record whether the airway was successfully established following first instrumentation with device and/or adjunct within 120 seconds with capnography confirmation, in the absence of any complications related to airway management. | 33 | 32 | 1 | 97% | **Strong** |
| Overall success without complications | Record whether the airway was successfully established within three attempts with capnography confirmation, in the absence of any complications related to airway management. | 34 | 33 | 1 | 97% | **Strong** |
| Difficult airway | For any participant undergoing airway management under optimised conditions (operator, position, device), record and report each of the following: difficult facemask ventilation, classified as Grade C or D (Lim classification); difficult supraglottic airway ventilation, defined as at least one failed attempt at supraglottic airway insertion; difficult laryngoscopy, with no single threshold agreed; difficult tracheal intubation, defined as at least one failed attempt at tracheal intubation; difficult front-of-neck airway, defined as at least one failed attempt at front-of-neck airway; need for emergency front-of-neck airway. | 35 | 34 | 1 | 97% | **Strong** |

Table S7 Detailed information on outcomes and outcome measurement instruments

| **Domain** | **Outcome** | **Detailed definition** | **Outcome measurement instrument** | **Comment** |
| --- | --- | --- | --- | --- |
| *Adverse events* | Death | Airway-related mortality | Death certificate or hospital record; no single timepoint proposed | Where investigators are reporting airway-related mortality, an adjudication mechanism (e.g. expert panel) is recommended. All-cause mortality may be reported from registries alone. Timepoints will depend on study context and should be identified by investigators. |
|  | Cardiac arrest | A sudden cessation of function of the heart requiring delivery of five or more chest compressions and/or defibrillation within 24 hours of airway management event | Need for five or more chest compressions and/or defibrillation | This definition aligns with NAP7 [1]. |
|  | Serious complications | An adverse event that results in death, is life-threatening, requires hospitalisation or prolongation of existing hospitalisation, results in persistent or significant disability or incapacity, or is a birth defect | No single outcome measurement instrument proposed | The definition aligns with the Medicines and Healthcare products Regulatory Agency, the European Medicines Agency and the Food and Drug Administration definitions [2-4]. |
|  | Pulmonary complications | Composite outcome of any of the following:   - atelectasis; - pulmonary aspiration; - acute respiratory distress syndrome | Atelectasis: detected on imaging (e.g. computed tomography, chest radiograph, ultrasound)  Pulmonary aspiration: clinical, radiological or bronchoscopic evidence of entry of gastric contents into trachea and/or lungs  Acute respiratory distress syndrome: diagnosed using the 2023 global definition | The definitions align with the StEP-COMPAC group’s peri-operative core outcome set with a change in requirement for imaging verification of aspiration [5] and an updated definition of ARDS [6]. |
|  | Neurological complications | Composite outcome of any new brain damage or spinal cord injury | Brain damage: confirmed evidence of hypoxic-ischaemic brain injury following procedure  Spinal cord injury: confirmed clinical diagnosis | This definition aligns with NAP4 [7]. |
|  | Airway trauma | Injury to any area of the nasal cavity, nasopharynx, oropharynx, pharynx, glottis, subglottis or trachea | No single outcome measurement instrument proposed | Given the heterogeneous clinical manifestations of airway trauma, no single outcome measurement instrument can be proposed. |
|  | Unrecognised oesophageal intubation | Oesophageal placement of a tracheal tube not identified by absence of sustained exhaled carbon dioxide or leading to a complication | No single outcome measurement instrument proposed | Although no single outcome measurement instrument is proposed for research purposes, waveform capnography remains the core modality to exclude oesophageal intubation [8,9]. |
|  | Hypoxaemia | Defined as oxygen saturation <90%, with severe hypoxaemia defined as oxygen saturation <80% | Measured with continuous pulse oximetry for at least 30 minutes following commencement of study intervention. Record the lowest values. Report episodes of hypoxaemia (SpO2 <90%) and severe hypoxaemia (SpO2 <80%). | This definition aligns with the WHO threshold for hypoxaemia and BTS guidelines [10,11]. |
| *Procedural effectiveness* | First attempt success without complications | Composite outcome of first attempt success (successfully established airway following first instrumentation with device and/or adjunct within 120 seconds confirmed with capnography) and absence of complications related to airway management | Record whether the airway was successfully established following first instrumentation with device and/or adjunct within 120 seconds with capnography confirmation, in the absence of any complications related to airway management | First attempt success should be recorded as part of this composite outcome. First instrumentation should be considered when a device and/or adjunct first engages the airway (i.e. passes the lips or makes contact with the patient). Timing should stop at the start of the first confirmatory capnography trace, however, confirmation for clinical purposes should be in line with relevant and current clinical guidelines (sustained exhaled carbon dioxide). |
|  | Overall success without complications | Composite outcome of overall success (successfully established airway within three attempts confirmed with capnography), and absence of complications related to airway management | Record whether the airway was successfully established within three attempts with capnography confirmation, in the absence of any complications related to airway management | We suggest investigators record the number of attempts as part of this composite outcome. First instrumentation should be considered when a device and/or adjunct first engages the airway (i.e. passes the lips or makes contact with the patient). Timing should stop at the start of the first confirmatory capnography trace, however, confirmation for clinical purposes should be in line with relevant and current clinical guidelines (sustained exhaled carbon dioxide). |
|  | Difficult airway | Composite outcome of any of the following:   - difficult facemask ventilation; - difficult supraglottic airway ventilation; - difficult laryngoscopy; - difficult tracheal intubation; - difficult front-of-neck airway; - emergency front-of-neck airway | For any participant undergoing airway management under optimised conditions (operator, position, device), record and report each of the following:   - difficult facemask ventilation, classified as Grade C or D according to the Lim classification; - difficult supraglottic airway ventilation, defined as at least one failed attempt at supraglottic airway insertion; - difficult laryngoscopy, with no single threshold agreed; - difficult tracheal intubation, defined as at least one failed attempt at tracheal intubation; - difficult front-of-neck airway, defined as at least one failed attempt at front-of-neck airway; - need for emergency front-of-neck airway | The panel could not propose a consensus definition for difficult laryngoscopy using a single scale. Investigators may adopt scales such as the Cormack-Lehane or the percentage of glottic opening (POGO) scale, with relevant thresholds for difficulty, depending on study context. |

References

1. Kane AD, Soar J, Armstrong RA, et al. Patient characteristics, anaesthetic workload and techniques in the UK: An analysis from the 7th National Audit Project (NAP7) activity survey. *Anaesthesia* 2023; **78:** 701-11. 10.1111/anae.15989
2. U.S. Food & Drug Administration. What is a serious adverse event? 2025. <https://www.fda.gov/safety/reporting-serious-problems-fda/what-serious-adverse-event> (accessed 22/07/2025).
3. NHS Health Research Authority. Safety and progress reports (other research) procedural table, 2024. <https://www.hra.nhs.uk/approvals-amendments/managing-your-approval/safety-reporting/safety-and-progress-reports-other-research-procedural-table/> (accessed 22/07/2025).
4. European Medicines Agency. Serious adverse reaction, 2025. <https://www.ema.europa.eu/en/glossary-terms/serious-adverse-reaction> (accessed 22/07/2025).
5. Boney O, Moonesinghe SR, Myles PS, Grocott MPW. Core outcome measures for perioperative and anaesthetic care (COMPAC): A modified Delphi process to develop a core outcome set for trials in perioperative care and anaesthesia. *Br J Anaesth* 2022; **128:** 174-85. 10.1016/j.bja.2021.09.027
6. Matthay MA, Arabi Y, Arroliga AC, et al. A new global definition of acute respiratory distress syndrome. *Am J Respir Crit Care Med* 2024; **209:** 37-47. 10.1164/rccm.202303-0558WS
7. Cook TM, Woodall N, Frerk C. Major complications of airway management in the UK: Results of the fourth national audit project of the Royal College of Anaesthetists and the Difficult Airway Society. Part 1: Anaesthesia. *Br J Anaesth* 2011; **106:** 617-31. 10.1093/bja/aer058
8. Chrimes N, Higgs A, Hagberg CA, et al. Preventing unrecognised oesophageal intubation: A consensus guideline from the project for universal management of airways and international airway societies. *Anaesthesia* 2022; **77:** 1395-415. 10.1111/anae.15817
9. Hansel J, Law JA, Chrimes N, Higgs A, Cook TM. Clinical tests for confirming tracheal intubation or excluding oesophageal intubation: A diagnostic test accuracy systematic review and meta-analysis. *Anaesthesia* 2023; **78:** 1020-30. 10.1111/anae.16059
10. O'Driscoll BR, Howard LS, Earis J, Mak V. British thoracic society guideline for oxygen use in adults in healthcare and emergency settings. *BMJ Open Respir Res* 2017; **4:** e000170. 10.1136/bmjresp-2016-000170
11. World Health Organization. Clinical care of severe acute respiratory infections – tool kit, 2022. <https://www.who.int/publications/i/item/clinical-care-of-severe-acute-respiratory-infections-tool-kit> (accessed 22/07/2025).

Table S8 Plain language summary of included outcomes

| **Outcome** | **Plain language outcome** | **Plain language detailed explanation** |
| --- | --- | --- |
| Death | Death | Death following an airway event. |
| Cardiac arrest | Heart stops beating | The heart stops beating which then requires more than 5 sets of chest compressions (CPR) or the use of a defibrillator. |
| Serious complications | Serious complications | A serious complication that either leads to death, or a hospital stay, or a longer than expected hospital stay. The patient is left with a significant disability or incapacity or has a birth defect. |
| Pulmonary complications | Complications relating to the lungs | Composite outcome of any of the following:   - atelectasis: a blockage of the air passages causing the collapse of part of the lung; - pulmonary aspiration: contamination of the lung after inhaling liquid or matter; - acute respiratory distress syndrome: a combination of symptoms including difficulty breathing, low levels of oxygen, signs of poor circulation. |
| Neurological complications | Injuries to the brain or nervous system | Evidence of brain injury or injury to the nervous system such as paralysis or lack of speech. |
| Airway trauma | Injury to the airway | Injury to any part of the airway from the nose, through the mouth and voice box down to the trachea (windpipe). |
| Unrecognised oesophageal intubation | Failure to notice that the tracheal tube has been placed in the oesophagus | Failing to notice that the level of carbon dioxide leaving the body does not confirm that the tracheal tube has been placed correctly, and that it has been placed in the oesophagus in error. |
| Hypoxaemia | Low levels of oxygen in the blood | Readings from a monitor that show low levels of oxygen present in the blood. Less than 90% oxygen is of concern, less than 80% oxygen is considered to be severe. |
| First attempt success without complications | The ability to successfully insert the airway device at the first attempt without having any complications | The first attempt at inserting an airway device, within 120 seconds of the start of the process, with successful insertion confirmed by a continuous measurement of the level of carbon dioxide indicated on the monitor, without having experienced any complications whilst undertaking the procedure. |
| Overall success without complications | A successful insertion of an airway device without experiencing any complications | The overall success of the airway procedure, within three attempts at insertion of the airway device. Successful insertion confirmed by a continuous measurement of the level of carbon dioxide indicated on the monitor. No additional complications relating to airway management recorded. |
| Difficult airway | The process of inserting an airway device being identified as being difficult by the airway manager | Composite outcome of any of the following:   - difficult facemask ventilation: difficulty in providing ventilation using a facemask; - difficult supraglottic airway ventilation: difficulty in insertion of a specific airway device, known as a supraglottic airway; these sit above the voicebox rather than a tube in the trachea (windpipe); - difficult laryngoscopy: difficulty experienced when examining the voice box and any other surrounding structures before placing a tube; - difficult tracheal intubation: difficulty with at least one attempt at inserting the tracheal tube into the trachea (windpipe); - difficult front-of-neck airway: difficulty in establishing an airway through the front of the patient’s neck; - emergency front-of-neck airway: the need for the patient to have an emergency procedure to ensure that an airway can be made in the front of the patient’s neck. |
